# Functional Morphology and Morphological Diversification of Hind Limb Cross-Sectional Traits in Mustelid Mammals

**DOI:** 10.1093/iob/obz032

**Published:** 2020-01-08

**Authors:** P Parsi-Pour, B M Kilbourne

**Affiliations:** 1 Institut für Biologie, Humboldt-Universität zu Berlin, Philippstraße 13, 10115 Berlin, Germany; 2 Museum für Naturkunde Berlin, Leibniz Institut für Evolutions- und Biodiversitätsforschung, Invalidenstraße 43, 10115 Berlin, Germany

## Abstract

Locomotor habits in mammals are strongly tied to limb bones’ lengths, diameters, and proportions. By comparison, fewer studies have examined how limb bone cross-sectional traits relate to locomotor habit. Here, we tested whether climbing, digging, and swimming locomotor habits reflect biomechanically meaningful differences in three cross-sectional traits rendered dimensionless— cross-sectional area (CSA), second moments of area (SMA), and section modulus (MOD)—using femora, tibiae, and fibulae of 28 species of mustelid. CSA and SMA represent resistance to axial compression and bending, respectively, whereas MOD represents structural strength. Given the need to counteract buoyancy in aquatic environments and soil’s high density, we predicted that natatorial and fossorial mustelids have higher values of cross-sectional traits. For all three traits, we found that natatorial mustelids have the highest values, followed by fossorial mustelids, with both of these groups significantly differing from scansorial mustelids. However, phylogenetic relatedness strongly influences diversity in cross-sectional morphology, as locomotor habit strongly correlates with phylogeny. Testing whether hind limb bone cross-sectional traits have evolved adaptively, we fit Ornstein–Uhlenbeck (OU) and Brownian motion (BM) models of trait diversification to cross-sectional traits. The cross-sectional traits of the femur, tibia, and fibula appear to have, respectively, diversified under a multi-rate BM model, a single rate BM model, and a multi-optima OU model. In light of recent studies on mustelid body size and elongation, our findings suggest that the mustelid body plan—and perhaps that of other mammals—is likely the sum of a suite of traits evolving under different models of trait diversification.

## Synopsis

Die Fortbewegungsarten von Säugetieren sind stark an Länge, Durchmesser und Proportionen der Extremitätenknochen gebunden. Nur einzelne Studien haben bisher untersucht, wie sich die Querschnittsmerkmale des Extremitätenknochens auf den Bewegungsapparat auswirken. Hier haben wir getestet, ob Kletter-, Grab- und Schwimmgewohnheiten des Bewegungsapparates biomechanisch sinnvolle Unterschiede in drei dimensionslosen Querschnittsmerkmalen widerspiegeln: Querschnittsfläche (CSA), Flächenträgheitsmoment (SMA) und Widerstandsmoment (MOD). Untersucht wurden Femora, Tibiae und Fibulae von 28 Mustelidenarten. CSA und SMA stellen die Beständigkeit gegen axiale Kompression und Biegung dar, wohingegen MOD die strukturelle Festigkeit darstellt. Angesichts der Notwendigkeit, dem Auftrieb in Gewässern und der hohen Dichte des Bodens entgegenzuwirken, besagte unsere Ausgangshypothese, dass schwimmende und grabende Musteliden höhere Werte in den Querschnittsmerkmalen aufweisen. Für alle drei Merkmale haben wir festgestellt, dass schwimmende Musteliden die höchsten Werte zeigen, gefolgt von grabenden Musteliden, wobei sich beide Gruppen signifikant von kletternden Musteliden unterscheiden. Die phylogenetische Verwandtschaft der untersuchten Arten beeinflusst jedoch die Vielfalt in der Querschnittsmorphologie stark, da die Fortbewegungsart stark mit der Phylogenie korreliert. Wenn wir testen, ob die Querschnittsmerkmale des Hinterbeinknochens adaptiv evolviert haben, passen wir die Modelle Ornstein-Uhlenbeck (OU) und Brownian Motion (BM) zur Diversifizierung der Merkmale an die Querschnittsmerkmale an. Die Querschnittsmerkmale von Femora, Tibiae und Fibulae scheinen sich unter einem Multi-Rate BM-Modell, einem Single-Rate BM-Modell und einem Multi-Optima OU-Modell diversifiziert zu haben. Im Lichte neuerer Studien über Größe und Dehnungskapazität des musteliden Körpers deuten unsere Ergebnisse darauf hin, dass der mustelide Bauplan—und vielleicht auch der anderer Säugetiere—wahrscheinlich die Summe einer Reihe von Merkmalen ist, die sich unter verschiedenen Modellen der Diversifizierung der Merkmale entwickeln. Translated to German by the authors with assistance from Constanze Bickelmann, Christiane Funk, and John Nyakatura.

Morfologia funcional e diversificação morfológica dos tratos seccionais ósseos dos membros posteriores de mamíferos mustelídeos (Functional morphology and morphological diversification of hindlimb bone cross-sectional traits in mustelid mammals)Os hábitos locomotores em mamíferos são fortemente associados ao comprimento, diâmetro e proporções dos ossos que formam os membros. Comparativamente, poucos estudos examinaram como os tratos seccionais dos ossos dos membros são relacionadas com o hábito locomotor. Aqui, testamos se os hábitos locomotores de escalada, escavação e natação refletem significantemente nas diferenças biomecanicas em três tratos seccionais obtidos sem dimensão – área de seção transversal (CSA), segundo momento de área (SMA) e módulo de seção (MOD) - usando o femur, tíbia e fíbula de 28 espécies de mustelídeos. CSA e SMA representam resistência à compressão e flexão axial, respectivamente, enquanto MOD representa resistência estrutural. Dada a necessidade de neutralizar a flutuabilidade em ambientes aquáticos e a alta densidade do solo, previmos que as mustelídeos natatoriais e fossoriais tenham maiores valores de tratos seccionais. Para os três tratos, descobrimos que os mustelídeos natatórios têm os valores mais altos, seguidos pelos mustelídeos fossoriais, com ambos grupos diferindo significativamente dos mustelídeos escaladores. No entanto, relações filogenéticas influenciam fortemente a diversidade na morfologia seccional, uma vez que o hábito locomotor se correlaciona fortemente com a filogenia. Testando se os traços seccionais dos ossos dos membros posteriores evoluíram adaptativamente, ajustamos os modelos de diversificação de traços de Ornstein-Uhlenbeck (OU) e movimento browniano (BM) aos traços seccionais. As características seccionais do fêmur, tíbia e fíbula parecem ter se diversificado, respectivamente, sob um modelo de BM com múltiplas taxas, um modelo de BM com taxa única e um modelo de UO multi-optimo. À luz de estudos recentes sobre tamanho e alongamento do corpo de mustelídeos, nossas descobertas sugerem que o plano de corpo de mustelídeos—e talvez o de outros mamíferos—é provavelmente a soma de um conjunto de características que evoluem sob diferentes modelos de traços de diversificação.Translated to Portuguese by Diego Vaz (dbistonvaz@vims.edu)

Morfología funcional y diversificación morfológica de los rasgos transversales del hueso de la extremidad posterior en mamíferos mustélidos (Functional morphology and morphological diversification of hindlimb bone cross-sectional traits in mustelid mammals)Los hábitos locomotores en los mamíferos están fuertemente ligados a las longitudes, diámetros y proporciones de los huesos de las extremidades. Comparativamente, menos estudios han examinado cómo los rasgos de la sección transversal del hueso de la extremidad se relacionan con el hábito locomotor. Aquí, probamos si los hábitos locomotores de escalada, excavación y natación reflejan diferencias biomecánicamente significativas en tres rasgos transversales que se convierten en un área de sección transversal adimensional (CSA), segundo momento de área (SMA) y módulo de sección (MOD). Esto usando fémures, tibias y peronés de 28 especies de mustélidos. CSA y SMA representan resistencia a la compresión axial y la flexión, respectivamente, mientras que MOD representa la resistencia estructural. Dada la necesidad de contrarrestar la flotabilidad en ambientes acuáticos y la alta densidad del suelo, predijimos que los mustélidos natatorios y fosforiales tienen valores más altos de rasgos de sección transversal. Para los tres rasgos, encontramos que los mustélidos nadadores tienen los valores más altos, seguidos por los mustélidos fosoriales, y ambos grupos difieren significativamente de los mustélidos trepadores. Sin embargo, la relación filogenética influye fuertemente en la diversidad de la morfología transversal, ya que el hábito locomotor se correlaciona fuertemente con la filogenia. Al probar si los rasgos de la sección transversal del hueso de la extremidad posterior han evolucionado de forma adaptativa, ajustamos los modelos de diversificación de rasgos de Ornstein-Uhlenbeck (OU) y movimiento Browniano (BM) a los rasgos de sección transversal. Los rasgos de sección transversal del fémur, la tibia y el peroné parecen haberse diversificado respectivamente bajo un modelo de BM de tasa múltiple, un modelo de BM de tasa única y un modelo de OU multi-optimo. A la luz de los estudios recientes sobre el tamaño y el alargamiento del cuerpo de los mustélidos, nuestros hallazgos sugieren que el plan del cuerpo de los mustélidos, y tal vez el de otros mamíferos, es probablemente la suma de un conjunto de rasgos que evolucionan bajo diferentes modelos de diversificación de rasgos.Translated to Spanish by J Heras (herasj01@gmail.com)

## Introduction

**Fig. 1:**
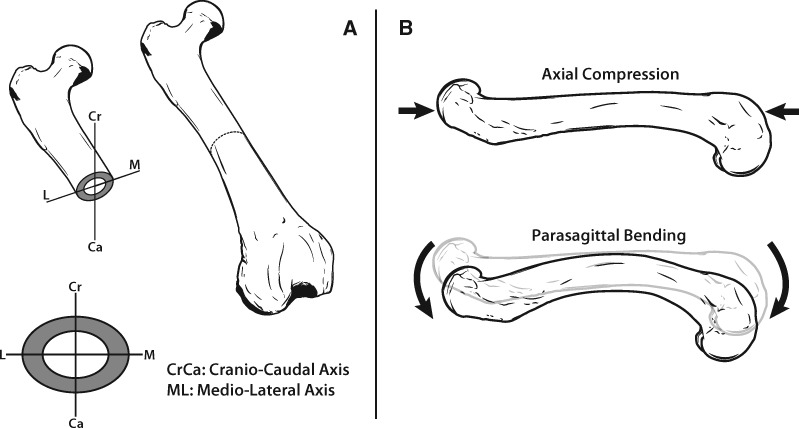
A bone cross-section as it relates to compression and bending loading regimes. (**A**), A femur in cross-section, with the bone tissue within the section being shown in gray. Two anatomical axes were applied to the cross-section: cranio-caudal (CC) and medio-lateral (ML). The total bone tissue within the cross-section corresponds to the bone’s CSA, whereas the distribution of bone tissue about the anatomical axes corresponds to the bone’s SMA. (**B**) CSA and SMA as related to the two of the primary loading regimes encountered by limb bones. CSA represents resistance to forces causing axial compression along the length of the bone, whereas SMA represents resistance to bending moments about a given anatomical axis. SMA_ML_ is associated with bending about the ML axis (i.e., bending in the CC plane), whereas SMACC is associated with bending about the CC axis (i.e., bending in the ML plane). The farther bone tissue is from an axis of bending, the greater value of SMA and the greater the resistance to bending. Thus, in this figure, SMACC is greater than SMA_ML_ due to the oblong shape of the cross-section depicted in (**A**). The example of bending is exaggerated for illustrative purposes, and parasagittal bending occurs about the mediolateral axis which would project perpendicularly out of the page in this example. All cross-sectional traits were measured with BoneJ 1.4.2 (Doube et al., 2010).

One of the fundamental roles of the tetrapod limb skeleton is to withstand the mechanical loads incurred by the limb during locomotion and limb function. Notably, the morphological traits associated with a bone’s cross-sectional geometry determine the bone’s ability to resist specific loading regimes (Beer et al. 2001). A bone’s cross-sectional area (CSA) determines its resistance to axial compression and represents the total amount of bone tissue in the cross-section, whereas the second moment of area (SMA) determines its resistance to bending and represents the distribution of bone tissue about a specific axis of bending (Fig. 1). A bone’s section modulus (MOD) represents its structural strength stemming from its cross-sectional shape. Greater values of MOD increase bending strength relative to bending stress. Though adaptations in bone cross-sectional traits have been previously studied before in primates and birds (Demes et al. 1991; Habib and Ruff 2008; Simons et al. 2011), these traits have received considerably less attention than the skeleton’s external dimensions, which have been studied for well over a century (Dublin 1903; Osburn 1903; Shimer 1903; Lull 1904; Gregory 1912; Maynard Smith and Savage 1956; Hildebrand 1985; Van Valkenburgh 1987; Carrano 1999; Elissamburu and Vizcano 2004; Polly 2007; Samuels and Van Valkenburgh 2008; Fabre et al. 2013, 2015; Samuels et al. 2013; Rose et al. 2014; Botton-Divet et al. 2016, 2017; Kilbourne 2017). Here, we test whether the hind limb long bones of fossorial (digging), natatorial (swimming), scansorial (climbing), and generalist taxa are characterized by distinct cross-sectional morphologies in terms of CSA, SMA, and MOD and whether morphological diversity of these traits is possibly due to adaptive evolution. We additionally test whether morphological disparity in cross-sectional traits occurs to differing degrees in the fore- and hind limb among locomotor habits.

Mustelids, a carnivoran lineage including badgers, otters, weasels, martens, and polecats, encompass an impressive ecological diversity (Koepfli et al. 2008; Sato et al. 2012), with various specializations in their locomotor apparatus for functions as varied as climbing, digging, and swimming (Kenyon 1969; Fish 1994; Moore et al. 2013; Rose et al. 2014; Botton-Divet et al. 2016, 2017; Kilbourne 2017, Kilbourne and Hutchinson 2019). The functional diversity present in Mustelidae makes them an excellent monophyletic group to understand how hind limb function relates to hind limb cross-sectional traits, and to test models of trait evolution that may potentially govern the diversification of cross-sectional morphology.

The differing limb functions and locomotor habits displayed by mustelids likely exert different mechanical loads upon the bones of natatorial, fossorial, scansorial, and generalist mustelids. During swimming in natatorial mustelids, hind limb paddling is employed in both surface and, to a lesser extent, subsurface swimming (Procter 1963; Fish 1994). Drag-based swimming with the hind limbs should impose high mechanical loads upon the hind limb skeleton due to the high density of water (1.0 g/cm^3^). However, buoyancy may mitigate increased mechanical loads incurred by hind limb paddling during swimming (e.g., Young and Blob 2015; Young et al. 2017); although, buoyancy itself represents a mechanical load that is often countered by bones of greater cross-sectional robustness in natatorial taxa (Houssaye et al. 2016; Houssaye and Botton-Divet 2018). Although the limbs of fossorial mustelids also function in soil, an even denser substance than water (1.8–2.6 g/cm^3^; Rowell 2014), digging is largely accomplished by the forelimbs. In contrast, the hind limbs act primarily to stabilize the torso against the large forces generated during digging and/or to clear out excavated earth from the site of digging (Hildebrand 1985). The hind limbs play an indispensible role during terrestrial locomotion and climbing, although the limbs of generalist and scansorial mustelids are not functioning in dense substances and must instead counteract gravitational and inertial forces. In particular, a scansorial habit may favor an overall gracile or lightweight body plan, including the limbs, in order to better traverse thin supports during locomotion. Due to the physical differences in their environments, the hind limb bones of mustelids of differing locomotor habits likely experience different predominant loading regimes of bending and compression and their cross-sectional traits may reflect adaptations to particular locomotor habits. Moreover, a recent study on mustelid forelimb cross-sectional traits (Kilbourne and Hutchinson 2019) also allows for a comparison of fore- and hind limb cross-sectional morphology in mustelids and an assessment of whether or not fore- and hind limbs likely underwent a single model of trait evolution with regard to these traits.

Comparing long bone cross-sectional traits among fossorial, natatorial, scansorial, and generalist mustelids, we test whether fossorial and natatorial mustelids have greater values of cross-sectional traits. This hypothesis is based on the limbs of these taxa functioning in denser substances, with natatorial taxa in addition having to counter buoyancy during diving and swimming. We also predict that the cross-sectional traits of scansorial mustelids will be the lowest values among our sampled taxa. We further test whether a model of adaptive diversification best characterizes morphological diversity in the cross-sectional traits of the mustelid hind limb, specifically predicting that the best fitting set of selective regimes will be based upon the four locomotor habits found within extant mustelids.

## Materials and Methods

**Fig. 2:**
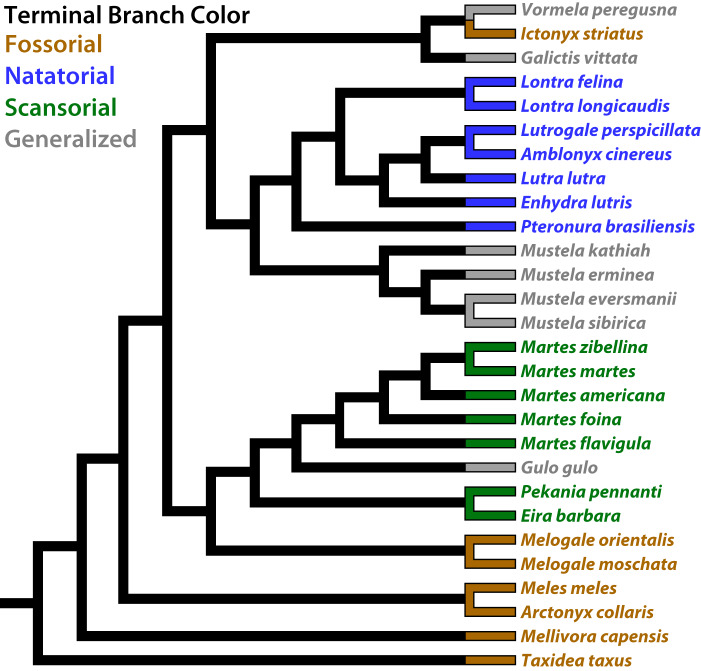
Phylogeny of the 28 sampled mustelid species. The color of terminal branches and taxon corresponds to the locomotor habit for each mustelid species. This phylogeny is a pruned subset of the musteloid phylogeny of Law et al. (2018).

To encompass all main locomotion types, the femur, tibia, and fibula of 40 specimens spanning 28 mustelid species were sampled (Fig. 2), with seven taxa each for fossorial, natatorial, scansorial, and generalist mustelids (Table 1). Specimens were obtained from the Museum für Naturkunde (Berlin, Germany), the Natural History Museum of Denmark (Copenhagen, Denmark), the Národní Museum (Prague, Czech Republic), and the Field Museum of Natural History (Chicago, IL). No consistent side of the specimens was used, since left and right limbs were not always both preserved with the specimen. For detailed information including housing and specimen numbers, see Supplementary data.

**Table 1:** Sampled mustelid species and their locomotor habit, with sample size (*N*) for each species

Species	*N*	Common name	Habit	Reference	Scan location
*Amblonyx cinereus*	2	Asian small-clawed otter	Natatorial	Larivière (2003)	1
*Arctonyx collaris*	1	Hog badger	Fossorial	Nowak (2005)	1
*Eira barbara*	2	Tayra	Scansorial	Presley (2000)	1
*Enhydra lutris*	1	Sea otter	Natatorial	Estes (1980)	1
*Galictis vittata*	1	Greater grison	Generalized	Yensen and Tarifa (2003)	2
*Gulo gulo*	1	Wolverine	Generalized	Pasitschniak-Arts and Larivière (1995)	1
*Ictonyx striatus*	2	Zorilla	Fossorial	Larivière (2002)	1
*Lontra felina*	1	Marine otter	Natatorial	Larivière (1998)	1
*Lontra longicaudis*	1	Long-tailed otter	Natatorial	Larivière (1999)	1
*Lutra lutra*	2	Eurasian otter	Natatorial	Hung and Law (2016)	1
*Lutrogale perspicillata*	1	Smooth-coated otter	Natatorial	Hwang and Larivière (2005)	2
*Martes americana*	2	North American marten	Scansorial	Clark et al. (1987)	2
*Martes flavigula*	2	Yellow-throated marten	Scansorial	Larivière and Jennings (2009)	1
*Martes foina*	2	Beech marten	Scansorial	Larivière and Jennings (2009)	1
*Martes martes*	2	Pine marten	Scansorial	Larivière and Jennings (2009)	1
*Martes zibellina*	2	Sable	Scansorial	Larivière and Jennings (2009)	1
*Meles meles*	2	European badger	Fossorial	Larivière and Jennings (2009)	1
*Mellivora capensis*	1	Honey badger	Fossorial	Vanderhaar and Ten Hwang (2003)	1
*Melogale moschata*	1	Chinese ferret-badger	Fossorial	Storz and Wozencraft (1999)	1
*Melogale orientalis*	1	Javan ferret-badger	Fossorial	Nowak (2005)	1
*Mustela erminea*	2	Ermine	Generalized	King (1983)	1
*Mustela eversmanii*	1	Steppe polecat	Generalized	Larivière and Jennings (2009)	1
*Mustela kathiah*	1	Yellow-bellied weasel	Generalized	Larivière and Jennings (2009)	1
*Mustela sibirica*	1	Siberian weasel	Generalized	Law (2018)	1
*Pekania pennanti*	1	Fisher	Scansorial	Powell (1981)	1
*Pteronura brasiliensis*	1	Giant otter	Natatorial	Noonan et al. (2017)	1
*Taxidea taxus*	2	North American badger	Fossorial	Long (1973)	1
*Vormela peregusna*	1	Marbled polecat	Generalized	Gorsuch and Larivière (2005)	1

CT scans were made at one of two facilities: (1) Museum für Naturkunde Berlin, Berlin, Germany or (2) University of Chicago, Chicago, IL, USA.

To obtain cross-sectional traits, bones were scanned with micro computed tomography (µCT) at the Museum für Naturkunde Berlin and the University of Chicago using, respectively, a Phoenix|x-ray Nanotom scanner and a combination Nanotom-v|tome|x scanner (GE Sensing and Inspection Technologies GmbH, Wunstorf, Germany), with volumes being produced with the software datos|x-reconstruction (version 1.5.0.22). Oriented image stacks in DICOM format were produced with VG Studio Max 2.0 and 2.1 (Volume Graphics, Heidelberg, Germany), with a minimum number of 1000 slices and bones oriented so their proximo-distal axis was vertical. To reduce these to an analytically manageable number of images, the slice numbers corresponding to the proximal and distal tips of a bone were first identified. The difference between the numbers of the image slices capturing the proximal and distal tips of the bone was divided by 20, which resulted in 19 image slices per bone. These 19 slices corresponded to 5% increments of bone length, representing 5–95% of bone length. ImageJ (Schneider et al. 2012) was used to segment the bone out against a black background so as to reduce background noise, and the ImageJ plug-in BoneJ (Doube et al. 2010) was used to measure cross-sectional traits from each segmented slice. Next, we converted the image stack from 16 to 8 bit and set the cranio-caudal axis orientation to 270°. Having segmented the bones out against a black background, we set the maximum grayscale value associated with bone tissue to 255, and a minimum grayscale value associated with bone tissue was manually chosen for each slice. These maximum and minimum values were input into BoneJ to calculate CSA, SMA, and MOD for each image slice. SMA and MOD were measured about the cranio-caudal (SMA_CC_/MOD_CC_) and medio-lateral axes (SMA_ML_/MOD_ML_).

To mitigate the influence of body size upon these traits, we first took, respectively, the square, fourth, and third root of CSA, SMA, and MOD (e.g., CSA^1/2^, SMA^1/4^, MOD^1/3^), as these traits have corresponding units of mm^2^, mm^4^, and mm^3^. These transformations reduced the traits to a singular linear dimension (mm); dividing these transformed trait values by a bone’s length consequently rendered these traits dimensionless. Raw values of cross-sectional traits and bone lengths are available in Supplementary data.

### Ratios of cross-sectional traits

As in Kilbourne and Hutchinson (2019), we also calculated two parameters R_ML_ and R_CC_, respectively, corresponding to the ratios of the dimensionless values of SMA_ML_/CSA and SMA_CC_/CSA. Comparing these parameters among mustelid locomotor habits is informative as to whether the resistance to bending relative to the resistance to axial compression differs among mustelid locomotor habits.

To determine among mustelid locomotor habits any differences in the cross-sectional traits of forelimbs relative to hind limbs, we also calculated ratios for CSA and SMA for pairs of serial homologues between the fore- and hind limbs (humerus vs. femur, radius vs. tibia, ulna vs. fibula). These ratios should reveal whether mechanical load resistance of forelimb relative to hind limb bones varies among mustelid locomotor habit.

### Analyses

Using R version 3.5.1 (R Development Core Team 2018), one-way analysis of variance (ANOVA) along with Tukey’s pairwise post hoc comparison was used to assess biomechanically relevant differences in femoral, tibial, and fibular cross-sectional traits, as well as ratios of cross-sectional traits, with locomotor habit serving as the independent factor (*P*_significant_ < 0.05). Given that we tested for trait differences among locomotor habits for 19 increments along a bone’s length, we applied a Bonferroni adjustment, such that *P*_Bonferroni_ = 0.0026 (i.e., 0.05/19). Additionally, to determine if phylogeny has influenced how cross-sectional traits differ among locomotor habits, we performed a phylogenetic ANOVA using the method of Adams and Collyer (2018), as well as performing a two-block partial least squares analysis between locomotor habit and phylogenetic covariance to determine if locomotor habit clusters within the phylogeny (Adams and Collyer 2018). The latter partial least squares analysis between locomotor habit and phylogenetic covariance tests whether locomotor habit is concentrated within sublineages of the phylogeny. This is of concern as a concentration of grouping variables within the phylogeny can influence biological inferences allowed by the data (see Adams and Collyer 2018 for more detailed information).

The standard ANOVA and phylogenetic ANOVAs are intended to work in concert. The non-phylogenetic analyses ideally should reveal whether biomechanical demands of ecological niches are reflected in morphology. More specifically, these analyses are focused on what differences in morphological structures directly entail for the capability of organisms to cope with the physical forces encountered during actual function in their natural environment. For instance, from continuum mechanics, the relationship between normal stress (σ) and CSA is σ = *F*/CSA, where *F* is a force applied perpendicularly to the bone’s cross-section. The normal stress directly experienced by an organism’s bone is solely due to the incurred force and the value of CSA possessed by that individual, regardless of the trait values of its ancestors or the specific evolutionary process underlying the diversification of its lineage. However, in spite of their dictating an individual’s biomechanical capability or performance, the trait values of the individual are related to those of its relative due to common descent. To address this, the phylogenetic analyses should specifically reveal whether phylogenetic relatedness has tied into how ecological niche is distributed among mustelids and whether phylogenetic relatedness has influenced any biomechanically meaningful morphological differences associated with those niches.

Two-block partial least squares, phylogenetic ANOVAs, and pairwise comparisons for phylogenetic ANOVAs were performed with R package geomorph (Adams et al. 2018). Pairwise comparisons were performed with the function “morphol.disparity.”

### Trait diversification

The phylogenetic relatedness of mustelids provides the opportunity to estimate the evolutionary processes responsible for the morphological diversification of cross-sectional traits in this clade. To this end, we fitted two kinds of trait diversification models: Brownian motion (BM) and Ornstein–Uhlenbeck (OU). The BM model is a model of stochastic evolution, whereas the OU model is a model of adaptive evolution (Hansen 1997; Butler and King 2004). According to the BM model, an increase in trait value is just as likely as a decrease in trait value at any given point of a trait’s evolution. In contrast, the OU model specifies evolution toward a phenotypic “optimum” corresponding to a peak in an adaptive landscape, with evolution toward this optimum/peak being determined by α, a critical parameter denoting the rate of adaptation or the strength of selection (Hansen 1997; Cooper et al. 2016). Notably, there is a variant of BM models that includes multiple rates of evolution (e.g., O’Meara et al. 2006) and phenotypic means for groups (e.g., locomotor habits) under study (Thomas et al. 2006); likewise, there are variants of the OU model that allow for multiple phenotypic optima/adaptive peaks (e.g., Butler and King 2004; Beaulieu et al. 2012).

**Fig. 3:**
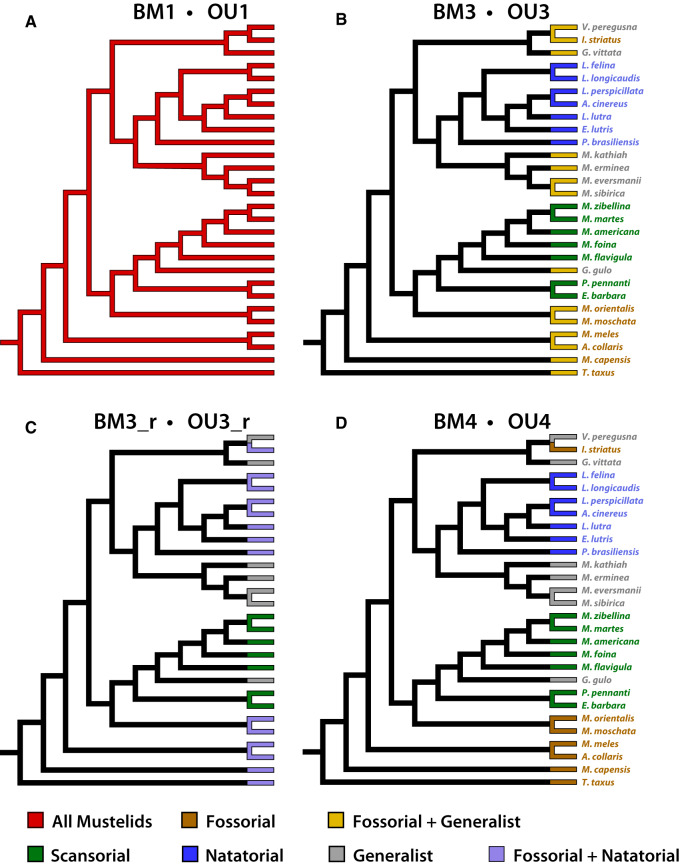
Four hypothetical selective regimes for cross-sectional morphology in the femur, tibia, and fibula. The phylogeny in (**A**) represents a single rate (BM1) or single phenotypic optimum (OU1) acting across all branches of the phylogeny, both internal and external. The phylogeny in (**B**) distinguishes three distinct rates (BM3) or optima (OU3) acting across the terminal branches of the phylogeny, with natatorial, scansorial, and remaining mustelids (i.e., fossorial and generalist taxa) each having their own rate/optimum. The phylogeny in (**C**) distinguishes three distinct rates (BM3_r) or optima (OU3_r) acting across the terminal branches of the phylogeny, with fossorial and natatorial mustelids being together distinguished by their own rate/optimum, as well as scansorial and generalist mustelids being each distinguished by their own individual rate/optimum. The phylogeny in (**D**) distinguishes four distinct rates (BM4) or optima (OU4) acting at the terminal branches of the phylogeny, one for each of the four locomotor habits within Mustelidae. For the BM3/OU3, BM3_r/OU3_r, and BM4/OU4 models, rates and optima acting along internal branches were estimated using stochastic character mapping (Huelsenbeck et al., 2003; Bollback, 2006), which reflects uncertainty in character states of internal branches (see main text).

Following Kilbourne and Hutchinson (2019), we fit three variants for both the BM and OU model (Fig. 3). For the BM model, we fit a model with a single rate of evolution (BM1) and three models with multiple rates of evolution. Two of these multi-rate models had three rates. The first three-rate model (BM3) had a distinct rate for natatorial and scansorial mustelids each and a distinct rate for remaining mustelids (i.e., fossorial and generalist taxa). The distinct rate for scansorial mustelids was based upon the overall gracile morphology of their limb skeleton (Holmes 1980; Kilbourne 2017), whereas the distinct rate for natatorial mustelids was based upon robust bone cross-sectional morphologies typically associated with aquatic taxa (Houssaye 2009; Houssaye and Botton-Divet 2018). The second three-rate model (BM3_r) had a distinct rate for scansorial mustelids, generalist mustelids, and, as a single grouping, fossorial and natatorial mustelids. The grouping of fossorial and natatorial taxa was based upon the *a priori* expectation that these two locomotor habits have a robust cross-sectional morphology. A four-rate model (BM4) estimated distinct rates of BM evolution for each of the four locomotor habits within Mustelidae. Also note that the multi-rate BM models—BM3, BM3_r, and BM4—are group mean models, where a mean trait value is associated with each of the groups modeled as having a distinct rate of evolution.

Each of the BM models had a corresponding OU model; thus, we fit a single optimum OU model (OU1), two OU models with three optima, and a four optima model (OU4), for which each locomotor habit has its own optimum. The first three optima OU model specified one optimum for scansorial mustelids, a second for natatorial mustelids, and a third for remaining mustelids (OU3), whereas the second three optima OU model (OU3_r) specified a distinct optimum for fossorial mustelids together with natatorial mustelids (i.e., the locomotor habits predicted to robust cross-sectional morphologies) and distinct optima each for scansorial and generalist mustelids. We fit the BM and OU models using the R package OUwie (Beaulieu et al. 2012). We also fit an early burst (EB) model of trait evolution (Harmon et al. 2010) in addition to the BM and OU models. The EB model typically represents a scenario with a decreasing rate of phenotypic evolution. However, depending on how the rate parameter is bounded, it can also be used to represent a scenario with an increasing rate of evolution. The EB model was fit using the R package geiger (Harmon et al., 2008).

For multi-rate BM and multi-optima OU models, a critical issue is incorporating uncertainty in the ecological niches/locomotor habits occurring along a phylogeny’s internal branches. To address this uncertainty, we used stochastic character mapping (Huelsenbeck et al. 2003; Bollback 2006). Following this method, the differing locomotor habits exhibited by the phylogeny’s terminal taxa are stochastically mapped onto the internal branches. To this specific mapping of locomotor habits, the multi-rate BM and multi-optima OU models were fit, with the parameters for each model being estimated. This process of stochastic mapping and model fitting is then repeated for a total of 500 iterations. For a given model, the 500 iterations generated a pool of 500 estimates for each of the model’s parameters; a mean value was then calculated for each parameter from its corresponding pool of estimates. Stochastic character mapping was performed using the R package phytools (Revell 2012). To additionally estimate uncertainty in parameter estimates, we also performed parametric bootstrapping (with 1000 replicates) for each parameter to generate 95% confidence intervals. For each of our models, parameter estimates and confidence intervals are in Tables A1–A6 in Supplementary Appendix.

To fit our models of trait evolution, as well as our phylogenetic ANOVAs, we used the phylogeny of Law et al. (2018) pruned to our sample of 28 mustelids (Fig. 2). As in Kilbourne and Hutchinson (2019), the BM and OU models were also implemented using parallel processing using the R package parallel.

## Results

**Fig. 4:**
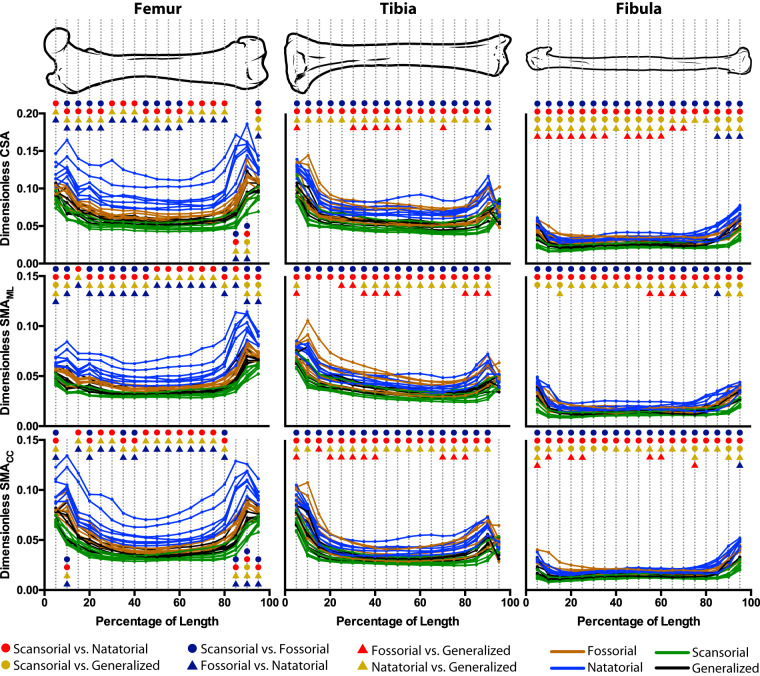
Differences in femoral, tibial, and fibular CSA and SMA among mustelid locomotor habits. CSA, SMA_ML_, and SMA_CC_ were quantified in 5% increments along the length of our sampled bones, and an ANOVA was performed for each increment. To render the traits dimensionless, the square and fourth roots were applied, respectively, to CSA and SMA, with the resulting values then being divided by bone length. For each increment, colored symbols indicate a significant difference (adjusted *P *<* *0.05) for a given pairwise comparison among locomotor habits. A lack of significant pairwise differences indicates an overall ANOVA result of *P *>* *0.0026 (the Bonferroni-corrected *P*-value) for that increment.

For the femur, tibia, and fibula, we found significant differences (*P*_Bonferroni_ ≤ 0.0026) in cross-sectional traits among mustelid locomotor habits largely across the lengths of all three bones, indicating that there are biomechanically relevant morphological differences among mustelid locomotor habits (Fig. 4).

The femur has the greatest range of values for each cross-sectional trait of the three hind limb bones studied (Fig. 4 and Supplementary Fig. S1). Natatorial mustelids have the highest values of femoral cross-sectional traits, whereas scansorial mustelids have the lowest values. There is little to no overlap between natatorial mustelids and non-natatorial mustelids across the bone’s entire length, suggesting that the femora of natatorial mustelids are distinguished from other mustelids by exceptionally high values of dimensionless cross-sectional traits. In particular, *Enhydra lutris* and *Pteronura brasiliensis* appear to have distinctly higher values of femoral cross-sectional traits compared to other natatorial mustelids. Pairwise post hoc comparisons find that natatorial mustelids indeed have significantly greater values of femoral cross-sectional traits compared to other mustelid locomotor habits (*P*_Adjusted_ < 0.05). To a much lesser degree, fossorial mustelids also significantly differ from scansorial mustelids, with this pairwise difference not running the entire length of the femur for any of the five traits (Fig. 4 and Supplementary Fig. S1).

For the tibia, the lowest values of cross-sectional traits are exhibited by scansorial mustelids (Fig. 4 and Supplementary Fig. S1). The highest are exhibited by fossorial and natatorial mustelids, which strongly overlap in trait values. Notably, our sampled fossorial mustelids seem to separate into two groups with regard to tibial cross-sectional traits. *Arctonyx collaris*, *Meles meles*, *Mellivora capensis*, and *Taxidea taxus* have higher trait values, overlapping with natatorial mustelids, if not exceeding their trait values. *Melogale moschata*, *M.orientalis*, and *Ictonyx striatus* have lower trait values, overlapping more with scansorial and generalized mustelids. Fossorial mustelids significantly differ from scansorial mustelids in all five sampled traits for all increments of tibial length except for 95%; however, these significant differences are likely due to the aforementioned high trait values of *A. collaris*, *M. meles*, *M. capensis*, and *T. taxus*. Fossorial mustelids also tend to significantly differ from generalist mustelids, though these differences are not pervasive across the entire length of the tibia apart from MOD_ML_ (Supplementary Fig. S1). Natatorial mustelids’ high values of cross-sectional traits significantly distinguish them from scansorial and generalist mustelids. The significant difference between natatorial and scansorial mustelids is consistent across the length of the tibia. However, significant differences between natatorial and generalist mustelids are consistent across the length of the tibia with regard to CSA and SMA_CC_; with regard to SMA_ML_ and MOD, significant differences between these groups are not as extensive across the tibia’s length.

The range of values covered by fibular cross-sectional traits is much narrower in comparison to the femur and tibia (Fig. 4 and Supplementary Fig. S1). Thus, any morphological differences among locomotor habits beneficial to biomechanics are not as pronounced in the fibula as in the remaining two bones. Additionally, the narrower range of fibular trait values is in part the result of each locomotor habit having a narrower range of trait values. The highest values of fibular cross-sectional traits are possessed by natatorial and fossorial mustelids and the lowest values by scansorial mustelids, as is the case for the femur and tibia. Largely across the fibula’s length, scansorial mustelids possess significantly lower trait values of cross-sectional traits than both natatorial and fossorial mustelids. Likewise, generalist mustelids tend to possess significantly lower fibular cross-sectional trait values than natatorial and fossorial mustelids, though the difference between fossorial and generalist mustelids is not as extreme as it is between natatorial and generalist mustelids.

### Ratios of resistances to bending and compression

**Fig. 5:**
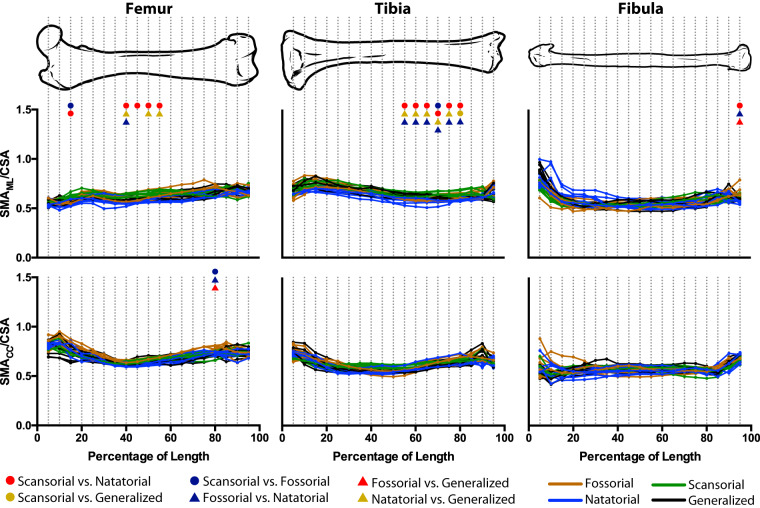
Differences in resistance ratio R for the femur, tibia, and fibula among mustelid locomotor habits. Dimensionless CSA and SMA values were used to calculate R for each of the 5% increments of bone length. For pairwise comparisons of locomotor habits, colored symbols indicate a significant difference (adjusted *P *<* *0.05) for a given comparison, with a lack of significant pairwise differences indicating a non-significant result for the overall ANOVA (*P *>* *0.0026; the Bonferroni-corrected *P*-value).

*R*-values for all three bones tend to be below 1.0, corresponding to a relatively greater resistance to axial compression than bending. Bending resistance relative to compression resistance is largely uniform across mustelid locomotor habits (Fig. 5), as there is considerable overlap among locomotor habits—both R_ML_ and R_CC_—along the entire length of our three sampled hind limb bones. Moreover, along the lengths of the three bones, ANOVAs fail to recover any trends in morphological differences among locomotor habits. The only exceptions to this are between the 40% and 55% increments of femoral R_ML_ and the 55% and 80% increments of tibia R_ML_. The former indicates that in the region of the femoral midshaft natatorial mustelids tends to have lower R_ML_ than scansorial and generalist mustelids, whereas the latter indicates that in the distal half of the tibia, natatorial mustelids have lower values of R_ML_ than other locomotor habits. However, the considerable overlap among locomotor habits in Fig. 5 suggests that in spite of statistical significance, the morphological differences represented by variation in R_ML_ are not dramatic.

### Ratios of fore- and hind limb cross-sectional traits

**Fig. 6:**
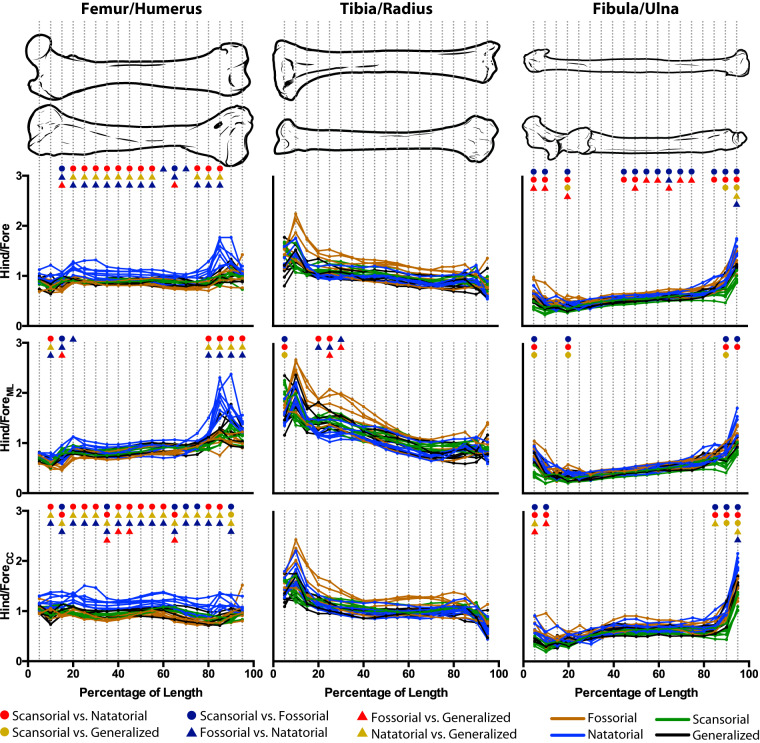
Ratios of hind- to forelimb cross-sectional traits. Hind limb–forelimb ratios were calculated for a given trait (e.g., CSA) and analyzed for significant differences among locomotor habits for each increment of bone length (as a percentage of total bone length). The ratios of hind- and forelimb traits are the femur/humerus, tibia/radius, and fibula/ulna. A colored symbol for a given pairwise comparison indicates a significant difference (adjusted *P *<* *0.05) for that given comparison. For a given increment of bone length, a lack of significant pairwise differences indicating a non-significant result for the overall ANOVA (*P *>* *0.0026; the Bonferroni-corrected *P*-value).

In a comparison of serial homologues of the fore- and hind limb, the femora of natatorial mustelids have a higher ratio of femoral to humeral CSA and SMA_cc_ than other mustelid locomotor habits (Fig. 6). This indicates, that in comparison to other mustelids, aquatic mustelids possess femora tending to have a greater resistance to axial compression and bending about the cranio-caudal axis (i.e., within the transverse plane) than the humerus. This finding also applies to SMA_ML_ with regard to the distal ends of the humerus and femur. It should be noted though that ratios of femoral to humeral cross-sectional properties tend to cluster close to a value of 1.0, suggesting that in mustelids the humerus and femur have similar capacities to withstand bending and compression. There are a few increments for CSA and SMA_CC_ ratios where fossorial mustelids have significantly lower ratios than other mustelid locomotor groups, but for the most part fossorial mustelids overlap in their values with scansorial and generalist mustelids.

Comparing tibial to radial cross-sectional properties finds ratios for CSA and SMA_ML_ that tend to decrease distally along the tibia’s length, whereas the ratio for SMA_CC_ is, by comparison, more constant along the tibia’s length (Fig. 6). For all three traits, there is considerable overlap among mustelid locomotor habits, though in the proximal half of the tibia there are three fossorial taxa whose trait values stand out from other mustelids: *A.collaris*, *M.meles*, and *T.taxus*. For CSA and SMA_CC_, mustelid locomotor habits do not significantly differ in their ratio values. For SMA_ML_, fossorial mustelids tend to differ from other locomotor habits at 25–30% of bone length, likely due to the high values of the three aforementioned taxa. The higher ratio values of fossorial mustelid suggest these specialists, more so than in other mustelids, tend to have tibiae that can withstand greater mechanical loads as compared to their radii.

With regard to ratios of fibular to ulnar cross-sectional traits, ratios of trait values tend to increase distally along bone length (Fig. 6). In comparison to femoral–humeral and tibial–radial ratios, fibular–ulnar ratios tend to cluster more together. Intermittently along bone length, scansorial mustelids have significantly lower values of fibular–ulnar CSA ratios than fossorial and scansorial mustelids, and, likewise intermittently, generalist mustelids have significantly lower values of CSA ratios than fossorial mustelids. With regard to fibular–ulnar SMA_ML_ ratios, there is a lack of significant differences among locomotor habits, with the exception of scansorial mustelids having significantly lower SMA_ML_ ratios than other mustelids at the bones’ proximal and distal extremes. With regard to fibular–ulnar SMA_CC_ ratios, scansorial and generalist mustelids tend to have lower values of ratios corresponding to the bones’ proximal ends than fossorial and natatorial mustelids. In contrast, ratio values corresponding to the bones’ distal ends significantly distinguish scansorial mustelids from other mustelids and, to a lesser extent, natatorial from generalist mustelids.

### Phylogenetic ANOVAs

Locomotor habit significantly clusters among the terminal branches of the mustelid phylogeny (*r *=* *0.912, *P *=* *0.0001). For the 85% increment of femoral length, phylogenetic ANOVAs find that femoral CSA (*P *=* *0.018), SMA_ML_ (*P *=* *0.019), and MOD_ML_ (*P *=* *0.016) differ among mustelid locomotor habits. Among pairwise comparisons for these traits, the only comparison approaching significance was the natatorial–fossorial comparison (CSA: 0.0580; SMA_ML_: 0.0510; MOD_ML_: 0.0530). Otherwise, no significant differences are present among locomotor habits in CSA, SMA_ML_, and MOD_ML_ for the remaining increments of femoral length (*P*_CSA_ = 0.060–0.379; *P*_SMAML_ = 0.218–0.664; *P*_MODML_ = 0.168–0.777). For femoral SMA_CC_ and MOD_CC_, there are no significant differences among any mustelid locomotor habits along the femur’s length (*P*_SMACC_ = 0.051–0.544; *P*_MODCC_ = 0.058–0.718). Phylogenetic ANOVAs uncover that tibial CSA, SMA_ML_, and MOD_ML_ do not significantly differ among locomotor habits along the entire length of the tibia. However, phylogenetic ANOVAs are significant for SMA_CC_ and MOD_CC_ from the increments in the region of 30–50% tibial length (*P *=* *0.017–0.045). However, pairwise comparisons for these traits uncover no significant differences. For the fibula, the results are more variable. Phylogenetic ANOVAs are significant for the 15, 20, 65, and 90% increments of fibular length for CSA (*P *=* *0.026–0.049), SMA_ML_ (*P *=* *0.010–0.039), and MOD_ML_ (*P *=* *0.011–0.039). Furthermore, 60% and 70% increments were also significant for SMA_ML_ (*P *=* *0.026–0.029) and MOD_ML_ (*P *=* *0.037–0.042). Phylogenetic ANOVAs produced significant results only for the 90% increment for both fibular SMA_CC_ (*P *=* *0.016) and MOD_CC_ (*P *=* *0.047). Pairwise comparisons uncover no significant differences for any of the fibular traits for which significant phylogenetic ANOVAs were recovered.

### Trait diversification

**Fig. 7:**
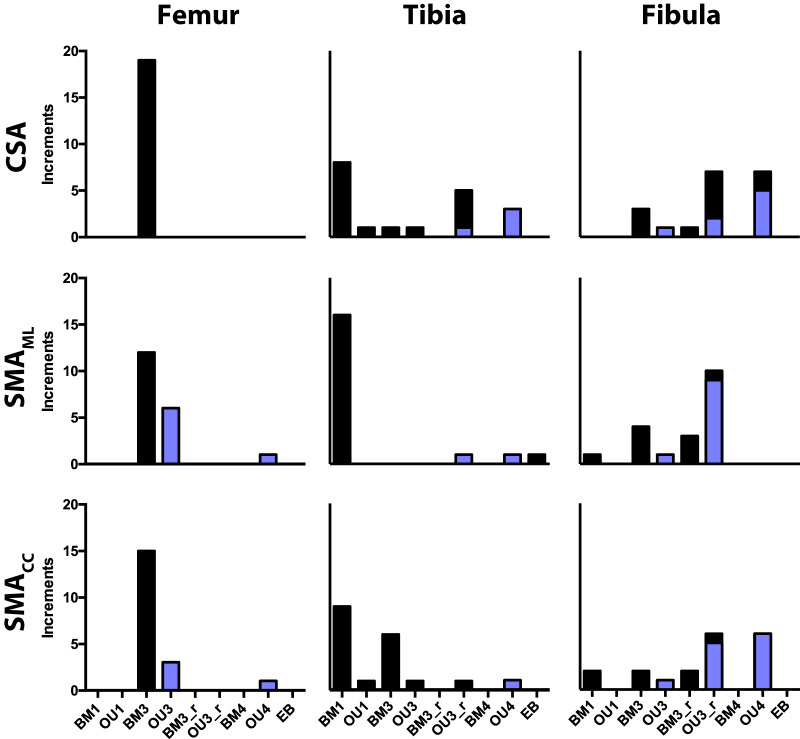
Frequency of best fit for each model of trait evolution for femoral, tibial, and fibular CSA and SMA. For each model, the total number of increments best fit by the model are presented. Superimposed blue bars indicate the number of OU models that also had a significant value of α, which represents the strength of selection.

Regarding trait diversification models, femoral CSA and SMA are the best fit by the three-rate BM3 or the three-optima OU3 model (Fig. 7 and Supplementary Table S1). Between these two models, BM3 is more frequently the best fitting model for all three traits. With regard to tibial traits (Fig. 7 and Supplementary Table S2), the best fitting model of trait evolution is predominantly a single rate BM model (BM1), with this model being the best fit for as few as eight out of nineteen increments (CSA) up to sixteen out of nineteen increments (SMA_ML_). It should be noted though that the Akaike weights for tibial traits’ tend not to exceed 60% for the best fitting model, often being much lower. Of all the models, the models OU3_r and OU4 are the predominant best fitting models for the greatest number of increments for fibular CSA, SMA_ML_, and SMA_CC_ (Fig. 7 and Supplementary Table S3).

In instances where an OU model was the best fitting model, inspecting values of α finds that most of these OU models actually have values of α that are associated with wide confidence limits (Supplementary Tables S4–S6), indicating difficulty in estimating this parameter. Nevertheless, in most instances, the α-values are significant, the confidence limits excluding a value of 0.0 (Supplementary Tables S4–S6; Fig. 7). For all best fitting OU models for femoral SMA_ML_ and SMA_CC_, which was primarily OU3, α-values are significant (Supplementary Table S4). For tibial cross-sectional traits (particularly CSA) when OU3_r is the best fitting model, the α-value tend not to be significant (Supplementary Table S5). With regard to fibular cross-sectional traits, α-values tend to be significant for the best fitting OU models (Supplementary Table S6). An exception to this is all but one instance when OU3_r is the best fitting model for fibular CSA. α-values for these models cannot be distinguished from 0.0.

## Discussion

We found that cross-sectional morphology of hind limb bones in mustelids likely confers biomechanical benefits tied to locomotor habits, as was the case for forelimb bones (Kilbourne and Hutchinson 2019). Our results further underscore that form–function relationships apply not only to the external structure of the skeleton but also to the internal structure, with the former reflecting the ability of the skeleton to act as primarily a system of levers and the latter reflecting the ability of the skeleton to resist mechanical loads. Studies on the external dimensions of the limb skeleton are numerous, encompassing virtually all major tetrapod groups (e.g., Zeffer et al. 2003; Joyce and Gauthier 2004; Schmidt and Fischer 2009); here we have presented novel findings on the internal structure of the limb skeleton in mustelids to expand our insights into whether limb bone function relates to cross-sectional morphology in an ecologically diverse tetrapod lineage. Furthermore, we used a phylogenetic comparative framework to assess whether biomechanical function is related to morphological diversification in cross-sectional traits.

Our results are in line with studies investigating cross-sectional traits and locomotor specializations in other tetrapod groups, including birds specialized for differing flight modes (Habib and Ruff 2008; Simons et al. 2011) and primates specialized in different locomotor modes (Demes et al. 1991; Ruff 2002). Houssaye et al. (2018) also found that cross-sectional traits distinguish within phenotypic space taxa with differing locomotor specializations, though they used a multivariate approach. The distribution of cross-sectional traits along the lengths of the femur, tibia, and fibula (i.e., the shapes of the curves in Fig. 4 and Supplementary Fig. S1) also are largely in line with the findings of Doube et al. (2009, 2012, 2018), with the epiphyses tending to have somewhat higher values of dimensionless cross-sectional traits than the diaphysis. However, our results stand slightly in contrast to a recent study on sciuromorph femora (Scheidt et al. 2019)—aerial (i.e., gliding) taxa were found to have lower values of epiphyseal cross-sectional traits than fossorial and scansorial taxa, and fossorial and scansorial taxa were rarely found to differ from one another. Our findings also agree with Houssaye and Botton-Divet (2018) in their comparison of bone cross-sectional traits and microanatomy between generalized and natatorial mustelids, though this study did not examine cross-sectional traits in a specifically biomechanical context (i.e., CSA being a measure of resistance to axial compression).

### Biomechanical implications of cross-sectional morphology

In agreement with our prediction, natatorial mustelids have femora, tibiae, and fibulae with greater cross-sectional traits than scansorial and generalized mustelids (Fig. 4 and Supplementary Fig. S1). In comparison to fossorial mustelids, natatorial mustelids have markedly higher values of femoral cross-sectional traits with almost no overlap between these two groups; however, there is extensive overlap in trait values between these two groups for tibial and fibular traits. Though the greater values of cross-sectional traits should contribute to a greater resistance to axial compression and bending, the higher trait values for natatorial mustelids are likely more related to the need of otters to counteract buoyancy in aquatic environments and less so to the need to counteract increased compression or bending loads. In studies on terrestrial versus aquatic locomotion in turtles, Young and Blob (2015) and Young et al. (2017) found that swimming produces lower values of shear strain than terrestrial locomotion, with the lower values associated with swimming likely due to the fact that supporting body weight against gravity is not a relevant mechanical load in aquatic environments. These results parallel those for the humerus, radius, and ulna found by Kilbourne and Hutchinson (2019).

In comparing natatorial and non-natatorial mustelids, there is a noticeably higher disparity between these two groups for femoral cross-sectional traits than for the tibia and fibula (Fig. 4 and Supplementary Fig. S1). In particular, unlike the tibia and fibula, there is almost no overlap in femoral cross-sectional traits among natatorial and fossorial mustelids. This disparity may be due to our rendering a bone’s cross-sectional traits dimensionless by dividing by the bone’s length, as natatorial carnivorans tend to have relatively short femora so as to reduce induced drag during the recovery stroke of the paddling hind limb (Samuels et al. 2013; see also Samuels and Van Valkenburgh 2008). The relatively short femur of natatorial mustelids may especially underlie morphological disparity between fossorial and natatorial mustelids that is present in the femur yet absent in our other two sampled long bones. However, it is worth noting that natatorial mustelids still have higher values of cross-sectional traits compared to scansorial and generalized mustelids for both the tibia and fibula (Fig. 4 and Supplementary Fig. S1), indicating that the results for the femur are not solely due to its relatively shorter length.

Also in line with our prediction, fossorial mustelids tend to have higher values of femoral, tibial, and fibular cross-sectional traits than scansorial and generalized mustelids (Fig. 4 and Supplementary Fig. S1). Though badgers primarily dig with the forelimb (Hildebrand 1985; Moore et al. 2013), our results suggest that biomechanically advantageous traits for digging occur in both the fore- and hind limbs. Moore et al. (2013) report that the hind limbs of the North American badger (*T.taxus*) are used to remove dirt and debris from the area of digging and that the hind limbs essentially brace the animal, moving the center of mass forward during part of the power stroke as the animal digs. Given the rather impressive feats of digging exhibited by badger species, such as caching food items several times their size (Frehner et al. 2017), nightly digging a new burrow (Long 1973), or digging extensive tunnel systems (Roper 1992), the act of bracing the body during these activities may subject the hind limb bones to high mechanical loads. This suggestion, however, needs to be further corroborated by *in vivo* data collected from digging badgers.

Scansorial mustelids tend to have lower values of cross-sectional traits than other mustelids, fitting our prediction. Lower values of cross-sectional traits may be tied to lower bone mass, and thus a lower overall body mass, which is likely advantageous to navigating thin supports or ascending vertically (Cartmill 1985). The results parallel previous findings for the external dimensions of the limb skeleton (Fabre et al. 2013; Kilbourne 2017; Botton-Divet et al. 2018), in which the fore- and hind limb bones of scansorial mustelids are more gracile than those of mustelids of other locomotor habits. However, it should be noted that the relatively elongate limb bones of scansorial mustelids may influence our results, as we rendered cross-sectional traits dimensionless by dividing by bone length (see above).

However, there is a possibility that the morphological differences in hind limb cross-sectional morphology—and the conferred biomechanical benefits—among mustelid locomotor habits are due to allometric effects. The relationship between size and locomotor habit appears to be complex (Supplementary Fig. S2). Notably, natatorial mustelids appear to exhibit a steeper allometric trend than non-natatorial mustelids; furthermore, natatorial mustelids also exhibit body masses larger than all but the largest fossorial and scansorial mustelids and the wolverine (*Gulo gulo*). However, at the same time, where the four locomotor habits overlap in the scaling plots reveals that the locomotor habits are staggered in the values of the cross-sectional traits, with natatorial mustelids having the highest values, followed by fossorial and generalist mustelids, and scansorial mustelids having the lowest values. This staggering of the scaling trends of mustelid locomotor habits largely parallels the distribution of locomotor habits when looking at the cross-sectional traits in a univariate manner (Fig. 4 and Supplementary Fig. S1). Thus, our results regarding morphological differences in cross-sectional morphology are likely owed to a mix of locomotor habits and scale effects, and we intend to further investigate scaling trends in mustelid cross-sectional traits in future studies.

It is also possible that since some of our locomotor habits coincide with monophyly within our phylogeny that our results pertain particularly to these monophyletic lineages and not to locomotor habits in and of themselves. That is to say, our findings for natatorial and scansorial mustelids may not apply more broadly to natatorial and scansorial mammals but only apply exclusively to the mustelid lineages Lutrinae (e.g., otters) and Guloninae (e.g., martens). While this is a possibility, we think it unlikely, as robust limb bones (including cross-sectional properties) are known to characterize several lineages of natatorial mammals, as well as other tetrapods (Fawcett 1942; Déméré 1994; Taylor 1994; Samuels and Van Valkenburgh 2008: Houssaye 2009; Amson et al. 2014, 2015; Houssaye and Botton-Divet 2018). Likewise, limb skeletons of scansorial mammals tend to be characterized by more gracile proportions (Cartmill 1985; Polly 2007; Samuels and Van Valkenburgh 2008). Nonetheless, our biomechanical interpretation of our results would still apply to our sampled taxa at the very least.

### Axial compression versus bending: ratios of load resistance

Our ratios of R_ML_ and R_CC_ tended not to differ among mustelid locomotor habit (Fig. 5), this result largely mirroring the previous result for the forelimb skeleton (Kilbourne and Hutchinson 2019). These results indicate that our sampled hind limb bones are uniform across locomotor habits in their distribution of bone tissue relative to the total amount of bone tissue. Thus, the differing locomotor modes in mustelids do not appear to be associated with relative differences in the resistance to bending versus compression. As values of R are by and large well below 1.0, this suggests that the femur, tibia, and fibula are more geared to withstand axial compression than bending. The one exception to this uniformity among hind limb bones is the distal half of the tibia—compared to other mustelids, natatorial mustelids have lower values of R_ML_ (i.e., a relatively lower resistance to bending in the parasagittal plane). This strongly implies a difference in cross-sectional shape in the tibiae of natatorial mustelids, with these taxa having a relatively narrower distribution of bone about the tibia’s mediolateral axis compared to non-natatorial mustelids. A narrower distribution of bone about the mediolateral axis relative to the total amount of bone tissue could reflect greater bone compactness in the tibia, which would be advantageous to counteract buoyancy (see Houssaye and Botton-Divet 2018). The similarity and overlap in R among natatorial and non-natatorial mustelids, in spite of the greater values of CSA and SMA, exhibited by natatorial mustelids may also point toward the increased bone compactness of otters reported by Houssaye and Botton-Divet (2018). Similar to the tibia, the humerus exhibits significant differences in R_CC_ among mustelid locomotor habits, distinguishing fossorial and natatorial mustelids (Kilbourne and Hutchinson 2019). Thus, in mustelids, there appears to be both uniformity regarding the limb skeleton’s relative resistance to differing loading regimes (e.g., femur, fibula, radius, and ulna) and morphological differences linked to biomechanical demands (e.g., tibia and humerus).

### Fore- versus hind limb cross-sectional traits

Comparing serial homologues of the fore- and hind limb, we found no difference in the cross-sectional traits of the hind limb relative to those of the forelimb that is consistent across all three pairs of homologues (Fig. 6). With regard to the stylopodial bones, the greater ratio values of natatorial mustelids for CSA and SMA_CC_ are likely a product of the markedly greater CSA and SMA of the femur (Fig. 4), as well as this bone’s relatively short length (see above).

With regard to the tibia versus the radius, the general shape of the curves for the ratio values tends to decrease as proceeding distally along bone length (Fig. 6). The high values of the tibia–radius ratios for 0–50% bone length are likely due to the differences in the overall shape of the bones: the broad proximal head of the tibia articulates with both femoral condyles compared to the other smaller head of the radius, which articulates with the capitulum of the humerus. Surprisingly, *A.collaris*, *M.meles*, and *T.taxus* have much higher values of tibia–radius ratios compared to other mustelids, indicating that the internal morphology of the tibia is more robust than the radius in spite of the forelimb being the primary organ used in digging. To understand what this difference between the tibia and radius entails for digging biomechanics, *in vivo* data on the function of both the fore- and hind limbs are needed.

The values of fibula–ulna ratios distinguish mustelid locomotor groups in terms of the total bone tissue in the cross-section (i.e., CSA; Fig. 6), the primary distinction between scansorial mustelids versus fossorial and natatorial mustelids (the former having lower ratios than the latter two). With regard to the distribution of bone tissue in the cross-section (i.e., SMA), the few significant differences among locomotor groups at the proximal and distal ends of the bones tend to distinguish scansorial from non-scansorial mustelids. These results suggest that in comparing the fibula and ulna, a more critical biomechanical factor may be the total amount of bone tissue compared to a specific distribution of that bone tissue within the cross-section, which further suggests that axial compression resistance may be a more critical factor than bending in mustelid limb function. This result is a bit surprising, given how gracile, even spindly, the fibula is; however, the variation in ratio values is likely owed more to the ulna than the fibula. Comparing the curves of the fibular (Fig. 4) and ulnar (Kilbourne and Hutchinson 2019; Fig. 4 therein) cross-sectional traits finds a greater distinction among mustelid locomotor habits for the ulna’s traits. This suggests that the variation in fibula–ulna ratios is influenced more so by the ulna’s trait values and its distinctions among locomotor habits than the fibula’s.

### The evolution of cross-sectional morphology

The strong correlation between mustelid locomotor habit and phylogeny indicates that the differing locomotor habits within Mustelidae are likely tied to a single origin (e.g., natatorial Lutrinae, primarily scansorial Guloninae). Thus, the functional morphology and the biomechanical implications of limb morphology are intimately linked to Mustelidae’s evolutionary history. The results of phylogenetic ANOVAs further corroborate this interpretation, with the inclusion of phylogenetic data in the ANOVAs yielding results that are largely not significant for any of the three bones sampled here. The dearth of significant results for the phylogenetic ANOVAs is an indication that, in spite of an apparent link between morphology and a niche’s biomechanical demands, phylogenetic history nonetheless has a considerable influence upon the morphological diversity of cross-sectional traits in mustelids.

Regarding trait diversification models, the cross-sectional traits for each of our three sampled bones at first glance appears to be primarily characterized by a different model of trait evolution (Fig. 7 and Supplementary Tables S1–S3), femoral traits being characterized by BM3, tibial traits by BM1, and fibular traits by OU3_r and OU4. This would suggest that the cross-sectional morphologies of the femur, tibia, and fibula have each diversified under a separate evolutionary model. Though in most instances, confidence limits of α for the best fitting OU models are able to exclude a value of 0.0, the width of these confidence limits suggests difficulty in fitting the models to our data. Thus, we suggest that our results and their interpretations be viewed somewhat tentatively until further sampling in future work.

Our results suggest that the cross-sectional properties of the femur and tibia by and large did not evolve adaptively, in contrast to the apparent adaptive evolution of the mustelid forelimb skeleton’s external dimensions (Kilbourne, 2017). However, it is worth noting that the OU model dictates that evolution along a phylogeny’s branches is continuously under selective regimes with specific phenotypic optima/adaptive peaks (Hansen, 1997; Cooper et al., 2016). However, morphological traits do not necessarily evolve and persist by being under constant selection, and the OU model appears not to reflect a situation where there is selection for a given trait value at a particular instant of time in the phylogeny with that trait value subsequently becoming fixed. In this scenario, the appearance of new mustelid locomotor habits would be associated with initial selection upon cross-sectional morphology meeting the biomechanics of the niche, with the new cross-sectional morphology at some point becoming fixed after the origin of the locomotor habit. Alternatively, BM evolution of cross-sectional morphology could be associated with evolutionary constraints (O’Meara et al., 2006; Thomas et al., 2006). The likely adaptive evolution of the mustelid forelimb skeleton’s external dimensions is primarily characterized by a selective regime for scansorial mustelids to have more gracile long bones compared to other mustelids (Kilbourne, 2017). The greater gracility of the limb bones of scansorial taxa might necessitate lower values of CSA and SMA (i.e., it being physically impossible to have an overall gracile long bone with a robust cross-section).

Another factor that could lead to the discrepancy in the fitting of trait diversification models to the external and internal morphology of the limb skeleton is a difference in sample size. Kilbourne (2017) notably had a sample size of 41 mustelid species, whereas Kilbourne and Hutchinson (2019) and this study have a sample of 28 species. Notably, the relatively low Akaike weights (<60%) of the best fitting models for many of the cross-sectional traits of the tibia and fibula (Supplementary Tables S1–S3) suggest that our best fitting models do not necessarily have a resounding probability of being the best descriptor of our traits’ variation. This could be due to the *a priori* models themselves being across the board poor fits to our data. Conversely, our sampling of mustelids may be too few to robustly fit our multi-rate and multi-optima models of trait evolution. Thus future work is needed with an increased sample of mustelids, if not carnivorans more broadly, and possibly “data-driven” approaches (Uyeda et al., 2018) to better discern the most likely scenarios of cross-sectional morphology’s diversification and narrow parameter estimates (such as that of α).

Our current results, in combination with those of Kilbourne (2017) and Kilbourne and Hutchinson (2019), suggest that differing traits of the mustelid limb skeleton may diversify by differing evolutionary processes. Moreover, these results together suggest that a single model of trait evolution does not necessarily govern the differing traits that comprise a single organ (e.g., a limb bone). These findings are similar to recent findings by Law et al. (2018), in which the evolutionary rates of two metrics of body size—body mass and length—were found to be decoupled within a subclade containing Ictonychinae, Mustelinae, and Lutrinae. Law et al. (2018) also found that the decoupled diversification of these two traits in mustelids is linked to body elongation and increased clade carrying capacity. Additionally, Law et al. (2019) found that differing regions of the mustelid axial skeleton diversify under disparate models of trait evolution, with head elongation likely undergoing adaptive evolution and the other regions of the axial skeleton likely undergoing a multi-peak BM model. Furthermore, Law et al. (2019) also found that body elongation is associated with a reduced length of the forelimb but not of the hind limb. Together these results strongly suggest that the mustelid body plan can be considered the sum of a suite of several traits diversifying under different models of trait evolution. Whether a “suite-of-traits” model underlies the body plans of other mammalian lineages, and to what extent such a model is characteristic of mammals, remains to be tested. However, this topic is likely of high importance to our understanding of mammalian phenotypic evolution and the evolution of functional systems (e.g., feeding and locomotion).

## Supplementary Material

obz032_Supplementary_Data
