# Supplementary material for: Functional Morphology and Morphological Diversification of Hind Limb Cross-Sectional Traits in Mustelid Mammals
Source: Integr Org Biol. 2020 Jan 8;2(1):obz032. doi: 10.1093/iob/obz032 (PMC7671153; doi:10.1093/iob/obz032)

Supporting Figures

Figure S1. Differences in femoral, tibial, and fibular section modulus among mustelid locomotor habits. MOD_{ML} and MOD_{CC} were measured at 5% increments along each bone's length, with differences among locomotor habit being tested at these increments. A dimensionless value of MOD was calculated dimensionless by taking the third root and dividing by bone length. A colored symbols for a given pairwise comparison indicates a significant difference (Adjusted $P < 0.05$) for that given comparison; a lack of significant pairwise differences for a given increment indicates an overall ANOVA result of $P > 0.0026$ (the Bonferroni-corrected P-value).

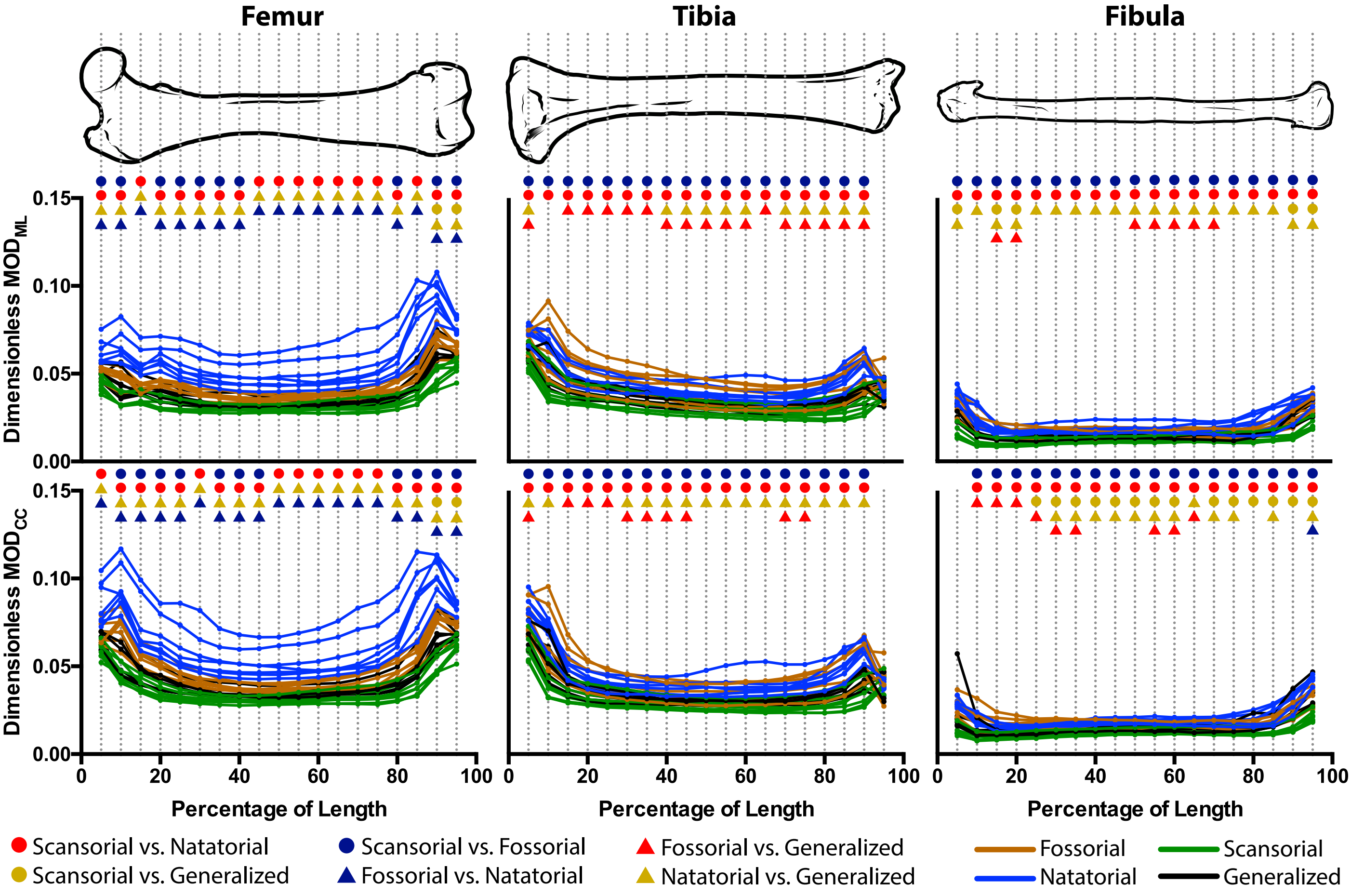

Figure S2. Scale effects in femoral cross-sectional traits at mid-shift with special emphasis on slope. The estimated value for each slope is followed by the 95% confidence limits in parentheses. Both x- and y-axes are on a $\log_{10}$ scale. Please note that the corresponding figure in Kilbourne & Hutchinson (2019) was plotted on a semi-log scale. Scansorial, natatorial, fossorial, and generalist mustelids are in green, blue, brown, and black, respectively.

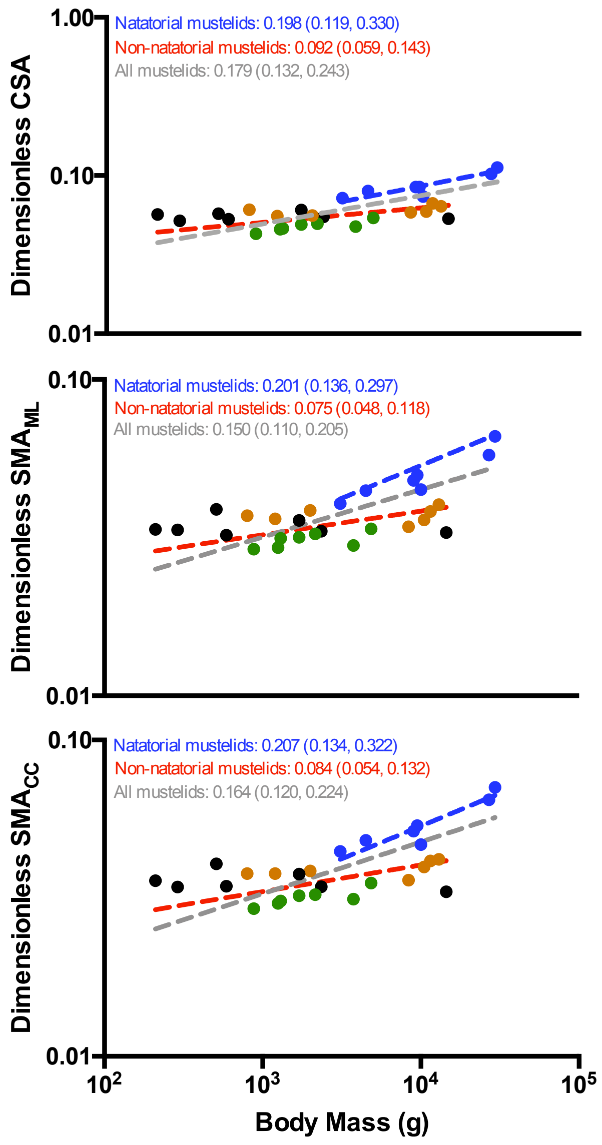

Supplement: obz032_Supplementary_Data [file obz032_supplementary_data.zip › Hindlimb Cross-Section MS Supporting Figures.pdf]
