# Supplementary material for: Functional Morphology and Morphological Diversification of Hind Limb Cross-Sectional Traits in Mustelid Mammals
Source: Integr Org Biol. 2020 Jan 8;2(1):obz032. doi: 10.1093/iob/obz032 (PMC7671153; doi:10.1093/iob/obz032)
Supplement: obz032_Supplementary_Data [file obz032_supplementary_data.zip › Tables A1-A6.docx]

**Table A1**. Parameter output for an Ornstein-Uhlenbeck model with three optima corresponding to fossorial+generalist, natatorial, and scansorial mustelids. %, α, and σ^2^ denote percentage of bone length, the rate/strength of adaptation, and rate of stochastic evolution, respectively. θ_Fos+Gen,_ θ_Nat, and_ θ_Sca_ respectively denote the phenotypic optima (i.e., adaptive peaks) corresponding to fossorial and generalists treated as a single group, natatorial, and scansorial mustelids. Parameter estimates are followed by 95% confidence limits in parentheses.

| **%** | **α** | **σ^2^** | **θ_Fos+Gen_** | **θ_Nat_** | **θ_Sca_** |
| --- | --- | --- | --- | --- | --- |
|  | **Femoral CSA** | | | | |
| **5** | 1.52 (0.183, 15.315) | 2.69 x 10^-4^ (2.161 x 10^-5^, 2.622 x 10^-3^) | 0.10 (0.092, 0.102) | 0.12 (0.117, 0.133) | 0.08 (0.075, 0.093) |
| **10** | 1.91 (0.163, 14.966) | 6.46 x 10^-4^ (4.121 x 10^-5^, 4.304 x 10^-3^) | 0.09 (0.082, 0.096) | 0.13 (0.121, 0.145) | 0.07 (0.052, 0.077) |
| **15** | 1.22 (0.197, 15.329) | 2.84 x 10^-4^ (2.983 x 10^-5^, 3.516 x 10^-3^) | 0.07 (0.068, 0.079) | 0.11 (0.099, 0.117) | 0.06 (0.050, 0.069) |
| **20** | 1.42 (0.187, 15.233) | 2.47 x 10^-4^ (2.365 x 10^-5^, 2.386 x 10^-3^) | 0.07 (0.063, 0.073) | 0.11 (0.099, 0.118) | 0.05 (0.043, 0.061) |
| **25** | 0.90 (0.190,15.219) | 1.75 x 10^-4^ (2.505 x 10^-5^, 2.624 x 10^-3^) | 0.06 (0.058, 0.069) | 0.10 (0.091, 0.109) | 0.05 (0.041, 0.059) |
| **30** | 1.47 (0.181, 15.411) | 3.33 x 10^-4^ (2.788 x 10^-5^, 3.293 x 10^-3^) | 0.06 (0.056, 0.067) | 0.10 (0.088, 0.107) | 0.05 (0.040, 0.060) |
| **35** | 1.51 (0.182, 15.333) | 2.39 x 10^-4^ (2.160 x 10^-5^, 2.043 x 10^-3^) | 0.06 (0.056, 0.066) | 0.09 (0.087, 0.103) | 0.05 (0.042, 0.058) |
| **40** | 1.36 (0.179, 15. 188) | 1.99 x 10^-4^ (1.988 x 10^-5^, 1.959 x 10^-3^) | 0.06 (0.056, 0.065) | 0.09 (0.085, 0.101) | 0.05 (0.041, 0.057) |
| **45** | 1.38 (0.202, 15.394) | 1.86 x 10^-4^ (1.994 x 10^-5^, 1.888 x 10^-3^) | 0.06 (0.054, 0.063) | 0.09 (0.083, 0.098) | 0.05 (0.041, 0.056) |
| **50** | 1.50 (0.191, 15.430) | 1.65 x 10^-4^ (1.591 x 10^-5^, 1.781 x 10^-3^) | 0.06 (0.054, 0.062) | 0.09 (0.083, 0.096) | 0.05 (0.040, 0.055) |
| **55** | 0.83 (0.192, 15.512) | 9.75 x 10^-4^ (1.699 x 10^-5^, 1.900 x 10^-3^) | 0.06 (0.054, 0.062) | 0.09 (0.082, 0.095) | 0.05 (0.039, 0.054) |
| **60** | 0.66 (0.182, 15.457) | 7.47 x 10^-4^ (1.510 x 10^-5^, 1.705 x 10^-3^) | 0.06 (0.054, 0.062) | 0.09 (0.082, 0.096) | 0.05 (0.041, 0.054) |
| **65** | 0.45 (0.188, 15.487) | 5.36 x 10^-4^ (1.692 x 10^-5^, 1.617 x 10^-3^) | 0.06 (0.055, 0.063) | 0.09 (0.083, 0.096) | 0.05 (0.041, 0.056) |
| **70** | 0.87 (0.181, 15.383) | 1.39 x 10^-4^ (2.089 x 10^-5^, 2.423 x 10^-3^) | 0.06 (0.055, 0.064) | 0.09 (0.083, 0.100) | 0.05 (0.040, 0.057) |
| **75** | 0.70 (0.176, 15.430) | 1.29 x 10^-4^ (2.310 x 10^-5^, 2.616 x 10^-3^) | 0.06 (0.055, 0.065) | 0.09 (0.087, 0.104) | 0.05 (0.040, 0.058) |
| **80** | 0.60 (0.173, 15.467) | 1.47 x 10^-4^ (3.274 x 10^-5^, 3.689 x 10^-3^) | 0.06 (0.058, 0.070) | 0.11 (0.097, 0.117) | 0.05 (0.041, 0.062) |
| **85** | 6.52 (0.224, 15.581) | 2.71 x 10^-4^ (6.787 x 10^-5^, 7.262 x 10^-3^) | 0.08 (0.068, 0.084) | 0.14 (0.127, 0.152) | 0.06 (0.043, 0.067) |
| **90** | 1.96 (0.216, 15.319) | 7.29 x 10^-4^ (5.426 x 10^-5^, 5.486 x 10^-3^) | 0.11 (0.102, 0.117) | 0.16 (0.146, 0.168) | 0.08 (0.064, 0.088) |
| **95** | 1.04 (0.171, 15.093) | 1.36 x 10^-4^ (1.515 x 10^-5^, 1.805 x 10^-3^) | 0.10 (0.099, 0.109) | 0.13 (0.124, 0.138) | 0.09 (0.079, 0.095) |
|  | **Femoral SMA_ML_** | | | | |
| **5** | 2.03 (0.204, 15.141) | 7.37 x 10^-5^ (5.363 x 10^-6^, 5.411 x 10^-4^) | 0.05 (0.053, 0.057) | 0.07 (0.063, 0.069) | 0.05 (0.042, 0.050) |
| **10** | 2.03 (0.191, 15.592) | 2.21 x 10^-4^ (1.667 x 10^-5^, 1.699 x 10^-3^) | 0.05 (0.046, 0.053) | 0.07 (0.063, 0.075) | 0.04 (0.030, 0.044) |
| **15** | 1.13 (0.175, 15.160) | 5.63 x 10^-5^ (6.234 x 10^-6^, 7.466 x 10^-4^) | 0.04 (0.040, 0.046) | 0.06 (0.055, 0.064) | 0.04 (0.033, 0.042) |
| **20** | 0.84 (0.173, 15.426) | 4.24 x 10^-5^ (5.986 x 10^-6^, 6.589 x 10^-4^) | 0.04 (0.040, 0.045) | 0.06 (0.059, 0.069) | 0.03 (0.029, 0.039) |
| **25** | 0.41 (0.167, 15.146) | 2.64 x 10^-5^ (7.921 x 10^-6^, 8.748 x 10^-4^) | 0.04 (0.037, 0.043) | 0.06 (0.053, 0.064) | 0.03 (0.027, 0.038) |
| **30** | 1.13 (0.167, 15.309) | 5.72 x 10^-5^ (5.964 x 10^-6^, 7.168 x 10^-4^) | 0.04 (0.035, 0.041) | 0.05 (0.050, 0.059) | 0.03 (0.027, 0.037) |
| **35** | 1.38 (0.189, 15.334) | 5.08 x 10^-5^ (4.607 x 10^-6^, 5.414 x 10^-4^) | 0.04 (0.034, 0.039) | 0.05 (0.048, 0.056) | 0.03 (0.027, 0.035) |
| **40** | 1.42 (0.184, 15.258) | 4.91 x 10^-5^ (4.618 x 10^-6^, 4.845 x 10^-4^) | 0.04 (0.034, 0.038) | 0.05 (0.047, 0.054) | 0.03 (0.027, 0.035) |
| **45** | 1.37 (0.177, 15.270) | 5.11 x 10^-5^ (5.033 x 10^-6^, 5.280 x 10^-4^) | 0.04 (0.033, 0.038) | 0.05 (0.047, 0.055) | 0.03 (0.027, 0.035) |
| **50** | 1.27 (0.185, 15.210) | 5.18 x 10^-5^ (5.601 x 10^-6^, 5.952 x 10^-4^) | 0.04 (0.034, 0.038) | 0.05 (0.047, 0.055) | 0.03 (0.027, 0.035) |
| **55** | 0.97 (0.191, 15.166) | 4.83 x 10^-5^ (6.983 x 10^-6^, 6.723 x 10^-4^) | 0.04 (0.034, 0.039) | 0.05 (0.048, 0.057) | 0.03 (0.027, 0.036) |
| **60** | 0.93 (0.154, 15.109) | 4.99 x 10^-5^ (6.592 x 10^-6^, 7.603 x 10^-4^) | 0.04 (0.034, 0.040) | 0.05 (0.048, 0.057) | 0.03 (0.026, 0.036) |
| **65** | 1.04 (0.169, 15.445) | 6.09 x 10^-5^ (7.335 x 10^-6^, 8.801 x 10^-4^) | 0.04 (0.034, 0.040) | 0.05 (0.049, 0.059) | 0.03 (0.026, 0.036) |
| **70** | 1.10 (0.170, 15.112) | 8.20 x 10^-5^ (8.852 x 10^-6^, 1.061 x 10^-3^) | 0.04 (0.035, 0.042) | 0.06 (0.051, 0.061) | 0.03 (0.025, 0.037) |
| **75** | 1.24 (0.146, 14.980) | 9.97 x 10^-5^ (9.570 x 10^-6^, 1.091 x 10^-3^) | 0.04 (0.036, 0.043) | 0.06 (0.054, 0.065) | 0.03 (0.026, 0.038) |
| **80** | 1.74 (0.182, 15.241) | 1.72 x 10^-4^ (1.245 x 10^-5^, 1.409 x 10^-3^) | 0.04 (0.040, 0.047) | 0.07 (0.060, 0.072) | 0.03 (0.028, 0.040) |
| **85** | 6.46 (0.230, 15.873) | 1.32 x 10^-3^ (2.944 x 10^-5^, 3.655 x 10^-3^) | 0.05 (0.046, 0.057) | 0.09 (0.082, 0.098) | 0.04 (0.030, 0.047) |
| **90** | 2.16 (0.206, 15.280) | 2.43 x 10^-4^ (1.795 x 10^-5^, 1.755 x 10^-3^) | 0.07 (0.070, 0.078) | 0.10 (0.098, 0.111) | 0.05 (0.044, 0.058) |
| **95** | 1.31 (0.207, 15.224) | 4.22 x 10^-5^ (4.525 x 10^-6^, 4.555 x 10^-4^) | 0.07 (0.067, 0.071) | 0.09 (0.083, 0.091) | 0.06 (0.055, 0.063) |
|  | **Femoral SMA_CC_** | | | | |
| **5** | 1.74 (0.165, 15.238) | 2.29 x 10^-4^ (1.654 x 10^-5^, 2.055 x 10^-3^) | 0.08 (0.074, 0.083) | 0.10 (0.096, 0.110) | 0.07 (0.060, 0.075) |
| **10** | 0.21 (0.094, 14.618) | 7.96 x 10^-5^ (3.203 x 10^-5^, 3.824 x 10^-3^) | 0.08 (0.068, 0.084) | 0.12 (0.099, 0.131) | 0.05 (0.031, 0.068) |
| **15** | 0.29 (0.138, 14.978) | 7.85 x 10^-5^ (2.823 x 10^-5^, 3.598 x 10^-3^) | 0.06 (0.051, 0.063) | 0.09 (0.076, 0.098) | 0.04 (0.029, 0.055) |
| **20** | 0.24 (0.107, 14.931) | 3.64 x 10^-5^ (1.384 x 10^-5^, 1.709 x 10^-3^) | 0.05 (0.047, 0.057) | 0.08 (0.070, 0.089) | 0.04 (0.026, 0.047) |
| **25** | 0.33 (0.168, 14.938) | 4.74 x 10^-5^ (1.752 x 10^-5^, 1.764 x 10^-3^) | 0.05 (0.041, 0.050) | 0.07 (0.062, 0.079) | 0.03 (0.025, 0.044) |
| **30** | 0.55 (0.166, 15.112) | 6.18 x 10^-5^ (1.331 x 10^-5^, 1.512 x 10^-3^) | 0.04 (0.037, 0.046) | 0.06 (0.057, 0.071) | 0.03 (0.025, 0.040) |
| **35** | 0.84 (0.170, 14.991) | 5.16 x 10^-5^ (7.585 x 10^-6^, 8.015 x 10^-4^) | 0.04 (0.036, 0.042) | 0.06 (0.054, 0.064) | 0.03 (0.026, 0.037) |
| **40** | 0.94 (0.183, 15.133) | 4.72 x 10^-5^ (6.116 x 10^-6^, 6.242 x 10^-4^) | 0.04 (0.035, 0.041) | 0.06 (0.052, 0.061) | 0.03 (0.027, 0.036) |
| **45** | 0.80 (0.192, 15.415) | 3.72 x 10^-5^ (6.406 x 10^-6^, 6.524 x 10^-4^) | 0.04 (0.035, 0.040) | 0.06 (0.051, 0.060) | 0.03 (0.027, 0.036) |
| **50** | 0.48 (0.183, 15.328) | 2.36 x 10^-5^ (6.578 x 10^-6^, 7.452 x 10^-4^) | 0.04 (0.035, 0.040) | 0.06 (0.051, 0.060) | 0.03 (0.027, 0.036) |
| **55** | 0.42 (0.181, 15.023) | 2.46 x 10^-5^ (7.953 x 10^-6^, 8.184 x 10^-4^) | 0.04 (0.035, 0.041) | 0.06 (0.052, 0.062) | 0.03 (0.027, 0.037) |
| **60** | 0.40 (0.173, 15.199) | 2.96 x 10^-5^ (9.127 x 10^-6^, 1.013 x 10^-3^) | 0.04 (0.036, 0.043) | 0.06 (0.053, 0.064) | 0.03 (0.027, 0.039) |
| **65** | 0.39 (0.164, 15.093) | 3.67 x 10^-5^ (1.100 x 10^-5^, 1.308 x 10^-3^) | 0.04 (0.037, 0.044) | 0.06 (0.055, 0.068) | 0.03 (0.027, 0.040) |
| **70** | 0.34 (0.162, 15.154) | 4.38 x 10^-5^ (1.462 x 10^-5^, 1.810 x 10^-3^) | 0.04 (0.038, 0.046) | 0.06 (0.058, 0.073) | 0.03 (0.026, 0.042) |
| **75** | 0.36 (0.159, 15.158) | 5.21 x 10^-5^ (1.669 x 10^-5^, 1.916 x 10^-3^) | 0.04 (0.039, 0.049) | 0.07 (0.062, 0.078) | 0.03 (0.026, 0.043) |
| **80** | 0.35 (0.166, 15.137) | 6.07 x 10^-5^ (2.089 x 10^-5^, 2.217 x 10^-3^) | 0.05 (0.044, 0.054) | 0.08 (0.070, 0.088) | 0.04 (0.027, 0.046) |
| **85** | 2.00 (0.206, 15.670) | 5.03 x 10^-4^ (3.938 x 10^-5^, 3.979 x 10^-3^) | 0.06 (0.052, 0.064) | 0.10 (0.092, 0.111) | 0.04 (0.033, 0.051) |
| **90** | 1.78 (0.196, 15.449) | 2.71 x 10^-4^ (2.267 x 10^-5^, 2.238 x 10^-3^) | 0.08 (0.076, 0.085) | 0.11 (0.106, 0.120) | 0.06 (0.045, 0.065) |
| **95** | 1.41 (0.165, 15.155) | 8.98 x 10^-5^ (7.395 x 10^-6^, 8.667 x 10^-4^) | 0.08 (0.075, 0.080) | 0.10 (0.092, 0.102) | 0.07 (0.061, 0.072) |
|  | **Tibial CSA** | | | | |
| **5** | 0.21 (0.085, 13.923) | 7.86 x 10^-5^ (3.093 x 10^-5^, 3.933 x 10^-3^) | 0.11 (0.101, 0.118) | 0.13 (0.118, 0.146) | 0.09 (0.074, 0.109) |
| **10** | 2.31 x 10^-4^ (2.061 x 10^-9^, 0.358) | 2.60 x 10^-5^ (1.390 x 10^-5^, 1.268 x 10^-4^) | 0.11 (0.083, 0.125) | 0.14 (0.064, 0.147) | 0.03 (0.056, 0.158) |
| **15** | 7.85 x 10^-6^ (2.061 x 10^-9^, 0.382) | 9.66 x 10^-6^ (5.247 x 10^-6^, 4.546 x 10^-5^) | 0.08 (0.070, 0.094) | 0.10 (0.057, 0.108) | 0.04 (0.049, 0.115) |
| **20** | 3.08 x 10^-3^ (2.061 x 10^-9^, 0.366) | 7.35 x 10^-6^ (3.717 x 10^-6^, 3.165 x 10^-5^) | 0.08 (0.065, 0.086) | 0.09 (0.055, 0.097) | 0.04 (0.045, 0.103) |
| **25** | 2.37 x 10^-4^ (2.061 x 10^-9^, 0.459) | 5.75 x 10^-6^ (2.990 x 10^-6^, 3.064 x 10^-5^) | 0.07 (0.062, 0.082) | 0.09 (0.051, 0.092) | 0.04 (0.045, 0.097) |
| **30** | 1.31 x 10^-4^ (2.061 x 10^-9^, 0.386) | 5.19 x 10^-6^ (2.823 x 10^-6^, 2.394 x 10^-5^) | 0.07 (0.062, 0.079) | 0.09 (0.051, 0.090) | 0.04 (0.044, 0.094) |
| **35** | 5.49 x 10^-3^ (2.061 x 10^-9^, 0.476) | 5.69 x 10^-6^ (2.960 x 10^-6^, 2.781 x 10^-5^) | 0.07 (0.060, 0.077) | 0.08 (0.051, 0.088) | 0.04 (0.043, 0.091) |
| **40** | 3.53 x 10^-3^ (2.061 x 10^-9^, 0.418) | 5.77 x 10^-6^ (3.002 x 10^-6^, 3.091 x 10^-5^) | 0.07 (0.058, 0.077) | 0.09 (0.050, 0.089) | 0.04 (0.041, 0.093) |
| **45** | 3.16 x 10^-3^ (2.061 x 10^-9^, 0.359) | 6.32 x 10^-6^ (3.412 x 10^-6^, 2.817 x 10^-5^) | 0.07 (0.058, 0.078) | 0.09 (0.049, 0.091) | 0.04 (0.041, 0.091) |
| **50** | 1.34 x 10^-3^ (2.061 x 10^-9^, 0.391) | 6.65 x 10^-6^ (3.610 x 10^-6^, 3.076 x 10^-5^) | 0.07 (0.058, 0.077) | 0.09 (0.046, 0.088) | 0.04 (0.037, 0.093) |
| **55** | 2.51 x 10^-4^ (2.061 x 10^-9^, 0.415) | 6.95 x 10^-6^ (3.660 x 10^-6^, 3.360 x 10^-5^) | 0.07 (0.056, 0.077) | 0.09 (0.045, 0.087) | 0.04 (0.036, 0.095) |
| **60** | 8.65 x 10^-5^ (2.061 x 10^-9^, 0.438) | 6.84 x 10^-6^ (3.635 x 10^-6^, 3.368 x 10^-5^) | 0.07 (0.055, 0.076) | 0.08 (0.043, 0.089) | 0.04 (0.036, 0.093) |
| **65** | 2.07 x 10^-9^ (2.061 x 10^-9^, 0.434) | 5.74 x 10^-6^ (2.954 x 10^-6^, 2.927 x 10^-5^) | 0.06 (0.055, 0.073) | 0.08 (0.043, 0.084) | 0.03 (0.038, 0.090) |
| **70** | 2.00 x 10^-5^ (2.061 x 10^-9^, 0.394) | 4.78 x 10^-6^ (2.573 x 10^-6^, 2.326 x 10^-5^) | 0.06 (0.056, 0.072) | 0.08 (0.044, 0.079) | 0.03 (0.041, 0.086) |
| **75** | 8.80 x 10^-5^ (2.061 x 10^-9^, 0.398) | 5.21 x 10^-6^ (2.768 x 10^-6^, 2.505 x 10^-5^) | 0.06 (0.054, 0.072) | 0.08 (0.047, 0.082) | 0.03 (0.038, 0.087) |
| **80** | 2.08 x 10^-4^ (2.061 x 10^-9^, 0.412) | 6.05 x 10^-6^ (3.365 x 10^-6^, 2.707 x 10^-5^) | 0.07 (0.055, 0.075) | 0.09 (0.045, 0.085) | 0.03 (0.038, 0.092) |
| **85** | 0.01 (2.061 x 10^-9^, 0.431) | 1.11 x 10^-5^ (5.349 x 10^-6^, 4.656 x 10^-5^) | 0.07 (0.059, 0.082) | 0.10 (0.053, 0.100) | 0.03 (0.031, 0.093) |
| **90** | 0.06 (2.061 x 10^-9^, 1.776) | 2.53 x 10^-5^ (1.044 x 10^-5^, 2.709 x 10^-4^) | 0.08 (0.070, 0.092) | 0.12 (0.082, 0.132) | 0.04 (0.025, 0.091) |
| **95** | 6.23 (0.216, 15.698) | 1.73 x 10^-3^ (4.509 x 10^-5^, 4.777 x 10^-3^) | 0.07 (0.067, 0.079) | 0.07 (0.058, 0.076) | 0.07 (0.061, 0.079) |
|  | **Tibial SMA_ML_** | | | | |
| **5** | 0.06 (2.061 x 10^-9^, 0.986) | 5.55 x 10^-6^ (2.106 x 10^-6^, 3.916 x 10^-5^) | 0.08 (0.071, 0.081) | 0.09 (0.071, 0.093) | 0.06 (0.047, 0.079) |
| **10** | 1.99 x 10^-4^ (2.061 x 10^-9^, 0.424) | 1.36 x 10^-5^ (7.125 x 10^-6^, 6.301 x 10^-5^) | 0.08 (0.071, 0.081) | 0.09 (0.046, 0.110) | 0.03 (0.037, 0.121) |
| **15** | 2.06 x 10^-9^ (2.061 x 10^-9^, 0.435) | 5.80 x 10^-6^ (2.965 x 10^-6^, 2.979 x 10^-5^) | 0.06 (0.055, 0.073) | 0.07 (0.042, 0.085) | 0.03 (0.038, 0.091) |
| **20** | 2.25 x 10^-9^ (2.061 x 10^-9^, 0.405) | 4.08 x 10^-6^ (2.235 x 10^-6^, 1.790 x 10^-5^) | 0.06 (0.049, 0.065) | 0.06 (0.039, 0.076) | 0.03 (0.036, 0.080) |
| **25** | 1.07 x 10^-5^ (2.061 x 10^-9^, 0.377) | 3.26 x 10^-6^ (1.856 x 10^-6^, 1.593 x 10^-5^) | 0.05 (0.046, 0.061) | 0.06 (0.039, 0.070) | 0.03 (0.035, 0.073) |
| **30** | 2.06 x 10^-9^ (2.061 x 10^-9^, 0.394) | 2.64 x 10^-6^ (1.435 x 10^-6^, 1.230 x 10^-5^) | 0.05 (0.044, 0.058) | 0.06 (0.037, 0.065) | 0.03 (0.031, 0.068) |
| **35** | 8.74 x 10^-6^ (2.061 x 10^-9^, 0.437) | 2.46 x 10^-6^ (1.419 x 10^-6^, 1.489 x 10^-5^) | 0.05 (0.041, 0.055) | 0.05 (0.034, 0.062) | 0.03 (0.032, 0.065) |
| **40** | 5.83 x 10^-5^ (2.061 x 10^-9^, 0.376) | 2.45 x 10^-6^ (1.374 x 10^-6^, 1.162 x 10^-5^) | 0.05 (0.040, 0.052) | 0.05 (0.033, 0.058) | 0.03 (0.028, 0.063) |
| **45** | 7.45 x 10^-5^ (2.061 x 10^-9^, 0.471) | 2.41 x 10^-6^ (1.395 x 10^-6^, 1.292 x 10^-5^) | 0.04 (0.038, 0.051) | 0.05 (0.032, 0.058) | 0.03 (0.028, 0.061) |
| **50** | 1.18 x 10^-4^ (2.061 x 10^-9^, 0.455) | 2.44 x 10^-6^ (1.298 x 10^-6^, 1.206 x 10^-5^) | 0.04 (0.036, 0.048) | 0.05 (0.030, 0.057) | 0.02 (0.027, 0.059) |
| **55** | 5.67 x 10^-4^ (2.061 x 10^-9^, 0.432) | 2.50 x 10^-6^ (1.349 x 10^-6^, 1.291 x 10^-5^) | 0.04 (0.035, 0.047) | 0.05 (0.027, 0.055) | 0.02 (0.024, 0.058) |
| **60** | 1.43 x 10^-3^ (2.061 x 10^-9^, 0.460) | 2.41 x 10^-6^ (1.231 x 10^-6^, 1.223 x 10^-5^) | 0.04 (0.034, 0.046) | 0.05 (0.028, 0.053) | 0.02 (0.023, 0.056) |
| **65** | 5.14 x 10^-4^ (2.061 x 10^-9^, 0.392) | 2.17 x 10^-6^ (1.159 x 10^-6^, 9.249 x 10^-6^) | 0.04 (0.033, 0.045) | 0.05 (0.027, 0.051) | 0.02 (0.023, 0.055) |
| **70** | 6.25 x 10^-3^ (2.061 x 10^-9^, 0.411) | 2.06 x 10^-6^ (1.068 x 10^-6^, 1.124 x 10^-5^) | 0.04 (0.033, 0.044) | 0.04 (0.027, 0.050) | 0.02 (0.024, 0.052) |
| **75** | 0.02 (2.061 x 10^-9^, 0.529) | 2.49 x 10^-6^ (1.283 x 10^-6^, 1.188 x 10^-5^) | 0.04 (0.033, 0.044) | 0.05 (0.030, 0.052) | 0.02 (0.020, 0.049) |
| **80** | 0.02 (2.061 x 10^-9^, 0.463) | 3.09 x 10^-6^ (1.555 x 10^-6^, 1.533 x 10^-5^) | 0.04 (0.035, 0.045) | 0.05 (0.031, 0.054) | 0.02 (0.020, 0.052) |
| **85** | 0.03 (2.061 x 10^-9^, 0.590) | 5.54 x 10^-6^ (2.510 x 10^-6^, 2.843 x 10^-5^) | 0.04 (0.038, 0.052) | 0.06 (0.037, 0.066) | 0.02 (0.016, 0.056) |
| **90** | 0.02 (2.061 x 10^-9^, 0.606) | 6.89 x 10^-6^ (3.357 x 10^-6^, 4.006 x 10^-5^) | 0.05 (0.045, 0.062) | 0.08 (0.042, 0.079) | 0.03 (0.024, 0.069) |
| **95** | 0.38 (0.142, 14.930) | 2.68 x 10^-5^ (7.722 x 10^-6^, 9.382 x 10^-4^) | 0.05 (0.044, 0.051) | 0.05 (0.040, 0.051) | 0.04 (0.037, 0.049) |
|  | **Tibial SMA_CC_** | | | | |
| **5** | 0.02 (2.061 x 10^-9^, 0.631) | 8.97 x 10^-6^ (4.424 x 10^-6^, 5.770 x 10^-5^) | 0.09 (0.078, 0.097) | 0.11 (0.072, 0.114) | 0.05 (0.052, 0.109) |
| **10** | 7.71 x 10^-5^ (2.061 x 10^-9^, 0.395) | 1.47 x 10^-5^ (7.752 x 10^-6^, 7.134 x 10^-5^) | 0.08 (0.062, 0.091) | 0.10 (0.045, 0.111) | 0.03 (0.031, 0.120) |
| **15** | 2.06 x 10^-9^ (2.061 x 10^-9^, 0.424) | 5.33 x 10^-6^ (3.064 x 10^-6^, 2.689 x 10^-5^) | 0.06 (0.046, 0.066) | 0.06 (0.038, 0.074) | 0.03 (0.032, 0.079) |
| **20** | 0.01 (2.061 x 10^-9^, 0.502) | 3.90 x 10^-6^ (2.005 x 10^-6^, 2.029 x 10^-5^) | 0.05 (0.040, 0.054) | 0.05 (0.034, 0.061) | 0.02 (0.023, 0.060) |
| **25** | 0.01 (2.061 x 10^-9^, 0.421) | 2.91 x 10^-6^ (1.557 x 10^-6^, 1.519 x 10^-5^) | 0.04 (0.036, 0.049) | 0.05 (0.030, 0.057) | 0.02 (0.023, 0.058) |
| **30** | 0.01 (2.061 x 10^-9^, 0.474) | 2.41 x 10^-6^ (1.264 x 10^-6^, 1.289 x 10^-5^) | 0.04 (0.035, 0.046) | 0.05 (0.028, 0.053) | 0.03 (0.023, 0.053) |
| **35** | 0.02 01 (2.061 x 10^-9^, 0.510) | 2.18 x 10^-6^ (1.055 x 10^-6^, 1.142 x 10^-5^) | 0.04 (0.033, 0.043) | 0.04 (0.030, 0.050) | 0.03 (0.022, 0.049) |
| **40** | 0.02 (2.061 x 10^-9^, 1.009) | 2.17 x 10^-6^ (1.087 x 10^-6^, 1.957 x 10^-5^) | 0.04 (0.032, 0.041) | 0.04 (0.029, 0.050) | 0.03 (0.021, 0.047) |
| **45** | 0.02 (2.061 x 10^-9^, 0.695) | 2.22 x 10^-6^ (1.122 x 10^-6^, 1.606 x 10^-5^) | 0.04 (0.032, 0.041) | 0.05 (0.029, 0.049) | 0.03 (0.020, 0.045) |
| **50** | 0.01 (2.061 x 10^-9^, 0.448) | 2.06 x 10^-6^ (1.093 x 10^-6^, 1.094 x 10^-5^) | 0.04 (0.031, 0.042) | 0.05 (0.026, 0.049) | 0.02 (0.021, 0.050) |
| **55** | 7.18 x 10^-5^ (2.061 x 10^-9^, 0.385) | 2.13 x 10^-6^ (1.146 x 10^-6^, 1.046 x 10^-5^) | 0.04 (0.031, 0.042) | 0.05 (0.024, 0.049) | 0.02 (0.021, 0.052) |
| **60** | 2.08 x 10^-9^ (2.061 x 10^-9^, 0.382) | 2.16 x 10^-6^ (1.143 x 10^-6^, 9.972 x 10^-6^) | 0.04 (0.031, 0.043) | 0.05 (0.025, 0.050) | 0.02 (0.021, 0.053) |
| **65** | 2.27 x 10^-9^ (2.061 x 10^-9^, 0.439) | 2.26 x 10^-6^ (1.251 x 10^-6^, 1.171 x 10^-5^) | 0.04 (0.032, 0.044) | 0.05 (0.026, 0.051) | 0.02 (0.021, 0.055) |
| **70** | 2.07 x 10^-9^ (2.061 x 10^-9^, 0.360) | 2.27 x 10^-6^ (1.256 x 10^-6^, 1.023 x 10^-5^) | 0.04 (0.033, 0.045) | 0.05 (0.027, 0.051) | 0.02 (0.023, 0.055) |
| **75** | 1.54 x 10^-4^ (2.061 x 10^-9^, 0.387) | 2.82 x 10^-6^ (1.495 x 10^-6^, 1.293 x 10^-5^) | 0.04 (0.034, 0.048) | 0.05 (0.027, 0.055) | 0.02 (0.023, 0.059) |
| **80** | 6.53 x 10^-4^ (2.061 x 10^-9^, 0.378) | 3.81 x 10^-6^ (2.103 x 10^-6^, 1.725 x 10^-5^) | 0.04 (0.037, 0.051) | 0.06 (0.029, 0.060) | 0.02 (0.023, 0.065) |
| **85** | 0.01 (2.061 x 10^-9^, 0.600) | 6.72 x 10^-6^ (3.513 x 10^-6^, 3.952 x 10^-5^) | 0.05 (0.041, 0.059) | 0.07 (0.035, 0.073) | 0.02 (0.021, 0.068) |
| **90** | 0.04 (2.061 x 10^-9^, 0.904) | 8.21 x 10^-6^ (3.538 x 10^-6^, 5.042 x 10^-5^) | 0.06 (0.052, 0.067) | 0.08 (0.049, 0.083) | 0.02 (0.021, 0.067) |
| **95** | 1.85 (0.186, 15.588) | 1.90 x 10^-4^ (1.571 x 10^-6^, 1.694 x 10^-5^) | 0.05 (0.042, 0.050) | 0.04 (0.037, 0.049) | 0.05 (0.039, 0.051) |
|  | **Fibular CSA** | | | | |
| **5** | 0.12 (0.060 8.108) | 1.08 x 10^-5^ (5.188 x 10^-6^, 4.285 x 10^-4^) | 0.05 (0.041, 0.050) | 0.06 (0.047, 0.062) | 0.02 (0.009, 0.035) |
| **10** | 3.39 x 10^-5^ (2.061 x 10^-9^, 0.389) | 2.29 x 10^-6^ (1.297 x 10^-6^, 1.073 x 10^-5^) | 0.04 (0.030, 0.042) | 0.05 (0.023, 0.049) | 0.01 (0.020, 0.052) |
| **15** | 3.67 x 10^-4^ (2.061 x 10^-9^, 0.388) | 1.37 x 10^-6^ (7.988 x 10^-7^, 6.624 x 10^-6^) | 0.03 (0.027, 0.036) | 0.04 (0.021, 0.042) | 0.01 (0.019, 0.044) |
| **20** | 7.80 x 10^-4^ (2.061 x 10^-9^, 0.372) | 1.09 x 10^-6^ (6.377 x 10^-7^, 5.088 x 10^-6^) | 0.03 (0.026, 0.035) | 0.04 (0.022, 0.039) | 0.01 (0.020, 0.042) |
| **25** | 7.23 x 10^-3^ (2.061 x 10^-9^, 0.487) | 1.09 x 10^-6^ (5.919 x 10^-7^, 5.817 x 10^-6^) | 0.03 (0.027, 0.035) | 0.04 (0.023, 0.040) | 0.02 (0.020, 0.043) |
| **30** | 1.89 x 10^-5^ (2.061 x 10^-9^, 0.412) | 9.94 x 10^-7^ (5.799 x 10^-7^, 4.969 x 10^-6^) | 0.03 (0.028, 0.036) | 0.04 (0.023, 0.041) | 0.02 (0.020, 0.042) |
| **35** | 2.79 x 10^-3^ (2.061 x 10^-9^, 0.463) | 1.14 x 10^-6^ (6.010 x 10^-7^, 5.906 x 10^-6^) | 0.03 (0.028, 0.036) | 0.04 (0.023, 0.041) | 0.02 (0.021, 0.044) |
| **40** | 0.04 (2.061 x 10^-9^, 0.579) | 1.59 x 10^-6^ (7.720 x 10^-7^, 8.407 x 10^-6^) | 0.03 (0.028, 0.035) | 0.04 (0.027, 0.042) | 0.02 (0.016, 0.035) |
| **45** | 0.02 (2.061 x 10^-9^, 0.746) | 1.28 x 10^-6^ (6.042 x 10^-7^, 9.648 x 10^-6^) | 0.03 (0.028, 0.035) | 0.04 (0.027, 0.042) | 0.02 (0.018, 0.039) |
| **50** | 0.04 (2.061 x 10^-9^, 0.734) | 1.32 x 10^-6^ (6.251 x 10^-7^, 8.775 x 10^-6^) | 0.03 (0.029, 0.035) | 0.04 (0.029, 0.042) | 0.02 (0.018, 0.034) |
| **55** | 0.02 (2.061 x 10^-9^, 0.490) | 1.18 x 10^-6^ (6.056 x 10^-7^, 6.093 x 10^-6^) | 0.03 (0.029, 0.036) | 0.04 (0.027, 0.042) | 0.02 (0.019, 0.038) |
| **60** | 0.06 (2.607 x 10^-9^, 3.322) | 1.60 x 10^-6^ (7.658 x 10^-7^, 3.548 x 10^-5^) | 0.03 (0.029, 0.034) | 0.04 (0.031, 0.042) | 0.02 (0.016, 0.033) |
| **65** | 0.11 (0.036, 9.449) | 2.82 x 10^-6^ (1.297 x 10^-6^, 1.374 x 10^-4^) | 0.03 (0.028, 0.033) | 0.04 (0.032, 0.042) | 0.02 (0.016, 0.029) |
| **70** | 0.14 (0.051, 8.187) | 3.64 x 10^-6^ (1.748 x 10^-6^, 1.317 x 10^-4^) | 0.03 (0.028, 0.033) | 0.04 (0.033, 0.042) | 0.02 (0.016, 0.028) |
| **75** | 0.26 (0.139, 14.512) | 7.75 x 10^-6^ (2.676 x 10^-6^, 3.407 x 10^-4^) | 0.03 (0.028, 0.032) | 0.04 (0.034, 0.041) | 0.02 (0.018, 0.026) |
| **80** | 0.36 (0.145, 14.760) | 1.35 x 10^-5^ (4.154 x 10^-6^, 4.606 x 10^-4^) | 0.03 (0.028, 0.033) | 0.04 (0.036, 0.044) | 0.02 (0.018, 0.027) |
| **85** | 0.27 (0.106, 14.739) | 1.33 x 10^-5^ (4.673 x 10^-6^, 5.758 x 10^-4^) | 0.03 (0.031, 0.037) | 0.05 (0.041, 0.051) | 0.02 (0.016, 0.029) |
| **90** | 0.35 (0.131, 14.195) | 3.06 x 10^-5^ (7.644 x 10^-6^, 9.866 x 10^-4^) | 0.04 (0.038, 0.045) | 0.06 (0.049, 0.062) | 0.02(0.016, 0.032) |
| **95** | 0.23 (0.122, 9.692) | 1.85 x 10^-5^ (7.318 x 10^-6^, 5.653 x 10^-4^) | 0.05 (0.049, 0.057) | 0.07 (0.065, 0.079) | 0.03 (0.024, 0.043) |
|  | **Fibular SMA_ML_** | | | | |
| **5** | 0.26 (0.129, 13.774) | 1.14 x 10^-5^ (4.265 x 10^-6^, 4.182 x 10^-4^) | 0.04 (0.033, 0.039) | 0.04 (0.039, 0.049) | 0.02 (0.008, 0.023) |
| **10** | 0.19 (0.079, 13.120) | 1.24 x 10^-5^ (4.937 x 10^-6^, 5.108 x 10^-4^) | 0.02 (0.019, 0.026) | 0.03 (0.024, 0.037) | 8.88 x 10^-3^ (0.001, 0.017) |
| **15** | 0.08 (0.005, 8.111) | 1.75 x 10^-6^ (7.867 x 10^-7^, 7.351 x 10^-5^) | 0.02 (0.015, 0.020) | 0.02 (0.017, 0.026) | 7.56 x 10^-3^ (0.002, 0.017) |
| **20** | 0.06 (2.061 x 10^-9^, 5.564) | 9.81 x 10^-7^ (4.645 x 10^-7^, 3.383 x 10^-5^) | 0.02 (0.014, 0.018) | 0.02 (0.015, 0.023) | 7.79 x 10^-3^ (0.005, 0.017) |
| **25** | 0.03 (2.061 x 10^-9^, 0.702) | 7.57 x 10^-7^ (3.729 x 10^-7^, 5.056 x 10^-5^) | 0.02 (0.014, 0.019) | 0.02 (0.013, 0.024) | 8.65 x 10^-3^ (0.006, 0.020) |
| **30** | 2.30 x 10^-9^ (2.061 x 10^-9^, 0.398) | 5.59 x 10^-7^ (3.083 x 10^-7^, 2.760 x 10^-6^) | 0.02 (0.014, 0.020) | 0.02 (0.011, 0.024) | 8.91 x 10^-3^ (0.009, 0.025) |
| **35** | 2.29 x 10^-9^ (2.061 x 10^-9^, 0.462) | 5.79 x 10^-7^ (3.403 x 10^-7^, 3.124 x 10^-6^) | 0.02 (0.015, 0.020) | 0.02 (0.011, 0.024) | 8.89 x 10^-3^ (0.009, 0.026) |
| **40** | 0.08 (5.687 x 10^-9^, 4.667) | 1.09 x 10^-6^ (4.680 x 10^-7^, 2.755 x 10^-5^) | 0.02 (0.015, 0.018) | 0.02 (0.016, 0.023) | 9.71 x 10^-3^ (0.005, 0.017) |
| **45** | 0.15 (0.050, 9.254) | 1.48 x 10^-6^ (5.546 x 10^-7^, 6.196 x 10^-5^) | 0.02 (0.015, 0.018) | 0.02 (0.017, 0.023) | 0.01 (0.008, 0.015) |
| **50** | 0.18 (0.074, 13.563) | 1.62 x 10^-6^ (6.632 x 10^-7^, 7.151 x 10^-5^) | 0.02 (0.015, 0.018) | 0.02 (0.018, 0.022) | 0.01 (0.008, 0.014) |
| **55** | 0.18 (0.069, 14.116) | 1.71 x 10^-6^ (6.954 x 10^-7^, 8.492 x 10^-5^) | 0.02 (0.015, 0.018) | 0.02 (0.018, 0.022) | 0.01 (0.008, 0.014) |
| **60** | 0.25 (0.096, 14.116) | 2.67 x 10^-6^ (8.563 x 10^-7^, 1.121 x 10^-5^) | 0.02 (0.015, 0.018) | 0.02 (0.018, 0.022) | 0.01 (0.009, 0.015) |
| **65** | 0.34 (0.146, 14.920) | 3.91 x 10^-6^ (1.236 x 10^-6^, 1.469 x 10^-4^) | 0.02 (0.015, 0.018) | 0.02 (0.018, 0.022) | 0.01 (0.010, 0.015) |
| **70** | 0.34 (0.134, 15.137) | 3.83 x 10^-6^ (1.189 x 10^-6^, 1.467 x 10^-4^) | 0.02 (0.015, 0.018) | 0.02 (0.018, 0.022) | 0.01 (0.010, 0.015) |
| **75** | 0.51 (0.149, 15.102) | 7.46 x 10^-6^ (1.959 x 10^-6^, 2.109 x 10^-4^) | 0.02 (0.015, 0.018) | 0.02 (0.018, 0.023) | 0.01 (0.010, 0.015) |
| **80** | 6.01 (0.218, 15.890) | 1.50 x 10^-4^ (4.046 x 10^-6^, 4.183 x 10^-4^) | 0.02 (0.016, 0.019) | 0.02 (0.021, 0.026) | 0.01 (0.010, 0.016) |
| **85** | 0.31 (0.115, 14.686) | 9.22 x 10^-6^ (2.971 x 10^-6^, 3.918 x 10^-4^) | 0.02 (0.019, 0.023) | 0.03 (0.025, 0.032) | 0.01 (0.009, 0.018) |
| **90** | 0.14 (0.050, 11.321) | 6.72 x 10^-6^ (2.834 x 10^-6^, 3.076 x 10^-4^) | 0.03 (0.025, 0.031) | 0.04 (0.030, 0.042) | 0.01 (0.006, 0.022) |
| **95** | 0.04 (2.061 x 10^-9^, 1.204) | 1.99 x 10^-6^ (9.620 x 10^-7^, 1.564 x 10^-4^) | 0.03 (0.031, 0.038) | 0.05 (0.032, 0.048) | 0.02 (0.015, 0.037) |
|  | **Fibular SMA_CC_** | | | | |
| **5** | 0.04 (2.061 x 10^-9^, 1.663) | 3.63 x 10^-6^ (1.746 x 10^-6^, 4.046 x 10^-5^) | 0.03 (0.024, 0.034) | 0.04 (0.023, 0.042) | 7.633 x 10^-3^ (0.006, 0.032) |
| **10** | 2.06 x 10^-9^ (2.061 x 10^-9^, 0.402) | 1.47 x 10^-6^ (8.313 x 10^-7^, 7.258 x 10^-6^) | 0.02 (0.017, 0.027) | 0.03 (0.012, 0.032) | 6.56 x 10^-3^ (0.009, 0.036) |
| **15** | 2.09 x 10^-9^ (2.061 x 10^-9^, 0.418) | 6.17 x 10^-7^ (3.630 x 10^-7^, 3.087 x 10^-6^) | 0.02 (0.015, 0.022) | 0.02 (0.011, 0.025) | 7.79 x 10^-3^ (0.009, 0.027) |
| **20** | 2.10 x 10^-9^ (2.061 x 10^-9^, 0.374) | 4.09 x 10^-7^ (2.376 x 10^-7^, 1.989 x 10^-6^) | 0.02 (0.016, 0.021) | 0.02 (0.012, 0.024) | 8.53 x 10^-3^ (0.011, 0.026) |
| **25** | 0.04 (2.061 x 10^-9^, 0.975) | 7.08 x 10^-7^ (3.290 x 10^-7^, 4.344 x 10^-6^) | 0.02 (0.016, 0.020) | 0.02 (0.013, 0.022) | 9.66 x 10^-3^ (0.007, 0.020) |
| **30** | 0.05 (2.061 x 10^-9^, 6.877) | 6.03 x 10^-7^ (2.844 x 10^-7^, 2.518 x 10^-5^) | 0.02 (0.016, 0.019) | 0.02 (0.015, 0.022) | 0.01 (0.008, 0.018) |
| **35** | 0.06 (2.683 x 10^-9^, 1.414) | 6.82 x 10^-7^ (3.090 x 10^-7^, 6.656 x 10^-6^) | 0.02 (0.016, 0.020) | 0.02 (0.016, 0.022) | 0.01 (0.008, 0.018) |
| **40** | 0.10 (0.018, 7.363) | 9.25 x 10^-7^ (3.904 x 10^-7^, 3.540 x 10^-5^) | 0.02 (0.016, 0.019) | 0.02 (0.017, 0.023) | 0.01 (0.008, 0.017) |
| **45** | 0.07 (2.214 x 10^-9^, 6.635) | 7.07 x 10^-7^ (3.200 x 10^-7^, 3.000 x 10^-5^) | 0.02 (0.016, 0.019) | 0.02 (0.017, 0.024) | 0.01 (0.008 0.018) |
| **50** | 0.10 (0.020, 8.170) | 8.38 x 10^-7^ (3.462 x 10^-7^, 3.677 x 10^-5^) | 0.02 (0.016, 0.019) | 0.02 (0.018, 0.024) | 0.01 (0.009, 0.017) |
| **55** | 0.12 (0.030, 7.180) | 8.93 x 10^-7^ (3.869 x 10^-7^, 3.354 x 10^-5^) | 0.02 (0.016, 0.019) | 0.02 (0.018, 0.024) | 0.01 (0.009, 0.017) |
| **60** | 0.16 (0.043, 8.976) | 1.14 x 10^-6^ (3.980 x 10^-7^, 3.854 x 10^-5^) | 0.02 (0.016, 0.019) | 0.02 (0.018, 0.023) | 0.01 (0.009, 0.016) |
| **65** | 0.17 (0.062, 13.487) | 1.26 x 10^-6^ (4.563 x 10^-7^, 6.056 x 10^-5^) | 0.02 (0.016, 0.018) | 0.02 (0.018, 0.023) | 0.01 (0.010, 0.016) |
| **70** | 0.22 (0.096, 14.108) | 1.58 x 10^-6^ (5.428 x 10^-7^, 7.519 x 10^-5^) | 0.02 (0.016, 0.018) | 0.02 (0.018, 0.023) | 0.01 (0.010, 0.015) |
| **75** | 0.67 (0.165, 14.911) | 5.41 x 10^-6^ (1.012 x 10^-6^, 1.079 x 10^-4^) | 0.02 (0.016, 0.018) | 0.02 (0.019, 0.023) | 0.01 (0.010, 0.014) |
| **80** | 0.32 (0.139, 14.877) | 3.41 x 10^-6^ (1.204 x 10^-6^, 1.243 x 10^-4^) | 0.02 (0.016, 0.018) | 0.02 (0.019, 0.024) | 0.01 (0.009, 0.015) |
| **85** | 0.15 (0.055, 10.779) | 3.47 x 10^-6^ (1.458 x 10^-6^, 1.536 x 10^-4^) | 0.02 (0.017, 0.022) | 0.03 (0.022, 0.030) | 0.01 (0.005, 0.018) |
| **90** | 2.66 (0.182, 15.378) | 1.25 x 10^-4^ (6.594 x 10^-6^, 6.703 x 10^-4^) | 0.03 (0.024, 0.029) | 0.03 (0.030, 0.038) | 0.02 (0.011, 0.020) |
| **95** | 0.40 (0.146, 14.442) | 1.85 x 10^-5^ (5.513 x 10^-6^, 5.315 x 10^-4^) | 0.04 (0.033, 0.039) | 0.05 (0.045, 0.054) | 0.02 (0.017, 0.029) |

**Table A2**. Parameter output for a multi-rate Brownian motion model with three means corresponding to fossorial+generalist, natatorial, and scansorial mustelids. σ^2^ _Fos+Gen_ , σ^2^ _Nat_, and σ^2^ _Sca_ denote rates of stochastic evolution for fossorial and generalist mustelids treated as a single group, natatorial mustelids, and scansorial mustelids, respectively. θ_0_ represents the starting state of trait values. Parameter estimates are followed by 95% confidence limits in parentheses, which were generated by parametric bootstrapping. Note that the bootstrapping routine does not provide confidence intervals for group means (not shown).

| **%** | **σ^2^ _Fos+Gen_** | **σ^2^ _Nat_** | **σ^2^ _Sca_** | **θ_0_** |
| --- | --- | --- | --- | --- |
|  | **Femoral CSA** | | | |
| **5** | 8.09 x 10^-6^ (2.986 x 10^-6^, 1.538 x 10^-5^) | 3.83 x 10^-5^ (1.142 x 10^-5^, 1.149 x 10^-4^) | 8.68 x 10^-6^ (2.074 x 10^-6^, 2.660 x 10^-5^) | 0.10 (0.085, 0.109) |
| **10** | 2.21 x 10^-5^ (8.891 x 10^-6^, 4.546 x 10^-5^) | 6.88 x 10^-5^ (1.981 x 10^-5^, 2.296 x 10^-4^) | 7.63 x 10^-6^ (2.062 x 10^-6^, 2.995 x 10^-5^) | 0.09 (0.068, 0.110) |
| **15** | 5.14 x 10^-6^ (1.765 x 10^-6^, 9.894 x 10^-6^) | 7.00 x 10^-5^ (1.655 x 10^-5^, 2.075 x 10^-4^) | 5.02 x 10^-6^ (1.737 x 10^-6^, 2.295 x 10^-5^) | 0.08 (0.065, 0.084) |
| **20** | 6.72 x 10^-6^ (2.806 x 10^-6^, 1.352 x 10^-5^) | 5.18 x 10^-5^ (1.526 x 10^-5^, 1.695 x 10^-4^) | 4.18 x 10^-6^ (1.205 x 10^-6^, 1.621 x 10^-5^) | 0.07 (0.059, 0.082) |
| **25** | 3.74 x 10^-6^ (1.651 x 10^-6^, 7.758 x 10^-6^) | 6.06 x 10^-5^ (1.677 x 10^-5^, 1.818 x 10^-4^) | 2.08 x 10^-6^ (7.643 x 10^-7^, 8.064 x 10^-6^) | 0.07 (0.057, 0.073) |
| **30** | 2.13 x 10^-6^ (8.316 x 10^-7^, 4.282 x 10^-6^) | 7.47 x 10^-5^ (2.005 x 10^-5^, 1.958 x 10^-4^) | 1.78 x 10^-6^ (4.632 x 10^-7^, 5.787 x 10^-6^) | 0.06 (0.056, 0.068) |
| **35** | 1.88 x 10^-6^ (7.396 x 10^-7^, 3.774 x 10^-6^) | 5.65 x 10^-5^ (1.306 x 10^-5^, 1.642 x 10^-4^) | 1.66 x 10^-6^ (4.824 x 10^-7^, 6.006 x 10^-6^) | 0.06 (0.056, 0.068) |
| **40** | 2.17 x 10^-6^ (8.169 x 10^-7^, 4.141 x 10^-6^) | 4.97 x 10^-5^ (1.379 x 10^-5^, 1.510 x 10^-4^) | 1.93 x 10^-6^ (4.829 x 10^-7^, 7.057 x 10^-6^) | 0.06 (0.056, 0.068) |
| **45** | 2.67 x 10^-6^ (9.938 x 10^-7^, 5.185 x 10^-6^) | 4.57 x 10^-5^ (1.269 x 10^-5^, 1.352 x 10^-4^) | 1.54 x 10^-6^ (4.316 x 10^-7^, 5.739 x 10^-6^) | 0.06 (0.054, 0.067) |
| **50** | 2.22 x 10^-6^ (8.324 x 10^-7^, 4.223 x 10^-6^) | 4.21 x 10^-5^ (1.208 x 10^-5^, 1.249 x 10^-4^) | 1.67 x 10^-6^ (4.864 x 10^-7^, 5.983 x 10^-6^) | 0.06 (0.053, 0.066) |
| **55** | 1.81 x 10^-6^ (5.878 x 10^-7^, 3.356 x 10^-6^) | 4.11 x 10^-5^ (1.124 x 10^-5^, 1.309 x 10^-4^) | 1.89 x 10^-6^ (5.998 x 10^-7^, 7.120 x 10^-6^) | 0.06 (0.053, 0.065) |
| **60** | 1.60 x 10^-6^ (6.015 x 10^-7^, 3.128 x 10^-6^) | 3.84 x 10^-5^ (1.103 x 10^-5^, 1.115 x 10^-4^) | 2.25 x 10^-6^ (7.080 x 10^-7^, 7.765 x 10^-6^) | 0.06 (0.054, 0.065) |
| **65** | 1.44 x 10^-6^ (5.652 x 10^-7^, 2.712 x 10^-6^) | 4.21 x 10^-5^ (1.031 x 10^-5^, 1.179 x 10^-4^) | 2.72 x 10^-6^ (9.205 x 10^-7^, 9.919 x 10^-6^) | 0.06 (0.055, 0.065) |
| **70** | 1.42 x 10^-6^ (5.116 x 10^-7^, 2.662 x 10^-6^) | 5.40 x 10^-5^ (1.224 x 10^-5^, 1.502 x 10^-4^) | 3.11 x 10^-6^ (9.173 x 10^-7^, 1.156 x 10^-5^) | 0.06 (0.056, 0.066) |
| **75** | 1.89 x 10^-6^ (6.862 x 10^-7^, 3.448 x 10^-6^) | 5.98 x 10^-5^ (1.428 x 10^-5^, 1.673 x 10^-4^) | 3.75 x 10^-6^ (1.181 x 10^-6^, 1.362 x 10^-5^) | 0.06 (0.056, 0.067) |
| **80** | 3.17 x 10^-6^ (1.255 x 10^-6^, 6.100 x 10^-6^) | 7.77 x 10^-5^ (1.861 x 10^-5^, 2.495 x 10^-4^) | 4.82 x 10^-6^ (1.382 x 10^-6^, 1.649 x 10^-5^) | 0.07 (0.059, 0.074) |
| **85** | 6.26 x 10^-6^ (2.529 x 10^-6^, 1.306 x 10^-5^) | 2.75 x 10^-4^ (6.217 x 10^-5^, 7.161 x 10^-4^) | 9.86 x 10^-6^ (2.750 x 10^-6^, 3.546 x 10^-5^) | 0.08 (0.066, 0.089) |
| **90** | 3.04 x 10^-5^ (1.322 x 10^-5^, 6.239 x 10^-5^) | 9.64 x 10^-5^ (2.461 x 10^-5^, 3.250 x 10^-4^) | 1.45 x 10^-5^ (4.072 x 10^-6^, 4.948 x 10^-5^) | 0.11 (0.083, 0.132) |
| **95** | 5.71 x 10^-6^ (2.085 x 10^-6^, 1.043 x 10^-5^) | 1.67 x 10^-5^ (6.480 x 10^-6^, 6.457 x 10^-5^) | 1.71 x 10^-5^ (4.856 x 10^-6^, 4.593 x 10^-5^) | 0.11 (0.094, 0.113) |
|  | **Femoral SMA_ML_** | | | |
| **5** | 3.68 x 10^-6^ (1.384 x 10^-6^, 6.909 x 10^-6^) | 5.85 x 10^-6^ (1.420 x 10^-6^, 1.648 x 10^-5^) | 2.76 x 10^-6^ (6.827 x 10^-7^, 8.834 x 10^-6^) | 0.06 (0.048, 0.064) |
| **10** | 1.15 x 10^-5^ (3.966 x 10^-6^, 2.294 x 10^-5^) | 1.71 x 10^-5^ (5.126 x 10^-6^, 4.861 x 10^-5^) | 3.56 x 10^-6^ (7.416 x 10^-7^, 1.110 x 10^-6^) | 0.05 (0.038, 0.065) |
| **15** | 1.68 x 10^-6^ (5.587 x 10^-7^, 2.963 x 10^-5^) | 1.14 x 10^-5^ (2.858 x 10^-6^, 3.502 x 10^-5^) | 2.29 x 10^-6^ (5.209 x 10^-7^, 6.514 x 10^-6^) | 0.04 (0.038, 0.048) |
| **20** | 3.06 x 10^-6^ (1.104 x 10^-6^, 5.832 x 10^-6^) | 1.01 x 10^-5^ (3.803 x 10^-6^, 3.948 x 10^-5^) | 1.60 x 10^-6^ (5.444 x 10^-7^, 5.864 x 10^-6^) | 0.04 (0.036, 0.051) |
| **25** | 2.40 x 10^-6^ (9.052 x 10^-7^, 4.418 x 10^-6^) | 1.47 x 10^-5^ (3.615 x 10^-6^, 4.538 x 10^-5^) | 1.17 x 10^-6^ (2.812 x 10^-7^, 3.993 x 10^-6^) | 0.04 (0.035, 0.048) |
| **30** | 1.90 x 10^-6^ (7.239 x 10^-7^, 3.561 x 10^-6^) | 1.26 x 10^-5^ (3.444 x 10^-6^, 3.807 x 10^-5^) | 8.81 x 10^-7^ (2.018 x 10^-7^, 2.727 x 10^-6^) | 0.04 (0.033, 0.044) |
| **35** | 1.59 x 10^-6^ (6.075 x 10^-7^, 2.818 x 10^-6^) | 9.79 x 10^-6^ (2.601 x 10^-6^, 3.097 x 10^-5^) | 6.78 x 10^-7^ (1.427 x 10^-7^, 1.892 x 10^-6^) | 0.04 (0.033, 0.042) |
| **40** | 1.22 x 10^-6^ (4.324 x 10^-7^, 2.270 x 10^-6^) | 1.00 x 10^-5^ (2.318 x 10^-6^, 3.141 x 10^-5^) | 6.24 x 10^-7^ (1.473 x 10^-7^, 1.789 x 10^-6^) | 0.04 (0.032, 0.041) |
| **45** | 1.10 x 10^-6^ (4.420 x 10^-7^, 1.963 x 10^-6^) | 1.14 x 10^-5^ (3.175 x 10^-6^, 3.215 x 10^-5^) | 5.85 x 10^-7^ (1.130 x 10^-7^, 1.564 x 10^-6^) | 0.04 (0.031, 0.040) |
| **50** | 6.97 x 10^-7^ (2.743 x 10^-7^, 1.368 x 10^-6^) | 1.31 x 10^-5^ (3.597 x 10^-6^, 4.011 x 10^-5^) | 6.91 x 10^-7^ (1.727 x 10^-7^, 2.021 x 10^-6^) | 0.04 (0.032, 0.040) |
| **55** | 5.35 x 10^-7^ (1.895 x 10^-7^, 1.060 x 10^-6^) | 1.61 x 10^-5^ (3.729 x 10^-6^, 4.610 x 10^-5^) | 9.07 x 10^-7^ (2.267 x 10^-7^, 2.541 x 10^-6^) | 0.04 (0.034, 0.040) |
| **60** | 5.34 x 10^-7^ (1.842 x 10^-7^, 1.028 x 10^-6^) | 1.72 x 10^-5^ (3.853 x 10^-6^, 4.427 x 10^-5^) | 1.24 x 10^-6^ (2.928 x 10^-7^, 3.861 x 10^-6^) | 0.04 (0.034, 0.040) |
| **65** | 6.10 x 10^-7^ (2.261 x 10^-7^, 1.147 x 10^-6^) | 1.83 x 10^-5^ (3.876 x 10^-6^, 4.813 x 10^-5^) | 1.63 x 10^-6^ (4.233 x 10^-7^, 4.998 x 10^-6^) | 0.04 (0.035, 0.041) |
| **70** | 9.14 x 10^-7^ (3.551 x 10^-7^, 1.752 x 10^-6^) | 2.20 x 10^-5^ (4.757 x 10^-6^, 5.780 x 10^-5^) | 1.62 x 10^-6^ (4.202 x 10^-7^, 5.069 x 10^-6^) | 0.04 (0.035, 0.043) |
| **75** | 1.27 x 10^-6^ (5.018 x 10^-7^, 2.436 x 10^-6^) | 2.41 x 10^-5^ (5.777 x 10^-6^, 6.632 x 10^-5^) | 1.77 x 10^-6^ (5.607 x 10^-7^, 5.558 x 10^-6^) | 0.04 (0.035, 0.045) |
| **80** | 2.51 x 10^-6^ (1.013 x 10^-6^, 4.835 x 10^-6^) | 3.26 x 10^-5^ (7.685 x 10^-6^, 9.306 x 10^-5^) | 2.26 x 10^-6^ (5.406 x 10^-7^, 6.780 x 10^-6^) | 0.05 (0.038, 0.051) |
| **85** | 5.42 x 10^-6^ (2.235 x 10^-6^, 1.039 x 10^-5^) | 1.17 x 10^-4^ (2.856 x 10^-5^, 2.985 x 10^-4^) | 2.87 x 10^-6^ (7.745 x 10^-7^, 9.208 x 10^-6^) | 0.05 (0.042, 0.062) |
| **90** | 6.52 x 10^-6^ (2.966 x 10^-6^, 1.463 x 10^-5^) | 3.34 x 10^-5^ (1.029 x 10^-5^, 1.183 x 10^-4^) | 5.90 x 10^-6^ (2.078 x 10^-6^, 2.614 x 10^-5^) | 0.08 (0.063, 0.085) |
| **95** | 9.14 x 10^-7^ (3.280 x 10^-7^, 1.744 x 10^-6^) | 7.83 x 10^-6^ (2.496 x 10^-6^, 3.210 x 10^-5^) | 2.63 x 10^-6^ (9.939 x 10^-7^, 9.952 x 10^-6^) | 0.07 (0.065, 0.074) |
|  | **Femoral SMA_CC_** | | | |
| **5** | 6.02 x 10^-6^ (2.583 x 10^-6^, 1.230 x 10^-5^) | 3.31 x 10^-5^ (8.816 x 10^-6^, 9.679 x 10^-5^) | 2.67 x 10^-6^ (6.867 x 10^-6^, 9.188 x 10^-5^) | 0.08 (0.071, 0.092) |
| **10** | 2.63 x 10^-6^ (1.094 x 10^-5^, 5.645 x 10^-5^) | 4.45 x 10^-5^ (1.487 x 10^-5^, 1.461 x 10^-4^) | 3.18 x 10^-6^ (9.565 x 10^-7^, 1.454 x 10^-5^) | 0.08 (0.057, 0.101) |
| **15** | 5.25 x 10^-6^ (2.150 x 10^-6^, 1.028 x 10^-5^) | 6.39 x 10^-5^ (1.569 x 10^-5^, 1.654 x 10^-4^) | 2.40 x 10^-6^ (9.091 x 10^-7^, 1.089 x 10^-5^) | 0.06 (0.049, 0.069) |
| **20** | 4.31 x 10^-6^ (1.704 x 10^-6^, 9.033 x 10^-6^) | 2.95 x 10^-5^ (6.696 x 10^-6^, 8.663 x 10^-5^) | 2.07 x 10^-6^ (6.617 x 10^-7^, 8.309 x 10^-6^) | 0.06 (0.045, 0.063) |
| **25** | 1.85 x 10^-6^ (7.551 x 10^-7^, 4.033 x 10^-6^) | 3.83 x 10^-5^ (7.833 x 10^-6^, 1.053 x 10^-4^) | 1.16 x 10^-6^ (4.002 x 10^-7^, 4.996 x 10^-6^) | 0.05 (0.041, 0.053) |
| **30** | 8.64 x 10^-7^ (3.970 x 10^-7^, 1.890 x 10^-6^) | 3.36 x 10^-5^ (7.441 x 10^-6^, 8.086 x 10^-5^) | 7.89 x 10^-7^ (2.952 x 10^-7^, 3.370 x 10^-6^) | 0.04 (0.039, 0.047) |
| **35** | 6.01 x 10^-7^ (2.731 x 10^-7^, 1.401 x 10^-6^) | 2.03 x 10^-5^ (4.827 x 10^-6^, 5.414 x 10^-5^) | 5.36 x 10^-7^ (2.096 x 10^-7^, 2.295 x 10^-6^) | 0.04 (0.036, 0.043) |
| **40** | 6.45 x 10^-7^ (2.685 x 10^-7^, 1.392 x 10^-6^) | 1.71 x 10^-5^ (4.102 x 10^-6^, 4.823 x 10^-5^) | 4.48 x 10^-7^ (1.168 x 10^-7^, 1.609 x 10^-6^) | 0.04 (0.035, 0.042) |
| **45** | 6.73 x 10^-7^ (2.756 x 10^-7^, 1.432 x 10^-6^) | 1.55 x 10^-5^ (4.076 x 10^-6^, 4.666 x 10^-5^) | 3.80 x 10^-7^ (8.966 x 10^-8^, 1.263 x 10^-6^) | 0.04 (0.034, 0.042) |
| **50** | 6.01 x 10^-7^ (2.487 x 10^-7^, 1.161 x 10^-6^) | 1.75 x 10^-5^ (4.174 x 10^-6^, 4.624 x 10^-5^) | 4.63 x 10^-7^ (1.271 x 10^-7^, 1.545 x 10^-6^) | 0.04 (0.035, 0.041) |
| **55** | 6.99 x 10^-7^ (2.435 x 10^-7^, 1.346 x 10^-6^) | 1.95 x 10^-5^ (4.487 x 10^-6^, 5.017 x 10^-5^) | 7.13 x 10^-7^ {2.263 x 10^-7^, 2.339 x 10^-6^) | 0.04 (0.035, 0.042) |
| **60** | 8.07 x 10^-7^ (2.987 x 10^-7^, 1.541 x 10^-6^) | 2.31 x 10^-5^ (5.179 x 10^-6^, 5.908 x 10^-5^) | 1.23 x 10^-6^ {3.003 x 10^-7^, 3.885 x 10^-6^) | 0.04 (0.036, 0.044) |
| **65** | 9.44 x 10^-7^ (3.141 x 10^-7^, 1.707 x 10^-6^) | 2.84 x 10^-5^ (6.376 x 10^-6^, 7.209 x 10^-5^) | 1.89 x 10^-6^ {5.210 x 10^-7^, 5.438 x 10^-6^) | 0.04 (0.037, 0.045) |
| **70** | 1.23 x 10^-6^ (4.547 x 10^-7^, 2.284 x 10^-6^) | 3.81 x 10^-5^ (8.541 x 10^-6^, 9.299 x 10^-5^) | 2.41 x 10^-6^ (6.101 x 10^-7^, 7.134 x 10^-6^) | 0.04 (0.038, 0.048) |
| **75** | 1.49 x 10^-6^ (5.413 x 10^-7^, 2.699 x 10^-6^) | 4.09 x 10^-5^ (1.007 x 10^-5^, 1.088 x 10^-4^) | 3.27 x 10^-6^ (9.541 x 10^-7^, 1.015 x 10^-5^) | 0.05 (0.040, 0.050) |
| **80** | 2.65 x 10^-6^ (9.073 x 10^-7^, 4.867 x 10^-6^) | 4.41 x 10^-5^ (1.181 x 10^-5^, 1.200 x 10^-4^) | 4.48 x 10^-6^ (1.216 x 10^-6^, 1.508 x 10^-5^) | 0.05 (0.044, 0.058) |
| **85** | 4.71 x 10^-6^ (1.821 x 10^-6^, 8.979 x 10^-6^) | 1.03 x 10^-4^ (2.793 x 10^-5^, 2.968 x 10^-4^) | 6.88 x 10^-6^ (1.983 x 10^-6^, 2.351 x 10^-5^) | 0.06 (0.051, 0.070) |
| **90** | 1.15 x 10^-5^ (4.813 x 10^-6^, 2.502 x 10^-5^) | 3.19 x 10^-5^ (1.075 x 10^-5^, 1.130x 10^-4^) | 6.51 x 10^-6^ (2.000 x 10^-6^, 2.742 x 10^-5^) | 0.08 (0.065, 0.096) |
| **95** | 1.85 x 10^-6^ (6.037 x 10^-7^, 3.365 x 10^-6^) | 1.21 x 10^-5^ (3.610 x 10^-6^, 3.991 x 10^-5^) | 4.75 x 10^-6^ (1.336 x 10^-6^, 1.486 x 10^-5^) | 0.08 (0.073, 0.084) |
|  | **Tibial CSA** | | | |
| **5** | 3.44 x 10^-5^ (1.238 x 10^-5^, 6.736 x 10^-5^) | 1.53 x 10^-5^ (2.957 x 10^-6^, 3.421 x 10^-5^) | 2.16 x 10^-5^ (5.793 x 10^-6^, 6.230 x 10^-5^) | 0.12 (0.093, 0.142) |
| **10** | 2.74 x 10^-5^ (1.093 x 10^-5^, 5.477 x 10^-5^) | 2.68 x 10^-5^ (6.341 x 10^-6^, 7.126 x 10^-5^) | 2.20 x 10^-5^ (7.495 x 10^-6^, 7.826 x 10^-5^) | 0.11 (0.082, 0.127) |
| **15** | 9.73 x 10^-6^ (3.689 x 10^-6^, 1.942 x 10^-5^) | 1.11 x 10^-5^ (2.496 x 10^-6^, 2.663 x 10^-5^) | 8.11 x 10^-6^ (2.820 x 10^-6^, 2.913 x 10^-5^) | 0.08 (0.068, 0.096) |
| **20** | 7.07 x 10^-6^ (2.343 x 10^-6^, 1.323 x 10^-5^) | 9.66 x 10^-6^ (2.180 x 10^-6^, 2.145 x 10^-5^) | 4.95 x 10^-6^ (1.352 x 10^-6^, 2.051 x 10^-5^) | 0.08 (0.063, 0.086) |
| **25** | 5.48 x 10^-6^ (1.908 x 10^-6^, 1.016 x 10^-5^) | 7.11 x 10^-6^ (1.478 x 10^-6^, 1.683 x 10^-5^) | 4.99 x 10^-6^ (1.591 x 10^-6^, 2.027 x 10^-5^) | 0.07 (0.061, 0.081) |
| **30** | 4.51 x 10^-6^ (1.684 x 10^-6^, 8.487 x 10^-6^) | 7.68 x 10^-6^ (1.805 x 10^-6^, 1.986 x 10^-5^) | 4.23 x 10^-6^ (1.421 x 10^-6^, 1.662 x 10^-5^) | 0.07 (0.061, 0.078) |
| **35** | 4.81 x 10^-6^ (1.758 x 10^-6^, 9.065 x 10^-6^) | 8.20 x 10^-6^ (2.061 x 10^-6^, 2.097 x 10^-5^) | 4.09 x 10^-6^ (1.343 x 10^-6^, 1.516 x 10^-5^) | 0.07 (0.059, 0.078) |
| **40** | 4.98 x 10^-6^ (1.791 x 10^-6^, 9.206 x 10^-6^) | 9.11 x 10^-6^ (2.038 x 10^-6^, 2.320 x 10^-5^) | 3.56 x 10^-6^ (1.147 x 10^-6^, 1.362 x 10^-5^) | 0.07 (0.058, 0.077) |
| **45** | 5.29 x 10^-6^ (2.059 x 10^-6^, 1.057 x 10^-5^) | 1.08 x 10^-5^ (2.691 x 10^-6^, 3.050 x 10^-5^) | 3.55 x 10^-6^ (1.130 x 10^-6^, 1.402 x 10^-5^) | 0.07 (0.057, 0.076) |
| **50** | 5.28 x 10^-6^ (1.981 x 10^-6^, 1.032 x 10^-5^) | 1.28 x 10^-5^ (2.799 x 10^-6^, 3.496 x 10^-5^) | 3.35 x 10^-6^ (1.130 x 10^-6^, 1.402 x 10^-5^) | 0.07 (0.056, 0.077) |
| **55** | 5.52 x 10^-6^ (1.956 x 10^-6^, 1.001 x 10^-5^) | 1.41 x 10^-5^ (3.842 x 10^-6^, 3.733 x 10^-5^) | 3.06 x 10^-6^ (1.016 x 10^-6^, 1.168 x 10^-5^) | 0.07 (0.056, 0.075) |
| **60** | 4.96 x 10^-6^ (1.897 x 10^-6^, 8.976 x 10^-6^) | 1.43 x 10^-5^ (2.599 x 10^-6^, 3.959 x 10^-5^) | 3.72 x 10^-6^ (1.144 x 10^-6^, 1.355 x 10^-5^) | 0.06 (0.054, 0.073) |
| **65** | 4.11 x 10^-6^ (1.487 x 10^-6^, 7.638 x 10^-6^) | 1.10 x 10^-5^ (2.648 x 10^-6^, 2.978 x 10^-5^) | 4.18 x 10^-6^ (1.134 x 10^-6^, 1.606 x 10^-5^) | 0.06 (0.054, 0.070) |
| **70** | 3.67 x 10^-6^ (1.443 x 10^-6^, 6.704 x 10^-6^) | 7.92 x 10^-6^ (1.802 x 10^-6^, 2.303 x 10^-5^) | 4.11 x 10^-6^ (1.338 x 10^-6^, 1.636 x 10^-5^) | 0.06 (0.054, 0.070) |
| **75** | 3.87 x 10^-6^ (1.395 x 10^-6^, 6.966 x 10^-6^) | 7.79 x 10^-6^ (2.094 x 10^-6^, 2.436 x 10^-5^) | 5.54 x 10^-6^ (1.794 x 10^-6^, 2.092 x 10^-5^) | 0.06 (0.055, 0.071) |
| **80** | 4.02 x 10^-6^ (1.486 x 10^-6^, 7.867 x 10^-6^) | 9.84 x 10^-6^ (2.878 x 10^-6^, 3.307 x 10^-5^) | 6.64 x 10^-6^ (2.354 x 10^-6^, 2.565 x 10^-5^) | 0.06 (0.056, 0.072) |
| **85** | 6.23 x 10^-6^ (2.260 x 10^-6^, 1.130 x 10^-5^) | 2.00 x 10^-5^ (5.992 x 10^-6^, 6.122 x 10^-5^) | 7.63 x 10^-6^ (2.979 x 10^-6^, 3.523 x 10^-5^) | 0.07 (0.059, 0.081) |
| **90** | 1.36 x 10^-5^ (4.890 x 10^-6^, 2.487 x 10^-5^) | 2.30 x 10^-5^ (7.790 x 10^-6^, 7.784 x 10^-5^) | 1.62 x 10^-5^ (4.880 x 10^-6^, 6.615 x 10^-5^) | 0.08 (0.068, 0.099) |
| **95** | 4.36 x 10^-5^ (1.441 x 10^-5^, 7.239 x 10^-5^) | 9.07 x 10^-6^ (1.734 x 10^-6^, 2.090 x 10^-5^) | 2.02 x 10^-5^ (3.003 x 10^-6^, 5.115 x 10^-5^) | 0.07 (0.043, 0.097) |
|  | **Tibial SMA_ML_** | | | |
| **5** | 3.13 x 10^-6^ (1.103 x 10^-6^, 5.825 x 10^-6^) | 3.17 x 10^-6^ (7.207 x 10^-7^, 8.113 x 10^-6^) | 4.95 x 10^-6^ (1.931 x 10^-6^, 1.841 x 10^-5^) | 0.08 (0.070, 0.084) |
| **10** | 1.69 x 10^-5^ (6.205 x 10^-6^, 3.154 x 10^-5^) | 1.02 x 10^-5^ (1.859 x 10^-6^, 2.175 x 10^-5^) | 9.46 x 10^-6^ (2.522 x 10^-6^, 3.500 x 10^-5^) | 0.08 (0.059, 0.094) |
| **15** | 7.29 x 10^-6^ (2.721 x 10^-6^, 1.423 x 10^-5^) | 4.69 x 10^-6^ (8.004 x 10^-7^, 1.025 x 10^-5^) | 3.64 x 10^-6^ (1.081 x 10^-6^, 1.378 x 10^-5^) | 0.06 (0.052, 0.074) |
| **20** | 4.79 x 10^-6^ (1.860 x 10^-6^, 9.714 x 10^-6^) | 4.19 x 10^-6^ (7.675 x 10^-7^, 9.093 x 10^-6^) | 2.44 x 10^-6^ (7.298 x 10^-7^, 9.342 x 10^-6^) | 0.06 (0.047, 0.065) |
| **25** | 3.42 x 10^-6^ (1.244 x 10^-6^, 6.218 x 10^-6^) | 4.02 x 10^-6^ (7.005 x 10^-7^, 8.473 x 10^-6^) | 2.20 x 10^-6^ (7.515 x 10^-7^, 8.648 x 10^-6^) | 0.05 (0.045, 0.060) |
| **30** | 2.66 x 10^-6^ (9.803 x 10^-7^, 4.970 x 10^-6^) | 3.37 x 10^-6^ (6.221 x 10^-7^, 7.311 x 10^-6^) | 1.88 x 10^-6^ (5.780 x 10^-7^, 7.200 x 10^-6^) | 0.05 (0.043, 0.057) |
| **35** | 2.89 x 10^-6^ (9.851 x 10^-7^, 5.434 x 10^-6^) | 2.53 x 10^-6^ (5.884 x 10^-7^, 5.749 x 10^-6^) | 1.47 x 10^-6^ (4.905 x 10^-7^, 5.777 x 10^-6^) | 0.05 (0.041, 0.055) |
| **40** | 3.09 x 10^-6^ (1.179 x 10^-6^, 5.777 x 10^-6^) | 2.23 x 10^-6^ (5.171 x 10^-7^, 4.848 x 10^-6^) | 1.28 x 10^-6^ (3.598 x 10^-7^, 5.113 x 10^-6^) | 0.05 (0.039, 0.054) |
| **45** | 3.10 x 10^-6^ (1.211 x 10^-6^, 6.102 x 10^-6^) | 2.10 x 10^-6^ (4.154 x 10^-7^, 5.067 x 10^-6^) | 1.21 x 10^-6^ (2.931 x 10^-7^, 4.713 x 10^-6^) | 0.04 (0.037, 0.052) |
| **50** | 2.89 x 10^-6^ (1.144 x 10^-6^, 5.547 x 10^-6^) | 2.70 x 10^-6^ (5.642 x 10^-7^, 6.544 x 10^-6^) | 1.19 x 10^-6^ (3.372 x 10^-7^, 4.294 x 10^-6^) | 0.04 (0.035, 0.050) |
| **55** | 2.68 x 10^-6^ (9.829 x 10^-7^, 5.100 x 10^-6^) | 3.47 x 10^-6^ (7.794 x 10^-7^, 8.155 x 10^-6^) | 1.09 x 10^-6^ (2.548 x 10^-7^, 3.828 x 10^-6^) | 0.04 (0.034, 0.047) |
| **60** | 2.30 x 10^-6^ (8.442 x 10^-7^, 4.410 x 10^-6^) | 3.70 x 10^-6^ (8.799 x 10^-7^, 8.573 x 10^-6^) | 1.26 x 10^-6^ (3.697 x 10^-7^, 4.571 x 10^-6^) | 0.04 (0.033, 0.045) |
| **65** | 1.92 x 10^-6^ (6.641 x 10^-7^, 3.466 x 10^-6^) | 3.35 x 10^-6^ (7.331 x 10^-7^, 7.999 x 10^-6^) | 1.51 x 10^-6^ (4.636 x 10^-7^, 5.344 x 10^-6^) | 0.04 (0.033, 0.044) |
| **70** | 1.57 x 10^-6^ (6.383 x 10^-7^, 2.866 x 10^-6^) | 2.88 x 10^-6^ (5.850 x 10^-7^, 6.972 x 10^-6^) | 1.86 x 10^-6^ (4.762 x 10^-7^, 6.165 x 10^-6^) | 0.04 (0.033, 0.043) |
| **75** | 1.51 x 10^-6^ (5.660 x 10^-7^, 2.570 x 10^-6^) | 2.89 x 10^-6^ (5.966 x 10^-7^, 7.169 x 10^-6^) | 2.73 x 10^-6^ (6.157 x 10^-7^, 8.229 x 10^-6^) | 0.04 (0.033, 0.044) |
| **80** | 1.63 x 10^-6^ (5.528 x 10^-7^, 2.968 x 10^-6^) | 3.83 x 10^-6^ (9.407 x 10^-7^, 1.004 x 10^-5^) | 3.47 x 10^-6^ (8.920 x 10^-7^, 1.184 x 10^-5^) | 0.04 (0.035, 0.045) |
| **85** | 2.44 x 10^-6^ (8.356 x 10^-7^, 4.624 x 10^-6^) | 8.66 x 10^-6^ (2.203 x 10^-6^, 2.399 x 10^-5^) | 4.50 x 10^-6^ (1.331 x 10^-6^, 1.478 x 10^-5^) | 0.05 (0.038, 0.051) |
| **90** | 4.41 x 10^-6^ (1.643 x 10^-6^, 8.409 x 10^-6^) | 8.67 x 10^-6^ (2.753 x 10^-6^, 2.946 x 10^-5^) | 6.27 x 10^-6^ (1.728 x 10^-6^, 2.319 x 10^-5^) | 0.05 (0.046, 0.063) |
| **95** | 7.72 x 10^-6^ (2.579 x 10^-6^, 1.250 x 10^-5^) | 3.99 x 10^-6^ (6.240 x 10^-7^, 8.441 x 10^-6^) | 4.33 x 10^-6^ (1.028 x 10^-6^, 1.165 x 10^-5^) | 0.05 (0.038, 0.060) |
|  | **Tibial SMA_CC_** | | | |
| **5** | 7.73 x 10^-6^ (2.831 x 10^-6^, 1.511 x 10^-5^) | 8.99 x 10^-6^ (2.093 x 10^-6^, 2.311 x 10^-5^) | 6.97 x 10^-6^ (2.117 x 10^-6^, 2.861 x 10^-5^) | 0.09 (0.076, 0.099) |
| **10** | 1.89 x 10^-5^ (7.623 x 10^-6^, 3.893 x 10^-5^) | 1.15 x 10^-5^ (2.216 x 10^-6^, 2.636 x 10^-5^) | 8.64 x 10^-6^ (2.691 x 10^-6^, 3.307 x 10^-5^) | 0.08 (0.059, 0.095) |
| **15** | 6.19 x 10^-6^ (2.435 x 10^-6^, 1.173 x 10^-5^) | 5.01 x 10^-6^ (1.043 x 10^-6^, 1.137 x 10^-5^) | 3.82 x 10^-6^ (9.491 x 10^-7^, 1.371 x 10^-5^) | 0.06 (0.045, 0.066) |
| **20** | 3.17 x 10^-6^ (1.051 x 10^-6^, 6.220 x 10^-6^) | 4.89 x 10^-6^ (9.906 x 10^-7^, 1.152 x 10^-5^) | 2.78 x 10^-6^ (6.807 x 10^-7^, 9.929 x 10^-6^) | 0.05 (0.038, 0.053) |
| **25** | 2.20 x 10^-6^ (7.667 x 10^-7^, 4.143 x 10^-6^) | 4.40 x 10^-6^ (8.415 x 10^-7^, 1.028 x 10^-5^) | 2.24 x 10^-6^ (6.869 x 10^-7^, 7.892 x 10^-6^) | 0.04 (0.037, 0.048) |
| **30** | 1.56 x 10^-6^ (5.997 x 10^-7^, 2.712 x 10^-6^) | 4.23 x 10^-6^ (8.249 x 10^-7^, 9.362 x 10^-6^) | 1.70 x 10^-6^ (4.840 x 10^-7^, 5.134 x 10^-6^) | 0.04 (0.035, 0.045) |
| **35** | 1.15 x 10^-6^ (3.747 x 10^-7^, 2.085 x 10^-6^) | 4.13 x 10^-6^ (9.124 x 10^-7^, 1.004 x 10^-5^) | 1.25 x 10^-6^ (3.338 x 10^-7^, 4.015 x 10^-6^) | 0.04 (0.034, 0.042) |
| **40** | 9.07 x 10^-7^ (3.121 x 10^-7^, 1.661 x 10^-6^) | 4.85 x 10^-6^ (1.114 x 10^-6^, 1.188 x 10^-5^) | 9.50 x 10^-7^ (2.613 x 10^-7^, 3.125 x 10^-6^) | 0.04 (0.033, 0.040) |
| **45** | 8.60 x 10^-7^ (3.199 x 10^-7^, 1.646 x 10^-6^) | 5.18 x 10^-6^ (1.062 x 10^-6^, 1.234 x 10^-5^) | 8.76 x 10^-7^ (2.565 x 10^-7^, 2.978 x 10^-6^) | 0.04 (0.032, 0.040) |
| **50** | 9.12 x 10^-7^ (3.353 x 10^-7^, 1.726 x 10^-6^) | 5.44 x 10^-6^ (1.388 x 10^-6^, 1.383 x 10^-5^) | 8.58 x 10^-7^ (2.786 x 10^-7^, 2.879 x 10^-6^) | 0.04 (0.032, 0.040) |
| **55** | 1.05 x 10^-6^ (3.800 x 10^-7^, 2.002 x 10^-6^) | 5.94 x 10^-6^ (1.271 x 10^-6^, 1.568 x 10^-5^) | 8.89 x 10^-7^ (2.317 x 10^-7^, 3.043 x 10^-6^) | 0.04 (0.032, 0.040) |
| **60** | 1.11 x 10^-6^ (3.907 x 10^-7^, 2.173 x 10^-6^) | 5.80 x 10^-6^ (1.397 x 10^-6^, 1.514 x 10^-5^) | 1.08 x 10^-6^ (3.011 x 10^-7^, 3.744 x 10^-6^) | 0.04 (0.032, 0.041) |
| **65** | 1.28 x 10^-6^ (4.579 x 10^-7^, 2.426 x 10^-6^) | 5.40 x 10^-6^ (1.347 x 10^-6^, 1.480 x 10^-5^) | 1.41 x 10^-6^ (3.903 x 10^-7^, 5.317 x 10^-6^) | 0.04 (0.033, 0.042) |
| **70** | 1.49 x 10^-6^ (5.468 x 10^-7^, 2.700 x 10^-6^) | 4.47 x 10^-6^ (1.075 x 10^-6^, 1.211 x 10^-5^) | 1.83 x 10^-6^ (6.051 x 10^-7^, 6.637 x 10^-6^) | 0.04 (0.034, 0.044) |
| **75** | 1.90 x 10^-6^ (7.155 x 10^-7^, 3.484 x 10^-6^) | 5.07 x 10^-6^ (1.193 x 10^-6^, 1.287 x 10^-5^) | 2.58 x 10^-6^ (7.883 x 10^-7^, 8.822 x 10^-6^) | 0.04 (0.035, 0.046) |
| **80** | 2.27 x 10^-6^ (8.361 x 10^-7^, 4.221 x 10^-6^) | 7.55 x 10^-6^ (1.746 x 10^-6^, 1.936 x 10^-5^) | 3.41 x 10^-6^ (1.018 x 10^-6^, 1.300 x 10^-5^) | 0.04 (0.037, 0.049) |
| **85** | 3.79 x 10^-6^ (1.461 x 10^-6^, 7.435 x 10^-6^) | 1.25 x 10^-5^ (2.934 x 10^-6^, 3.328 x 10^-5^) | 4.29 x 10^-6^ (1.785 x 10^-6^, 1.715 x 10^-5^) | 0.05 (0.041, 0.058) |
| **90** | 6.36 x 10^-6^ (2.387 x 10^-6^, 1.206 x 10^-5^) | 6.04 x 10^-6^ (1.291 x 10^-6^, 1.609 x 10^-5^) | 5.93 x 10^-6^  (2.216 x 10^-6^, 2.785 x 10^-5^) | 0.06 (0.050, 0.071) |
| **95** | 1.53 x 10^-5^ (4.789 x 10^-6^, 2.434 x 10^-5^) | 1.89 x 10^-6^ (3.833 x 10^-7^, 4.787 x 10^-6^) | 4.74 x 10^-6^ (9.248 x 10^-7^, 1.157 x 10^-5^) | 0.05 (0.031, 0.062) |
|  | **Fibular CSA** | | | |
| **5** | 7.61 x 10^-6^ (3.728 x 10^-6^, 1.862 x 10^-5^) | 2.25 x 10^-6^ (6.394 x 10^-7^, 7.991 x 10^-6^) | 2.26 x 10^-6^ (5.335 x 10^-7^, 7.445 x 10^-6^) | 0.05 (0.035, 0.060) |
| **10** | 2.44 x 10^-6^ (1.254 x 10^-6^, 6.377 x 10^-6^) | 3.40 x 10^-6^ (7.125 x 10^-7^, 8.822 x 10^-6^) | 9.00 x 10^-7^ (2.852 x 10^-7^, 3.534 x 10^-6^) | 0.04 (0.028, 0.043) |
| **15** | 1.75 x 10^-6^ (8.280 x 10^-7^, 4.128 x 10^-6^) | 1.30 x 10^-6^ (2.463 x 10^-7^, 3.047 x 10^-6^) | 6.04 x 10^-7^ (1.620 x 10^-7^, 2.066 x 10^-6^) | 0.03 (0.025, 0.037) |
| **20** | 1.47 x 10^-6^ (6.407 x 10^-7^, 3.292 x 10^-6^) | 6.83 x 10^-7^ (1.427 x 10^-7^, 1.546 x 10^-6^) | 6.16 x 10^-7^ (1.600 x 10^-7^, 2.247 x 10^-6^) | 0.03 (0.025, 0.036) |
| **25** | 1.51 x 10^-6^ (7.184 x 10^-7^, 3.387 x 10^-6^) | 3.43 x 10^-7^ (6.622 x 10^-8^, 7.918 x 10^-7^) | 6.79 x 10^-7^ (1.523 x 10^-7^, 2.188 x 10^-6^) | 0.03 (0.026, 0.037) |
| **30** | 1.33 x 10^-6^ (5.794 x 10^-7^, 2.993 x 10^-6^) | 7.34 x 10^-7^ (1.490 x 10^-7^, 1.645 x 10^-6^) | 5.13 x 10^-7^ (1.185 x 10^-7^, 1.744 x 10^-6^) | 0.03 (0.026, 0.037) |
| **35** | 1.24 x 10^-6^ (5.324 x 10^-7^, 2.830 x 10^-6^) | 1.43 x 10^-6^ (2.645 x 10^-7^, 3.430 x 10^-6^) | 5.20 x 10^-7^ (1.126 x 10^-7^, 1.827 x 10^-6^) | 0.03 (0.027, 0.037) |
| **40** | 1.31 x 10^-6^ (5.458 x 10^-7^, 2.784 x 10^-6^) | 1.57 x 10^-6^ (3.749 x 10^-7^, 3.982 x 10^-6^) | 5.88 x 10^-7^ (1.258 x 10^-7^, 2.066 x 10^-6^) | 0.03 (0.027, 0.036) |
| **45** | 1.09 x 10^-6^ (4.846 x 10^-7^, 2.339 x 10^-6^) | 1.55 x 10^-6^ (4.766 x 10^-7^, 4.513 x 10^-6^) | 5.59 x 10^-7^ (1.566 x 10^-7^, 1.991 x 10^-6^) | 0.03 (0.027, 0.036) |
| **50** | 9.36 x 10^-7^ (4.013 x 10^-7^, 2.037 x 10^-6^) | 1.47 x 10^-6^ (3.823 x 10^-7^, 4.423 x 10^-6^) | 6.60 x 10^-7^ (2.016 x 10^-7^, 2.566 x 10^-6^) | 0.03 (0.028, 0.036) |
| **55** | 8.60 x 10^-7^ (3.610 x 10^-7^, 1.972 x 10^-6^) | 1.72 x 10^-6^ (4.226 x 10^-7^, 5.016 x 10^-6^) | 5.63 x 10^-7^ (1.953 x 10^-7^, 2.398 x 10^-6^) | 0.03 (0.028, 0.036) |
| **60** | 8.11 x 10^-7^ (3.501 x 10^-7^, 1.726 x 10^-6^) | 2.14 x 10^-6^ (5.396 x 10^-7^, 6.035 x 10^-6^) | 5.62 x 10^-7^ (1.763 x 10^-7^, 2.090 x 10^-6^) | 0.03 (0.028, 0.036) |
| **65** | 1.00 x 10^-6^ (3.764 x 10^-7^, 2.121 x 10^-6^) | 3.02 x 10^-6^ (6.428 x 10^-7^, 7.526 x 10^-6^) | 6.47 x 10^-7^ (1.851 x 10^-7^, 2.204 x 10^-6^) | 0.03 (0.028, 0.036) |
| **70** | 1.05 x 10^-6^ (4.189 x 10^-7^, 2.121 x 10^-6^) | 3.49 x 10^-6^ (8.224 x 10^-7^, 9.069 x 10^-6^) | 7.67 x 10^-7^ (2.065 x 10^-7^, 2.425 x 10^-6^) | 0.03 (0.027, 0.036) |
| **75** | 1.07 x 10^-6^ (4.350 x 10^-7^, 2.050 x 10^-6^) | 5.74 x 10^-6^ (1.080 x 10^-6^, 1.533 x 10^-5^) | 1.01 x 10^-6^ (2.267 x 10^-7^, 3.144 x 10^-6^) | 0.03 (0.026, 0.036) |
| **80** | 9.00 x 10^-7^ (3.837 x 10^-7^, 1.943 x 10^-6^) | 1.07 x 10^-5^ (2.268 x 10^-6^, 2.433 x 10^-5^) | 8.61 x 10^-7^ (2.947 x 10^-7^, 3.136 x 10^-6^) | 0.03 (0.027, 0.036) |
| **85** | 7.10 x 10^-7^ (2.989 x 10^-7^, 1.480 x 10^-6^) | 1.32 x 10^-5^ (2.768 x 10^-6^, 3.088 x 10^-5^) | 8.68 x 10^-7^ (4.975 x 10^-7^, 5.675 x 10^-6^) | 0.04 (0.031, 0.039) |
| **90** | 3.58 x 10^-6^ (1.573 x 10^-6^, 8.499 x 10^-6^) | 1.87 x 10^-5^ (4.679 x 10^-6^, 4.575 x 10^-5^) | 1.65 x 10^-6^ (5.795 x 10^-7^, 8.282 x 10^-6^) | 0.04 (0.035, 0.052) |
| **95** | 3.67 x 10^-6^ (1.587 x 10^-6^, 8.049 x 10^-6^) | 1.25 x 10^-5^ (3.673 x 10^-6^, 4.112 x 10^-5^) | 2.53 x 10^-6^ (8.996 x 10^-7^, 1.114 x 10^-5^) | 0.06 (0.046, 0.063) |
|  | **Fibular SMA_ML_** | | | |
| **5** | 5.45 x 10^-6^ (2.468 x 10^-6^, 1.317 x 10^-5^) | 2.25 x 10^-6^ (4.601 x 10^-7^, 5.521 x 10^-6^) | 1.83 x 10^-6^ (4.957 x 10^-7^, 7.124 x 10^-6^) | 0.04 (0.026, 0.048) |
| **10** | 2.67 x 10^-6^ (1.212 x 10^-6^, 5.803 x 10^-6^) | 1.06 x 10^-5^ (2.324 x 10^-6^, 2.625 x 10^-5^) | 7.07 x 10^-7^ (1.522 x 10^-7^, 2.499 x 10^-6^) | 0.02 (0.017, 0.032) |
| **15** | 9.75 x 10^-7^ (3.855 x 10^-7^, 2.137 x 10^-6^) | 1.51 x 10^-6^ (3.138 x 10^-7^, 3.706 x 10^-6^) | 5.06 x 10^-7^ (1.183 x 10^-7^, 1.455 x 10^-6^) | 0.02 (0.014, 0.022) |
| **20** | 5.74 x 10^-7^ (2.462 x 10^-7^, 1.283 x 10^-6^) | 1.09 x 10^-6^ (2.525 x 10^-7^, 2.811 x 10^-6^) | 3.30 x 10^-7^ (9.051 x 10^-8^, 1.038 x 10^-6^) | 0.02 (0.013, 0.020) |
| **25** | 5.99 x 10^-7^ (2.438 x 10^-7^, 1.225 x 10^-6^) | 8.87 x 10^-7^ (1.941 x 10^-7^, 2.313 x 10^-6^) | 3.01 x 10^-7^ (6.769 x 10^-8^, 8.527 x 10^-7^) | 0.02 (0.013, 0.020) |
| **30** | 4.83 x 10^-7^ (2.138 x 10^-7^, 1.059 x 10^-6^) | 1.13 x 10^-6^ (2.825 x 10^-7^, 3.152 x 10^-6^) | 1.66 x 10^-7^ (3.406 x 10^-8^, 4.948 x 10^-7^) | 0.02 (0.014, 0.020) |
| **35** | 4.46 x 10^-7^ (1.937 x 10^-7^, 9.457 x 10^-7^) | 1.31 x 10^-6^ (3.284 x 10^-7^, 3.371 x 10^-6^) | 1.55 x 10^-7^ (3.071 x 10^-8^, 4.983 x 10^-7^) | 0.02 (0.014, 0.020) |
| **40** | 5.01 x 10^-7^ (2.016 x 10^-7^, 1.095 x 10^-6^) | 1.26 x 10^-6^ (2.773 x 10^-7^, 3.009 x 10^-6^) | 1.90 x 10^-7^ (4.018 x 10^-8^, 6.219 x 10^-7^) | 0.02 (0.013, 0.020) |
| **45** | 4.40 x 10^-7^ (1.939 x 10^-7^, 9.259 x 10^-7^) | 1.16 x 10^-6^ (2.735 x 10^-7^, 3.024 x 10^-6^) | 2.15 x 10^-7^ (4.600 x 10^-8^, 6.054 x 10^-7^) | 0.02 (0.014, 0.020) |
| **50** | 4.09 x 10^-7^ (1.733 x 10^-7^, 8.810 x 10^-7^) | 1.14 x 10^-6^ (2.457 x 10^-7^, 2.897 x 10^-6^) | 2.64 x 10^-7^ (6.395 x 10^-8^, 8.855 x 10^-7^) | 0.02 (0.014, 0.020) |
| **55** | 3.26 x 10^-7^ (1.418 x 10^-7^, 6.756 x 10^-7^) | 1.60 x 10^-6^ (3.634 x 10^-7^, 3.925 x 10^-6^) | 2.57 x 10^-7^ (6.881 x 10^-8^, 9.323 x 10^-7^) | 0.02 (0.015, 0.020) |
| **60** | 3.14 x 10^-7^ (1.209 x 10^-7^, 6.574 x 10^-7^) | 2.27 x 10^-6^ (4.558 x 10^-7^, 5.232 x 10^-6^) | 3.01 x 10^-7^ (6.929 x 10^-8^, 9.399 x 10^-7^) | 0.02 (0.015, 0.020) |
| **65** | 3.23 x 10^-7^ (1.221 x 10^-7^, 6.016 x 10^-7^) | 2.88 x 10^-6^ (6.293 x 10^-7^, 6.250 x 10^-6^) | 4.29 x 10^-7^ (9.128 x 10^-8^, 1.138 x 10^-6^) | 0.02 (0.015, 0.020) |
| **70** | 2.88 x 10^-7^ (1.126 x 10^-7^, 5.481 x 10^-7^) | 2.76 x 10^-6^ (5.326 x 10^-7^, 6.075 x 10^-6^) | 5.25 x 10^-7^ (1.197 x 10^-8^, 1.426 x 10^-6^) | 0.02 (0.015, 0.020) |
| **75** | 3.01 x 10^-7^ (1.050 x 10^-7^, 5.646 x 10^-7^) | 4.02 x 10^-6^ (7.783 x 10^-7^, 8.319 x 10^-6^) | 8.78 x 10^-7^ (2.030 x 10^-8^, 2.245 x 10^-6^) | 0.02 (0.015, 0.020) |
| **80** | 4.50 x 10^-7^ (1.615 x 10^-7^, 8.438 x 10^-7^) | 9.65 x 10^-6^ (1.842 x 10^-6^, 2.011 x 10^-5^) | 7.62 x 10^-7^ (2.0210 x 10^-7^, 2.237 x 10^-6^) | 0.02 (0.015, 0.021) |
| **85** | 3.59 x 10^-7^ (1.419 x 10^-7^, 6.734 x 10^-7^) | 8.43 x 10^-6^ (1.842 x 10^-6^, 2.062 x 10^-5^) | 6.99 x 10^-7^ (3.165 x 10^-7^, 3.527 x 10^-6^) | 0.02 (0.019, 0.024) |
| **90** | 1.31 x 10^-6^ (6.171 x 10^-7^, 3.395 x 10^-6^) | 7.46 x 10^-6^ (1.541 x 10^-6^, 1.836 x 10^-5^) | 9.12 x 10^-7^ (3.749 x 10^-7^, 4.674 x 10^-6^) | 0.03 (0.023, 0.033) |
| **95** | 1.17 x 10^-6^ (4.714 x 10^-7^, 2.566 x 10^-6^) | 1.98 x 10^-6^ (4.542 x 10^-7^, 5.958 x 10^-6^) | 1.62 x 10^-6^ (6.025 x 10^-7^, 6.752 x 10^-6^) | 0.04 (0.027, 0.040) |
|  | **Fibular SMA_CC_** | | | |
| **5** | 3.15 x 10^-6^ (1.420 x 10^-6^, 7.241 x 10^-6^) | 3.29 x 10^-6^ (6.474 x 10^-7^, 7.849 x 10^-6^) | 8.85 x 10^-7^ (2.004 x 10^-7^, 3.440 x 10^-6^) | 0.03 (0.021, 0.038) |
| **10** | 1.91 x 10^-6^ (8.721 x 10^-7^, 7.241 x 10^-6^) | 1.78 x 10^-6^ (3.526 x 10^-7^, 4.129 x 10^-6^) | 2.39 x 10^-7^ (6.251 x 10^-8^, 9.491 x 10^-7^) | 0.02 (0.016, 0.028) |
| **15** | 8.02 x 10^-7^ (3.430 x 10^-7^, 1.776 x 10^-6^) | 7.36 x 10^-7^ (1.449 x 10^-7^, 1.749 x 10^-6^) | 1.19 x 10^-7^ (3.382 x 10^-8^, 5.668 x 10^-7^) | 0.02 (0.014, 0.022) |
| **20** | 6.07 x 10^-7^ (2.517 x 10^-7^, 1.215 x 10^-6^) | 2.26 x 10^-7^ (4.377 x 10^-8^, 4.657 x 10^-7^) | 1.48 x 10^-7^ (4.119 x 10^-8^, 6.418 x 10^-7^) | 0.02 (0.014, 0.021) |
| **25** | 6.80 x 10^-7^ (2.528 x 10^-7^, 1.397 x 10^-6^) | 4.21 x 10^-7^ (7.581 x 10^-8^, 9.344 x 10^-7^) | 2.25 x 10^-7^ (4.552 x 10^-8^, 8.045 x 10^-7^) | 0.02 (0.014, 0.021) |
| **30** | 3.70 x 10^-7^ (1.538 x 10^-7^, 7.873 x 10^-7^) | 6.35 x 10^-7^ (1.225 x 10^-7^, 1.382 x 10^-6^) | 2.70 x 10^-7^ (7.157 x 10^-8^, 8.818 x 10^-7^) | 0.02 (0.015, 0.021) |
| **35** | 3.71 x 10^-7^ (1.395 x 10^-7^, 7.774 x 10^-7^) | 7.73 x 10^-7^ (1.343 x 10^-7^, 1.513 x 10^-6^) | 2.48 x 10^-7^ (6.295 x 10^-8^, 9.109 x 10^-7^) | 0.02 (0.015, 0.021) |
| **40** | 4.48 x 10^-7^ (1.511 x 10^-7^, 8.945 x 10^-7^) | 7.52 x 10^-7^ (1.718 x 10^-7^, 1.765 x 10^-6^) | 3.15 x 10^-7^ (7.646 x 10^-8^, 1.009 x 10^-6^) | 0.02 (0.015, 0.020) |
| **45** | 3.68 x 10^-7^ (1.439 x 10^-7^, 6.937 x 10^-7^) | 7.07 x 10^-7^ (1.722 x 10^-7^, 1.681 x 10^-6^) | 2.80 x 10^-7^ (7.419 x 10^-8^, 9.679 x 10^-7^) | 0.02 (0.015, 0.020) |
| **50** | 3.04 x 10^-7^ (1.288 x 10^-7^, 5.828 x 10^-7^) | 8.13 x 10^-7^ (1.964 x 10^-7^, 2.011 x 10^-6^) | 3.05 x 10^-7^ (9.720 x 10^-8^, 9.852 x 10^-7^) | 0.02 (0.015, 0.020) |
| **55** | 3.22 x 10^-7^ (1.210 x 10^-7^, 6.366 x 10^-7^) | 7.61 x 10^-7^ (1.776 x 10^-7^, 2.014 x 10^-6^) | 3.17 x 10^-7^ (8.889 x 10^-8^, 1.006 x 10^-6^) | 0.02 (0.016, 0.020) |
| **60** | 3.36 x 10^-7^ (1.291 x 10^-7^, 6.648 x 10^-7^) | 8.15 x 10^-7^ (1.997 x 10^-7^, 1.981 x 10^-6^) | 3.19 x 10^-7^ (8.773 x 10^-8^, 9.677 x 10^-7^) | 0.02 (0.016, 0.020) |
| **65** | 3.82 x 10^-7^ (1.318 x 10^-7^, 7.276 x 10^-7^) | 7.91 x 10^-7^ (1.767 x 10^-7^, 1.911 x 10^-6^) | 4.45 x 10^-7^ (1.111 x 10^-7^, 1.201 x 10^-6^) | 0.02 (0.015, 0.020) |
| **70** | 5.14 x 10^-7^ (1.893 x 10^-7^, 9.723 x 10^-7^) | 6.55 x 10^-7^ (1.462 x 10^-7^, 1.740 x 10^-6^) | 4.85 x 10^-7^ (1.223 x 10^-7^, 1.394 x 10^-6^) | 0.02 (0.015, 0.020) |
| **75** | 6.18 x 10^-7^ (2.348 x 10^-7^, 1.206 x 10^-6^) | 1.28 x 10^-6^ (2.706 x 10^-7^, 3.049 x 10^-6^) | 4.50 x 10^-7^ (1.164 x 10^-7^, 1.460 x 10^-6^) | 0.02 (0.014, 0.020) |
| **80** | 5.26 x 10^-7^ (2.033 x 10^-7^, 1.031 x 10^-6^) | 1.99 x 10^-6^ (4.674 x 10^-7^, 5.528 x 10^-6^) | 3.78 x 10^-7^ (1.124 x 10^-7^, 1.316 x 10^-6^) | 0.02 (0.014, 0.020) |
| **85** | 3.46 x 10^-7^ (1.488 x 10^-7^, 7.360 x 10^-7^) | 4.37 x 10^-6^ (9.623 x 10^-7^, 1.072 x 10^-5^) | 4.91 x 10^-7^ (1.876 x 10^-7^, 2.239 x 10^-6^) | 0.02 (0.017, 0.023) |
| **90** | 1.50 x 10^-6^ (7.266 x 10^-7^, 3.611 x 10^-6^) | 1.41 x 10^-5^ (2.847 x 10^-6^, 3.539 x 10^-5^) | 6.36 x 10^-7^ (2.272 x 10^-7^, 3.057 x 10^-6^) | 0.03 (0.022, 0.033) |
| **95** | 3.21 x 10^-6^ (1.442 x 10^-6^, 7.026 x 10^-6^) | 8.04 x 10^-6^ (1.996 x 10^-6^, 2.310 x 10^-5^) | 9.01 x 10^-7^ (3.656 x 10^-7^, 5.423 x 10^-6^) | 0.04 (0.029, 0.045) |

**Table A3**. Parameter output for an Ornstein-Uhlenbeck model with four optima corresponding to fossorial, natatorial, scansorial, and generalist mustelids. %, α, and σ^2^ denote percentage of bone length, the rate/strength of adaptation, and rate of stochastic evolution, respectively. θ_Fos,_ θ_Nat,_ θ_Sca_, and θ_Gen_ respectively denote the phenotypic optima (i.e., adaptive peaks) corresponding to fossorial, natatorial, scansorial, generalist mustelids. Parameter estimates are followed by 95% confidence limits in parentheses.

| **%** | **α** | **σ^2^** | **θ_Fos_** | **θ_Nat_** | **θ_Sca_** | **θ_Gen_** |
| --- | --- | --- | --- | --- | --- | --- |
|  | **Femoral CSA** | | | | | |
| **5** | 2.54 (0.199, 19.947) | 4.51 x 10^-4^ (2.459 x 10^-5^, 3.891 x 10^-3^) | 0.10 (0.090, 0.105) | 0.12 (0.117, 0.131) | 0.08 (0.077, 0.092) | 0.10 (0.090, 0.106) |
| **10** | 8.11 (0.237, 19.948) | 2.47 x 10^-3^ (4.822 x 10^-5^, 7.208 x 10^-3^) | 0.09 (0.085, 0.103) | 0.13 (0.121, 0.139) | 0.07 (0.056, 0.074) | 0.08 (0.073, 0.092) |
| **15** | 2.49 (0.185, 19.946) | 5.56 x 10^-4^ (3.046 x 10^-5^, 4.261 x 10^-3^) | 0.08 (0.068, 0.085) | 0.11 (0.098, 0.117) | 0.06 (0.051, 0.069) | 0.07 (0.061, 0.079) |
| **20** | 2.39 (0.197, 19.945) | 3.64 x 10^-4^ (2.277 x 10^-5^, 3.304 x 10^-3^) | 0.07 (0.066, 0.080) | 0.11 (0.100, 0.114) | 0.05 (0.046, 0.060) | 0.06 (0.057, 0.072) |
| **25** | 1.71 (0.178, 19.947) | 3.02 x 10^-4^ (2.356 x 10^-5^, 3.385 x 10^-3^) | 0.07 (0.061, 0.075) | 0.10 (0.091, 0.107) | 0.05 (0.043, 0.060) | 0.06 (0.051, 0.067) |
| **30** | 1.98 (0.184, 19.944) | 4.31 x 10^-4^ (2.679 x 10^-5^, 4.400 x 10^-3^) | 0.06 (0.052, 0.076) | 0.10 (0.088, 0.106) | 0.05 (0.041, 0.061) | 0.06 (0.049, 0.068) |
| **35** | 2.07 (0.188, 19.945) | 3.16 x 10^-4^ (1.902 x 10^-5^, 3.087 x 10^-3^) | 0.06 (0.057, 0.070) | 0.09 (0.087, 0.102) | 0.05 (0.043, 0.059) | 0.06 (0.052, 0.066) |
| **40** | 1.92 (0.178, 19.946) | 2.72 x 10^-4^ (1.851 x 10^-5^, 2.901 x 10^-3^) | 0.06 (0.057, 0.069) | 0.09 (0.085, 0.099) | 0.05 (0.042, 0.058) | 0.06 (0.051, 0.065) |
| **45** | 2.18 (0.190, 19.944) | 2.79 x 10^-4^ (1.631 x 10^-5^, 2.552 x 10^-3^) | 0.06 (0.055, 0.068) | 0.09 (0.083, 0.096) | 0.05 (0.042, 0.056) | 0.06 (0.050, 0.065) |
| **50** | 2.27 (0.182, 19.947) | 2.56 x 10^-4^ (1.468 x 10^-5^, 2.339 x 10^-3^) | 0.06 (0.055, 0.066) | 0.09 (0.083, 0.095) | 0.05 (0.042, 0.055) | 0.06 (0.049, 0.062) |
| **55** | 1.67 (0.186, 19.945) | 1.90 x 10^-4^ (1.461 x 10^-5^, 2.301 x 10^-3^) | 0.06 (0.054, 0.065) | 0.09 (0.082, 0.095) | 0.05 (0.041, 0.054) | 0.06 (0.050, 0.063) |
| **60** | 1.39 (0.195, 19.947) | 1.51 x 10^-4^ (1.525 x 10^-5^, 2.196 x 10^-3^) | 0.06 (0.055, 0.066) | 0.09 (0.082, 0.094) | 0.05 (0.041, 0.054) | 0.06 (0.051, 0.063) |
| **65** | 1.10 (0.194, 19.945) | 1.29 x 10^-4^ (1.453 x 10^-5^, 2.370 x 10^-3^) | 0.06 (0.055, 0.066) | 0.09 (0.082, 0.096) | 0.05 (0.042, 0.056) | 0.06 (0.051, 0.064) |
| **70** | 1.40 (0.172, 19.946) | 2.20 x 10^-4^ (1.730 x 10^-5^, 3.049 x 10^-3^) | 0.06 (0.055, 0.068) | 0.09 (0.084, 0.100) | 0.05 (0.041, 0.057) | 0.06 (0.050, 0.066) |
| **75** | 1.40 (0.170, 19.946) | 2.54 x 10^-4^ (2.250 x 10^-5^, 3.484 x 10^-3^) | 0.06 (0.056, 0.070) | 0.09 (0.086, 0.102) | 0.05 (0.040, 0.058) | 0.06 (0.050, 0.067) |
| **80** | 1.17 (0.180, 19.947) | 2.73 x 10^-4^ (2.277 x 10^-5^, 4.540 x 10^-3^) | 0.07 (0.060, 0.076) | 0.11 (0.097, 0.116) | 0.05 (0.043, 0.061) | 0.06 (0.052, 0.070) |
| **85** | 3.94 (0.224, 19.948) | 1.59 x 10^-3^ (6.785 x 10^-5^, 9.435 x 10^-3^) | 0.08 (0.069, 0.090) | 0.14 (0.127, 0.150) | 0.06 (0.046, 0.066) | 0.07 (0.061, 0.085) |
| **90** | 3.42 (0.220, 19.948) | 1.17 x 10^-3^ (5.503 x 10^-5^, 7.720 x 10^-3^) | 0.11 (0.104, 0.124) | 0.16 (0.146, 0.166) | 0.08 (0.069, 0.086) | 0.10 (0.096, 0.116) |
| **95** | 2.09 (0.220, 19.947) | 2.67 x 10^-4^ (1.818 x 10^-5^, 2.886 x 10^-3^) | 0.11 (0.100, 0.111) | 0.13 (0.124, 0.135) | 0.09 (0.082, 0.095) | 0.10 (0.096, 0.109) |
|  | **Femoral SMA_ML_** | | | | | |
| **5** | 5.33 (0.210, 19.947) | 1.84 x 10^-4^ (5.825 x 10^-5^, 8.529 x 10^-4^) | 0.06 (0.053, 0.059) | 0.07 (0.063, 0.069) | 0.05 (0.043, 0.050) | 0.05 (0.050, 0.057) |
| **10** | 3.23 (0.226, 19.948) | 3.24 x 10^-4^ (1.709 x 10^-5^, 2.416 x 10^-4^) | 0.05 (0.047, 0.058) | 0.07 (0.063, 0.074) | 0.04 (0.032, 0.043) | 0.05 (0.042, 0.053) |
| **15** | 1.57 (0.180, 19.948) | 7.57 x 10^-5^ (6.836 x 10^-6^, 9.743 x 10^-4^) | 0.04 (0.040, 0.048) | 0.06 (0.055, 0.063) | 0.04 (0.033, 0.042) | 0.04 (0.038, 0.046) |
| **20** | 2.02 (0.208, 19.947) | 8.40 x 10^-5^ (5.591 x 10^-6^, 8.909 x 10^-4^) | 0.05 (0.042, 0.049) | 0.06 (0.059, 0.066) | 0.03 (0.031, 0.039) | 0.04 (0.036, 0.043) |
| **25** | 1.85 (0.191, 19.946) | 9.75 x 10^-5^ (6.738 x 10^-6^, 1.065 x 10^-4^) | 0.04 (0.040, 0.048) | 0.06 (0.053, 0.061) | 0.03 (0.028, 0.037) | 0.04 (0.033, 0.041) |
| **30** | 2.12 (0.183, 19.946) | 9.35 x 10^-5^ (5.210 x 10^-6^, 8.924 x 10^-4^) | 0.04 (0.037, 0.044) | 0.05 (0.050, 0.058) | 0.03 (0.028, 0.036) | 0.03 (0.031, 0.039) |
| **35** | 2.34 (0.201, 19.946) | 7.90 x 10^-5^ (4.543 x 10^-6^, 7.012 x 10^-4^) | 0.04 (0.036, 0.042) | 0.05 (0.048, 0.055) | 0.03 (0.028, 0.035) | 0.03 (0.032, 0.038) |
| **40** | 2.21 (0.202, 19.946) | 7.20 x 10^-5^ (4.070 x 10^-6^, 6.440 x 10^-4^) | 0.04 (0.034, 0.041) | 0.05 (0.047, 0.054) | 0.03 (0.028, 0.035) | 0.03 (0.032, 0.038) |
| **45** | 2.21 (0.211, 19.945) | 7.74 x 10^-5^ (4.797 x 10^-6^, 6.780 x 10^-4^) | 0.04 (0.034, 0.040) | 0.05 (0.047, 0.054) | 0.03 (0.028, 0.035) | 0.03 (0.031, 0.038) |
| **50** | 1.90 (0.183, 19.947) | 7.34 x 10^-5^ (5.153 x 10^-6^, 7.623 x 10^-4^) | 0.04 (0.034, 0.041) | 0.05 (0.048, 0.055) | 0.03 (0.027, 0.035) | 0.03 (0.030, 0.038) |
| **55** | 1.57 (0.195, 19.947) | 7.40 x 10^-5^ (6.314 x 10^-6^, 9.341 x 10^-4^) | 0.04 (0.034, 0.041) | 0.05 (0.048, 0.056) | 0.03 (0.027, 0.035) | 0.03 (0.031, 0.039) |
| **60** | 1.53 (0.195, 19.947) | 7.71 x 10^-5^ (6.216 x 10^-6^, 1.017 x 10^-3^) | 0.04 (0.035, 0.042) | 0.05 (0.048, 0.057) | 0.03 (0.027, 0.036) | 0.03 (0.031, 0.040) |
| **65** | 1.71 (0.150, 19.947) | 9.30 x 10^-5^ (6.788 x 10^-6^, 1.101 x 10^-3^) | 0.04 (0.035, 0.043) | 0.05 (0.049, 0.058) | 0.03 (0.027, 0.036) | 0.03 (0.031, 0.040) |
| **70** | 1.69 (0.162, 19.946) | 1.18 x 10^-4^ (8.803 x 10^-6^, 1.364 x 10^-3^) | 0.04 (0.036, 0.045) | 0.06 (0.051, 0.061) | 0.03 (0.026, 0.037) | 0.04 (0.031, 0.041) |
| **75** | 1.95 (0.183, 19.944) | 1.46 x 10^-4^ (1.002 x 10^-5^, 1.503 x 10^-3^) | 0.04 (0.038, 0.046) | 0.06 (0.053, 0.064) | 0.03 (0.027, 0.038) | 0.04 (0.032, 0.043) |
| **80** | 2.72 (0.190, 19.947) | 2.45 x 10^-4^ (1.261 x 10^-5^, 2.058 x 10^-3^) | 0.05 (0.041, 0.051) | 0.07 (0.060, 0.071) | 0.03 (0.029, 0.040) | 0.04 (0.035, 0.046) |
| **85** | 4.13 (0.216, 19.948) | 8.16 x 10^-4^ (3.298 x 10^-5^, 4.689 x 10^-3^) | 0.05 (0.047, 0.062) | 0.09 (0.083, 0.098) | 0.04 (0.031, 0.046) | 0.05 (0.042, 0.057) |
| **90** | 3.40 (0.232, 19.948) | 3.50 x 10^-4^ (1.922 x 10^-5^, 2.582 x 10^-3^) | 0.08 (0.072, 0.082) | 0.10 (0.098, 0.109) | 0.05 (0.031, 0.046) | 0.07 (0.067, 0.077) |
| **95** | 2.84 (0.210, 19.948) | 8.25 x 10^-5^ (4.658 x 10^-6^, 6.779 x 10^-4^) | 0.07 (0.067, 0.073) | 0.09 (0.083, 0.089) | 0.06 (0.057, 0.063) | 0.07 (0.064, 0.070) |
|  | **Femoral SMA_CC_** | | | | | |
| **5** | 3.63 (0.191, 19.948) | 4.52 x 10^-4^ (1.727 x 10^-5^, 2.613 x 10^-3^) | 0.08 (0.075, 0.087) | 0.10 (0.095, 0.108) | 0.07 (0.062, 0.076) | 0.07 (0.068, 0.082) |
| **10** | 2.90 (0.191, 19.946) | 8.22 x 10^-4^ (3.384 x 10^-5^, 5.554 x 10^-3^) | 0.08 (0.074, 0.092) | 0.11 (0.099, 0.120) | 0.05 (0.042, 0.064) | 0.06 (0.054, 0.076) |
| **15** | 0.40 (0.163, 19.946) | 9.92 x 10^-5^ (2.532 x 10^-5^, 4.775 x 10^-3^) | 0.06 (0.053, 0.070) | 0.09 (0.076, 0.096) | 0.04 (0.033, 0.056) | 0.05 (0.041, 0.061) |
| **20** | 0.62 (0.150, 19.946) | 7.63 x 10^-5^ (1.282 x 10^-5^, 2.367 x 10^-3^) | 0.06 (0.050, 0.062) | 0.08 (0.068, 0.084) | 0.04 (0.029, 0.046) | 0.04 (0.039, 0.053) |
| **25** | 0.85 (0.170, 19.946) | 1.09 x 10^-4^ (1.572 x 10^-5^, 2.621 x 10^-3^) | 0.05 (0.043, 0.055) | 0.07 (0.062, 0.076) | 0.03 (0.027, 0.043) | 0.04 (0.034, 0.048) |
| **30** | 1.05 (0.147, 19.946) | 1.11 x 10^-4^ (1.038 x 10^-5^, 1.995 x 10^-3^) | 0.04 (0.038, 0.050) | 0.06 (0.062, 0.076) | 0.03 (0.026, 0.040) | 0.04 (0.033, 0.046) |
| **35** | 1.45 (0.182, 19.945) | 8.47 x 10^-5^ (7.789 x 10^-6^, 1.107 x 10^-3^) | 0.04 (0.037, 0.045) | 0.06 (0.054, 0.063) | 0.03 (0.027, 0.037) | 0.04 (0.033, 0.043) |
| **40** | 1.57 (0.182, 19.947) | 7.49 x 10^-5^ (5.976 x 10^-6^, 9.463 x 10^-4^) | 0.04 (0.036, 0.043) | 0.06 (0.052, 0.060) | 0.03 (0.027, 0.036) | 0.04 (0.033, 0.041) |
| **45** | 1.54 (0.187, 19.947) | 6.84 x 10^-5^ (5.406 x 10^-6^, 8.580 x 10^-4^) | 0.04 (0.035, 0.043) | 0.05 (0.052, 0.059) | 0.03 (0.027, 0.036) | 0.04 (0.032, 0.040) |
| **50** | 0.94 (0.182, 19.946) | 4.48 x 10^-5^ (6.164 x 10^-6^, 9.218 x 10^-4^) | 0.04 (0.035, 0.043) | 0.06 (0.052, 0.060) | 0.03 (0.027, 0.036) | 0.04 (0.032, 0.041) |
| **55** | 0.71 (0.186, 19.946) | 4.03 x 10^-5^ (7.613 x 10^-6^, 1.119 x 10^-3^) | 0.04 (0.036, 0.044) | 0.06 (0.052, 0.061) | 0.03 (0.028, 0.037) | 0.04 (0.032, 0.041) |
| **60** | 0.69 (0.186, 19.946) | 4.92 x 10^-5^ (7.981 x 10^-6^, 1.380 x 10^-3^) | 0.04 (0.037, 0.046) | 0.06 (0.053, 0.063) | 0.03 (0.028, 0.039) | 0.04 (0.033, 0.043) |
| **65** | 0.67 (0.182, 19.946) | 6.05 x 10^-5^ (1.149 x 10^-5^, 1.698 x 10^-3^) | 0.04 (0.037, 0.048) | 0.06 (0.055, 0.067) | 0.03 (0.028, 0.040) | 0.04 (0.033, 0.044) |
| **70** | 0.74 (0.152, 19.945) | 9.12 x 10^-5^ (1.370 x 10^-5^, 2.320 x 10^-3^) | 0.04 (0.038, 0.051) | 0.06 (0.058, 0.072) | 0.03 (0.027, 0.041) | 0.04 (0.033, 0.046) |
| **75** | 0.88 (0.160, 19.945) | 1.19 x 10^-4^ (1.419 x 10^-5^, 2.328 x 10^-3^) | 0.05 (0.041, 0.054) | 0.07 (0.062, 0.077) | 0.03 (0.028, 0.043) | 0.04 (0.033, 0.047) |
| **80** | 1.20 (0.175, 19.945) | 1.83 x 10^-4^ (1.773 x 10^-5^, 2.850x 10^-3^) | 0.05 (0.046, 0.060) | 0.08 (0.070, 0.086) | 0.04 (0.029, 0.046) | 0.04 (0.036, 0.051) |
| **85** | 3.01 (0.216, 19.948) | 6.77 x 10^-4^ (3.845 x 10^-5^, 5.152 x 10^-3^) | 0.06 (0.055, 0.070) | 0.10 (0.092, 0.108) | 0.04 (0.034, 0.052) | 0.05 (0.046, 0.061) |
| **90** | 2.96 (0.237, 19.947) | 4.07 x 10^-4^ (2.433 x 10^-5^, 3.160 x 10^-3^) | 0.08 (0.078, 0.090) | 0.11 (0.106, 0.118) | 0.06 (0.050, 0.064) | 0.08 (0.072, 0.084) |
| **95** | 3.09 (0.182, 19.947) | 1.85 x 10^-4^ (8.085 x 10^-6^, 1.290 x 10^-3^) | 0.08 (0.075, 0.083) | 0.10 (0.091, 0.100) | 0.07 (0.063, 0.072) | 0.07 (0.071, 0.080) |
|  | **Tibial CSA** | | | | | |
| **5** | 4.91 (0.187, 19.946) | 1.24 x 10^-3^ (3.519 x 10^-5^, 5.336 x 10^-3^) | 0.12 (0.110, 0.137) | 0.13 (0.119, 0.137) | 0.09 (0.084, 0.104) | 0.10 (0.085, 0.105) |
| **10** | 0.03 (2.061 x 10^-9^, 12.366) | 3.06 x 10^-5^ (1.554 x 10^-5^, 2.485 x 10^-3^) | 0.11 (0.093, 0.130) | 0.13 (0.083, 0.143) | 0.04 (0.052, 0.128) | 0.07 (0.074, 0.129) |
| **15** | 4.82 x 10^-3^ (2.061 x 10^-9^, 9.371 x 10^-3^) | 9.17 x 10^-6^ (4.931 x 10^-6^, 7.249 x 10^-5^) | 0.09 (0.074, 0.099) | 0.09 (0.064, 0.108) | 0.04 (0.062, 0.114) | 0.06 (0.069, 0.106) |
| **20** | 0.05 (2.453 x 10^-9^, 15.197) | 9.62 x 10^-6^ (4.677 x 10^-6^, 9.231 x 10^-4^) | 0.08 (0.069, 0.087) | 0.08 (0.065, 0.092) | 0.04 (0.044, 0.079) | 0.06 (0.056, 0.081) |
| **25** | 0.04 (2.061 x 10^-9^, 5.313) | 7.02 x 10^-6^ (3.525 x 10^-6^, 3.327 x 10^-4^) | 0.07 (0.066, 0.083) | 0.08 (0.062, 0.090) | 0.04 (0.047, 0.079) | 0.05 (0.056, 0.080) |
| **30** | 0.04 (2.061 x 10^-9^, 3.494) | 6.40 x 10^-6^ (3.012 x 10^-6^, 1.211 x 10^-4^) | 0.07 (0.066, 0.080) | 0.08 (0.063, 0.086) | 0.04 (0.045, 0.074) | 0.05 (0.054, 0.076) |
| **35** | 0.07 (0.016, 16.328) | 8.00 x 10^-6^ (3.833 x 10^-6^, 6.635 x 10^-4^) | 0.07 (0.065, 0.078) | 0.08 (0.063, 0.084) | 0.04 (0.040, 0.066) | 0.05 (0.051, 0.070) |
| **40** | 0.07 (0.021, 16.783) | 8.09 x 10^-6^ (4.128 x 10^-6^, 6.355 x 10^-4^) | 0.07 (0.064, 0.076) | 0.08 (0.063, 0.083) | 0.04 (0.041, 0.065) | 0.05 (0.049, 0.068) |
| **45** | 0.07 (0.026, 15.421) | 8.89 x 10^-6^ (4.155 x 10^-6^, 7.349 x 10^-4^) | 0.07 (0.064, 0.077) | 0.08 (0.062, 0.085) | 0.04 (0.038, 0.065) | 0.05 (0.048, 0.067) |
| **50** | 0.07 (0.019, 17.414) | 9.36 x 10^-6^ (4.441 x 10^-6^, 8.830 x 10^-4^) | 0.07 (0.063, 0.077) | 0.08 (0.063, 0.085) | 0.04 (0.039, 0.067) | 0.05 (0.048, 0.069) |
| **55** | 0.05 (3.628 x 10^-9^, 16.866) | 9.23 x 10^-6^ (4.332 x 10^-6^, 8.433 x 10^-4^) | 0.07 (0.061, 0.077) | 0.08 (0.060, 0.086) | 0.04 (0.038, 0.070) | 0.05 (0.048, 0.072) |
| **60** | 0.04 (2.061 x 10^-9^, 13.132) | 8.28 x 10^-6^ (3.928 x 10^-6^, 6.129 x 10^-4^) | 0.07 (0.060, 0.077) | 0.08 (0.060, 0.085) | 0.04 (0.038, 0.073) | 0.05 (0.047, 0.073) |
| **65** | 0.01 (2.061 x 10^-9^, 2.479) | 5.85 x 10^-6^ (2.935 x 10^-6^, 1.009 x 10^-4^) | 0.07 (0.058, 0.076) | 0.08 (0.050, 0.084) | 0.03 (0.046, 0.085) | 0.04 (0.050, 0.081) |
| **70** | 0.02 (2.061 x 10^-9^, 2.587) | 5.12 x 10^-6^ (2.530 x 10^-6^, 7.233 x 10^-5^) | 0.07 (0.058, 0.074) | 0.07 (0.054, 0.082) | 0.03 (0.043, 0.076) | 0.05 (0.050, 0.075) |
| **75** | 0.03 (2.061 x 10^-9^, 4.442) | 5.93 x 10^-6^ (2.842 x 10^-6^, 1.886 x 10^-4^) | 0.07 (0.058, 0.074) | 0.08 (0.055, 0.083) | 0.03 (0.040, 0.074) | 0.05 (0.049, 0.073) |
| **80** | 0.03 (2.061 x 10^-9^, 2.572) | 7.25 x 10^-6^ (3.479 x 10^-6^, 1.609 x 10^-4^) | 0.07 (0.060, 0.076) | 0.08 (0.058, 0.087) | 0.03 (0.040, 0.073) | 0.05 (0.050, 0.075) |
| **85** | 0.07 (0.021, 18.416) | 1.51 x 10^-5^ (7.490 x 10^-6^, 1.602 x 10^-3^) | 0.07 (0.064, 0.082) | 0.09 (0.070, 0.097) | 0.03 (0.034, 0.068) | 0.05 (0.050, 0.075) |
| **90** | 0.42 (0.093, 19.929) | 1.05 x 10^-4^ (1.800 x 10^-5^, 3.209 x 10^-3^) | 0.09 (0.076, 0.095) | 0.11 (0.091, 0.117) | 0.05 (0.041, 0.074) | 0.06 (0.056, 0.080) |
| **95** | 6.55 (0.203, 19.948) | 1.82 x 10^-3^ (4.095 x 10^-5^, 6.213 x 10^-3^) | 0.07 (0.066, 0.083) | 0.07 (0.058, 0.076) | 0.07 (0.061, 0.080) | 0.07 (0.061, 0.080) |
|  | **Tibial SMA_ML_** | | | | | |
| **5** | 0.37 (0.088, 19.929) | 1.83 x 10^-5^ (3.624 x 10^-6^, 6.512 x 10^-4^) | 0.08 (0.074, 0.083) | 0.08 (0.075, 0.086) | 0.06 (0.057, 0.071) | 0.06 (0.062, 0.073) |
| **10** | 0.03 (2.061 x 10^-9^, 13.565) | 1.64 x 10^-5^ (8.893 x 10^-6^, 1.215 x 10^-3^) | 0.08 (0.067, 0.094) | 0.08 (0.058, 0.105) | 0.03 (0.040, 0.095) | 0.06 (0.056, 0.098) |
| **15** | 2.69 x 10^-3^ (2.061 x 10^-9^, 0.889) | 5.46 x 10^-6^ (3.091 x 10^-6^, 4.518 x 10^-5^) | 0.07 (0.058, 0.078) | 0.06 (0.050, 0.086) | 0.03 (0.045, 0.089) | 0.05 (0.050, 0.083) |
| **20** | 0.02 (2.061 x 10^-9^, 4.541) | 4.33 x 10^-6^ (2.266 x 10^-6^, 1.899 x 10^-4^) | 0.06 (0.052, 0.067) | 0.06 (0.046, 0.072) | 0.03 (0.039, 0.070) | 0.04 (0.045, 0.068) |
| **25** | 0.02 (2.061 x 10^-9^, 13.237) | 3.58 x 10^-6^ (1.812 x 10^-6^, 3.123 x 10^-4^) | 0.06 (0.049, 0.062) | 0.05 (0.045, 0.067) | 0.03 (0.036, 0.062) | 0.04 (0.042, 0.063) |
| **30** | 0.01 (2.061 x 10^-9^, 0.661) | 2.61 x 10^-6^ (1.380 x 10^-6^, 1.750 x 10^-5^) | 0.05 (0.047, 0.060) | 0.05 (0.042, 0.064) | 0.03 (0.038, 0.064) | 0.04 (0.042, 0.061) |
| **35** | 0.0 (2.061 x 10^-9^, 12.585) | 2.64 x 10^-6^ (1.342 x 10^-6^, 2.292 x 10^-4^) | 0.05 (0.044, 0.056) | 0.05 (0.041, 0.060) | 0.03 (0.033, 0.057) | 0.04 (0.039, 0.056) |
| **40** | 0.04 (3.595 x 10^-9^, 15.341) | 3.01 x 10^-6^ (1.458 x 10^-6^, 2.980 x 10^-4^) | 0.05 (0.043, 0.053) | 0.05 (0.039, 0.057) | 0.03 (0.029, 0.049) | 0.03 (0.035, 0.050) |
| **45** | 0.05 (2.776 x 10^-9^, 19.422) | 3.10 x 10^-6^ (1.588 x 10^-6^, 3.404 x 10^-4^) | 0.05 (0.041, 0.051) | 0.05 (0.039, 0.054) | 0.03 (0.027, 0.046) | 0.03 (0.034, 0.048) |
| **50** | 0.05 (7.775 x 10^-9^, 17.398) | 3.29 x 10^-6^ (1.622 x 10^-6^, 3.615 x 10^-4^) | 0.04 (0.039, 0.049) | 0.05 (0.037, 0.052) | 0.03 (0.025, 0.045) | 0.03 (0.032, 0.045) |
| **55** | 0.06 (5.582 x 10^-9^, 17.169) | 3.54 x 10^-6^ (1.623 x 10^-6^, 3.046 x 10^-4^) | 0.04 (0.037, 0.047) | 0.04 (0.035, 0.051) | 0.03 (0.024, 0.043) | 0.03 (0.030, 0.044) |
| **60** | 0.06 (0.006, 18.323) | 3.49 x 10^-6^ (1.627 x 10^-6^, 3.475 x 10^-4^) | 0.04 (0.036, 0.046) | 0.04 (0.034, 0.049) | 0.02 (0.023, 0.042) | 0.03 (0.029, 0.043) |
| **65** | 0.06 (7.434 x 10^-9^, 16.974) | 3.08 x 10^-6^ (1.520 x 10^-6^, 2.749 x 10^-4^) | 0.04 (0.036, 0.045) | 0.04 (0.033, 0.048) | 0.02 (0.023, 0.041) | 0.03 (0.028, 0.042) |
| **70** | 0.07 (0.017, 19.281) | 3.03 x 10^-6^ (1.467 x 10^-6^, 3.077 x 10^-4^) | 0.04 (0.036, 0.044) | 0.04 (0.033, 0.048) | 0.02 (0.023, 0.038) | 0.03 (0.028, 0.040) |
| **75** | 0.09 (0.025, 19.892) | 3.63 x 10^-6^ (1.609 x 10^-6^, 3.729 x 10^-4^) | 0.04 (0.036, 0.044) | 0.04 (0.035, 0.047) | 0.02 (0.023, 0.037) | 0.03 (0.028, 0.039) |
| **80** | 0.10 (0.032, 19.186) | 4.53 x 10^-6^ (2.145 x 10^-6^, 4.520 x 10^-4^) | 0.04 (0.038, 0.046) | 0.05 (0.038, 0.051) | 0.03 (0.023, 0.038) | 0.03 (0.028, 0.039) |
| **85** | 0.15 (0.057, 19.861) | 1.09 x 10^-5^ (3.693 x 10^-6^, 8.038 x 10^-4^) | 0.05 (0.041, 0.051) | 0.05 (0.045, 0.059) | 0.03 (0.023, 0.041) | 0.03 (0.029, 0.043) |
| **90** | 0.15 (0.046, 19.396) | 1.53 x 10^-5^ (4.612 x 10^-6^, 9.697 x 10^-4^) | 0.06 (0.050, 0.061) | 0.07 (0.054, 0.073) | 0.03 (0.027, 0.049) | 0.04 (0.035, 0.053) |
| **95** | 0.86 (0.176, 19.948) | 5.62 x 10^-5^ (7.869 x 10^-6^, 1.422 x 10^-3^) | 0.05 (0.045, 0.054) | 0.05 (0.041, 0.050) | 0.04 (0.038, 0.049) | 0.04 (0.040, 0.050) |
|  | **Tibial SMA_CC_** | | | | | |
| **5** | 0.13 (0.033, 19.549) | 1.72 x 10^-5^ (6.799 x 10^-6^, 1.098 x 10^-3^) | 0.09 (0.083, 0.098) | 0.10 (0.084, 0.106) | 0.06 (0.055, 0.081) | 0.07 (0.066, 0.087) |
| **10** | 0.01 (2.061 x 10^-9^, 0.932) | 1.53 x 10^-5^ (7.619 x 10^-6^, 1.248 x 10^-4^) | 0.08 (0.064, 0.097) | 0.09 (0.053, 0.109) | 0.02 (0.046, 0.110) | 0.05 (0.054, 0.105) |
| **15** | 4.49 x 10^-3^ (2.061 x 10^-9^, 1.150) | 4.93 x 10^-6^ (2.680 x 10^-6^, 4.263 x 10^-5^) | 0.06 (0.051, 0.069) | 0.06 (0.042, 0.078) | 0.02 (0.040, 0.078) | 0.04 (0.045, 0.074) |
| **20** | 0.08 (0.017, 18.678) | 5.55 x 10^-6^ (2.424 x 10^-6^, 5.233 x 10^-4^) | 0.05 (0.043, 0.054) | 0.05 (0.040, 0.056) | 0.03 (0.025, 0.045) | 0.03 (0.034, 0.048) |
| **25** | 0.07 (0.016, 19.443) | 4.03 x 10^-6^ (1.911 x 10^-6^, 4.469 x 10^-4^) | 0.04 (0.040, 0.050) | 0.04 (0.036, 0.052) | 0.03 (0.025, 0.043) | 0.03 (0.031, 0.045) |
| **30** | 0.08 (0.030, 19.778) | 3.48 x 10^-6^ (1.577 x 10^-6^, 3.763 x 10^-4^) | 0.04 (0.038, 0.046) | 0.04 (0.036, 0.048) | 0.03 (0.024, 0.040) | 0.03 (0.030, 0.041) |
| **35** | 0.10 (0.047, 19.795) | 3.31 x 10^-6^ (1.480 x 10^-6^, 3.309 x 10^-4^) | 0.04 (0.036, 0.043) | 0.04 (0.036, 0.046) | 0.03 (0.024, 0.037) | 0.03 (0.028, 0.038) |
| **40** | 0.09 (0.040, 19.832) | 3.12 x 10^-6^ (1.591 x 10^-6^, 2.956 x 10^-4^) | 0.04 (0.035, 0.042) | 0.04 (0.036, 0.045) | 0.03 (0.024, 0.036) | 0.03 (0.027, 0.037) |
| **45** | 0.10 (0.037, 19.834) | 3.41 x 10^-6^ (1.533 x 10^-6^, 2.988 x 10^-4^) | 0.04 (0.034, 0.041) | 0.04 (0.036, 0.045) | 0.03 (0.022, 0.036) | 0.03 (0.027, 0.038) |
| **50** | 0.08 (0.011, 18.138) | 3.07 x 10^-6^ (1.466 x 10^-6^, 3.071 x 10^-4^) | 0.04 (0.034, 0.042) | 0.04 (0.036, 0.047) | 0.02 (0.022, 0.037) | 0.03 (0.027, 0.038) |
| **55** | 0.04 (2.743 x 10^-9^, 12.280) | 2.73 x 10^-6^ (1.331 x 10^-6^, 1.579 x 10^-4^) | 0.04 (0.034, 0.043) | 0.04 (0.033, 0.048) | 0.02 (0.022, 0.041) | 0.03 (0.028, 0.041) |
| **60** | 0.02 (2.061 x 10^-9^, 1.067) | 2.24 x 10^-6^ (1.071 x 10^-6^, 1.946 x 10^-5^) | 0.04 (0.034, 0.045) | 0.04 (0.030, 0.050) | 0.02 (0.025, 0.050) | 0.03 (0.029, 0.047) |
| **65** | 0.01 (2.061 x 10^-9^, 0.582) | 2.18 x 10^-6^ (1.058 x 10^-6^, 1.128 x 10^-5^) | 0.04 (0.035, 0.046) | 0.05 (0.030, 0.051) | 0.02 (0.029, 0.052) | 0.03 (0.031, 0.050) |
| **70** | 0.02 (2.061 x 10^-9^, 2.502) | 2.45 x 10^-6^ (1.292 x 10^-6^, 8.293 x 10^-5^) | 0.04 (0.036, 0.047) | 0.05 (0.033, 0.052) | 0.02 (0.026, 0.049) | 0.03 (0.031, 0.048) |
| **75** | 0.05 (2.282 x 10^-9^, 15.184) | 3.73 x 10^-6^ (1.837 x 10^-6^, 3.423 x 10^-4^) | 0.04 (0.038, 0.048) | 0.05 (0.037, 0.053) | 0.02 (0.023, 0.044) | 0.03 (0.029, 0.044) |
| **80** | 0.07 (0.015, 15.559) | 5.63 x 10^-6^ (2.536 x 10^-6^, 5.087 x 10^-4^) | 0.05 (0.040, 0.052) | 0.05 (0.041, 0.058) | 0.02 (0.021, 0.043) | 0.03 (0.030, 0.046) |
| **85** | 0.10 (0.031, 19.344) | 1.11 x 10^-5^ (4.079 x 10^-6^, 8.410 x 10^-4^) | 0.05 (0.045, 0.059) | 0.06 (0.048, 0.067) | 0.02 (0.020, 0.046) | 0.03 (0.032, 0.051) |
| **90** | 0.11 (0.040, 19.103) | 1.26 x 10^-5^ (4.079 x 10^-6^, 8.410 x 10^-3^) | 0.06 (0.054, 0.068) | 0.07 (0.057, 0.076) | 0.03 (0.026, 0.052) | 0.05 (0.043, 0.060) |
| **95** | 2.57 (0.195, 19.948) | 2.57 x 10^-4^ (1.461 x 10^-5^, 2.273 x 10^-3^) | 0.05 (0.043, 0.053) | 0.04 (0.038, 0.049) | 0.05 (0.040, 0.052) | 0.04 (0.038, 0.049) |
|  | **Fibular CSA** | | | | | |
| **5** | 0.31 (0.129, 19.937) | 1.92 x 10^-5^ (5.586 x 10^-6^, 9.529 x 10^-4^) | 0.05 (0.045, 0.055) | 0.05 (0.045, 0.059) | 0.02 (0.016, 0.030) | 0.04 (0.036, 0.047) |
| **10** | 5.17 x 10^-3^ (2.061 x 10^-9^, 0.800) | 2.19 x 10^-6^ (1.153 x 10^-6^, 1.382 x 10^-4^) | 0.04 (0.032, 0.045) | 0.04 (0.028, 0.049) | 0.01 (0.027, 0.051) | 0.03 (0.029, 0.048) |
| **15** | 0.08 (0.042, 16.716) | 2.13 x 10^-6^ (1.166 x 10^-6^, 1.689 x 10^-4^) | 0.03 (0.029, 0.036) | 0.03 (0.028, 0.037) | 0.01 (0.014, 0.026) | 0.02 (0.023, 0.032) |
| **20** | 0.08 (0.014, 19.626) | 1.64 x 10^-6^ (6.943 x 10^-7^, 1.502 x 10^-4^) | 0.03 (0.029, 0.035) | 0.03 (0.027, 0.036) | 0.01 (0.015, 0.027) | 0.02 (0.023, 0.031) |
| **25** | 0.09 (0.033, 15.574) | 1.71 x 10^-6^ (8.167 x 10^-7^, 1.456 x 10^-4^) | 0.03 (0.029, 0.035) | 0.03 (0.028, 0.036) | 0.02 (0.016, 0.026) | 0.02 (0.024, 0.032) |
| **30** | 0.04 ^3^ (2.135 x 10^-9^, 3.060) | 1.24 x 10^-6^ (6.039 x 10^-7^, 3.117 x 10^-5^) | 0.03 (0.030, 0.036) | 0.04 (0.028, 0.039) | 0.02 (0.018, 0.031) | 0.02 (0.025, 0.034) |
| **35** | 0.07 (0.026, 13.782) | 1.73 x 10^-6^ (7.424 x 10^-7^, 1.276 x 10^-4^) | 0.03 (0.030, 0.036) | 0.03 (0.029, 0.039) | 0.02 (0.018, 0.030) | 0.02 (0.025, 0.034) |
| **40** | 0.13 (0.068, 19.785) | 2.50 x 10^-6^ (1.138 x 10^-6^, 1.779 x 10^-4^) | 0.03 (0.030, 0.035) | 0.04 (0.031, 0.039) | 0.02 (0.017, 0.027) | 0.03 (0.025, 0.032) |
| **45** | 0.15 (0.085, 19.813) | 2.44 x 10^-6^ (1.139 x 10^-6^, 1.525 x 10^-4^) | 0.03 (0.031, 0.035) | 0.04 (0.033, 0.039) | 0.02 (0.018, 0.027) | 0.03 (0.025, 0.031) |
| **50** | 0.22 (0.102, 19.923) | 2.96 x 10^-6^ (1.085 x 10^-6^, 1.925 x 10^-4^) | 0.03 (0.031, 0.035) | 0.04 (0.033, 0.039) | 0.02 (0.018, 0.026) | 0.03 (0.026, 0.031) |
| **55** | 0.26 (0.113, 19.849) | 3.35 x 10^-6^ (9.958 x 10^-7^, 1.525 x 10^-4^) | 0.03 (0.031, 0.035) | 0.04 (0.034, 0.039) | 0.02 (0.018, 0.026) | 0.03 (0.025, 0.031) |
| **60** | 0.36 (0.115, 19.921) | 4.47 x 10^-6^ (1.093 x 10^-6^, 1.882 x 10^-4^) | 0.03 (0.031, 0.035) | 0.04 (0.033, 0.038) | 0.02 (0.018, 0.025) | 0.03 (0.025, 0.030) |
| **65** | 0.62 (0.147, 19.945) | 9.04 x 10^-6^ (1.628 x 10^-6^, 2.629 x 10^-4^) | 0.03 (0.031, 0.035) | 0.04 (0.033, 0.038) | 0.02 (0.019, 0.026) | 0.03 (0.025, 0.030) |
| **70** | 0.55 (0.123, 19.944) | 9.08 x 10^-6^ (1.539 x 10^-6^, 3.005 x 10^-4^) | 0.03 (0.030, 0.035) | 0.04 (0.033, 0.039) | 0.02 (0.019, 0.025) | 0.03 (0.025, 0.030) |
| **75** | 1.39 (0.154, 19.946) | 3.03 x 10^-5^ (0.030 x 10^-6^, 0.035 x 10^-4^) | 0.03 (0.030, 0.035) | 0.04 (0.034, 0.040) | 0.02 (0.019, 0.026) | 0.03 (0.024, 0.030) |
| **80** | 1.15 (0.165, 19.944) | 3.83 x 10^-5^ (0.030 x 10^-6^, 0.035 x 10^-4^) | 0.03 (0.029, 0.036) | 0.04 (0.036, 0.043) | 0.02 (0.019, 0.027) | 0.03 (0.025, 0.033) |
| **85** | 0.50 (0.126, 19.941) | 2.34 x 10^-5^ (4.719 x 10^-6^, 7.752 x 10^-4^) | 0.04 (0.032, 0.040) | 0.04 (0.040, 0.049) | 0.02 (0.028, 0.037) | 0.03 (0.028, 0.037) |
| **90** | 0.99 (0.152, 19.945) | 7.83 x 10^-5^ (9.094 x 10^-6^, 1.481 x 10^-3^) | 0.04 (0.040, 0.049) | 0.06 (0.049, 0.061) | 0.03 (0.020, 0.032) | 0.04 (0.034, 0.045) |
| **95** | 0.39 (0.148, 19.943) | 2.82 x 10^-5^ (7.937 x 10^-6^, 1.205 x 10^-3^) | 0.06 (0.050, 0.060) | 0.07 (0.064, 0.076) | 0.03 (0.030, 0.044) | 0.05 (0.046, 0.058) |
|  | **Fibular SMA_ML_** | | | | | |
| **5** | 0.37 (0.177, 19.932) | 1.54 x 10^-5^ (4.979 x 10^-6^, 6.195 x 10^-4^) | 0.04 (0.034, 0.040) | 0.04 (0.039, 0.047) | 0.02 (0.014, 0.023) | 0.04 (0.033, 0.041) |
| **10** | 0.30 (0.127, 19.943) | 1.70 x 10^-5^ (5.607 x 10^-6^, 1.024 x 10^-3^) | 0.02 (0.020, 0.029) | 0.03 (0.025, 0.036) | 0.01 (0.004, 0.016) | 0.02 (0.015, 0.025) |
| **15** | 0.22 (0.110, 19.926) | 3.02 x 10^-6^ (1.126 x 10^-6^, 2.063 x 10^-4^) | 0.02 (0.017, 0.021) | 0.02 (0.018, 0.023) | 0.01 (0.006, 0.013) | 0.01 (0.012, 0.018) |
| **20** | 0.17 (0.087, 19.914) | 1.63 x 10^-6^ (6.523 x 10^-7^, 1.141 x 10^-4^) | 0.02 (0.016, 0.019) | 0.02 (0.016, 0.021) | 0.01 (0.007, 0.013) | 0.01 (0.012, 0.017) |
| **25** | 0.13 (0.057, 19.930) | 1.30 x 10^-6^ (5.861 x 10^-7^, 1.187 x 10^-4^) | 0.02 (0.016, 0.019) | 0.02 (0.017, 0.022) | 0.01 (0.007, 0.014) | 0.01 (0.012, 0.017) |
| **30** | 0.03 (2.061 x 10^-9^, 2.702) | 6.66 x 10^-7^ (3.223 x 10^-7^, 1.236 x 10^-5^) | 0.02 (0.015, 0.021) | 0.02 (0.014, 0.023) | 0.01 (0.010, 0.020) | 0.01 (0.013, 0.021) |
| **35** | 0.01 (2.061 x 10^-9^, 1.742) | 6.06 x 10^-7^ (3.339 x 10^-7^, 8.546 x 10^-6^) | 0.02 (0.015, 0.021) | 0.02 (0.013, 0.024) | 0.01 (0.012, 0.024) | 0.01 (0.013, 0.023) |
| **40** | 0.22 (0.072, 19.922) | 2.01 x 10^-6^ (6.573 x 10^-7^, 1.295 x 10^-4^) | 0.02 (0.015, 0.019) | 0.02 (0.017, 0.022) | 0.01 (0.008, 0.014) | 0.01 (0.013, 0.017) |
| **45** | 0.51 (0.134, 19.940) | 3.79 x 10^-6^ (7.194 x 10^-7^, 1.306 x 10^-4^) | 0.02 (0.016, 0.019) | 0.02 (0.018, 0.021) | 0.01 (0.010, 0.014) | 0.01 (0.013, 0.017) |
| **50** | 0.53 (0.153, 19.941) | 3.47 x 10^-6^ (7.457 x 10^-7^, 1.259 x 10^-4^) | 0.02 (0.016, 0.019) | 0.02 (0.018, 0.021) | 0.01 (0.010, 0.014) | 0.01 (0.013, 0.017) |
| **55** | 0.78 (0.162, 19.947) | 4.85 x 10^-6^ (7.376 x 10^-7^, 1.283 x 10^-4^) | 0.02 (0.017, 0.019) | 0.02 (0.018, 0.021) | 0.01 (0.010, 0.014) | 0.01 (0.013, 0.016) |
| **60** | 1.50 (0.241, 19.948) | 1.03 x 10^-5^ (1.259 x 10^-6^, 1.638 x 10^-4^) | 0.02 (0.017, 0.020) | 0.02 (0.018, 0.021) | 0.01 (0.011, 0.014) | 0.01 (0.013, 0.016) |
| **65** | 2.48 (0.222, 19.947) | 1.89 x 10^-5^ (1.282 x 10^-6^, 1.722 x 10^-4^) | 0.02 (0.017, 0.020) | 0.02 (0.018, 0.021) | 0.01 (0.011, 0.014) | 0.01 (0.013, 0.016) |
| **70** | 2.07 (0.225, 19.946) | 1.64 x 10^-5^ (1.362 x 10^-6^, 1.820 x 10^-4^) | 0.02 (0.017, 0.020) | 0.02 (0.018, 0.021) | 0.01 (0.011, 0.014) | 0.01 (0.013, 0.016) |
| **75** | 2.51 (0.212, 19.947) | 2.99 x 10^-5^ (1.594 x 10^-6^, 2.838 x 10^-4^) | 0.02 (0.016, 0.020) | 0.02 (0.019, 0.022) | 0.01 (0.011, 0.015) | 0.01 (0.013, 0.017) |
| **80** | 3.86 (0.244, 19.948) | 9.06 x 10^-5^ (4.315 x 10^-6^, 5.606 x 10^-4^) | 0.02 (0.016, 0.021) | 0.02 (0.021, 0.026) | 0.01 (0.011, 0.015) | 0.02 (0.014, 0.019) |
| **85** | 0.46 (0.137, 19.944) | 1.33 x 10^-5^ (2.837 x 10^-6^, 5.238 x 10^-4^) | 0.02 (0.019, 0.025) | 0.03 (0.025, 0.031) | 0.01 (0.010, 0.018) | 0.02 (0.016, 0.023) |
| **90** | 0.22 (0.102, 19.922) | 8.66 x 10^-6^ (3.230 x 10^-6^, 5.541 x 10^-4^) | 0.03 (0.025, 0.033) | 0.04 (0.030, 0.040) | 0.01 (0.008, 0.021) | 0.02 (0.021, 0.030) |
| **95** | 0.15 (0.051, 19.465) | 3.42 x 10^-6^ (1.355 x 10^-6^, 2.330 x 10^-4^) | 0.04 (0.033, 0.039) | 0.04 (0.035, 0.044) | 0.02 (0.018, 0.029) | 0.03 (0.028, 0.037) |
|  | **Fibular SMA_CC_** | | | | | |
| **5** | 0.30 (0.113, 19.796) | 9.77 x 10^-6^ (2.643 x 10^-6^, 3.464 x 10^-4^) | 0.03 (0.028, 0.035) | 0.03 (0.027, 0.037) | 0.01 (0.007, 0.020) | 0.02 (0.019, 0.028) |
| **10** | 1.32 x 10^-3^ (2.061 x 10^-9^, 0.632) | 1.27 x 10^-6^ (7.191 x 10^-7^, 8.582 x 10^-6^) | 0.02 (0.019, 0.029) | 0.02 (0.016, 0.033) | 4.16 x 10^-3^ (0.014, 0.034) | 0.01 (0.017, 0.032) |
| **15** | 2.39 x 10^-3^ (2.061 x 10^-9^, 0.765) | 5.15 x 10^-7^ (2.887 x 10^-7^, 3.924 x 10^-6^) | 0.02 (0.017, 0.023) | 0.02 (0.014, 0.025) | 6.07 x 10^-3^ (0.013, 0.026) | 0.01 (0.015, 0.025) |
| **20** | 4.52 x 10^-3^ (2.061 x 10^-9^, 0.804) | 3.57 x 10^-7^ (1.925 x 10^-7^, 2.755 x 10^-6^) | 0.02 (0.017, 0.022) | 0.02 (0.017, 0.022) | 7.24 x 10^-3^ (0.014, 0.025) | 0.01 (0.015, 0.023) |
| **25** | 0.18 (0.079, 19.930) | 1.36 x 10^-6^ (4.881 x 10^-7^, 9.728 x 10^-5^) | 0.02 (0.017, 0.020) | 0.02 (0.014, 0.019) | 9.93 x 10^-3^ (0.008, 0.014) | 0.01 (0.013, 0.018) |
| **30** | 0.14 (0.087, 19.924) | 8.92 x 10^-7^ (4.417 x 10^-7^, 7.315 x 10^-5^) | 0.02 (0.017, 0.020) | 0.02 (0.016, 0.020) | 0.01 (0.009, 0.014) | 0.01 (0.014, 0.018) |
| **35** | 0.12 (0.054, 19.670) | 8.78 x 10^-7^ (4.163 x 10^-7^, 7.635 x 10^-5^) | 0.02 (0.017, 0.020) | 0.02 (0.016, 0.021) | 0.01 (0.009, 0.015) | 0.02 (0.015, 0.019) |
| **40** | 0.15 (0.086, 19.912) | 1.08 x 10^-6^ (4.827 x 10^-7^, 9.187 x 10^-5^) | 0.02 (0.017, 0.020) | 0.02 (0.017, 0.021) | 0.01 (0.010, 0.015) | 0.02 (0.014, 0.018) |
| **45** | 0.12 (0.074, 19.900) | 8.58 x 10^-7^ (3.775 x 10^-7^, 7.914 x 10^-5^) | 0.02 (0.017, 0.020) | 0.02 (0.018, 0.022) | 0.01 (0.010, 0.016) | 0.01 (0.014, 0.018) |
| **50** | 0.18 (0.095, 19.939) | 1.06 x 10^-6^ (4.536 x 10^-7^, 8.744 x 10^-5^) | 0.02 (0.017, 0.020) | 0.02 (0.019, 0.022) | 0.01 (0.010, 0.015) | 0.02 (0.015, 0.018) |
| **55** | 0.32 (0.121, 19.935) | 1.66 x 10^-6^ (4.497 x 10^-7^, 8.102 x 10^-5^) | 0.02 (0.017, 0.020) | 0.02 (0.019, 0.022) | 0.01 (0.010, 0.015) | 0.02 (0.014, 0.017) |
| **60** | 0.42 (0.144, 19.943) | 2.10 x 10^-6^ (4.860 x 10^-7^, 8.345 x 10^-5^) | 0.02 (0.017, 0.020) | 0.02 (0.019, 0.022) | 0.01 (0.011, 0.015) | 0.02 (0.014, 0.017) |
| **65** | 0.44 (0.135, 19.937) | 2.39 x 10^-6^ (5.845 x 10^-7^, 9.073 x 10^-5^) | 0.02 (0.017, 0.020) | 0.02 (0.019, 0.021) | 0.01 (0.011, 0.015) | 0.02 (0.014, 0.018) |
| **70** | 0.63 (0.145, 19.942) | 3.61 x 10^-6^ (6.400 x 10^-7^, 1.046 x 10^-4^) | 0.02 (0.017, 0.019) | 0.02 (0.019, 0.022) | 0.01 (0.011, 0.015) | 0.02 (0.014, 0.018) |
| **75** | 1.94 (0.194, 19.947) | 1.33 x 10^-5^ (1.045 x 10^-6^, 1.451 x 10^-4^) | 0.02 (0.016, 0.019) | 0.02 (0.019, 0.022) | 0.01 (0.011, 0.014) | 0.02 (0.014, 0.017) |
| **80** | 0.88 (0.163, 19.946) | 8.12 x 10^-6^ (1.107 x 10^-6^, 1.669 x 10^-4^) | 0.02 (0.016, 0.019) | 0.02 (0.020, 0.023) | 0.01 (0.010, 0.014) | 0.02 (0.014, 0.018) |
| **85** | 0.25 (0.113, 19.938) | 4.67 x 10^-6^ (1.382 x 10^-6^, 2.639 x 10^-4^) | 0.02 (0.016, 0.019) | 0.03 (0.022, 0.028) | 0.01 (0.009, 0.016) | 0.02 (0.014, 0.020) |
| **90** | 2.89 (0.235, 19.948) | 1.25 x 10^-4^ (8.288 x 10^-6^, 1.121 x 10^-3^) | 0.03 (0.024, 0.031) | 0.03 (0.030, 0.037) | 0.02 (0.012, 0.019) | 0.02 (0.021, 0.028) |
| **95** | 0.77 (0.161, 19.944) | 3.40 x 10^-5^ (5.687 x 10^-6^, 9.079 x 10^-4^) | 0.04 (0.033, 0.041) | 0.05 (0.044, 0.052) | 0.02 (0.020, 0.029) | 0.03 (0.032, 0.040) |

**Table A4**. Parameter output for a multi-rate Brownian motion model with four means corresponding to fossorial, natatorial, scansorial, and generalist mustelids. σ^2^ _Fos_, σ^2^ _Nat_, σ^2^ _Sca_. σ^2^ _Gen_ denote rates of stochastic evolution for fossorial, natatorial, scansorial, and generalist mustelids, respectively. θ_0_ represents the starting state of trait values. Parameter estimates are followed by 95% confidence limits in parentheses. Note that the bootstrapping routine does not provide confidence intervals for group means (not shown).

| **%** | **σ^2^ _Fos_** | **σ^2^ _Nat_** | **σ^2^ _Sca_** | **σ^2^ _Gen_** | **θ_0_** |
| --- | --- | --- | --- | --- | --- |
|  | **Femoral CSA** | | | | |
| **5** | 6.62 x 10^-6^ (8.966 x 10^-7^, 1.417 x 10^-3^) | 3.68 x 10^-5^ (9.782 x 10^-6^, 1.182 x 10^-4^) | 8.93 x 10^-6^ (2.323 x 10^-6^, 1.182 x 10^-5^) | 1.06 x 10^-5^ (1.590 x 10^-6^, 2.232 x 10^-5^) | 0.10 (0.086, 0.109) |
| **10** | 1.10 x 10^-5^ (1.881 x 10^-6^, 2.867 x 10^-5^) | 6.32 x 10^-5^ (1.867 x 10^-5^, 2.225 x 10^-4^) | 8.00 x 10^-6^ (3.404 x 10^-6^, 3.780 x 10^-5^) | 3.97 x 10^-5^ (6.428 x 10^-6^, 7.952 x 10^-5^) | 0.09 (0.075, 0.106) |
| **15** | 5.28 x 10^-6^ (8.353 x 10^-7^, 1.072 x 10^-5^) | 6.60 x 10^-5^ (1.543 x 10^-5^, 1.951 x 10^-4^) | 5.41 x 10^-6^ (1.944 x 10^-6^, 2.005 x 10^-5^) | 5.11 x 10^-6^ (9.868 x 10^-7^, 1.241 x 10^-5^) | 0.08 (0.064, 0.085) |
| **20** | 3.66 x 10^-6^ (5.628 x 10^-7^, 8.749 x 10^-6^) | 4.76 x 10^-5^ (1.545 x 10^-5^, 1.730 x 10^-4^) | 4.19 x 10^-6^ (1.718 x 10^-6^, 1.912 x 10^-5^) | 1.08 x 10^-5^ (1.673 x 10^-6^, 2.334 x 10^-5^) | 0.07 (0.062, 0.081) |
| **25** | 1.03 x 10^-6^ (1.841 x 10^-7^, 2.635 x 10^-6^) | 5.78 x 10^-5^ (1.376 x 10^-5^, 1.730 x 10^-4^) | 2.09 x 10^-6^ (1.203 x 10^-6^, 1.383 x 10^-5^) | 6.78 x 10^-6^ (1.090 x 10^-6^, 1.416 x 10^-5^) | 0.07 (0.061, 0.072) |
| **30** | 6.71 x 10^-7^ (1.394 x 10^-7^, 1.894 x 10^-6^) | 7.16 x 10^-5^ (1.792 x 10^-5^, 2.030 x 10^-4^) | 1.74 x 10^-6^ (6.467 x 10^-7^, 7.316 x 10^-6^) | 3.79 x 10^-6^ (5.733 x 10^-7^, 7.800 x 10^-6^) | 0.06 (0.059, 0.068) |
| **35** | 6.61 x 10^-7^ (1.257 x 10^-7^, 1.821 x 10^-6^) | 5.31 x 10^-5^ (1.606 x 10^-5^, 1.612 x 10^-4^) | 1.65 x 10^-6^ (6.978 x 10^-7^, 7.721 x 10^-6^) | 3.35 x 10^-6^ (4.618 x 10^-7^, 6.800 x 10^-6^) | 0.06 (0.060, 0.068) |
| **40** | 1.22 x 10^-6^ (1.906 x 10^-7^, 3.241 x 10^-6^) | 4.88 x 10^-5^ (1.193 x 10^-5^, 1.564 x 10^-4^) | 1.94 x 10^-6^ (5.898 x 10^-7^, 7.330 x 10^-6^) | 3.39 x 10^-6^ (5.712 x 10^-7^, 6.572 x 10^-6^) | 0.06 (0.057, 0.069) |
| **45** | 1.21 x 10^-6^ (2.221 x 10^-7^, 3.503 x 10^-6^) | 4.42 x 10^-5^ (1.203 x 10^-5^, 1.391 x 10^-4^) | 1.54 x 10^-6^ (5.742 x 10^-7^, 6.293 x 10^-6^) | 4.51 x 10^-6^ (6.131 x 10^-7^, 8.716 x 10^-6^) | 0.06 (0.056, 0.067) |
| **50** | 8.80 x 10^-7^ (1.423 x 10^-7^, 2.236 x 10^-6^) | 4.14 x 10^-5^ (1.213 x 10^-5^, 1.346 x 10^-4^) | 1.70 x 10^-6^ (6.404 x 10^-7^, 6.803 x 10^-6^) | 3.92 x 10^-6^ (5.363 x 10^-7^, 8.072 x 10^-6^) | 0.06 (0.055, 0.064) |
| **55** | 5.04 x 10^-7^ (9.676 x 10^-8^, 1.373 x 10^-6^) | 3.89 x 10^-5^ (1.050 x 10^-5^, 1.202 x 10^-4^) | 1.88 x 10^-6^ (7.288 x 10^-7^, 8.614 x 10^-6^) | 3.37 x 10^-6^ (4.500 x 10^-7^, 6.361 x 10^-6^) | 0.06 (0.057, 0.064) |
| **60** | 5.75 x 10^-7^ (9.405 x 10^-8^, 1.592 x 10^-6^) | 3.71 x 10^-5^ (1.083 x 10^-5^, 1.175 x 10^-4^) | 2.23 x 10^-6^ (7.481 x 10^-7^, 8.983 x 10^-6^) | 2.76 x 10^-6^ (3.974 x 10^-7^, 5.299 x 10^-6^) | 0.06 (0.057, 0.064) |
| **65** | 7.91 x 10^-7^ (1.312 x 10^-7^, 1.958 x 10^-6^) | 3.99 x 10^-5^ (1.118 x 10^-5^, 1.178 x 10^-4^) | 2.75 x 10^-6^ (9.252 x 10^-7^, 9.488 x 10^-6^) | 2.16 x 10^-6^ (3.260 x 10^-7^, 4.543 x 10^-6^) | 0.06 (0.056, 0.065) |
| **70** | 9.06 x 10^-7^ (1.707 x 10^-7^, 2.093 x 10^-6^) | 5.23 x 10^-5^ (1.360 x 10^-5^, 1.490 x 10^-4^) | 3.12 x 10^-6^ (9.745 x 10^-7^, 1.054 x 10^-5^) | 1.98 x 10^-6^ (3.348 x 10^-7^, 4.242 x 10^-6^) | 0.06 (0.057, 0.065) |
| **75** | 1.47 x 10^-6^ (2.595 x 10^-7^, 3.488 x 10^-6^) | 5.80 x 10^-5^ (1.443 x 10^-5^, 1.761 x 10^-4^) | 3.81 x 10^-6^ (1.358 x 10^-6^, 1.254 x 10^-5^) | 2.25 x 10^-6^ (3.394 x 10^-7^, 5.339 x 10^-6^) | 0.06 (0.057, 0.067) |
| **80** | 2.48 x 10^-6^ (4.422 x 10^-7^, 6.067 x 10^-6^) | 7.55 x 10^-5^ (2.260 x 10^-5^, 2.333 x 10^-4^) | 4.73 x 10^-6^ (1.311 x 10^-6^, 1.647 x 10^-5^) | 3.77 x 10^-6^ (4.700 x 10^-7^, 7.968 x 10^-6^) | 0.07 (0.059, 0.074) |
| **85** | 6.27 x 10^-6^ (1.075 x 10^-6^, 1.308 x 10^-5^) | 2.41 x 10^-4^ (6.933 x 10^-5^, 7.245 x 10^-4^) | 9.73 x 10^-6^ (3.171 x 10^-6^, 3.250 x 10^-5^) | 5.95 x 10^-6^ (9.285 x 10^-7^, 1.411 x 10^-5^) | 0.08 (0.066, 0.090) |
| **90** | 2.96 x 10^-5^ (9.182 x 10^-6^, 7.087 x 10^-5^) | 9.35 x 10^-5^ (2.924 x 10^-5^, 3.296 x 10^-4^) | 1.48 x 10^-5^ (4.803 x 10^-6^, 5.242 x 10^-5^) | 3.45 x 10^-5^ (5.619 x 10^-6^, 6.912 x 10^-5^) | 0.11 (0.078, 0.135) |
| **95** | 6.64 x 10^-6^ (5.372 x 10^-7^, 1.337 x 10^-5^) | 1.62 x 10^-5^ (6.461 x 10^-6^, 6.895 x 10^-5^) | 1.70 x 10^-5^ (4.041 x 10^-6^, 4.205 x 10^-5^) | 4.76 x 10^-6^ (7.169 x 10^-7^, 1.108 x 10^-5^) | 0.10 (0.092, 0.115) |
|  | **Femoral SMA_ML_** | | | | |
| **5** | 7.37 x 10^-7^ (1.143 x 10^-7^, 1.708 x 10^-6^) | 5.53 x 10^-6^ (1.402 x 10^-6^, 1.559 x 10^-5^) | 2.74 x 10^-6^ (7.814 x 10^-7^, 9.215 x 10^-6^) | 7.03 x 10^-6^ (1.187 x 10^-6^, 1.380 x 10^-5^) | 0.06 (0.052, 0.060) |
| **10** | 1.67 x 10^-6^ (2.812 x 10^-7^, 4.361 x 10^-6^) | 1.62 x 10^-5^ (3.861 x 10^-6^, 4.561 x 10^-5^) | 3.50 x 10^-6^ (1.224 x 10^-6^, 1.443 x 10^-5^) | 2.30 x 10^-5^ (4.213 x 10^-6^, 4.766 x 10^-5^) | 0.05 (0.045, 0.058) |
| **15** | 1.22 x 10^-6^ (1.667 x 10^-7^, 2.444 x 10^-6^) | 1.07 x 10^-5^ (2.670 x 10^-6^, 3.421 x 10^-5^) | 2.28 x 10^-6^ (6.223 x 10^-7^, 6.234 x 10^-6^) | 2.33 x 10^-6^ (4.064 x 10^-7^, 5.045 x 10^-6^) | 0.04 (0.039, 0.048) |
| **20** | 1.32 x 10^-6^ (2.127 x 10^-7^, 3.025 x 10^-6^) | 9.11 x 10^-6^ (3.292 x 10^-6^, 3.976 x 10^-5^) | 1.53 x 10^-6^ (6.241 x 10^-7^, 6.991 x 10^-6^) | 5.29 x 10^-6^ (7.472 x 10^-7^, 9.817 x 10^-6^) | 0.05 (0.039, 0.049) |
| **25** | 9.29 x 10^-7^ (1.637 x 10^-7^, 2.622 x 10^-6^) | 1.33 x 10^-5^ (4.550 x 10^-6^, 4.440 x 10^-5^) | 1.10 x 10^-6^ (4.406 x 10^-7^, 4.880 x 10^-6^) | 4.06 x 10^-6^ (6.101 x 10^-7^, 7.447 x 10^-6^) | 0.04 (0.037, 0.046) |
| **30** | 8.29 x 10^-7^ (1.417 x 10^-7^, 2.252 x 10^-6^) | 1.17 x 10^-5^ (3.037 x 10^-6^, 3.803 x 10^-5^) | 8.30 x 10^-7^ (2.696 x 10^-7^, 2.319 x 10^-6^) | 3.20 x 10^-6^ (4.438 x 10^-7^, 5.687 x 10^-6^) | 0.04 (0.035, 0.044) |
| **35** | 8.71 x 10^-7^ (1.535 x 10^-7^, 2.231 x 10^-6^) | 9.08 x 10^-6^ (2.803 x 10^-6^, 3.033 x 10^-5^) | 6.59 x 10^-7^ (1.886 x 10^-7^, 2.082 x 10^-6^) | 2.56 x 10^-6^ (3.668 x 10^-7^, 4.475 x 10^-6^) | 0.04 (0.033, 0.042) |
| **40** | 7.60 x 10^-7^ (1.225 x 10^-7^, 1.745 x 10^-6^) | 9.36 x 10^-6^ (2.927 x 10^-6^, 3.147 x 10^-5^) | 6.12 x 10^-7^ (1.453 x 10^-7^, 1.896 x 10^-6^) | 1.89 x 10^-6^ (2.708 x 10^-7^, 3.617 x 10^-6^) | 0.04 (0.032, 0.041) |
| **45** | 6.67 x 10^-7^ (1.112 x 10^-7^, 1.631 x 10^-6^) | 1.06 x 10^-5^ (2.970 x 10^-6^, 3.473 x 10^-5^) | 5.79 x 10^-7^ (1.545 x 10^-7^, 1.723 x 10^-6^) | 1.69 x 10^-6^ (2.258 x 10^-7^, 3.211 x 10^-6^) | 0.04 (0.032, 0.040) |
| **50** | 5.32 x 10^-7^ (9.190 x 10^-8^, 1.267 x 10^-6^) | 1.27 x 10^-5^ (3.751 x 10^-6^, 3.676 x 10^-5^) | 6.85 x 10^-7^ (1.875 x 10^-7^, 1.926 x 10^-6^) | 8.98 x 10^-7^ (1.288 x 10^-7^, 1.895 x 10^-6^) | 0.04 (0.033, 0.040) |
| **55** | 4.33 x 10^-7^ (7.520 x 10^-8^, 8.803 x 10^-7^) | 1.55 x 10^-5^ (3.893 x 10^-6^, 4.119 x 10^-5^) | 8.80 x 10^-7^ (2.749 x 10^-7^, 2.771 x 10^-6^) | 5.79 x 10^-7^ (1.070 x 10^-7^, 1.346 x 10^-6^) | 0.04 (0.034, 0.040) |
| **60** | 4.97 x 10^-7^ (8.573 x 10^-8^, 1.079 x 10^-6^) | 1.66 x 10^-5^ (4.004 x 10^-6^, 4.448 x 10^-5^) | 1.19 x 10^-6^ (3.682 x 10^-7^, 3.377 x 10^-6^) | 4.25 x 10^-7^ (7.835 x 10^-8^, 1.283 x 10^-6^) | 0.04 (0.034, 0.040) |
| **65** | 3.97 x 10^-7^ (5.268 x 10^-8^, 8.462 x 10^-7^) | 1.70 x 10^-5^ (4.017 x 10^-6^, 4.565 x 10^-5^) | 1.57 x 10^-6^ (4.776 x 10^-7^, 4. 770 x 10^-6^) | 6.82 x 10^-7^ (1.293 x 10^-7^, 1.793 x 10^-6^) | 0.04 (0.035, 0.040) |
| **70** | 5.07 x 10^-7^ (7.167 x 10^-8^, 1.128 x 10^-6^) | 2.10 x 10^-5^ (4.756 x 10^-6^, 5.907 x 10^-5^) | 1.57 x 10^-6^ (4.980 x 10^-7^, 5.421 x 10^-6^) | 1.21 x 10^-6^ (2.168 x 10^-7^, 2.931 x 10^-6^) | 0.04 (0.036, 0.040) |
| **75** | 5.36 x 10^-7^ (7.864 x 10^-8^, 1.200 x 10^-6^) | 2.30 x 10^-5^ (4.405 x 10^-6^, 6.328 x 10^-5^) | 1.74 x 10^-6^ (5.704 x 10^-7^, 6.188 x 10^-6^) | 2.01 x 10^-6^ (2.934 x 10^-7^, 4.356 x 10^-6^) | 0.04 (0.038, 0.044) |
| **80** | 1.57 x 10^-6^ (2.685 x 10^-7^, 3.487 x 10^-6^) | 3.06 x 10^-5^ (7.110 x 10^-6^, 9.295 x 10^-5^) | 2.24 x 10^-6^ (6.519 x 10^-7^, 7.524 x 10^-6^) | 3.65 x 10^-6^ (4.854 x 10^-7^, 7.504 x 10^-6^) | 0.05 (0.038, 0.051) |
| **85** | 1.99 x 10^-6^ (3.656 x 10^-7^, 4.806 x 10^-6^) | 1.14 x 10^-4^ (2.503 x 10^-5^, 3.104 x 10^-4^) | 2.88 x 10^-6^ (1.124 x 10^-6^, 1.239 x 10^-5^) | 1.00 x 10^-5^ (1.439 x 10^-6^, 1.896 x 10^-5^) | 0.05 (0.046, 0.060) |
| **90** | 4.00 x 10^-6^ (6.680 x 10^-7^, 9.335 x 10^-6^) | 3.21 x 10^-5^ (1.082 x 10^-5^, 1.162 x 10^-4^) | 6.23 x 10^-6^ (2.955 x 10^-6^, 3.081 x 10^-5^) | 1.04 x 10^-5^ (1.743 x 10^-6^, 2.025 x 10^-5^) | 0.08 (0.066, 0.084) |
| **95** | 1.17 x 10^-6^ (1.487 x 10^-7^, 2.414 x 10^-6^) | 7.66 x 10^-6^ (2.888 x 10^-6^, 3.134 x 10^-5^) | 2.70 x 10^-6^ (8.457 x 10^-7^, 8.120 x 10^-6^) | 5.22 x 10^-7^ (9.334 x 10^-8^, 1.495 x 10^-6^) | 0.07 (0.064, 0.074) |
|  | **Femoral SMA_CC_** | | | | |
| **5** | 2.86 x 10^-6^ (4.789 x 10^-7^, 6.754 x 10^-6^) | 3.13 x 10^-5^ (8.254 x 10^-6^, 9.517 x 10^-5^) | 2.75 x 10^-6^ (9.321 x 10^-7^, 1.020 x 10^-5^) | 1.10 x 10^-5^ (1.664 x 10^-6^, 2.259 x 10^-5^) | 0.08 (0.074, 0.089) |
| **10** | 7.20 x 10^-6^ (1.399 x 10^-6^, 2.517 x 10^-5^) | 4.01 x 10^-5^ (1.216 x 10^-5^, 1.303 x 10^-4^) | 3.32 x 10^-6^ (1.719 x 10^-6^, 2.513 x 10^-5^) | 5.03 x 10^-5^ (8.168 x 10^-6^, 9.994 x 10^-5^) | 0.08 (0.064, 0.094) |
| **15** | 2.94 x 10^-6^ (4.918 x 10^-6^, 7.820 x 10^-5^) | 6.04 x 10^-5^ (1.649 x 10^-5^, 1.609 x 10^-4^) | 2.50 x 10^-6^ (1.055 x 10^-6^, 1.227 x 10^-5^) | 7.78 x 10^-6^ (1.236 x 10^-6^, 1.896 x 10^-5^) | 0.06 (0.051, 0.068) |
| **20** | 2.25 x 10^-6^ (4.766 x 10^-7^, 6.404 x 10^-6^) | 2.75 x 10^-5^ (7.817 x 10^-6^, 8.923 x 10^-5^) | 2.09 x 10^-6^ (8.083 x 10^-7^, 1.089 x 10^-5^) | 6.33 x 10^-6^ (9.755 x 10^-7^, 1.334 x 10^-5^) | 0.06 (0.047, 0.062) |
| **25** | 9.64 x 10^-7^ (2.088 x 10^-7^, 2.695 x 10^-6^) | 3.81 x 10^-5^ (8.356 x 10^-6^, 1.062 x 10^-4^) | 1.17 x 10^-6^ (6.258 x 10^-7^, 6.298 x 10^-6^) | 2.46 x 10^-6^ (3.525 x 10^-7^, 5.697 x 10^-6^) | 0.05 (0.042, 0.053) |
| **30** | 4.93 x 10^-7^ (1.040 x 10^-7^, 1.540 x 10^-6^) | 3.24 x 10^-5^ (7.600 x 10^-6^, 8.831 x 10^-5^) | 7.85 x 10^-7^ (3.447 x 10^-7^, 3.968 x 10^-6^) | 1.09 x 10^-6^ (1.747 x 10^-7^, 2.584 x 10^-6^) | 0.04 (0.040, 0.047) |
| **35** | 3.44 x 10^-7^ (5.974 x 10^-8^, 8.900 x 10^-7^) | 1.93 x 10^-5^ (4.820 x 10^-6^, 5.781 x 10^-5^) | 5.34 x 10^-7^ (2.546 x 10^-7^, 3.051 x 10^-6^) | 8.33 x 10^-7^ (1.099 x 10^-7^, 1.801 x 10^-6^) | 0.04 (0.037, 0.043) |
| **40** | 3.93 x 10^-7^ (7.604 x 10^-8^, 1.017 x 10^-6^) | 1.61 x 10^-5^ (3.934 x 10^-6^, 4.741 x 10^-5^) | 4.48 x 10^-7^ (2.033 x 10^-7^, 2.081 x 10^-6^) | 9.29 x 10^-7^ (1.517 x 10^-7^, 2.009 x 10^-6^) | 0.04 (0.037, 0.042) |
| **45** | 3.15 x 10^-7^ (5.819 x 10^-8^, 8.525 x 10^-7^) | 1.51 x 10^-5^ (4.113 x 10^-6^, 4.450 x 10^-5^) | 3.78 x 10^-7^ (1.550 x 10^-7^, 1.892 x 10^-6^) | 1.09 x 10^-6^ (1.357 x 10^-7^, 2.087 x 10^-6^) | 0.04 (0.036, 0.041) |
| **50** | 2.74 x 10^-7^ (4.859 x 10^-8^, 6.559 x 10^-7^) | 1.61 x 10^-5^ (5.214 x 10^-6^, 4.569 x 10^-5^) | 4.43 x 10^-7^ (1.916 x 10^-7^, 2.161 x 10^-6^) | 9.91 x 10^-7^ (1.309 x 10^-7^, 1.856 x 10^-6^) | 0.04 (0.036, 0.041) |
| **55** | 2.74 x 10^-7^ (5.835 x 10^-8^, 8.150 x 10^-7^) | 1.78 x 10^-5^ (4.480 x 10^-6^, 5.145 x 10^-5^) | 6.55 x 10^-7^ (2.333 x 10^-7^, 2.637 x 10^-6^) | 1.13 x 10^-6^ (1.698 x 10^-7^, 2.112 x 10^-6^) | 0.04 (0.036, 0.042) |
| **60** | 3.51 x 10^-7^ (8.057 x 10^-8^, 1.069 x 10^-6^) | 2.19 x 10^-5^ (5.068 x 10^-6^, 6.537 x 10^-5^) | 1.14 x 10^-6^ (3.439 x 10^-7^, 4.211 x 10^-6^) | 1.14 x 10^-6^ (1.638 x 10^-7^, 2.302 x 10^-6^) | 0.04 (0.037, 0.044) |
| **65** | 6.28 x 10^-7^ (1.016 x 10^-7^, 1.570 x 10^-6^) | 2.75 x 10^-5^ (6.029 x 10^-6^, 7.139 x 10^-5^) | 1.78 x 10^-6^ (4.686 x 10^-7^, 5.376 x 10^-6^) | 1.18 x 10^-6^ (2.085 x 10^-7^, 2.624 x 10^-6^) | 0.04 (0.038, 0.045) |
| **70** | 8.66 x 10^-7^ (1.682 x 10^-7^, 1.984 x 10^-6^) | 3.70 x 10^-5^ (9.501 x 10^-6^, 9.448 x 10^-5^) | 2.30 x 10^-6^ (4.948 x 10^-7^, 7.222 x 10^-6^) | 1.44 x 10^-6^ (2.247 x 10^-7^, 3.224 x 10^-6^) | 0.04 (0.039, 0.048) |
| **75** | 1.01 x 10^-6^ (1.827 x 10^-7^, 2.497 x 10^-6^) | 3.96 x 10^-5^ (9.583 x 10^-6^, 1.085 x 10^-4^) | 3.16 x 10^-6^ (9.220 x 10^-7^, 1.027 x 10^-5^) | 1.54 x 10^-6^ (2.717 x 10^-7^, 3.871 x 10^-6^) | 0.05 (0.041, 0.051) |
| **80** | 1.71 x 10^-6^ (3.332 x 10^-7^, 4.237 x 10^-6^) | 4.27 x 10^-5^ (1.052 x 10^-5^, 1.267 x 10^-4^) | 4.28 x 10^-6^ (1.340 x 10^-6^, 1.445 x 10^-5^) | 3.02 x 10^-6^ (4.978 x 10^-7^, 7.521 x 10^-6^) | 0.05 (0.045, 0.058) |
| **85** | 2.03 x 10^-6^ (3.004 x 10^-7^, 4.931 x 10^-6^) | 1.01 x 10^-4^ (2.965 x 10^-5^, 3.089 x 10^-4^) | 6.62 x 10^-6^ (2.593 x 10^-6^, 2.581 x 10^-5^) | 7.34 x 10^-6^ (1.132 x 10^-6^, 1.545 x 10^-5^) | 0.06 (0.055, 0.070) |
| **90** | 3.13 x 10^-6^ (5.280 x 10^-7^, 7.213 x 10^-6^) | 3.00 x 10^-5^ (1.007 x 10^-5^, 1.076 x 10^-4^) | 6.61 x 10^-6^ (3.947 x 10^-6^, 3.924 x 10^-5^) | 2.23 x 10^-5^ (3.544 x 10^-6^, 4.246 x 10^-5^) | 0.08 (0.074, 0.091) |
| **95** | 1.22 x 10^-6^ (2.104 x 10^-7^, 2.722 x 10^-6^) | 1.13 x 10^-5^ (3.521 x 10^-6^, 4.116 x 10^-5^) | 4.80 x 10^-6^ (1.333 x 10^-6^, 1.372 x 10^-5^) | 2.68 x 10^-6^ (3.863 x 10^-7^, 5.871 x 10^-6^) | 0.08 (0.074, 0.084) |
|  | **Tibial CSA** | | | | |
| **5** | 5.34 x 10^-5^ (7.355 x 10^-6^, 1.034 x 10^-4^) | 1.46 x 10^-5^ (6.541 x 10^-6^, 6.832 x 10^-5^) | 2.03 x 10^-5^ (6.495 x 10^-6^, 6.066 x 10^-5^) | 5.77 x 10^-6^ (1.268 x 10^-6^ 1.774 x 10^-5^) | 0.12 (0.078, 0.141) |
| **10** | 3.23 x 10^-5^ (4.473 x 10^-6^, 7.781 x 10^-5^) | 2.54 x 10^-5^ (6.227 x 10^-6^, 7.783 x 10^-5^) | 2.23 x 10^-5^ (7.889 x 10^-6^, 8.302 x 10^-5^) | 1.70 x 10^-5^ (3.309 x 10^-6^, 4.626 x 10^-5^) | 0.11 (0.078, 0.130) |
| **15** | 1.08 x 10^-5^ (1.931 x 10^-6^, 2.490 x 10^-5^) | 1.03 x 10^-5^ (2.797 x 10^-6^, 3.013 x 10^-5^) | 8.11 x 10^-6^ (2.564 x 10^-6^, 2.833 x 10^-5^) | 5.31 x 10^-6^ (9.687 x 10^-7^, 1.480 x 10^-5^) | 0.09 (0.064, 0.096) |
| **20** | 5.69 x 10^-6^ (9.775 x 10^-7^, 1.311 x 10^-5^) | 8.91 x 10^-6^ (2.109 x 10^-6^, 2.304 x 10^-5^) | 5.17 x 10^-6^ (1.881 x 10^-6^, 2.130 x 10^-5^) | 7.29 x 10^-6^ (1.422 x 10^-6^, 1.867 x 10^-5^) | 0.08 (0.063, 0.086) |
| **25** | 4.45 x 10^-6^ (7.321 x 10^-7^, 9.883 x 10^-6^) | 6.45 x 10^-6^ (1.804 x 10^-6^, 1.630 x 10^-5^) | 5.04 x 10^-6^ (1.835 x 10^-6^, 2.087 x 10^-5^) | 5.31 x 10^-6^ (1.028 x 10^-6^, 1.429 x 10^-5^) | 0.07 (0.062, 0.081) |
| **30** | 3.26 x 10^-6^ (6.274 x 10^-7^, 8.834 x 10^-6^) | 6.97 x 10^-6^ (1.797 x 10^-6^, 1.969 x 10^-5^) | 4.12 x 10^-6^ (1.567 x 10^-6^, 1.631 x 10^-5^) | 4.09 x 10^-6^ (7.617 x 10^-7^, 1.150 x 10^-5^) | 0.07 (0.061, 0.079) |
| **35** | 3.03 x 10^-6^ (4.398 x 10^-7^, 8.567 x 10^-6^) | 7.47 x 10^-6^ (1.802 x 10^-6^, 1.864 x 10^-5^) | 4.00 x 10^-6^ (1.476 x 10^-6^, 1.681 x 10^-5^) | 5.25 x 10^-6^ (1.103 x 10^-6^, 1.559 x 10^-5^) | 0.07 (0.060, 0.078) |
| **40** | 3.23 x 10^-6^ (5.700 x 10^-7^, 9.286 x 10^-6^) | 8.27 x 10^-6^ (2.083 x 10^-6^, 2.221 x 10^-5^) | 3.51 x 10^-6^ (1.183 x 10^-6^, 1.454 x 10^-5^) | 5.17 x 10^-6^ (9.908 x 10^-7^, 1.351 x 10^-5^) | 0.07 (0.059, 0.077) |
| **45** | 3.74 x 10^-6^ (8.406 x 10^-7^, 1.047 x 10^-5^) | 9.81 x 10^-6^ (2.567 x 10^-6^, 2.787 x 10^-5^) | 3.58 x 10^-6^ (1.260 x 10^-6^, 1.483 x 10^-5^) | 5.20 x 10^-6^ (1.024 x 10^-6^, 1.381 x 10^-5^) | 0.07 (0.057, 0.077) |
| **50** | 3.90 x 10^-6^ (7.906 x 10^-7^, 1.116 x 10^-5^) | 1.16 x 10^-5^ (3.161 x 10^-6^, 3.745 x 10^-5^) | 3.39 x 10^-6^ (1.310 x 10^-6^, 1.246 x 10^-5^) | 5.02 x 10^-6^ (1.010 x 10^-6^, 1.229 x 10^-5^) | 0.07 (0.055, 0.077) |
| **55** | 4.00 x 10^-6^ (6.900 x 10^-7^, 1.110 x 10^-5^) | 1.27 x 10^-5^ (3.349 x 10^-6^, 3.808 x 10^-5^) | 3.10 x 10^-6^ (1.152 x 10^-6^, 1.260 x 10^-5^) | 5.86 x 10^-6^ (1.098 x 10^-6^, 1.293 x 10^-5^) | 0.07 (0.055, 0.075) |
| **60** | 3.39 x 10^-6^ (6.968 x 10^-7^, 9.250 x 10^-6^) | 1.26 x 10^-5^ (3.243 x 10^-6^, 3.567 x 10^-5^) | 3.76 x 10^-6^ (1.102 x 10^-6^, 1.349 x 10^-5^) | 5.47 x 10^-6^ (1.022 x 10^-6^, 1.297 x 10^-5^) | 0.07 (0.055, 0.074) |
| **65** | 3.18 x 10^-6^ (5.809 x 10^-7^, 8.421 x 10^-6^) | 9.93 x 10^-6^ (2.881 x 10^-6^, 3.250 x 10^-5^) | 4.28 x 10^-6^ (1.406 x 10^-6^, 1.525 x 10^-5^) | 3.67 x 10^-6^ (5.777 x 10^-7^, 9.911 x 10^-6^) | 0.07 (0.054, 0.071) |
| **70** | 3.05 x 10^-6^ (5.472 x 10^-7^, 7.899 x 10^-6^) | 7.00 x 10^-6^ (2.124 x 10^-6^, 2.327 x 10^-5^) | 4.29 x 10^-6^ (1.533 x 10^-6^, 1.510 x 10^-5^) | 3.11 x 10^-6^ (5.303 x 10^-7^, 8.024 x 10^-6^) | 0.07 (0.053, 0.071) |
| **75** | 3.52 x 10^-6^ (6.047 x 10^-7^, 8.543 x 10^-6^) | 6.89 x 10^-6^ (2.249 x 10^-6^, 2.520 x 10^-5^) | 5.69 x 10^-6^ (1.718 x 10^-6^, 1.928 x 10^-5^) | 2.90 x 10^-6^ (4.711 x 10^-7^, 8.131 x 10^-6^) | 0.07 (0.053, 0.071) |
| **80** | 3.68 x 10^-6^ (6.625 x 10^-7^, 9.831 x 10^-6^) | 8.79 x 10^-6^ (3.317 x 10^-6^, 3.258 x 10^-5^) | 6.85 x 10^-6^ (1.911 x 10^-6^, 2.293 x 10^-5^) | 2.98 x 10^-6^ (5.744 x 10^-7^, 7.963 x 10^-6^) | 0.07 (0.054, 0.074) |
| **85** | 6.50 x 10^-6^ (1.168 x 10^-6^, 1.552 x 10^-5^) | 1.88 x 10^-5^ (5.618 x 10^-6^, 6.760 x 10^-5^) | 8.12 x 10^-6^ (3.075 x 10^-6^, 3.086 x 10^-5^) | 3.85 x 10^-6^ (7.024 x 10^-7^, 9.533 x 10^-6^) | 0.07 (0.057, 0.081) |
| **90** | 1.52 x 10^-5^ (2.886 x 10^-6^, 3.389 x 10^-5^) | 2.10 x 10^-5^ (8.173 x 10^-6^, 8.491 x 10^-5^) | 1.64 x 10^-5^ (5.758 x 10^-6^, 6.017 x 10^-5^) | 9.71 x 10^-6^ (1.662 x 10^-6^, 2.444 x 10^-5^) | 0.09 (0.065, 0.099) |
| **95** | 5.38 x 10^-5^ (7.402 x 10^-6^, 1.037 x 10^-4^) | 8.93 x 10^-6^ (1.669 x 10^-6^, 2.033 x 10^-5^) | 2.03 x 10^-5^ (4.187 x 10^-6^, 4.740 x 10^-5^) | 3.42 x 10^-5^ (4.194 x 10^-6^, 6.760 x 10^-5^) | 0.07 (0.040, 0.101) |
|  | **Tibial SMA_ML_** | | | | |
| **5** | 3.10 x 10^-6^ (5.287 x 10^-7^, 6.136 x 10^-6^) | 2.92 x 10^-6^ (6.451 x 10^-7^, 8.147 x 10^-6^) | 5.17 x 10^-6^ (1.668 x 10^-6^, 1.737 x 10^-5^) | 2.16 x 10^-6^ (4.988 x 10^-7^, 7.484 x 10^-6^) | 0.08 (0.069, 0.085) |
| **10** | 1.20 x 10^-5^ (2.137 x 10^-6^, 3.038 x 10^-5^) | 9.93 x 10^-6^ (1.755 x 10^-6^, 2.206 x 10^-5^) | 9.75 x 10^-6^ (3.255 x 10^-6^, 3.990 x 10^-5^) | 2.16 x 10^-5^ (3.144 x 10^-6^, 4.223 x 10^-5^) | 0.08 (0.060, 0.096) |
| **15** | 6.50 x 10^-6^ (1.331 x 10^-6^, 1.759 x 10^-5^) | 4.50 x 10^-6^ (9.212 x 10^-7^, 1.003 x 10^-5^) | 3.84 x 10^-6^ (1.154 x 10^-6^, 1.420 x 10^-5^) | 6.40 x 10^-6^ (1.158 x 10^-6^, 1.538 x 10^-5^) | 0.07 (0.049, 0.075) |
| **20** | 3.54 x 10^-6^ (8.104 x 10^-7^, 9.705 x 10^-6^) | 4.03 x 10^-6^ (7.796 x 10^-7^, 8.745 x 10^-6^) | 2.59 x 10^-6^ (8.557 x 10^-7^, 9.731 x 10^-6^) | 5.31 x 10^-6^ (9.131 x 10^-7^, 1.247 x 10^-5^) | 0.06 (0.047, 0.066) |
| **25** | 2.61 x 10^-6^ (5.018 x 10^-7^, 7.396 x 10^-6^) | 3.87 x 10^-6^ (8.608 x 10^-7^, 8.726 x 10^-6^) | 2.21 x 10^-6^ (7.868 x 10^-7^, 8.577 x 10^-6^) | 3.36 x 10^-6^ (4.746 x 10^-7^, 7.592 x 10^-6^) | 0.06 (0.044, 0.062) |
| **30** | 2.19 x 10^-6^ (5.055 x 10^-7^, 6.985 x 10^-6^) | 3.22 x 10^-6^ (7.000 x 10^-7^, 7.565 x 10^-6^) | 1.84 x 10^-6^ (6.804 x 10^-7^, 6.631 x 10^-6^) | 2.11 x 10^-6^ (3.275 x 10^-7^, 5.074 x 10^-6^) | 0.05 (0.041, 0.058) |
| **35** | 2.11 x 10^-6^ (4.281 x 10^-7^, 5.880 x 10^-6^) | 2.36 x 10^-6^ (4.755 x 10^-7^, 5.593 x 10^-6^) | 1.44 x 10^-6^ (5.249 x 10^-7^, 5.999 x 10^-6^) | 3.03 x 10^-6^ (5.556 x 10^-7^, 7.034 x 10^-6^) | 0.05 (0.040, 0.056) |
| **40** | 2.42 x 10^-6^ (4.996 x 10^-7^, 6.500 x 10^-6^) | 2.05 x 10^-6^ (5.040 x 10^-7^, 4.982 x 10^-6^) | 1.25 x 10^-6^ (4.092 x 10^-7^, 5.466 x 10^-6^) | 3.22 x 10^-6^ (6.286 x 10^-7^, 7.314 x 10^-6^) | 0.05 (0.038, 0.054) |
| **45** | 2.52 x 10^-6^ (4.829 x 10^-7^, 6.708 x 10^-6^) | 1.91 x 10^-6^ (4.860 x 10^-7^, 4.808 x 10^-6^) | 1.21 x 10^-6^ (3.790 x 10^-7^, 4.390 x 10^-6^) | 3.25 x 10^-6^ (5.595 x 10^-7^, 8.038 x 10^-6^) | 0.05 (0.036, 0.052) |
| **50** | 2.20 x 10^-6^ (3.984 x 10^-7^, 5.499 x 10^-6^) | 2.44 x 10^-6^ (6.141 x 10^-7^, 6.086 x 10^-6^) | 1.20 x 10^-6^ (4.234 x 10^-7^, 4.566 x 10^-6^) | 3.30 x 10^-6^ (6.131 x 10^-7^, 7.692 x 10^-6^) | 0.04 (0.035, 0.050) |
| **55** | 1.92 x 10^-6^ (3.488 x 10^-7^, 4.831 x 10^-6^) | 3.16 x 10^-6^ (7.859 x 10^-7^, 7.785 x 10^-6^) | 1.09 x 10^-6^ (3.292 x 10^-7^, 4.291 x 10^-6^) | 3.28 x 10^-6^ (5.862 x 10^-7^, 7.196 x 10^-6^) | 0.04 (0.034, 0.048) |
| **60** | 1.39 x 10^-6^ (2.761 x 10^-7^, 3.468 x 10^-6^) | 3.36 x 10^-6^ (7.019 x 10^-7^, 8.304 x 10^-6^) | 1.27 x 10^-6^ (4.022 x 10^-7^, 4.421 x 10^-6^) | 3.08 x 10^-6^ (5.350 x 10^-7^, 6.672 x 10^-6^) | 0.04 (0.034, 0.046) |
| **65** | 1.13 x 10^-6^ (2.014 x 10^-7^, 2.822 x 10^-6^) | 3.04 x 10^-6^ (6.282 x 10^-7^, 7.032 x 10^-6^) | 1.52 x 10^-6^ (4.863 x 10^-7^, 5.123 x 10^-6^) | 2.58 x 10^-6^ (4.271 x 10^-7^, 5.526 x 10^-6^) | 0.04 (0.034, 0.045) |
| **70** | 9.71 x 10^-7^ (1.721 x 10^-7^, 2.306 x 10^-6^) | 2.63 x 10^-6^ (5.583 x 10^-7^, 6.552 x 10^-6^) | 1.89 x 10^-6^ (5.428 x 10^-7^, 6.338 x 10^-6^) | 1.94 x 10^-6^ (3.485 x 10^-7^, 4.489 x 10^-6^) | 0.04 (0.034, 0.044) |
| **75** | 9.42 x 10^-7^ (1.545 x 10^-7^, 2.064 x 10^-6^) | 2.60 x 10^-6^ (5.691 x 10^-7^, 6.257 x 10^-6^) | 2.78 x 10^-6^ (7.317 x 10^-7^, 8.213 x 10^-6^) | 1.70 x 10^-6^ (4.288 x 10^-7^, 5.017 x 10^-6^) | 0.04 (0.035, 0.043) |
| **80** | 1.11 x 10^-6^ (2.054 x 10^-7^, 2.674 x 10^-6^) | 3.44 x 10^-6^ (7.695 x 10^-7^, 9.129 x 10^-6^) | 3.52 x 10^-6^ (9.993 x 10^-7^, 1.071 x 10^-5^) | 1.49 x 10^-6^ (6.637 x 10^-7^, 4.989 x 10^-6^) | 0.04 (0.035, 0.045) |
| **85** | 1.97 x 10^-6^ (3.824 x 10^-7^, 5.469 x 10^-6^) | 8.09 x 10^-6^ (2.303 x 10^-6^, 2.514 x 10^-5^) | 4.50 x 10^-6^ (1.467 x 10^-6^, 1.468 x 10^-5^) | 1.86 x 10^-6^ (3.251 x 10^-7^, 5.036 x 10^-6^) | 0.05 (0.036, 0.052) |
| **90** | 3.74 x 10^-6^ (6.557 x 10^-7^, 9.282 x 10^-6^) | 7.97 x 10^-6^ (2.527 x 10^-6^, 2.784 x 10^-5^) | 6.11 x 10^-6^ (1.801 x 10^-6^, 2.083 x 10^-5^) | 3.91 x 10^-6^ (7.890 x 10^-7^, 1.078 x 10^-5^) | 0.06 (0.045, 0.064) |
| **95** | 8.93 x 10^-6^ (1.217 x 10^-6^, 1.727 x 10^-5^) | 3.92 x 10^-6^ (7.150 x 10^-7^, 8.069 x 10^-6^) | 4.32 x 10^-6^ (9.770 x 10^-7^, 1.143 x 10^-5^) | 6.85 x 10^-6^ (8.882 x 10^-7^, 1.358 x 10^-5^) | 0.05 (0.036, 0.061) |
|  | **Tibial SMA_CC_** | | | | |
| **5** | 6.84 x 10^-6^ (1.084 x 10^-6^, 1.587 x 10^-5^) | 8.08 x 10^-6^ (2.019 x 10^-6^, 2.165 x 10^-5^) | 6.90 x 10^-6^ (2.401 x 10^-6^, 2.867 x 10^-5^) | 7.45 x 10^-6^ (1.450 x 10^-6^, 2.195 x 10^-5^) | 0.09 (0.076, 0.100) |
| **10** | 1.75 x 10^-5^ (2.598 x 10^-6^, 4.536 x 10^-5^) | 1.08 x 10^-5^ (2.035 x 10^-6^, 2.617 x 10^-5^) | 8.62 x 10^-6^ (3.646 x 10^-6^, 3.828 x 10^-5^) | 1.82 x 10^-5^ (2.975 x 10^-6^, 4.374 x 10^-5^) | 0.08 (0.058, 0.098) |
| **15** | 5.38 x 10^-6^ (9.315 x 10^-7^, 1.474 x 10^-5^) | 4.43 x 10^-6^ (1.038 x 10^-6^, 1.157 x 10^-5^) | 3.77 x 10^-6^ (1.301 x 10^-6^, 1.575 x 10^-5^) | 5.33 x 10^-6^ (1.127 x 10^-6^, 1.393 x 10^-5^) | 0.06 (0.043, 0.067) |
| **20** | 2.12 x 10^-6^ (3.422 x 10^-7^, 5.865 x 10^-6^) | 4.43 x 10^-6^ (9.943 x 10^-7^, 1.102 x 10^-5^) | 2.75 x 10^-6^ (9.168 x 10^-7^, 1.067 x 10^-5^) | 3.53 x 10^-6^ (6.507 x 10^-7^, 9.351 x 10^-6^) | 0.05 (0.039, 0.054) |
| **25** | 1.80 x 10^-6^ (2.940 x 10^-7^, 4.824 x 10^-6^) | 4.01 x 10^-6^ (9.498 x 10^-7^, 9.822 x 10^-6^) | 2.18 x 10^-6^ (6.382 x 10^-7^, 7.676 x 10^-6^) | 1.85 x 10^-6^ (3.604 x 10^-7^, 5.059 x 10^-6^) | 0.04 (0.036, 0.049) |
| **30** | 1.22 x 10^-6^ (2.542 x 10^-7^, 3.357 x 10^-6^) | 3.87 x 10^-6^ (8.692 x 10^-7^, 9.552 x 10^-6^) | 1.61 x 10^-6^ (4.095 x 10^-7^, 5.098 x 10^-6^) | 1.19 x 10^-6^ (2.388 x 10^-7^, 3.228 x 10^-6^) | 0.04 (0.034, 0.046) |
| **35** | 8.79 x 10^-7^ (2.122 x 10^-7^, 2.547 x 10^-6^) | 3.81 x 10^-6^ (1.031 x 10^-6^, 9.766 x 10^-6^) | 1.19 x 10^-6^ (3.159 x 10^-7^, 3.708 x 10^-6^) | 8.54 x 10^-7^ (1.488 x 10^-7^, 2.295 x 10^-6^) | 0.04 (0.033, 0.043) |
| **40** | 7.45 x 10^-7^ (1.894 x 10^-7^, 2.667 x 10^-6^) | 4.58 x 10^-6^ (1.199 x 10^-6^, 1.293 x 10^-5^) | 9.05 x 10^-7^ (2.592 x 10^-7^, 2.619 x 10^-6^) | 5.04 x 10^-7^ (1.033 x 10^-7^, 1.296 x 10^-6^) | 0.04 (0.031, 0.043) |
| **45** | 7.92 x 10^-7^ (2.080 x 10^-7^, 2.664 x 10^-6^) | 4.92 x 10^-6^ (1.251 x 10^-6^, 1.352 x 10^-5^) | 8.44 x 10^-7^ (2.593 x 10^-7^, 2.459 x 10^-6^) | 4.17 x 10^-7^ (6.981 x 10^-8^, 9.537 x 10^-7^) | 0.04 (0.030, 0.040) |
| **50** | 8.30 x 10^-7^ (2.429 x 10^-7^, 2.616 x 10^-6^) | 5.29 x 10^-6^ (1.325 x 10^-6^, 1.509 x 10^-5^) | 8.31 x 10^-7^ (2.354 x 10^-7^, 2.423 x 10^-6^) | 4.92 x 10^-7^ (6.983 x 10^-8^, 1.000 x 10^-6^) | 0.04 (0.030, 0.040) |
| **55** | 8.74 x 10^-7^ (1.993 x 10^-7^, 2.950 x 10^-6^) | 5.69 x 10^-6^ (1.335 x 10^-6^, 1.720 x 10^-5^) | 8.66 x 10^-7^ (2.605 x 10^-7^, 2.894 x 10^-6^) | 7.64 x 10^-7^ (1.125 x 10^-7^, 1.733 x 10^-6^) | 0.04 (0.030, 0.040) |
| **60** | 8.83 x 10^-7^ (2.212 x 10^-7^, 2.955 x 10^-6^) | 5.83 x 10^-6^ (1.291 x 10^-6^, 1.573 x 10^-5^) | 1.05 x 10^-6^ (3.029 x 10^-7^, 3.463 x 10^-6^) | 7.67 x 10^-7^ (1.077 x 10^-7^, 1.822 x 10^-6^) | 0.04 (0.031, 0.041) |
| **65** | 1.03 x 10^-6^ (2.581 x 10^-7^, 3.071 x 10^-6^) | 5.33 x 10^-6^ (1.521 x 10^-6^, 1.508 x 10^-5^) | 1.38 x 10^-6^ (3.673 x 10^-7^, 4.623 x 10^-6^) | 8.79 x 10^-7^ (1.100 x 10^-7^, 2.059 x 10^-6^) | 0.04 (0.031, 0.043) |
| **70** | 1.24 x 10^-6^ (2.445 x 10^-7^, 3.824 x 10^-6^) | 4.05 x 10^-6^ (1.111 x 10^-6^, 1.288 x 10^-5^) | 1.82 x 10^-6^ (5.765 x 10^-7^, 6.013 x 10^-6^) | 1.12 x 10^-6^ (1.681 x 10^-7^, 2.655 x 10^-6^) | 0.04 (0.032, 0.044) |
| **75** | 1.60 x 10^-6^ (3.097 x 10^-7^, 4.825 x 10^-6^) | 4.57 x 10^-6^ (1.403 x 10^-6^, 1.471 x 10^-5^) | 2.56 x 10^-6^ (8.207 x 10^-7^, 8.239 x 10^-6^) | 1.44 x 10^-6^ (2.243 x 10^-7^, 3.601 x 10^-6^) | 0.04 (0.032, 0.046) |
| **80** | 2.04 x 10^-6^ (5.226 x 10^-7^, 6.453 x 10^-6^) | 7.02 x 10^-6^ (1.859 x 10^-6^, 2.330 x 10^-5^) | 3.44 x 10^-6^ (1.020 x 10^-6^, 1.117 x 10^-5^) | 1.44 x 10^-6^ (2.233 x 10^-7^, 3.439 x 10^-6^) | 0.05 (0.035, 0.050) |
| **85** | 3.39 x 10^-6^ (6.746 x 10^-7^, 9.271 x 10^-6^) | 1.17 x 10^-5^ (2.694 x 10^-6^, 3.472 x 10^-5^) | 4.42 x 10^-6^ (1.509 x 10^-6^, 1.652 x 10^-5^) | 2.99 x 10^-6^ (4.178 x 10^-7^, 7.386 x 10^-6^) | 0.05 (0.040, 0.058) |
| **90** | 3.80 x 10^-6^ (5.678 x 10^-7^, 8.078 x 10^-6^) | 5.44 x 10^-6^ (1.111 x 10^-6^, 1.331 x 10^-5^) | 6.14 x 10^-6^ (2.695 x 10^-6^, 3.000 x 10^-5^) | 9.25 x 10^-6^ (1.814 x 10^-6^, 2.524 x 10^-5^) | 0.06 (0.053, 0.070) |
| **95** | 2.08 x 10^-5^ (2.551 x 10^-6^, 4.226 x 10^-5^) | 1.91 x 10^-6^ (3.541 x 10^-7^, 4.877 x 10^-6^) | 4.75 x 10^-6^ (1.016 x 10^-6^, 1.170 x 10^-5^) | 9.43 x 10^-6^ (1.477 x 10^-6^, 2.278 x 10^-5^) | 0.05 (0.027, 0.065) |
|  | **Fibular CSA** | | | | |
| **5** | 1.01 x 10^-5^ (2.383 x 10^-6^, 3.486 x 10^-5^) | 2.48 x 10^-6^ (6.654 x 10^-7^, 8.767 x 10^-6^) | 2.31 x 10^-6^ (5.618 x 10^-7^, 8.917 x 10^-6^) | 3.99 x 10^-6^ (8.801 x 10^-7^, 1.187 x 10^-5^) | 0.05 (0.028, 0.065) |
| **10** | 2.83 x 10^-6^ (6.505 x 10^-7^, 9.413 x 10^-6^) | 3.28 x 10^-6^ (9.784 x 10^-7^, 9.618 x 10^-6^) | 9.23 x 10^-7^ (3.604 x 10^-7^, 4.214 x 10^-6^) | 1.33 x 10^-6^ (2.315 x 10^-7^, 3.487 x 10^-6^) | 0.04 (0.025, 0.044) |
| **15** | 2.00 x 10^-6^ (4.523 x 10^-7^, 6.832 x 10^-6^) | 1.24 x 10^-6^ (3.233 x 10^-7^, 3.581 x 10^-6^) | 6.01 x 10^-7^ (1.584 x 10^-7^, 2.353 x 10^-6^) | 9.08 x 10^-7^ (1.542 x 10^-7^, 2.279 x 10^-6^) | 0.03 (0.022, 0.038) |
| **20** | 1.32 x 10^-6^ (2.551 x 10^-7^, 4.572 x 10^-6^) | 6.43 x 10^-7^ (1.474 x 10^-7^, 1.571 x 10^-6^) | 6.01 x 10^-7^ (2.166 x 10^-7^, 2.705 x 10^-6^) | 1.19 x 10^-6^ (2.257 x 10^-7^, 2.872 x 10^-6^) | 0.03 (0.024, 0.037) |
| **25** | 9.51 x 10^-7^ (2.064 x 10^-7^, 2.853 x 10^-6^) | 3.05 x 10^-7^ (4.877 x 10^-8^, 7.258 x 10^-7^) | 6.69 x 10^-7^ (3.395 x 10^-7^, 3.265 x 10^-6^) | 1.82 x 10^-6^ (3.220 x 10^-7^, 4.318 x 10^-6^) | 0.03 (0.026, 0.036) |
| **30** | 1.04 x 10^-6^ (2.511 x 10^-7^, 3.585 x 10^-6^) | 6.67 x 10^-7^ (1.580 x 10^-7^, 1.792 x 10^-6^) | 5.13 x 10^-7^ (2.079 x 10^-7^, 2.089 x 10^-6^) | 1.28 x 10^-6^ (2.321 x 10^-7^, 3.341 x 10^-6^) | 0.03 (0.026, 0.037) |
| **35** | 1.00 x 10^-6^ (2.217 x 10^-7^, 3.324 x 10^-6^) | 1.29 x 10^-6^ (3.134 x 10^-7^, 3.629 x 10^-6^) | 5.22 x 10^-7^ (1.819 x 10^-7^, 2.311 x 10^-6^) | 1.23 x 10^-6^ (2.024 x 10^-7^, 2.885 x 10^-6^) | 0.03 (0.026, 0.037) |
| **40** | 1.15 x 10^-6^ (2.525 x 10^-7^, 3.646 x 10^-6^) | 1.38 x 10^-6^ (4.282 x 10^-7^, 4.488 x 10^-6^) | 5.89 x 10^-7^ (1.783 x 10^-7^, 2.469 x 10^-6^) | 1.28 x 10^-6^ (2.391 x 10^-7^, 2.016 x 10^-6^) | 0.03 (0.026, 0.037) |
| **45** | 1.09 x 10^-6^ (2.548 x 10^-7^, 3.149 x 10^-6^) | 1.35 x 10^-6^ (4.757 x 10^-7^, 4.253 x 10^-6^) | 5.57 x 10^-7^ (1.682 x 10^-7^, 2.066 x 10^-6^) | 8.35 x 10^-7^ (1.370 x 10^-7^, 1.924 x 10^-6^) | 0.03 (0.026, 0.037) |
| **50** | 8.16 x 10^-7^ (1.820 x 10^-7^, 2.222 x 10^-6^) | 1.28 x 10^-6^ (4.264 x 10^-7^, 4.254 x 10^-6^) | 6.53 x 10^-7^ (2.396 x 10^-7^, 2.881 x 10^-6^) | 8.20 x 10^-7^ (1.383 x 10^-7^, 1.996 x 10^-6^) | 0.03 (0.027, 0.037) |
| **55** | 5.76 x 10^-7^ (1.391 x 10^-7^, 1.755 x 10^-6^) | 1.50 x 10^-6^ (4.664 x 10^-7^, 4.973 x 10^-6^) | 5.46 x 10^-7^ (2.870 x 10^-7^, 3.027 x 10^-6^) | 8.28 x 10^-7^ (1.484 x 10^-7^, 1.2.280 x 10^-6^) | 0.03 (0.028, 0.036) |
| **60** | 5.19 x 10^-7^ (1.253 x 10^-7^, 1.599 x 10^-6^) | 1.92 x 10^-6^ (4.488 x 10^-7^, 6.079 x 10^-6^) | 5.41 x 10^-7^ (2.789 x 10^-7^, 2.589 x 10^-6^) | 8.05 x 10^-7^ (1.260 x 10^-7^, 1.926 x 10^-6^) | 0.03 (0.028, 0.036) |
| **65** | 5.58 x 10^-7^ (1.298 x 10^-7^, 1.864 x 10^-6^) | 2.77 x 10^-6^ (6.749 x 10^-7^, 7.421 x 10^-6^) | 5.97 x 10^-7^ (2.839 x 10^-7^, 2.726 x 10^-6^) | 1.22 x 10^-6^ (1.598 x 10^-7^, 2.549 x 10^-6^) | 0.03 (0.028, 0.036) |
| **70** | 5.78 x 10^-7^ (1.223 x 10^-7^, 1.791 x 10^-6^) | 3.24 x 10^-6^ (8.465 x 10^-7^, 8.653 x 10^-6^) | 6.92 x 10^-7^ (3.009 x 10^-7^, 3.079 x 10^-6^) | 1.39 x 10^-6^ (1.985 x 10^-7^, 2.624 x 10^-6^) | 0.03 (0.028, 0.036) |
| **75** | 4.27 x 10^-7^ (7.076 x 10^-8^, 1.033 x 10^-6^) | 5.32 x 10^-6^ (1.180 x 10^-6^, 1.274 x 10^-5^) | 9.26 x 10^-7^ (3.930 x 10^-7^, 4.207 x 10^-6^) | 1.64 x 10^-6^ (2.663 x 10^-7^, 3.427 x 10^-6^) | 0.03 (0.029, 0.035) |
| **80** | 4.69 x 10^-7^ (8.660 x 10^-8^, 1.217 x 10^-6^) | 9.93 x 10^-6^ (2.180 x 10^-6^, 2.539 x 10^-5^) | 8.10 x 10^-7^ (4.010 x 10^-7^, 4.151 x 10^-6^) | 1.24 x 10^-6^ (1.860 x 10^-7^, 2.804 x 10^-6^) | 0.03 (0.029, 0.036) |
| **85** | 6.26 x 10^-7^ (1.312 x 10^-7^, 1.750 x 10^-6^) | 1.24 x 10^-5^ (3.026 x 10^-6^, 3.343 x 10^-5^) | 9.34 x 10^-7^ (5.158 x 10^-7^, 5.299 x 10^-6^) | 6.75 x 10^-7^ (9.656 x 10^-8^, 1.500 x 10^-6^) | 0.04 (0.031, 0.039) |
| **90** | 4.84 x 10^-6^ (1.036 x 10^-6^, 1.204 x 10^-5^) | 1.76 x 10^-5^ (4.420 x 10^-6^, 5.262 x 10^-5^) | 1.79 x 10^-6^ (8.044 x 10^-7^, 1.021 x 10^-5^) | 1.74 x 10^-6^ (3.044 x 10^-7^, 4.640 x 10^-6^) | 0.05 (0.031, 0.054) |
| **95** | 4.99 x 10^-6^ (8.419 x 10^-7^, 1.189 x 10^-5^) | 1.19 x 10^-5^ (3.500 x 10^-6^, 3.904 x 10^-5^) | 2.69 x 10^-6^ (1.127 x 10^-6^, 1.248 x 10^-5^) | 1.86 x 10^-6^ (3.303 x 10^-7^, 5.502 x 10^-6^) | 0.06 (0.044, 0.066) |
|  | **Fibular SMA_ML_** | | | | |
| **5** | 7.46 x 10^-6^ (1.532 x 10^-6^, 1.992 x 10^-5^) | 2.22 x 10^-6^ (4.808 x 10^-7^, 5.560 x 10^-6^) | 1.92 x 10^-6^ (7.417 x 10^-7^, 9.083 x 10^-6^) | 3.31 x 10^-6^ (5.832 x 10^-7^, 7.435 x 10^-6^) | 0.04 (0.022, 0.051) |
| **10** | 3.46 x 10^-6^ (7.283 x 10^-7^, 9.722 x 10^-6^) | 1.04 x 10^-5^ (2.414 x 10^-6^, 2.660 x 10^-5^) | 7.53 x 10^-7^ (1.834 x 10^-7^, 2.896 x 10^-6^) | 1.53 x 10^-6^ (3.043 x 10^-7^, 4.492 x 10^-6^) | 0.02 (0.014, 0.032) |
| **15** | 1.24 x 10^-6^ (2.236 x 10^-7^, 3.149 x 10^-6^) | 1.42 x 10^-6^ (3.857 x 10^-6^, 4.061 x 10^-5^) | 5.11 x 10^-7^ (1.291 x 10^-7^, 1.636 x 10^-6^) | 5.62 x 10^-7^ (9.302 x 10^-8^, 1.217 x 10^-6^) | 0.02 (0.012, 0.023) |
| **20** | 5.80 x 10^-7^ (1.322 x 10^-7^, 1.703 x 10^-6^) | 1.02 x 10^-6^ (2.255 x 10^-7^, 2.851 x 10^-6^) | 3.31 x 10^-7^ (8.840 x 10^-8^, 1.134 x 10^-6^) | 4.56 x 10^-7^ (6.326 x 10^-8^, 1.002 x 10^-6^) | 0.02 (0.012, 0.020) |
| **25** | 3.22 x 10^-7^ (6.692 x 10^-8^, 9.430 x 10^-7^) | 8.00 x 10^-7^ (2.060 x 10^-7^, 2.214 x 10^-6^) | 3.03 x 10^-7^ (1.087 x 10^-7^, 1.118 x 10^-6^) | 8.27 x 10^-7^ (1.234 x 10^-7^, 1.752 x 10^-6^) | 0.02 (0.013, 0.020) |
| **30** | 4.04 x 10^-7^ (1.322 x 10^-7^, 1.703 x 10^-6^) | 1.03 x 10^-6^ (3.089 x 10^-7^, 3.286 x 10^-6^) | 1.72 x 10^-7^ (4.289 x 10^-7^, 5.025 x 10^-6^) | 4.03 x 10^-7^ (7.021 x 10^-8^, 9.998 x 10^-7^) | 0.02 (0.012, 0.021) |
| **35** | 4.20 x 10^-7^ (1.009 x 10^-7^, 1.441 x 10^-6^) | 1.20 x 10^-6^ (3.096 x 10^-7^, 3.328 x 10^-6^) | 1.61 x 10^-7^ (4.352 x 10^-8^, 5.431 x 10^-7^) | 3.52 x 10^-7^ (4.972 x 10^-8^, 8.385 x 10^-7^) | 0.02 (0.013, 0.020) |
| **40** | 6.09 x 10^-7^ (1.242 x 10^-7^, 1.496 x 10^-6^) | 1.15 x 10^-6^ (2.851 x 10^-7^, 3.364 x 10^-6^) | 1.99 x 10^-7^ (5.230 x 10^-8^, 6.567 x 10^-7^) | 3.19 x 10^-7^ (5.671 x 10^-8^, 8.365 x 10^-7^) | 0.02 (0.013, 0.020) |
| **45** | 5.64 x 10^-7^ (1.091 x 10^-7^, 1.300 x 10^-6^) | 1.08 x 10^-6^ (3.363 x 10^-7^, 3.423 x 10^-6^) | 2.30 x 10^-7^ (7.438 x 10^-8^, 8.715 x 10^-7^) | 2.13 x 10^-7^ (4.115 x 10^-8^, 6.248 x 10^-7^) | 0.02 (0.013, 0.020) |
| **50** | 4.76 x 10^-7^ (7.485 x 10^-8^, 1.198 x 10^-6^) | 1.05 x 10^-6^ (2.645 x 10^-7^, 2.950 x 10^-6^) | 2.73 x 10^-7^ (8.587 x 10^-8^, 8.960 x 10^-7^) | 2.37 x 10^-7^ (4.743 x 10^-8^, 6.638 x 10^-7^) | 0.02 (0.013, 0.020) |
| **55** | 3.30 x 10^-7^ (8.258 x 10^-8^, 1.007 x 10^-6^) | 1.50 x 10^-6^ (3.732 x 10^-7^, 4.063 x 10^-6^) | 2.58 x 10^-7^ (8.834 x 10^-8^, 9.426 x 10^-7^) | 1.69 x 10^-7^ (2.759 x 10^-8^, 5.146 x 10^-7^) | 0.02 (0.014, 0.020) |
| **60** | 3.08 x 10^-7^ (7.785 x 10^-8^, 1.012 x 10^-6^) | 2.20 x 10^-6^ (4.455 x 10^-7^, 5.343 x 10^-6^) | 2.91 x 10^-7^ (8.039 x 10^-8^, 8.719 x 10^-7^) | 1.22 x 10^-7^ (3.051 x 10^-8^, 4.025 x 10^-7^) | 0.02 (0.014, 0.020) |
| **65** | 2.60 x 10^-7^ (6.674 x 10^-8^, 8.997 x 10^-7^) | 2.76 x 10^-6^ (6.369 x 10^-7^, 6.507 x 10^-6^) | 4.13 x 10^-7^ (9.013 x 10^-8^, 1.181 x 10^-6^) | 1.77 x 10^-7^ (3.294 x 10^-8^, 4.976 x 10^-7^) | 0.02 (0.014, 0.020) |
| **70** | 2.18 x 10^-7^ (4.964 x 10^-8^, 7.431 x 10^-7^) | 2.62 x 10^-6^ (5.583 x 10^-7^, 6.593 x 10^-6^) | 4.93 x 10^-7^ (1.415 x 10^-7^, 1.327 x 10^-6^) | 1.83 x 10^-7^ (3.345 x 10^-8^, 4.529 x 10^-7^) | 0.02 (0.015, 0.020) |
| **75** | 1.88 x 10^-7^ (2.762 x 10^-8^, 4.110 x 10^-7^) | 3.79 x 10^-6^ (7.427 x 10^-7^, 8.858 x 10^-6^) | 8.04 x 10^-7^ (1.903 x 10^-7^, 2.218 x 10^-6^) | 3.03 x 10^-7^ (6.406 x 10^-8^, 9.079 x 10^-7^) | 0.02 (0.016, 0.020) |
| **80** | 3.02 x 10^-7^ (3.657 x 10^-8^, 6.243 x 10^-7^) | 9.12 x 10^-6^ (1.678 x 10^-6^, 2.000 x 10^-5^) | 7.18 x 10^-7^ (2.130 x 10^-7^, 2.202 x 10^-6^) | 5.22 x 10^-7^ (8.945 x 10^-8^, 1.293 x 10^-6^) | 0.02 (0.016, 0.021) |
| **85** | 4.37 x 10^-7^ (7.900 x 10^-8^, 8.940 x 10^-7^) | 7.96 x 10^-6^ (1.784 x 10^-6^, 2.042 x 10^-5^) | 7.53 x 10^-7^ (3.524 x 10^-7^, 3.086 x 10^-6^) | 2.47 x 10^-7^ (2.875 x 10^-8^, 5.156 x 10^-6^) | 0.02 (0.018, 0.024) |
| **90** | 1.52 x 10^-6^ (3.224 x 10^-7^, 4.436 x 10^-6^) | 7.19 x 10^-6^ (1.824 x 10^-6^, 1.851 x 10^-5^) | 9.71 x 10^-7^ (5.958 x 10^-7^, 5.420 x 10^-6^) | 7.87 x 10^-7^ (1.355 x 10^-7^, 1.918 x 10^-6^) | 0.03 (0.022, 0.034) |
| **95** | 1.01 x 10^-6^ (1.981 x 10^-7^, 2.564 x 10^-6^) | 1.87 x 10^-6^ (5.126 x 10^-7^, 5.399 x 10^-6^) | 1.60 x 10^-6^ (7.339 x 10^-7^, 7.065 x 10^-6^) | 1.05 x 10^-6^ (2.120 x 10^-7^, 2.920 x 10^-6^) | 0.04 (0.031, 0.040) |
|  | **Fibular SMA_CC_** | | | | |
| **5** | 3.45 x 10^-6^ (9.229 x 10^-7^, 1.313 x 10^-5^) | 3.29 x 10^-6^ (7.839 x 10^-7^, 8.540 x 10^-6^) | 8.94 x 10^-7^ (2.303 x 10^-7^, 3.276 x 10^-6^) | 1.94 x 10^-6^ (3.795 x 10^-7^, 4.698 x 10^-6^) | 0.03 (0.017, 0.040) |
| **10** | 2.86 x 10^-6^ (6.145 x 10^-7^, 7.964 x 10^-6^) | 1.77 x 10^-6^ (4.604 x 10^-7^, 4.494 x 10^-6^) | 2.30 x 10^-7^ (6.927 x 10^-8^, 9.555 x 10^-7^) | 3.18 x 10^-7^ (9.679 x 10^-8^, 1.257 x 10^-6^) | 0.02 (0.012, 0.029) |
| **15** | 9.20 x 10^-7^ (2.605 x 10^-7^, 3.228 x 10^-6^) | 7.30 x 10^-7^ (1.534 x 10^-7^, 1.737 x 10^-6^) | 1.15 x 10^-7^ (3.831 x 10^-8^, 5.432 x 10^-7^) | 2.56 x 10^-7^ (4.767 x 10^-8^, 6.755 x 10^-7^) | 0.02 (0.011, 0.023) |
| **20** | 5.03 x 10^-7^ (1.134 x 10^-7^, 1.961 x 10^-6^) | 2.30 x 10^-7^ (3.562 x 10^-8^, 4.978 x 10^-7^) | 1.46 x 10^-7^ (5.360 x 10^-8^, 6.843 x 10^-7^) | 4.89 x 10^-7^ (7.518 x 10^-8^, 1.112 x 10^-6^) | 0.02 (0.013, 0.022) |
| **25** | 3.32 x 10^-7^ (1.003 x 10^-7^, 1.325 x 10^-6^) | 4.18 x 10^-7^ (7.510 x 10^-8^, 9.226 x 10^-7^) | 2.21 x 10^-7^ (8.544 x 10^-8^, 8.989 x 10^-7^) | 9.64 x 10^-7^(1.307 x 10^-7^, 1.794 x 10^-6^) | 0.02 (0.014, 0.021) |
| **30** | 2.88 x 10^-7^ (7.165 x 10^-8^, 9.127 x 10^-7^) | 6.17 x 10^-7^ (1.182 x 10^-7^, 1.362 x 10^-6^) | 2.65 x 10^-7^ (7.979 x 10^-8^, 1.057 x 10^-6^) | 3.80 x 10^-7^(5.800 x 10^-8^, 8.233 x 10^-7^) | 0.02 (0.015, 0.021) |
| **35** | 3.00 x 10^-7^ (7.419 x 10^-8^, 9.161 x 10^-7^) | 7.44 x 10^-7^ (1.411 x 10^-7^, 1.737 x 10^-6^) | 2.50 x 10^-7^ (7.704 x 10^-8^, 9.490 x 10^-7^) | 3.97 x 10^-7^ (6.357 x 10^-8^, 8.042 x 10^-7^) | 0.02 (0.015, 0.021) |
| **40** | 2.75 x 10^-7^ (5.610 x 10^-8^, 7.723 x 10^-7^) | 7.01 x 10^-7^ (1.595 x 10^-7^, 1.606 x 10^-6^) | 3.10 x 10^-7^ (1.042 x 10^-7^, 1.163 x 10^-6^) | 6.05 x 10^-7^ (1.027 x 10^-7^, 1.274 x 10^-6^) | 0.02 (0.015, 0.020) |
| **45** | 2.62 x 10^-7^ (5.255 x 10^-8^, 7.294 x 10^-7^) | 6.48 x 10^-7^ (1.573 x 10^-7^, 1.707 x 10^-6^) | 2.72 x 10^-7^ (8.677 x 10^-8^, 9.968 x 10^-7^) | 4.45 x 10^-7^ (6.257 x 10^-8^, 9.768 x 10^-7^) | 0.02 (0.015, 0.021) |
| **50** | 1.85 x 10^-7^ (3.671 x 10^-8^, 4.724 x 10^-7^) | 7.46 x 10^-7^ (1.784 x 10^-7^, 1.961 x 10^-6^) | 2.91 x 10^-7^ (1.023 x 10^-7^, 1.129 x 10^-6^) | 3.92 x 10^-7^ (6.091 x 10^-8^, 8.495 x 10^-7^) | 0.02 (0.016, 0.020) |
| **55** | 1.37 x 10^-7^ (2.677 x 10^-8^, 3.681 x 10^-7^) | 6.87 x 10^-7^ (1.557 x 10^-7^, 1.721 x 10^-6^) | 2.94 x 10^-7^ (1.326 x 10^-7^, 1.362 x 10^-6^) | 4.60 x 10^-7^ (8.225 x 10^-8^, 1.002 x 10^-6^) | 0.02 (0.016, 0.020) |
| **60** | 1.29 x 10^-7^ (2.219 x 10^-8^, 3.531 x 10^-7^) | 7.44 x 10^-7^ (1.520 x 10^-7^, 1.802 x 10^-6^) | 2.96 x 10^-7^ (1.184 x 10^-7^, 1.305 x 10^-6^) | 5.25 x 10^-7^ (7.252 x 10^-8^, 1.122 x 10^-6^) | 0.02 (0.017, 0.020) |
| **65** | 1.05 x 10^-7^ (1.829 x 10^-8^, 2.610 x 10^-7^) | 7.27 x 10^-7^ (1.642 x 10^-7^, 1.787 x 10^-6^) | 3.98 x 10^-7^ (1.340 x 10^-7^, 1.597 x 10^-6^) | 6.82 x 10^-7^ (1.007 x 10^-7^, 1.345 x 10^-6^) | 0.02 (0.017, 0.020) |
| **70** | 1.10 x 10^-7^ (1.685 x 10^-8^, 2.521 x 10^-7^) | 6.03 x 10^-7^ (1.158 x 10^-7^, 1.442 x 10^-6^) | 4.41 x 10^-7^ (1.596 x 10^-7^, 1.778 x 10^-6^) | 9.74 x 10^-7^ (1.681 x 10^-7^, 1.910 x 10^-6^) | 0.02 (0.016, 0.019) |
| **75** | 1.16 x 10^-7^ (1.406 x 10^-8^, 2.237 x 10^-7^) | 1.19 x 10^-6^ (2.823 x 10^-7^, 3.145 x 10^-6^) | 4.18 x 10^-7^ (1.859 x 10^-7^, 1.942 x 10^-6^) | 1.17 x 10^-6^ (1.905 x 10^-7^, 2.471 x 10^-6^) | 0.02 (0.016, 0.019) |
| **80** | 7.78 x 10^-8^ (7.135 x 10^-9^, 1.198 x 10^-7^) | 1.83 x 10^-6^ (4.012 x 10^-7^, 4.428 x 10^-6^) | 3.57 x 10^-7^ (2.050 x 10^-7^, 2.142 x 10^-6^) | 1.01 x 10^-6^ (1.959 x 10^-7^, 2.214 x 10^-6^) | 0.02 (0.017, 0.019) |
| **85** | 2.89 x 10^-7^ (7.162 x 10^-8^, 1.063 x 10^-6^) | 4.17 x 10^-6^ (8.711 x 10^-7^, 1.126 x 10^-5^) | 5.03 x 10^-7^ (2.037 x 10^-7^, 1.910 x 10^-6^) | 2.46 x 10^-7^ (3.789 x 10^-8^, 5.872 x 10^-7^) | 0.02 (0.017, 0.023) |
| **90** | 2.18 x 10^-6^ (3.512 x 10^-7^, 5.447 x 10^-6^) | 1.38 x 10^-5^ (2.590 x 10^-6^, 3.013 x 10^-5^) | 6.81 x 10^-7^ (3.776 x 10^-7^, 3.941 x 10^-6^) | 5.80 x 10^-7^ (9.625 x 10^-8^, 1.510 x 10^-6^) | 0.03 (0.020, 0.035) |
| **95** | 4.77 x 10^-6^ (5.810 x 10^-7^, 1.011 x 10^-5^) | 8.00 x 10^-6^ (2.017 x 10^-6^, 2.375 x 10^-5^) | 1.02 x 10^-6^ (5.664 x 10^-7^, 6.823 x 10^-6^) | 1.49 x 10^-6^ (3.249 x 10^-7^, 4.912 x 10^-6^) | 0.04 (0.028, 0.048) |

**Table A5**. Parameter output for an Ornstein-Uhlenbeck model with three optima corresponding to fossorial+natatorial, scansorial, and generalist mustelids. %, α, and σ^2^ denote percentage of bone length, the rate/strength of adaptation, and rate of stochastic evolution, respectively. θ_Fos+Nat,_ θ_Sca_, and θ_Gen_ respectively denote the phenotypic optima (i.e., adaptive peaks) corresponding to fossorial and natatorial mustelids treated as a single group, scansorial mustelids, and generalist mustelids. Parameter estimates are followed by 95% confidence limits in parentheses, which were generated by parametric bootstrapping.

| **%** | **α** | **σ** | **θ_Fos+Nat_** | **θ_Sca_** | **θ_Gen_** |
| --- | --- | --- | --- | --- | --- |
|  | **Femoral CSA** | | | | |
| **5** | 0.04 (2.061 x 10^-9^, 17.795) | 2.43 x 10^-5^ (1.159 x 10^-5^, 2.765 x 10^-3^) | 0.10 (0.091, 0.117) | 0.07 (0.056, 0.119) | 0.08 (0.043, 0.129) |
| **10** | 0.04 (2.061 x 10^-9^, 19.881) | 4.83 x 10^-5^ (2.383 x 10^-5^, 7.932 x 10^-3^) | 0.10 (0.085, 0.127) | 0.04 (0.026, 0.117) | 0.04 (-0.006, 0.121) |
| **15** | 9.80 x 10^-3^ (2.061 x 10^-9^, 1.404) | 2.38 x 10^-5^ (1.323 x 10^-5^, 3.965 x 10^-4^) | 0.08 (0.066, 0.102) | 0.04 (0.034, 0.117) | 0.03 (0.008, 0.131) |
| **20** | 0.02 (2.061 x 10^-9^, 6.535) | 2.37 x 10^-5^ (1.193 x 10^-5^, 1.426 x 10^-3^) | 0.08 (0.065, 0.096) | 0.03 (0.030, 0.103) | 0.02 (-0.006, 0.101) |
| **25** | 8.64 x 10^-4^ (2.061 x 10^-9^, 1.003) | 1.83 x 10^-5^ (1.017 x 10^-5^, 1.548 x 10^-4^) | 0.07 (0.056, 0.091) | 0.03 (0.028, 0.119) | 0.01 (0.010, 0.135) |
| **30** | 3.17 x 10^-3^ (2.061 x 10^-9^, 1.895) | 2.16 x 10^-5^ (1.196 x 10^-5^, 2.237 x 10^-4^) | 0.07 (0.054, 0.088) | 0.04 (0.024, 0.109) | 0.02 (-0.010, 0.121) |
| **35** | 4.48 x 10^-3^ (2.061 x 10^-9^, 0.926) | 1.68 x 10^-5^ (9.343 x 10^-6^, 1.862 x 10^-5^) | 0.07 (0.056, 0.086) | 0.04 (0.056, 0.086) | 0.02 (0.007, 0.118) |
| **40** | 0.01 (2.061 x 10^-9^, 2.043) | 1.65 x 10^-5^ (8.689 x 10^-6^, 3.631 x 10^-4^) | 0.07 (0.056, 0.083) | 0.04 (0.028, 0.099) | 0.03 (0.003, 0.103) |
| **45** | 3.67 x 10^-3^ (2.061 x 10^-9^, 1.457) | 1.46 x 10^-5^ (7.380 x 10^-6^, 2.264 x 10^-4^) | 0.07 (0.055, 0.082) | 0.03 (0.029, 0.099) | 0.02 (0.005, 0.112) |
| **50** | 3.10 x 10^-4^ (2.061 x 10^-9^, 0.996) | 1.29 x 10^-5^ (7.328 x 10^-6^, 1.071 x 10^-4^) | 0.07 (0.053, 0.081) | 0.03 (0.033, 0.103) | 0.02 (0.013, 0.121) |
| **55** | 1.52 x 10^-4^ (2.061 x 10^-9^, 0.795) | 1.22 x 10^-5^ (6.701 x 10^-6^, 1.087 x 10^-4^) | 0.07 (0.053, 0.081) | 0.03 (0.034, 0.103) | 0.02 (0.012, 0.116) |
| **60** | 1.21 x 10^-4^ (2.061 x 10^-9^, 0.855) | 1.13 x 10^-5^ (6.106 x 10^-6^, 9.372 x 10^-5^) | 0.07 (0.055, 0.080) | 0.03 (0.032, 0.100) | 0.02 (0.019, 0.119) |
| **65** | 6.03 x 10^-5^ (2.061 x 10^-9^, 0.529) | 1.17 x 10^-5^ (6.378 x 10^-6^, 6.429 x 10^-5^) | 0.07 (0.054, 0.082) | 0.03 (0.035, 0.100) | 0.02 (0.013, 0.116) |
| **70** | 1.60 x 10^-4^ (2.061 x 10^-9^, 0.745) | 1.47 x 10^-5^ (8.235 x 10^-6^, 1.088 x 10^-4^) | 0.07 (0.053, 0.083) | 0.03 (0.031, 0.106) | 0.02 (0.016, 0.124) |
| **75** | 1.69 x 10^-4^ (2.061 x 10^-9^, 1.359) | 1.65 x 10^-5^ (8.977 x 10^-6^, 1.809 x 10^-4^) | 0.07 (0.054, 0.088) | 0.03 (0.031, 0.111) | 0.01 (0.012, 0.131) |
| **80** | 1.73 x 10^-4^ (2.061 x 10^-9^, 0.624) | 2.31 x 10^-5^ (1.206 x 10^-5^, 1.417 x 10^-4^) | 0.08 (0.059, 0.097) | 0.03 (0.026, 0.125) | 0.01 (0.010, 0.149) |
| **85** | 0.07 (2.285 x 10^-9^, 19.891) | 1.15 x 10^-4^ (5.057 x 10^-5^, 1.641 x 10^-2^) | 0.10 (0.075, 0.117) | 0.03 (-0.003, 0.109) | 0.02 (-0.053, 0.105) |
| **90** | 0.11 (8.157 x 10^-3^, 19.935) | 1.03 x 10^-4^ (3.937 x 10^-5^, 1.332 x 10^-2^) | 0.13 (0.108, 0.141) | 0.06 (0.031, 0.109) | 0.08 (0.031, 0.109) |
| **95** | 0.03 (2.061 x 10^-9^, 2.073) | 1.71 x 10^-5^ (7.867 x 10^-6^, 2.961 x 10^-4^) | 0.11 (0.097, 0.122) | 0.07 (0.065, 0.129) | 0.08 (0.050, 0.140) |
|  | **Femoral SMA_ML_** | | | | |
| **5** | 0.46 (0.078, 19.943) | 2.78 x 10^-5^ (4.872 x 10^-6^, 1.239 x 10^-3^) | 0.06 (0.056, 0.063) | 0.04 (0.038, 0.053) | 0.05 (0.038, 0.053) |
| **10** | 1.14 (0.052, 19.945) | 1.81 x 10^-4^ (1.229 x 10^-5^, 3.487 x 10^-3^) | 0.06 (0.052, 0.065) | 0.04 (0.023, 0.049) | 0.04 (0.010, 0.053) |
| **15** | 6.89 x 10^-3^ (2.061 x 10^-9^, 0.921) | 4.90 x 10^-6^ (2.704 x 10^-6^, 4.944 x 10^-5^) | 0.05 (0.039, 0.056) | 0.03 (0.026, 0.067) | 0.02 (0.013, 0.075) |
| **20** | 0.02 (2.061 x 10^-9^, 18.244) | 6.37 x 10^-6^ (2.984 x 10^-6^, 7.324 x 10^-4^) | 0.05 (0.041, 0.057) | 0.02 (0.022, 0.058) | 0.02 (0.004, 0.062) |
| **25** | 0.04 (2.061 x 10^-9^, 19.734) | 7.21 x 10^-6^ (3.446 x 10^-6^, 9.643 x 10^-4^) | 0.05 (0.0.39, 0.054) | 0.02 (0.018, 0.054) | 0.02 (-0.002, 0.050) |
| **30** | 0.03 (2.061 x 10^-9^, 4.168) | 5.96 x 10^-6^ (2.885 x 10^-6^, 3.510 x 10^-4^) | 0.04 (0.0.37, 0.050) | 0.02 (0.018, 0.050) | 0.02 (0.002, 0.052) |
| **35** | 0.03 (2.061 x 10^-9^, 18.757) | 4.71 x 10^-6^ (2.120 x 10^-6^, 5.355 x 10^-4^) | 0.04 (0.0.35, 0.048) | 0.02 (0.018, 0.051) | 0.02 (0.007, 0.051) |
| **40** | 0.02 (2.061 x 10^-9^, 1.991) | 4.29 x 10^-6^ (2.130 x 10^-6^, 7.153 x 10^-5^) | 0.04 (0.034, 0.047) | 0.02 (0.019, 0.052) | 0.02 (0.007, 0.054) |
| **45** | 0.02 (2.061 x 10^-9^, 1.360) | 4.34 x 10^-6^ (2.351 x 10^-6^, 6.741 x 10^-5^) | 0.04 (0.303, 0.047) | 0.02 (0.020, 0.054) | 0.02 (0.005, 0.056) |
| **50** | 3.84 x 10^-3^ (2.061 x 10^-9^, 0.649) | 4.03 x 10^-6^ (2.330 x 10^-6^, 3.072 x 10^-5^) | 0.04 (0.032, 0.048) | 0.02 (0.020, 0.058) | 0.01 (0.009, 0.064) |
| **55** | 3.93 x 10^-3^ (2.061 x 10^-9^, 0.731) | 4.51 x 10^-6^ (2.453 x 10^-6^, 3.511 x 10^-5^) | 0.04 (0.033, 0.049) | 0.02 (0.019, 0.059) | 0.01 (0.010, 0.066) |
| **60** | 0.02 (2.061 x 10^-9^, 1.260) | 5.25 x 10^-6^ (2.872 x 10^-6^, 5.772 x 10^-5^) | 0.04 (0.033, 0.049) | 0.02 (0.018, 0.056) | 0.01 (0.005, 0.060) |
| **65** | 0.02 (2.061 x 10^-9^, 1.267) | 6.10 x 10^-6^ (3.119 x 10^-6^, 6.579 x 10^-5^) | 0.04 (0.035, 0.050) | 0.02 (0.017, 0.058) | 0.01 (-0.002, 0.057) |
| **70** | 0.03 (2.061 x 10^-9^, 6.309) | 8.10 x 10^-6^ (3.909 x 10^-6^, 4.348 x 10^-4^) | 0.04 (0.035, 0.052) | 0.02 (0.013, 0.056) | 0.01 (-0.004, 0.058) |
| **75** | 0.04 (2.061 x 10^-9^, 14.426) | 1.00 x 10^-5^ (4.443 x 10^-6^, 9.267 x 10^-4^) | 0.05 (0.037, 0.054) | 0.02 (0.015, 0.056) | 0.01 (-0.009, 0.054) |
| **80** | 0.07 (2.061 x 10^-9^, 19.880) | 1.66 x 10^-5^ (7.111 x 10^-6^, 2.259 x 10^-3^) | 0.05 (0.043, 0.059) | 0.02 (0.014, 0.056) | 0.02 (-0.009, 0.055) |
| **85** | 0.13 (0.023, 19.942) | 7.57 x 10^-5^ (3.294 x 10^-5^, 9.700 x 10^-3^) | 0.07 (0.054, 0.079) | 0.03 (0.004, 0.063) | 0.03 (-0.028, 0.061) |
| **90** | 0.06 (2.061 x 10^-9^, 19.889) | 2.54 x 10^-5^ (1.193 x 10^-5^, 3.254 x 10^-3^) | 0.08 (0.072, 0.094) | 0.03 (0.028, 0.079) | 0.05 (0.019, 0.092) |
| **95** | 7.77 x 10^-4^ (2.061 x 10^-9^, 0.604) | 3.79 x 10^-6^ (2.014 x 10^-6^, 2.803 x 10^-5^) | 0.07 (0.066, 0.081) | 0.05 (0.053, 0.094) | 0.05 (0.040, 0.102) |
|  | **Femoral SMA_CC_** | | | | |
| **5** | 0.08 (2.198 x 10^-9^, 19.943) | 2.23 x 10^-5^ (9.973 x 10^-6^, 3.495 x 10^-3^) | 0.09 (0.079, 0.096) | 0.06 (0.047, 0.088) | 0.06 (0.014, 0.081) |
| **10** | 0.42 (0.015, 19.946) | 1.74 x 10^-4^ (2.679 x 10^-5^, 8.659 x 10^-3^) | 0.09 (0.080, 0.102) | 0.04 (0.021, 0.078) | 0.05 (-0.034, 0.070) |
| **15** | 6.81 x 10^-3^ (2.061 x 10^-9^, 2.310) | 1.99 x 10^-5^ (1.058 x 10^-5^, 3.742 x 10^-4^) | 0.07 (0.052, 0.083) | 0.02 (0.022, 0.099) | 0.01 (-0.005, 0.110) |
| **20** | 0.01 ^3^ (2.061 x 10^-9^, 2.922) | 1.14 x 10^-5^ (6.212 x 10^-6^, 4.222 x 10^-4^) | 0.06 (0.051, 0.072) | 0.02 (0.024, 0.077) | 0.01 (-0.001, 0.080) |
| **25** | 6.96 x 10^-3^ (2.061 x 10^-9^, 0.830) | 1.13 x 10^-5^ (5.968 x 10^-6^, 8.870 x 10^-5^) | 0.05 (0.041, 0.065) | 0.02 (0.020, 0.077) | 0.01 (-0.000, 0.084) |
| **30** | 9.58 x 10^-3^ (2.061 x 10^-9^, 1.342) | 9.74 x 10^-6^ (5.218 x 10^-6^, 1.247 x 10^-4^) | 0.05 (0.037, 0.059) | 0.02 (0.018, 0.070) | 0.01 (-0.004, 0.073) |
| **35** | 9.16 x 10^-4^ (2.061 x 10^-9^, 0.797) | 5.59 x 10^-6^ (3.097 x 10^-6^, 4.586 x 10^-5^) | 0.04 (0.035, 0.054) | 0.02 (0.023, 0.067) | 0.01 (0.013, 0.077) |
| **40** | 5.70 x 10^-4^ (2.061 x 10^-9^, 0.692) | 4.80 x 10^-6^ (2.632 x 10^-6^, 3.575 x 10^-5^) | 0.04 (0.034, 0.052) | 0.02 (0.022, 0.066) | 0.02 (0.010, 0.075) |
| **45** | 3.06 x 10^-4^ (2.061 x 10^-9^, 0.693) | 4.50 x 10^-6^ (2.586 x 10^-6^, 3.656 x 10^-5^) | 0.04 (0.034, 0.050) | 0.02 (0.020, 0.063) | 0.01 (0.012, 0.073) |
| **50** | 1.83 x 10^-4^ (2.061 x 10^-9^, 0.727) | 4.63 x 10^-6^ (2.439 x 10^-6^, 3.494 x 10^-5^) | 0.04 (0.034, 0.052) | 0.02 (0.020, 0.065) | 0.01 (0.011, 0.077) |
| **55** | 2.42 x 10^-4^ (2.061 x 10^-9^, 0.453) | 5.14 x 10^-6^ (2.803 x 10^-6^, 3.000 x 10^-5^) | 0.04 (0.034, 0.053) | 0.02 (0.021, 0.067) | 0.01 (0.009, 0.074) |
| **60** | 4.23 x 10^-4^ (2.061 x 10^-9^, 0.783) | 6.13 x 10^-6^ (3.368 x 10^-6^, 4.841 x 10^-5^) | 0.04 (0.035, 0.054) | 0.02 (0.022, 0.070) | 0.01 (0.006, 0.080) |
| **65** | 6.18 x 10^-4^ (2.061 x 10^-9^, 0.506) | 7.58 x 10^-6^ (4.262 x 10^-6^, 5.069 x 10^-5^) | 0.05 (0.035, 0.057) | 0.02 (0.018, 0.074) | 8.62 x 10^-3^ (0.004, 0.087) |
| **70** | 9.88 x 10^-4^ (2.061 x 10^-9^, 0.646) | 1.01 x 10^-5^ (5.547 x 10^-6^, 5.847 x 10^-5^) | 0.05 (0.036, 0.062) | 0.02 (0.016, 0.078) | 6.24 x 10^-3^ (0.005, 0.095) |
| **75** | 1.57 x 10^-3^ (2.061 x 10^-9^, 0.894) | 1.13 x 10^-5^ (6.163 x 10^-6^, 9.414 x 10^-5^) | 0.05 (0.039, 0.066) | 0.02 (0.020, 0.088) | 2.53 x 10^-3^ (0.005, 0.100) |
| **80** | 3.13 x 10^-3^ (2.061 x 10^-9^, 0.602) | 1.39 x 10^-5^ (7.851 x 10^-6^, 8.862 x 10^-5^) | 0.06 (0.044, 0.073) | 0.02 (0.022, 0.090) | 6.68 x 10^-4^ (-0.001, 0.100) |
| **85** | 0.07 (2.359 x 10^-9^, 19.942) | 5.21 x 10^-5^ (2.325 x 10^-5^, 7.999 x 10^-3^) | 0.07 (0.059, 0.088) | 0.03 (0.005, 0.079) | 0.01 (-0.040, 0.068) |
| **90** | 0.07 (2.061 x 10^-9^, 19.890) | 2.95 x 10^-5^ (1.242 x 10^-5^, 4.123 x 10^-3^) | 0.09 (0.078, 0.102) | 0.04 (0.030, 0.087) | 0.05 (0.019, 0.096) |
| **95** | 7.47 x 10^-3^ (2.061 x 10^-9^, 1.285) | 6.22 x 10^-6^ (3.144 x 10^-6^, 7.634 x 10^-5^) | 0.08 (0.073, 0.092) | 0.05 (0.055, 0.103) | 0.06 (0.042, 0.109) |
|  | **Tibial CSA** | | | | |
| **5** | 5.02 (0.165, 19.948) | 1.36 x 10^-3^ (4.121 x 10^-5^, 6.330 x 10^-3^) | 0.12 (0.116, 0.128) | 0.09 (0.084, 0.103) | 0.09 (0.067, 0.101) |
| **10** | 0.01 (2.061 x 10^-9^, 19.917) | 2.95 x 10^-5^ (1.580 x 10^-5^, 6.107 x 10^-3^) | 0.11 (0.093, 0.122) | 0.03 (0.036, 0.115) | 0.05 (-0.013, 0.103) |
| **15** | 7.38 x 10^-4^ (2.061 x 10^-9^, 1.249) | 9.43 x 10^-6^ (4.590 x 10^-6^, 1.080 x 10^-4^) | 0.09 (0.074, 0.099) | 0.04 (0.057, 0.117) | 0.05 (0.038, 0.130) |
| **20** | 0.03 (2.061 x 10^-9^, 19.931) | 9.08 x 10^-6^ (5.163 x 10^-6^, 1.757 x 10^-3^) | 0.08 (0.068, 0.083) | 0.04 (0.039, 0.075) | 0.05 (0.012, 0.067) |
| **25** | 0.02 (2.061 x 10^-9^, 19.931) | 6.69 x 10^-6^ (3.830 x 10^-6^, 1.266 x 10^-3^) | 0.07 (0.067, 0.080) | 0.04 (0.039, 0.073) | 0.04 (0.012, 0.067) |
| **30** | 0.03 (2.061 x 10^-9^, 19.928) | 6.01 x 10^-6^ (3.325 x 10^-6^, 1.172 x 10^-3^) | 0.07 (0.066, 0.077) | 0.04 (0.039, 0.068) | 0.04 (0.012, 0.061) |
| **35** | 0.05 (2.061 x 10^-9^, 19.942) | 7.60 x 10^-6^ (4.291 x 10^-6^, 1.363 x 10^-3^) | 0.07 (0.065, 0.075) | 0.04 (0.035, 0.062) | 0.04 (0.004, 0.056) |
| **40** | 0.04 (2.061 x 10^-9^, 19.924) | 7.32 x 10^-6^ (3.899 x 10^-6^, 1.418 x 10^-3^) | 0.07 (0.064, 0.075) | 0.04 (0.034, 0.064) | 0.04 (0.001, 0.058) |
| **45** | 0.04 (2.061 x 10^-9^, 19.930) | 8.09 x 10^-6^ (4.685 x 10^-6^, 1.538 x 10^-3^) | 0.07 (0.063, 0.076) | 0.04 (0.034, 0.064) | 0.04 (-0.009, 0.058) |
| **50** | 0.03 (2.061 x 10^-9^, 19.933) | 8.34 x 10^-6^ (4.915 x 10^-6^, 1.656 x 10^-3^) | 0.07 (0.063, 0.075) | 0.04 (0.032, 0.064) | 0.04 (-0.005, 0.057) |
| **55** | 0.02 (2.061 x 10^-9^, 19.933) | 8.21 x 10^-6^ (4.562 x 10^-6^, 1.689 x 10^-3^) | 0.07 (0.061, 0.076) | 0.04 (0.032, 0.067) | 0.03 (-0.001, 0.060) |
| **60** | 0.01 (2.061 x 10^-9^, 18.849) | 7.43 x 10^-6^ (4.235 x 10^-6^, 1.309 x 10^-3^) | 0.07 (0.060, 0.075) | 0.03 (0.033, 0.071) | 0.03 (0.004, 0.064) |
| **65** | 3.07 x 10^-3^ (2.061 x 10^-9^, 1.223) | 5.77 x 10^-6^ (3.019 x 10^-6^, 6.614 x 10^-5^) | 0.07 (0.058, 0.075) | 0.03 (0.040, 0.083) | 0.03 (0.025, 0.090) |
| **70** | 5.18 x 10^-3^ (2.061 x 10^-9^, 2.645) | 4.98 x 10^-6^ (2.725 x 10^-6^, 1.050 x 10^-5^) | 0.07 (0.058, 0.074) | 0.03 (0.039, 0.077) | 0.03 (0.020, 0.077) |
| **75** | 6.78 x 10^-3^ (2.061 x 10^-9^, 19.105) | 5.50 x 10^-6^ (3.167 x 10^-6^, 8.338 x 10^-5^) | 0.07 (0.059, 0.074) | 0.03 (0.038, 0.074) | 0.03 (0.017, 0.070) |
| **80** | 5.84 x 10^-3^ (2.061 x 10^-9^, 3.125) | 6.50 x 10^-6^ (3.676 x 10^-6^, 2.196 x 10^-4^) | 0.07 (0.060, 0.077) | 0.03 (0.037, 0.079) | 0.03 (0.013, 0.079) |
| **85** | 0.01 (2.061 x 10^-9^, 18.144) | 1.16 x 10^-5^ (6.685 x 10^-6^, 1.965 x 10^-3^) | 0.08 (0.066, 0.085) | 0.02 (0.031, 0.080) | 0.03 (-0.001, 0.073) |
| **90** | 0.03 (2.061 x 10^-9^, 19.939) | 2.31 x 10^-5^ (1.260 x 10^-5^, 4.646 x 10^-3^) | 0.09 (0.078, 0.101) | 0.03 (0.032, 0.089) | 0.04 (-0.020, 0.077) |
| **95** | 0.96 (2.061 x 10^-9^, 19.948) | 2.78 x 10^-4^ (4.035 x 10^-5^, 6.736 x 10^-3^) | 0.07 (0.063, 0.076) | 0.07 (0.062, 0.082) | 0.07 (0.061, 0.084) |
|  | **Tibial SMA_ML_** | | | | |
| **5** | 0.42 (0.022, 19.946) | 2.19 x 10^-5^ (3.390 x 10^-6^, 1.020 x 10^-3^) | 0.08 (0.075, 0.081) | 0.06 (0.056, 0.071) | 0.06 (0.030, 0.066) |
| **10** | 0.02 (2.061 x 10^-9^, 19.923) | 1.59 x 10^-5^ (8.922 x 10^-6^, 2.830 x 10^-3^) | 0.08 (0.068, 0.090) | 0.03 (0.029, 0.084) | 0.05 (0.005, 0.085) |
| **15** | 1.41 x 10^-5^ (2.061 x 10^-9^, 0.947) | 5.63 x 10^-6^ (3.165 x 10^-6^, 5.456 x 10^-5^) | 0.07 (0.056, 0.075) | 0.03 (0.043, 0.090) | 0.05 (0.033, 0.101) |
| **20** | 1.45 x 10^-3^ (2.061 x 10^-9^, 0.791) | 4.05 x 10^-6^ (2.434 x 10^-6^, 2.935 x 10^-5^) | 0.06 (0.051, 0.065) | 0.03 (0.036, 0.071) | 0.04 (0.025, 0.077) |
| **25** | 4.17 x 10^-3^ (2.061 x 10^-9^, 2.132) | 3.25 x 10^-6^ (1.864 x 10^-6^, 6.222 x 10^-5^) | 0.05 (0.048, 0.061) | 0.03 (0.033, 0.062) | 0.04 (0.024, 0.065) |
| **30** | 1.24 x 10^-3^ (2.061 x 10^-9^, 1.606) | 2.50 x 10^-6^ (1.326 x 10^-6^, 2.914 x 10^-5^) | 0.05 (0.046, 0.058) | 0.03 (0.036, 0.065) | 0.03 (0.027, 0.069) |
| **35** | 4.37 (2.061 x 10^-9^, 3.082) | 2.43 x 10^-6^ (1.392 x 10^-6^, 7.432 x 10^-5^) | 0.05 (0.044, 0.054) | 0.03 (0.029, 0.056) | 0.03 (0.019, 0.058) |
| **40** | 0.02 (2.061 x 10^-9^, 19.630) | 2.78 x 10^-6^ (1.471 x 10^-6^, 4.545 x 10^-4^) | 0.05 (0.042, 0.051) | 0.03 (0.026, 0.050) | 0.03 (0.010, 0.046) |
| **45** | 0.03 (2.061 x 10^-9^, 19.920) | 2.92 x 10^-6^ (1.640 x 10^-6^, 5.365 x 10^-4^) | 0.05 (0.041, 0.049) | 0.03 (0.024, 0.045) | 0.03 (0.008, 0.041) |
| **50** | 0.03 (2.061 x 10^-9^, 19.941) | 3.14 x 10^-6^ (1.897 x 10^-6^, 6.380 x 10^-4^) | 0.04 (0.039, 0.047) | 0.02 (0.021, 0.041) | 0.03 (0.004, 0.037) |
| **55** | 0.04 (4.985 x 10^-9^, 19.941) | 3.41 x 10^-6^ (2.124 x 10^-6^, 7.130 x 10^-4^) | 0.04 (0.038, 0.045) | 0.02 (0.021, 0.039) | 0.03 (0.003, 0.035) |
| **60** | 0.05 (1.612 x 10^-3^, 19.945) | 3.43 x 10^-6^ (1.807 x 10^-6^, 6.186 x 10^-4^) | 0.04 (0.037, 0.043) | 0.02 (0.021, 0.037) | 0.03 (0.002, 0.034) |
| **65** | 0.04 (8.234 x 10^-4^, 19.943) | 3.00 x 10^-6^ (1.847 x 10^-6^, 5.705 x 10^-4^) | 0.04 (0.036, 0.043) | 0.02 (0.022, 0.038) | 0.03 (0.007, 0.034) |
| **70** | 0.05 (2.077 x 10^-3^, 19.941) | 2.92 x 10^-6^ (1.440 x 10^-6^, 5.198 x 10^-4^) | 0.04 (0.036, 0.042) | 0.02 (0.021, 0.037) | 0.02 (0.005, 0.033) |
| **75** | 0.07 (6.007 x 10^-3^, 19.937) | 3.62 x 10^-6^ (1.743 x 10^-6^, 5.686 x 10^-4^) | 0.04 (0.036, 0.042) | 0.02 (0.021, 0.036) | 0.02 (0.004, 0.032) |
| **80** | 0.06 (3.651 x 10^-9^, 19.945) | 4.13 x 10^-6^ (2.082 x 10^-6^, 7.276 x 10^-4^) | 0.04 (0.038, 0.044) | 0.02 (0.018, 0.037) | 0.02 (-0.004, 0.032) |
| **85** | 0.05 (2.387 x 10^-9^, 19.942) | 6.58 x 10^-6^ (3.258 x 10^-6^, 1.298 x 10^-3^) | 0.05 (0.042, 0.052) | 0.02 (0.019, 0.042) | 0.02 (-0.009, 0.036) |
| **90** | 0.04 (2.061 x 10^-9^, 19.938) | 8.07 x 10^-6^ (4.522 x 10^-6^, 1.721 x 10^-3^) | 0.06 (0.050, 0.063) | 0.02 (0.022, 0.053) | 0.02 (-0.010, 0.047) |
| **95** | 0.70 (0.147, 19.946) | 4.78 x 10^-5^ (7.408 x 10^-6^, 1.495 x 10^-3^) | 0.05 (0.044, 0.051) | 0.04 (0.039, 0.050) | 0.04 (0.038, 0.050) |
|  | **Tibial SMA_CC_** | | | | |
| **5** | 0.15 (9.294 x 10^-3^, 19.947) | 2.18 x 10^-5^ (7.615 x 10^-6^, 2.491 x 10^-3^) | 0.09 (0.085, 0.095) | 0.06 (0.050, 0.076) | 0.06 (0.013, 0.071) |
| **10** | 0.01 (2.061 x 10^-9^, 19.862) | 1.56 x 10^-5^ (8.730 x 10^-6^, 2.917 x 10^-3^) | 0.08 (0.068, 0.092) | 0.02 (0.028, 0.090) | 0.03 (6.815 x 10^-4^, 0.091) |
| **15** | 3.26 x 10^-4^ (2.061 x 10^-9^, 0.630) | 4.91 x 10^-6^ (2.564 x 10^-6^, 3.309 x 10^-5^) | 0.06 (0.050, 0.066) | 0.02 (0.037, 0.083) | 0.03 (0.025, 0.092) |
| **20** | 0.07 (3.252 x 10^-9^, 19.942) | 5.55 x 10^-6^ (2.578 x 10^-6^, 8.687 x 10^-4^) | 0.05 (0.043, 0.051) | 0.03 (0.021, 0.043) | 0.03 (0.001, 0.039) |
| **25** | 0.06 (2.061 x 10^-9^, 19.921) | 3.88 x 10^-6^ (1.842 x 10^-6^, 6.322 x 10^-4^) | 0.04 (0.040, 0.047) | 0.03 (0.022, 0.041) | 0.02 (4.495 x 10^-4^, 0.037) |
| **30** | 0.07 (2.061 x 10^-9^, 19.942) | 3.31 x 10^-6^ (1.559 x 10^-6^, 5.730 x 10^-4^) | 0.04 (0.038, 0.044) | 0.03 (0.022, 0.038) | 0.02 (8.243 x 10^-4^, 0.037) |
| **35** | 0.08 (1.443 x 10^-8^, 19.944) | 2.99 x 10^-6^ (1.404 x 10^-6^, 4.974 x 10^-4^) | 0.04 (0.036, 0.042) | 0.03 (0.022, 0.037) | 0.02 (0.003, 0.037) |
| **40** | 0.06 (2.061 x 10^-9^, 19.939) | 2.59 x 10^-6^ (1.241 x 10^-6^, 4.639 x 10^-4^) | 0.04 (0.035, 0.041) | 0.03 (0.022, 0.037) | 0.02 (0.002, 0.032) |
| **45** | 0.05 (2.061 x 10^-9^, 19.914) | 2.55 x 10^-6^ (1.218 x 10^-6^, 4.271 x 10^-4^) | 0.04 (0.035, 0.041) | 0.02 (0.020, 0.037) | 0.02 (0.002, 0.032) |
| **50** | 0.02 (2.061 x 10^-9^, 19.465) | 2.23 x 10^-6^ (1.174 x 10^-6^, 3.190 x 10^-4^) | 0.04 (0.034, 0.042) | 0.02 (0.021, 0.041) | 0.02 (0.004, 0.036) |
| **55** | 7.02 x 10^-3^ (2.061 x 10^-9^, 2.496) | 2.19 x 10^-6^ (1.136 x 10^-6^, 5.423 x 10^-5^) | 0.04 (0.033, 0.044) | 0.02 (0.023, 0.048) | 0.02 (0.012, 0.048) |
| **60** | 2.10 x 10^-3^ (2.061 x 10^-9^, 0.701) | 2.11 x 10^-6^ (1.001 x 10^-6^, 1.603 x 10^-5^) | 0.04 (0.034, 0.045) | 0.02 (0.025, 0.053) | 0.02 (0.017, 0.061) |
| **65** | 6.40 x 10^-4 3^ (2.061 x 10^-9^, 0.581) | 2.16 x 10^-6^ (1.185 x 10^-6^, 1.143 x 10^-5^) | 0.04 (0.035, 0.047) | 0.02 (0.026, 0.055) | 0.02 (0.020, 0.063) |
| **70** | 5.08 x 10^-3^ (2.061 x 10^-9^, 0.725) | 2.27 x 10^-6^ (1.212 x 10^-6^, 1.711 x 10^-5^) | 0.04 (0.036, 0.047) | 0.02 (0.026, 0.054) | 0.02 (0.016, 0.059) |
| **75** | 0.02 (2.061 x 10^-9^, 19.730) | 3.17 x 10^-6^ (1.810 x 10^-6^, 4.995 x 10^-5^) | 0.04 (0.038, 0.048) | 0.02 (0.021, 0.046) | 0.02 (-0.007, 0.041) |
| **80** | 0.03 (2.061 x 10^-9^, 19.728) | 4.67 x 10^-6^ (2.633 x 10^-6^, 8.230 x 10^-4^) | 0.05 (0.040, 0.051) | 0.02 (0.017, 0.044) | 0.02 (-0.003, 0.039) |
| **85** | 0.05 (2.061 x 10^-9^, 19.942) | 8.59 x 10^-6^ (4.710 x 10^-6^, 1.627 x 10^-3^) | 0.05 (0.046, 0.058) | 0.02 (0.018, 0.046) | 0.02 (-0.013, 0.042) |
| **90** | 0.09 (0.014, 19.945) | 1.21 x 10^-5^ (5.881 x 10^-6^, 1.899 x 10^-3^) | 0.06 (0.056, 0.067) | 0.03 (0.024, 0.051) | 0.04 (0.004, 0.046) |
| **95** | 0.68 (0.180, 19.947) | 7.10 x 10^-5^ (1.474 x 10^-5^, 2.457 x 10^-3^) | 0.05 (0.041, 0.049) | 0.05 (0.040, 0.052) | 0.04 (0.038, 0.052) |
|  | **Fibular CSA** | | | | |
| **5** | 0.38 (0.128, 19.935) | 2.43 x 10^-5^ (7.212 x 10^-6^, 1.260 x 10^-3^) | 0.05 (0.048, 0.054) | 0.02 (0.014, 0.030) | 0.04 (0.019, 0.040) |
| **10** | 1.47 x 10^-3^ (2.061 x 10^-9^, 0.665) | 2.21 x 10^-6^ (1.149 x 10^-6^, 1.535 x 10^-5^) | 0.04 (0.032, 0.044) | 8.10 x 10^-3^ (0.023, 0.053) | 0.02 (0.018, 0.057) |
| **15** | 0.05 (2.061 x 10^-9^, 19.910) | 1.83 x 10^-6^ (8.862 x 10^-7^, 2.765 x 10^-4^) | 0.03 (0.029, 0.035) | 0.01 (0.012, 0.028) | 0.02 (0.005, 0.031) |
| **20** | 0.05 (2.061 x 10^-9^, 19.811) | 1.39 x 10^-6^ (6.716 x 10^-7^, 1.923 x 10^-4^) | 0.03 (0.028, 0.034) | 0.01 (0.014, 0.029) | 0.02 (0.009, 0.030) |
| **25** | 0.07 (2.061 x 10^-9^, 19.742) | 1.57 x 10^-6^ (6.738 x 10^-7^, 1.991 x 10^-4^) | 0.03 (0.029, 0.034) | 0.02 (0.013, 0.027) | 0.02 (0.009, 0.029) |
| **30** | 0.02 (2.061 x 10^-9^, 8.019) | 1.09 x 10^-6^ (5.525 x 10^-7^, 7.300 x 10^-5^) | 0.03 (0.030, 0.036) | 0.02 (0.019, 0.033) | 0.02 (0.013, 0.035) |
| **35** | 0.06 (2.061 x 10^-9^, 19.801) | 1.62 x 10^-6^ (7.557 x 10^-7^, 2.380 x 10^-4^) | 0.03 (0.030, 0.035) | 0.02 (0.016, 0.035) | 0.02 (0.010, 0.030) |
| **40** | 0.08 (5.637 x 10^-3^, 19.940) | 2.03 x 10^-6^ (9.493 x 10^-7^, 2.701 x 10^-4^) | 0.03 (0.030, 0.035) | 0.02 (0.015, 0.028) | 0.02 (0.008, 0.029) |
| **45** | 0.05 (2.061 x 10^-9^, 19.924) | 1.58 x 10^-6^ (8.370 x 10^-7^, 2.548 x 10^-4^) | 0.03 (0.031, 0.036) | 0.02 (0.016, 0.029) | 0.02 (0.007, 0.029) |
| **50** | 0.10 (1.809 x 10^-3^, 19.939) | 1.87 x 10^-6^ (7.497 x 10^-7^, 2.799 x 10^-4^) | 0.03 (0.031, 0.036) | 0.02 (0.016, 0.028) | 0.02 (0.008, 0.029) |
| **55** | 0.10 (2.061 x 10^-9^, 19.943) | 1.80 x 10^-6^ (7.772 x 10^-7^, 2.569 x 10^-4^) | 0.03 (0.032, 0.036) | 0.02 (0.014, 0.028) | 0.02 (0.006, 0.028) |
| **60** | 0.29 (1.247 x 10^-3^, 19.945) | 4.31 x 10^-6^ (9.288 x 10^-7^, 2.826 x 10^-4^) | 0.03 (0.032, 0.035) | 0.02 (0.015, 0.027) | 0.02 (0.006, 0.027) |
| **65** | 0.61 (0.036, 19.946) | 1.02 x 10^-5^ (1.401 x 10^-6^, 3.546 x 10^-4^) | 0.03 (0.032, 0.035) | 0.02 (0.016, 0.026) | 0.02 (0.009, 0.027) |
| **70** | 0.46 (0.067, 19.945) | 9.08 x 10^-6^ (1.478 x 10^-6^, 3.771 x 10^-4^) | 0.03 (0.031, 0.035) | 0.02 (0.016, 0.026) | 0.02 (0.011, 0.028) |
| **75** | 0.47 (0.076, 19.947) | 1.27 x 10^-5^ (2.357 x 10^-6^, 5.457 x 10^-4^) | 0.03 (0.032, 0.036) | 0.02 (0.016, 0.026) | 0.02 (0.012, 0.028) |
| **80** | 0.22 (0.069, 19.946) | 9.92 x 10^-6^ (3.324 x 10^-6^, 8.096 x 10^-4^) | 0.04 (0.032, 0.038) | 0.02 (0.015, 0.027) | 0.02 (0.008, 0.029) |
| **85** | 0.09 (5.889 x 10^-3^, 19.946) | 7.24 x 10^-6^ (2.872 x 10^-6^, 8.947 x 10^-4^) | 0.04 (0.034, 0.043) | 0.02 (0.012, 0.034) | 0.02 (3.237 x 10^-4^, 0.035) |
| **90** | 0.16 (0.045, 19.945) | 1.77 x 10^-5^ (6.757 x 10^-6^, 1.983 x 10^-3^) | 0.05 (0.044, 0.053) | 0.02 (0.012, 0.035) | 0.03 (-4.493 x 10^-4^, 0.038) |
| **95** | 0.06 (2.061 x 10^-9^, 19.912) | 9.19 x 10^-6^ (4.383 x 10^-6^, 1.339 x 10^-3^) | 0.06 (0.053, 0.066) | 0.02 (0.023, 0.056) | 0.03 (0.004, 0.057) |
|  | **Fibular SMA_ML_** | | | | |
| **5** | 0.21 (0.092, 3.053) | 1.09 x 10^-5^ (4.344 x 10^-6^, 1.161 x 10^-4^) | 0.04 (0.036, 0.042) | 0.02 (0.009, 0.024) | 0.03 (0.022, 0.039) |
| **10** | 0.25 (0.043, 19.935) | 1.59 x 10^-5^ (3.980 x 10^-6^, 1.048 x 10^-3^) | 0.03 (0.022, 0.030) | 8.50 x 10^-3^ (-3.35 x 10^-4^, 0.019) | 0.01 (-0.006, 0.023) |
| **15** | 0.22 (0.035, 19.941) | 3.33 x 10^-6^ (9.456 x 10^-7^, 2.422 x 10^-4^) | 0.02 (0.017, 0.021) | 8.41 x 10^-3^ (0.004, 0.013) | 0.01 (0.004, 0.016) |
| **20** | 0.14 (0.020, 19.932) | 1.52 x 10^-6^ (5.519 x 10^-7^, 1.738 x 10^-4^) | 0.02 (0.016, 0.019) | 8.26 x 10^-3^ (0.005, 0.014) | 9.79 x 10^-3^ (0.001, 0.015) |
| **25** | 0.08 (2.805 x 10^-9^, 19.932) | 1.04 x 10^-6^ (4.632 x 10^-7^, 1.376 x 10^-4^) | 0.02 (0.016, 0.020) | 8.11 x 10^-3^ (0.006, 0.015) | 8.05 x 10^-3^ (-2.463 x 10^-4^, 0.016) |
| **30** | 9.35 x 10^-3^ (2.061 x 10^-9^, 1.651) | 5.63 x 10^-7^ (3.132 x 10^-7^, 8.018 x 10^-4^) | 0.02 (0.016, 0.021) | 7.67 x 10^-3^ (0.009, 0.023) | 4.70 x 10^-3^ (0.005, 0.024) |
| **35** | 7.69 x 10^-3^ (2.061 x 10^-9^, 1.575) | 5.83 x 10^-7^ (3.123 x 10^-7^, 8.717 x 10^-6^) | 0.02 (0.015, 0.021) | 7.94 x 10^-3^ (0.009, 0.023) | 6.27 x 10^-3^ (0.004, 0.023) |
| **40** | 0.15 (0.001, 19.941) | 1.60 x 10^-6^ (5.001 x 10^-7^, 1.604 x 10^-4^) | 0.02 (0.016, 0.020) | 9.70 x 10^-3^ (0.007, 0.016) | 9.90 x 10^-3^ (0.001, 0.015) |
| **45** | 0.27 (0.016, 19.940) | 2.37 x 10^-6^ (4.982 x 10^-7^, 1.555 x 10^-4^) | 0.02 (0.017, 0.019) | 0.01 (0.008, 0.015) | 0.01 (0.002, 0.015) |
| **50** | 0.38 (0.030, 19.945) | 2.76 x 10^-6^ (4.362 x 10^-7^, 1.559 x 10^-4^) | 0.02 (0.017, 0.019) | 0.01 (0.008, 0.014) | 0.01 (0.003, 0.015) |
| **55** | 0.89 (0.038, 19.946) | 5.83 x 10^-6^ (5.319 x 10^-7^, 1.448 x 10^-4^) | 0.02 (0.017, 0.020) | 0.01 (0.008, 0.014) | 0.01 (0.003, 0.014) |
| **60** | 1.59 (0.075, 19.948) | 1.12 x 10^-5^ (6.945 x 10^-7^, 1.690 x 10^-4^) | 0.02 (0.017, 0.020) | 0.01 (0.009, 0.014) | 0.01 (0.005, 0.014) |
| **65** | 1.86 (0.150, 19.948) | 1.44 x 10^-5^ (1.101 x 10^-6^, 1.811 x 10^-4^) | 0.02 (0.018, 0.020) | 0.01 (0.011, 0.015) | 0.01 (0.008, 0.015) |
| **70** | 1.92 (0.124, 19.947) | 1.59 x 10^-5^ (9.879 x 10^-7^, 1.832 x 10^-4^) | 0.02 (0.018, 0.020) | 0.01 (0.010, 0.015) | 0.01 (0.008, 0.015) |
| **75** | 2.04 (0.137, 19.947) | 2.67 x 10^-5^ (1.570 x 10^-6^, 2.905 x 10^-4^) | 0.02 (0.018, 0.020) | 0.01 (0.010, 0.015) | 0.01 (0.008, 0.016) |
| **80** | 2.25 (0.158, 19.947) | 6.26 x 10^-5^ (3.307 x 10^-6^, 6.144 x 10^-4^) | 0.02 (0.019, 0.023) | 0.01 (0.010, 0.017) | 0.02 (0.008, 0.018) |
| **85** | 0.12 (0.024, 19.927) | 5.13 x 10^-6^ (2.074 x 10^-6^, 6.240 x 10^-4^) | 0.02 (0.021, 0.027) | 0.01 (0.007, 0.020) | 0.01 (-0.001, 0.022) |
| **90** | 0.13 (0.018, 19.941) | 6.27 x 10^-6^ (2.477 x 10^-6^, 7.015 x 10^-4^) | 0.03 (0.005, 0.023) | 0.01 (0.005, 0.023) | 0.02 (-0.003, 0.025) |
| **95** | 0.13 (1.975 x 10^-8^, 19.994) | 3.45 x 10^-6^ (1.300 x 10^-6^, 4.158 x 10^-4^) | 0.04 (0.034, 0.039) | 0.02 (0.014, 0.029) | 0.02 (0.005, 0.030) |
|  | **Fibular SMA_CC_** | | | | |
| **5** | 0.56 (0.054, 19.947) | 1.76 x 10^-5^ (2.457 x 10^-6^, 6.798 x 10^-4^) | 0.03 (0.029, 0.033) | 0.01 (0.005, 0.018) | 0.02 (-0.001, 0.020) |
| **10** | 2.39 x 10^-9^ (2.061 x 10^-9^, 0.730) | 1.29 x 10^-6^ (7.289 x 10^-7^, 9.762 x 10^-6^) | 0.02 (0.019, 0.028) | 4.14 x 10^-3^ (0.012, 0.036) | 2.69 x 10^-3^ (0.007, 0.040) |
| **15** | 3.83 x 10^-6^ (2.061 x 10^-9^, 0.608) | 5.34 x 10^-7^ (7.289 x 10^-7^, 9.762 x 10^-6^) | 0.02 (0.017, 0.022) | 6.26 x 10^-3^ (0.012, 0.026) | 5.55 x 10^-3^ (0.009, 0.029) |
| **20** | 1.66 x 10^-5^ (2.061 x 10^-9^, 0.616) | 3.62 x 10^-7^ (1.985 x 10^-7^, 2.114 x 10^-6^) | 0.02 (0.017, 0.021) | 7.48 x 10^-3^ (0.013, 0.024) | 9.00 x 10^-3^ (0.011, 0.027) |
| **25** | 0.09 (0.013, 19.925) | 8.85 x 10^-7^ (3.766 x 10^-7^, 1.153 x 10^-4^) | 0.02 (0.016, 0.019) | 9.41 x 10^-3^ (0.007, 0.015) | 0.01 (0.007, 0.017) |
| **30** | 0.15 (0.026, 19.926) | 9.65 x 10^-7^ (3.653 x 10^-7^, 9.287 x 10^-5^) | 0.02 (0.017, 0.019) | 0.01 (0.008, 0.015) | 0.01 (0.007, 0.017) |
| **35** | 0.13 (0.026, 19.918) | 9.55 x 10^-7^ (3.668 x 10^-7^, 1.071 x 10^-4^) | 0.02 (0.017, 0.019) | 0.01 (0.008, 0.015) | 0.01 (0.008, 0.015) |
| **40** | 0.13 (0.022, 19.939) | 1.04 x 10^-6^ (3.905 x 10^-7^, 1.200 x 10^-4^) | 0.02 (0.017, 0.020) | 0.01 (0.009, 0.015) | 0.01 (0.006, 0.016) |
| **45** | 0.07 (0.009, 19.931) | 7.10 x 10^-7^ (3.158 x 10^-7^, 1.077 x 10^-4^) | 0.02 (0.017, 0.020) | 0.01 (0.009, 0.016) | 0.01 (0.005, 0.017) |
| **50** | 0.11 (0.015, 19.937) | 8.46 x 10^-7^ (3.500 x 10^-7^, 1.050 x 10^-4^) | 0.02 (0.018, 0.020) | 0.01 (0.009, 0.016) | 0.01 (0.004, 0.016) |
| **55** | 0.20 (0.043, 19.945) | 1.29 x 10^-6^ (3.984 x 10^-7^, 1.086 x 10^-4^) | 0.02 (0.018, 0.020) | 0.01 (0.009, 0.015) | 0.01 (0.006, 0.016) |
| **60** | 0.33 (0.057, 19.946) | 1.96 x 10^-6^ (4.661 x 10^-7^, 1.101 x 10^-4^) | 0.02 (0.018, 0.020) | 0.01 (0.010, 0.015) | 0.01 (0.006, 0.016) |
| **65** | 0.41 (0.098, 19.944) | 2.51 x 10^-6^ (5.908 x 10^-7^, 1.179 x 10^-4^) | 0.02 (0.018, 0.020) | 0.01 (0.010, 0.015) | 0.01 (0.009, 0.016) |
| **70** | 0.48 (0.092, 19.945) | 3.16 x 10^-6^ (6.216 x 10^-7^, 1.463 x 10^-4^) | 0.02 (0.018, 0.020) | 0.01 (0.010, 0.015) | 0.01 (0.009, 0.017) |
| **75** | 1.00 (0.096, 19.945) | 8.70 x 10^-6^ (8.722 x 10^-7^, 1.794 x 10^-4^) | 0.02 (0.018, 0.020) | 0.01 (0.009, 0.015) | 0.02 (0.008, 0.016) |
| **80** | 0.23 (0.075, 19.945) | 2.76 x 10^-6^ (8.480 x 10^-7^, 2.075 x 10^-4^) | 0.02 (0.018, 0.021) | 0.01 (0.008, 0.015) | 0.01 (0.006, 0.016) |
| **85** | 0.08 (1.549 x 10^-8^, 19.939) | 2.31 x 10^-6^ (1.066 x 10^-6^, 3.493 x 10^-4^) | 0.02 (0.019, 0.025) | 8.76 x 10^-3^ (0.006, 0.019) | 0.01 (-0.004, 0.018) |
| **90** | 1.27 (0.097, 19.944) | 6.32 x 10^-5^ (5.084 x 10^-6^, 1.102 x 10^-3^) | 0.03 (0.028, 0.034) | 0.02 (0.008, 0.021) | 0.02 (0.008, 0.026) |
| **95** | 0.12 (0.020, 19.933) | 8.55 x 10^-6^ (3.681 x 10^-6^, 9.319 x 10^-4^) | 0.04 (0.037 0.046) | 0.02 (0.013, 0.034) | 0.03 (0.005, 0.037) |

**Table A6**. Parameter output for a multi-rate Brownian motion model with three means corresponding to fossorial+natatorial, scansorial, and generalist mustelids. σ^2^ _Fos+Nat_, σ^2^ _Sca_, and σ^2^ _Gen_ denote rates of stochastic evolution for fossorial and natatorial mustelids treated as a single group, scansorial mustelids, and generalist mustelids, respectively. θ_0_ represents the starting state of trait values. Parameter estimates are followed by 95% confidence limits in parentheses, which were generated by parametric bootstrapping. Note that the bootstrapping routine does not provide confidence intervals for group means (not shown).

| **%** | **σ^2^ _Fos+Nat_** | **σ^2^ _Sca_** | **σ^2^ _Gen_** | **θ_0_** |
| --- | --- | --- | --- | --- |
|  | **Femoral CSA** | | | |
| **5** | 2.32 x 10^-5^ (5.588 x 10^-6^, 3.079 x 10^-5^) | 8.98 x 10^-6^ (4.544 x 10^-6^, 4.687 x 10^-5^) | 1.24 x 10^-5^ (1.272 x 10^-6^, 4.973 x 10^-5^) | 0.10 (0.091, 0.115) |
| **10** | 3.95 x 10^-5^ (1.242 x 10^-5^, 6.412 x 10^-5^) | 8.02 x 10^-6^ (7.504 x 10^-6^, 9.037 x 10^-5^) | 5.57 x 10^-5^ (1.127 x 10^-6^, 1.037 x 10^-4^) | 0.10 (0.083, 0.119) |
| **15** | 3.60 x 10^-5^ (7.018 x 10^-6^, 3.827 x 10^-5^) | 5.35 x 10^-6^ (4.749 x 10^-6^, 4.824 x 10^-5^) | 5.13 x 10^-6^ (7.879 x 10^-7^, 5.772 x 10^-5^) | 0.08 (0.066, 0.100) |
| **20** | 2.72 x 10^-5^ (6.366 x 10^-6^, 3.379 x 10^-5^) | 4.19 x 10^-6^ (5.014 x 10^-6^, 4.471 x 10^-5^) | 1.68 x 10^-5^ (1.102 x 10^-6^, 5.216 x 10^-5^) | 0.08 (0.067, 0.096) |
| **25** | 2.71 x 10^-5^ (5.518 x 10^-6^, 3.275 x 10^-5^) | 2.23 x 10^-6^ (3.268 x 10^-6^, 3.748 x 10^-5^) | 1.09 x 10^-5^ (7.911 x 10^-7^, 4.536 x 10^-5^) | 0.07 (0.057, 0.090) |
| **30** | 3.34 x 10^-5^ (7.283 x 10^-6^, 3.631 x 10^-5^) | 1.93 x 10^-6^ (4.887 x 10^-6^, 4.650 x 10^-5^) | 6.27 x 10^-6^ (8.153 x 10^-7^, 5.691 x 10^-5^) | 0.07 (0.054, 0.089) |
| **35** | 2.56 x 10^-5^ (5.662 x 10^-6^, 2.697 x 10^-5^) | 1.80 x 10^-6^ (3.326 x 10^-6^, 3.492 x 10^-5^) | 5.37 x 10^-6^ (1.047 x 10^-6^, 3.834 x 10^-5^) | 0.07 (0.056, 0.084) |
| **40** | 2.38 x 10^-5^ (4.830 x 10^-6^, 2.468 x 10^-5^) | 2.07 x 10^-6^ (3.454 x 10^-6^, 3.316 x 10^-5^) | 5.29 x 10^-6^ (6.483 x 10^-7^, 3.721 x 10^-5^) | 0.07 (0.057, 0.083) |
| **45** | 2.15 x 10^-5^ (4.605 x 10^-6^, 2.257 x 10^-5^) | 1.63 x 10^-6^ (3.121 x 10^-6^, 2.931 x 10^-5^) | 7.25 x 10^-6^ (5.761 x 10^-7^, 3.587 x 10^-5^) | 0.07 (0.056, 0.081) |
| **50** | 1.96 x 10^-5^ (4.096 x 10^-6^, 2.144 x 10^-5^) | 1.77 x 10^-6^ (2.573 x 10^-6^, 2.569 x 10^-5^) | 6.22 x 10^-6^ (4.188 x 10^-7^, 3.127 x 10^-5^) | 0.07 (0.054, 0.081) |
| **55** | 1.85 x 10^-5^ (3.795 x 10^-6^, 2.119 x 10^-5^) | 1.96 x 10^-6^ (2.015 x 10^-6^, 2.523 x 10^-5^) | 5.42 x 10^-6^ (5.365 x 10^-7^, 2.960 x 10^-5^) | 0.07 (0.054, 0.080) |
| **60** | 1.72 x 10^-5^ (3.780 x 10^-6^, 1.924 x 10^-5^) | 2.34 x 10^-6^ (2.144 x 10^-6^, 2.349 x 10^-5^) | 4.48 x 10^-6^ (4.688 x 10^-7^, 2.804 x 10^-5^) | 0.07 (0.055, 0.080) |
| **65** | 1.79 x 10^-5^ (3.982 x 10^-6^, 2.026 x 10^-5^) | 2.87 x 10^-6^ (2.659 x 10^-6^, 2.492 x 10^-5^) | 3.36 x 10^-6^ (4.893 x 10^-7^, 2.699 x 10^-5^) | 0.07 (0.055, 0.080) |
| **70** | 2.30 x 10^-5^ (4.875 x 10^-6^, 2.594 x 10^-5^) | 3.30 x 10^-6^ (3.087 x 10^-6^, 2.887 x 10^-5^) | 3.02 x 10^-6^ (8.036 x 10^-7^, 3.647 x 10^-5^) | 0.07 (0.054, 0.085) |
| **75** | 2.60 x 10^-5^ (5.163 x 10^-6^, 3.018 x 10^-5^) | 3.94 x 10^-6^ (2.727 x 10^-6^, 3.558 x 10^-5^) | 3.08 x 10^-6^ (4.672 x 10^-7^, 4.110 x 10^-5^) | 0.07 (0.054, 0.087) |
| **80** | 3.61 x 10^-5^ (7.770 x 10^-6^, 4.064 x 10^-5^) | 4.87 x 10^-6^ (4.441 x 10^-6^, 4.545 x 10^-5^) | 5.79 x 10^-6^ (1.039 x 10^-6^, 6.294 x 10^-5^) | 0.07 (0.057, 0.096) |
| **85** | 1.28 x 10^-4^ (2.174 x 10^-5^, 1.262 x 10^-4^) | 1.01 x 10^-5^ (1.934 x 10^-5^, 1.730 x 10^-4^) | 4.69 x 10^-6^ (4.386 x 10^-6^, 2.166 x 10^-5^) | 0.08 (0.073, 0.115) |
| **90** | 6.75 x 10^-5^ (1.614 x 10^-5^, 8.991 x 10^-5^) | 1.49 x 10^-5^ (1.611 x 10^-5^, 1.463 x 10^-4^) | 4.72 x 10^-5^ (3.979 x 10^-6^, 1.536 x 10^-4^) | 0.12 (0.107, 0.139) |
| **95** | 1.42 x 10^-5^ (4.682 x 10^-6^, 2.371 x 10^-5^) | 1.75 x 10^-5^ (3.424 x 10^-6^, 3.119 x 10^-5^) | 5.99 x 10^-6^ (4.732 x 10^-7^, 3.655 x 10^-5^) | 0.11 (0.097, 0.120) |
|  | **Femoral SMA_ML_** | | | |
| **5** | 3.33 x 10^-6^ (1.235 x 10^-6^, 7.601 x 10^-6^) | 2.82 x 10^-6^ (1.260 x 10^-6^, 1.326 x 10^-5^) | 9.23 x 10^-6^ (2.749 x 10^-7^, 1.435 x 10^-5^) | 0.06 (0.055, 0.063) |
| **10** | 9.24 x 10^-6^ (3.592 x 10^-6^, 2.259 x 10^-5^) | 3.62 x 10^-6^ (3.824 x 10^-6^, 3.754 x 10^-5^) | 2.97 x 10^-5^ (6.453 x 10^-7^, 3.727 x 10^-5^) | 0.06 (0.050, 0.065) |
| **15** | 6.38 x 10^-6^ (1.609 x 10^-6^, 8.567 x 10^-6^) | 2.29 x 10^-6^ (1.036 x 10^-6^, 9.815 x 10^-6^) | 2.39 x 10^-6^ (1.368 x 10^-7^, 1.244 x 10^-5^) | 0.05 (0.038, 0.056) |
| **20** | 5.97 x 10^-6^ (1.642 x 10^-6^, 8.973 x 10^-6^) | 1.45 x 10^-6^ (1.240 x 10^-6^, 1.133 x 10^-5^) | 7.77 x 10^-6^ (2.508 x 10^-7^, 1.463 x 10^-5^) | 0.05 (0.040, 0.056) |
| **25** | 6.93 x 10^-6^ (1.701 x 10^-6^, 9.281 x 10^-6^) | 1.09 x 10^-6^ (1.364 x 10^-6^, 1.274 x 10^-5^) | 6.00 x 10^-6^ (5.479 x 10^-7^, 1.614 x 10^-5^) | 0.05 (0.039, 0.053) |
| **30** | 6.12 x 10^-6^ (1.476 x 10^-6^, 7.942 x 10^-6^) | 8.49 x 10^-7^ (1.144 x 10^-6^, 1.101 x 10^-5^) | 4.61 x 10^-6^ (2.041 x 10^-7^, 1.299 x 10^-5^) | 0.04 (0.037, 0.050) |
| **35** | 5.01 x 10^-6^ (1.253 x 10^-6^, 6.438 x 10^-6^) | 6.77 x 10^-7^ (8.622 x 10^-7^, 8.604 x 10^-6^) | 3.51 x 10^-6^ (1.991 x 10^-7^, 1.089 x 10^-5^) | 0.04 (0.035, 0.047) |
| **40** | 5.08 x 10^-6^ (1.230 x 10^-6^, 6.096 x 10^-6^) | 6.34 x 10^-7^ (8.401 x 10^-7^, 8.052 x 10^-6^) | 2.50 x 10^-6^ (1.818 x 10^-7^, 9.071 x 10^-6^) | 0.04 (0.034, 0.046) |
| **45** | 5.53 x 10^-6^ (1.196 x 10^-6^, 6.496 x 10^-6^) | 6.12 x 10^-7^ (7.180 x 10^-7^, 8.596 x 10^-6^) | 2.30 x 10^-6^ (1.953 x 10^-7^, 9.817 x 10^-6^) | 0.04 (0.033, 0.048) |
| **50** | 6.11 x 10^-6^ (1.220 x 10^-6^, 6.566 x 10^-6^) | 7.23 x 10^-7^ (8.396 x 10^-7^, 7.928 x 10^-6^) | 1.18 x 10^-6^ (1.897 x 10^-7^, 9.590 x 10^-6^) | 0.04 (0.033, 0.048) |
| **55** | 7.03 x 10^-6^ (1.494 x 10^-6^, 7.469 x 10^-6^) | 9.37 x 10^-7^ (7.939 x 10^-7^, 8.817 x 10^-6^) | 6.00 x 10^-7^ (2.025 x 10^-7^, 1.126 x 10^-6^) | 0.04 (0.033, 0.048) |
| **60** | 7.44 x 10^-6^ (1.346 x 10^-6^, 7.983 x 10^-6^) | 1.25 x 10^-6^ (9.636 x 10^-7^, 9.873 x 10^-6^) | 3.94 x 10^-7^ (2.484 x 10^-7^, 1.144 x 10^-5^) | 0.04 (0.033, 0.049) |
| **65** | 7.83 x 10^-6^ (1.639 x 10^-6^, 8.718 x 10^-6^) | 1.68 x 10^-6^ (1.059 x 10^-6^, 1.169 x 10^-6^) | 6.56 x 10^-7^ (2.472 x 10^-7^, 1.301 x 10^-5^) | 0.04 (0.035, 0.049) |
| **70** | 9.88 x 10^-6^ (2.181 x 10^-6^, 1.159 x 10^-5^) | 1.69 x 10^-6^ (1.345 x 10^-6^, 1.481 x 10^-5^) | 1.12 x 10^-6^ (3.134 x 10^-7^, 1.749 x 10^-5^) | 0.04 (0.034, 0.052) |
| **75** | 1.11 x 10^-5^ (2.159 x 10^-6^, 1.234 x 10^-5^) | 1.87 x 10^-6^ (1.568 x 10^-6^, 1.693 x 10^-5^) | 1.77 x 10^-6^ (5.959 x 10^-7^, 1.968 x 10^-5^) | 0.04 (0.036, 0.053) |
| **80** | 1.57 x 10^-5^ (2.950 x 10^-6^, 1.685 x 10^-5^) | 2.35 x 10^-6^ (2.572 x 10^-6^, 2.687 x 10^-5^) | 4.07 x 10^-6^ (5.600 x 10^-7^, 3.152 x 10^-5^) | 0.05 (0.042, 0.059) |
| **85** | 5.69 x 10^-5^ (9.761 x 10^-6^, 5.898 x 10^-5^) | 3.01 x 10^-6^ (9.976 x 10^-6^, 9.826 x 10^-5^) | 9.49 x 10^-6^ (2.880 x 10^-6^, 1.013 x 10^-4^) | 0.06 (0.052, 0.080) |
| **90** | 2.09 x 10^-5^ (4.899 x 10^-6^, 2.882 x 10^-5^) | 6.43 x 10^-6^ (4.348 x 10^-6^, 4.311 x 10^-5^) | 1.35 x 10^-5^ (1.089 x 10^-6^, 4.506 x 10^-5^) | 0.08 (0.072, 0.094) |
| **95** | 5.56 x 10^-6^ (1.190 x 10^-6^, 6.506 x 10^-5^) | 2.83 x 10^-6^ (8.378 x 10^-7^, 7.874 x 10^-6^) | 5.42 x 10^-7^ (1.558 x 10^-7^, 9.023 x 10^-5^) | 0.07 (0.066, 0.081) |
|  | **Femoral SMA_CC_** | | | |
| **5** | 1.70 x 10^-5^ (3.942 x 10^-6^, 2.378 x 10^-5^) | 2.74 x 10^-6^ (3.412 x 10^-6^, 3.649 x 10^-5^) | 1.51 x 10^-5^ (7.984 x 10^-7^, 4.192 x 10^-5^) | 0.09 (0.078, 0.096) |
| **10** | 2.45 x 10^-5^ (8.766 x 10^-6^, 5.383 x 10^-5^) | 3.30 x 10^-6^ (8.801 x 10^-6^, 8.011 x 10^-5^) | 6.69 x 10^-5^ (1.600 x 10^-6^, 9.875 x 10^-5^) | 0.09 (0.077, 0.103) |
| **15** | 2.86 x 10^-5^ (5.905 x 10^-6^, 3.289 x 10^-5^) | 2.50 x 10^-6^ (4.175 x 10^-6^, 3.752 x 10^-5^) | 9.84 x 10^-6^ (6.139 x 10^-7^, 4.647 x 10^-5^) | 0.07 (0.050, 0.084) |
| **20** | 1.43 x 10^-5^ (3.563 x 10^-6^, 1.719 x 10^-5^) | 2.11 x 10^-6^ (2.225 x 10^-6^, 2.338 x 10^-5^) | 8.64 x 10^-6^ (3.687 x 10^-6^, 2.873 x 10^-5^) | 0.06 (0.050, 0.072) |
| **25** | 1.69 x 10^-5^ (3.209 x 10^-6^, 1.755 x 10^-5^) | 1.23 x 10^-6^ (2.263 x 10^-6^, 2.261 x 10^-5^) | 3.45 x 10^-6^ (7.299 x 10^-7^, 2.768 x 10^-5^) | 0.05 (0.042, 0.065) |
| **30** | 1.52 x 10^-5^ (2.664 x 10^-6^, 1.472 x 10^-5^) | 8.41 x 10^-7^ (1.946 x 10^-6^, 1.831 x 10^-5^) | 1.24 x 10^-6^ (2.974 x 10^-7^, 2.298 x 10^-5^) | 0.05 (0.037, 0.059) |
| **35** | 9.41 x 10^-6^ (1.913 x 10^-6^, 9.704 x 10^-6^) | 5.65 x 10^-7^ (1.036 x 10^-6^, 1.096 x 10^-5^) | 8.55 x 10^-7^ (1.998 x 10^-7^, 1.362 x 10^-5^) | 0.04 (0.035, 0.054) |
| **40** | 8.05 x 10^-6^ (1.633 x 10^-6^, 8.029 x 10^-6^) | 4.59 x 10^-7^ (9.379 x 10^-7^, 9.945 x 10^-6^) | 9.80 x 10^-7^ (2.560 x 10^-7^, 1.167 x 10^-5^) | 0.04 (0.034, 0.052) |
| **45** | 7.28 x 10^-6^ (1.457 x 10^-6^, 7.448 x 10^-6^) | 3.91 x 10^-7^ (9.009 x 10^-7^, 9.265 x 10^-6^) | 1.34 x 10^-6^ (2.294 x 10^-7^, 1.125 x 10^-5^) | 0.04 (0.034, 0.051) |
| **50** | 7.49 x 10^-6^ (1.580 x 10^-6^, 7.781 x 10^-6^) | 4.60 x 10^-7^ (8.967 x 10^-7^, 9.318 x 10^-6^) | 1.26 x 10^-6^ (1.264 x 10^-7^, 1.082 x 10^-5^) | 0.04 (0.034, 0.051) |
| **55** | 8.10 x 10^-6^ (1.623 x 10^-6^, 8.351 x 10^-6^) | 6.77 x 10^-7^ (1.065 x 10^-6^, 1.058 x 10^-5^) | 1.47 x 10^-6^ (2.453 x 10^-7^, 1.220 x 10^-5^) | 0.04 (0.034, 0.052) |
| **60** | 9.57 x 10^-6^ (2.130 x 10^-6^, 1.052 x 10^-5^) | 1.17 x 10^-6^ (1.261 x 10^-6^, 1.297 x 10^-5^) | 1.52 x 10^-6^ (2.364 x 10^-7^, 1.530 x 10^-5^) | 0.04 (0.035, 0.055) |
| **65** | 1.18 x 10^-5^ (2.467 x 10^-6^, 1.349 x 10^-5^) | 1.84 x 10^-6^ (1.736 x 10^-6^, 1.457 x 10^-5^) | 1.63 x 10^-6^ (4.323 x 10^-7^, 1.817 x 10^-5^) | 0.05 (0.036, 0.057) |
| **70** | 1.58 x 10^-5^ (3.351 x 10^-6^, 1.724 x 10^-5^) | 2.39 x 10^-6^ (2.184 x 10^-6^, 1.982 x 10^-5^) | 1.97 x 10^-6^ (2.555 x 10^-7^, 2.562 x 10^-5^) | 0.05 (0.037, 0.062) |
| **75** | 1.77 x 10^-5^ (3.807 x 10^-6^, 1.950 x 10^-5^) | 3.26 x 10^-6^ (2.068 x 10^-6^, 2.258 x 10^-5^) | 1.94 x 10^-6^ (3.226 x 10^-7^, 2.824 x 10^-5^) | 0.05 (0.039, 0.065) |
| **80** | 2.04 x 10^-5^ (4.089 x 10^-6^, 2.228 x 10^-5^) | 4.40 x 10^-6^ (2.574 x 10^-6^, 2.827 x 10^-5^) | 4.37 x 10^-6^ (5.877 x 10^-7^, 3.179 x 10^-5^) | 0.06 (0.044, 0.073) |
| **85** | 5.01 x 10^-5^ (9.339 x 10^-6^, 5.350 x 10^-5^) | 6.91 x 10^-6^ (8.006 x 10^-6^, 8.168 x 10^-5^) | 8.62 x 10^-6^ (2.856 x 10^-6^, 9.712 x 10^-5^) | 0.07 (0.058, 0.087) |
| **90** | 1.89 x 10^-5^ (5.634 x 10^-6^, 3.178 x 10^-5^) | 6.74 x 10^-6^ (4.758 x 10^-6^, 5.026 x 10^-5^) | 3.10 x 10^-5^ (1.163 x 10^-6^, 5.455 x 10^-5^) | 0.09 (0.078, 0.102) |
| **95** | 6.86 x 10^-6^ (2.071 x 10^-6^, 1.078 x 10^-5^) | 5.11 x 10^-6^ (1.147 x 10^-6^, 1.197 x 10^-5^) | 3.73 x 10^-6^ (2.974 x 10^-7^, 1.485 x 10^-5^) | 0.08 (0.073, 0.091) |
|  | **Tibial CSA** | | | |
| **5** | 3.67 x 10^-5^ (6.142 x 10^-6^, 4.935 x 10^-5^) | 2.04 x 10^-5^ (7.523 x 10^-6^, 9.759 x 10^-5^) | 6.42 x 10^-6^ (2.181 x 10^-6^, 9.589 x 10^-5^) | 0.12 (0.112, 0.128) |
| **10** | 2.99 x 10^-5^ (8.412 x 10^-6^, 4.721 x 10^-5^) | 2.23 x 10^-5^ (5.444 x 10^-6^, 6.507 x 10^-5^) | 1.90 x 10^-5^ (2.000 x 10^-6^, 7.884 x 10^-5^) | 0.11 (0.092, 0.123) |
| **15** | 1.08 x 10^-5^ (2.901 x 10^-6^, 1.637 x 10^-5^) | 8.15 x 10^-6^ (1.627 x 10^-6^, 1.880 x 10^-5^) | 6.49 x 10^-6^ (2.335 x 10^-7^, 2.352 x 10^-5^) | 0.08 (0.074, 0.098) |
| **20** | 7.37 x 10^-6^ (2.231 x 10^-6^, 1.243 x 10^-5^) | 5.12 x 10^-6^ (1.956 x 10^-6^, 1.911 x 10^-5^) | 8.72 x 10^-6^ (6.003 x 10^-7^, 2.581 x 10^-5^) | 0.08 (0.068, 0.083) |
| **25** | 5.63 x 10^-6^ (1.665 x 10^-6^, 9.798 x 10^-6^) | 5.01 x 10^-6^ (1.511 x 10^-6^, 1.388 x 10^-5^) | 6.13 x 10^-6^ (3.562 x 10^-7^, 1.839 x 10^-5^) | 0.07 (0.066, 0.080) |
| **30** | 5.25 x 10^-6^ (1.482 x 10^-6^, 8.477 x 10^-6^) | 4.10 x 10^-6^ (1.255 x 10^-6^, 1.207 x 10^-5^) | 4.79 x 10^-6^ (4.528 x 10^-7^, 1.679 x 10^-5^) | 0.07 (0.065, 0.078) |
| **35** | 5.43 x 10^-6^ (1.421 x 10^-6^, 9.800 x 10^-6^) | 3.97 x 10^-6^ (1.537 x 10^-6^, 1.430 x 10^-5^) | 6.31 x 10^-6^ (5.025 x 10^-7^, 2.205 x 10^-5^) | 0.07 (0.064, 0.075) |
| **40** | 6.00 x 10^-6^ (1.757 x 10^-6^, 9.139 x 10^-6^) | 3.45 x 10^-6^ (1.321 x 10^-6^, 1.452 x 10^-5^) | 6.43 x 10^-6^ (4.394 x 10^-7^, 2.147 x 10^-5^) | 0.07 (0.063, 0.075) |
| **45** | 7.04 x 10^-6^ (1.926 x 10^-6^, 1.110 x 10^-5^) | 3.53 x 10^-6^ (1.929 x 10^-6^, 1.634 x 10^-5^) | 6.63 x 10^-6^ (6.406 x 10^-7^, 2.352 x 10^-5^) | 0.07 (0.063, 0.075) |
| **50** | 7.96 x 10^-6^ (1.963 x 10^-6^, 1.152 x 10^-5^) | 3.35 x 10^-6^ (1.846 x 10^-6^, 1.627 x 10^-5^) | 6.40 x 10^-6^ (5.683 x 10^-7^, 2.394 x 10^-5^) | 0.07 (0.063, 0.076) |
| **55** | 8.48 x 10^-6^ (2.028 x 10^-6^, 1.257 x 10^-5^) | 3.05 x 10^-6^ (1.821 x 10^-6^, 1.755 x 10^-5^) | 7.20 x 10^-6^ (3.863 x 10^-7^, 2.483 x 10^-5^) | 0.07 (0.062, 0.076) |
| **60** | 8.01 x 10^-6^ (1.981 x 10^-6^, 1.103 x 10^-5^) | 3.74 x 10^-6^ (1.668 x 10^-6^, 1.541 x 10^-5^) | 6.78 x 10^-6^ (3.508 x 10^-7^, 1.984 x 10^-5^) | 0.07 (0.060, 0.075) |
| **65** | 6.58 x 10^-6^ (1.767 x 10^-6^, 9.079 x 10^-6^) | 4.22 x 10^-6^ (1.036 x 10^-6^, 1.172 x 10^-5^) | 4.65 x 10^-6^ (2.910 x 10^-7^, 1.334 x 10^-5^) | 0.07 (0.058, 0.075) |
| **70** | 5.21 x 10^-6^ (1.474 x 10^-6^, 7.946 x 10^-6^) | 4.25 x 10^-6^ (1.118 x 10^-6^, 1.001 x 10^-5^) | 4.09 x 10^-6^ (1.640 x 10^-7^, 1.262 x 10^-5^) | 0.07 (0.059, 0.073) |
| **75** | 5.45 x 10^-6^ (1.642 x 10^-6^, 8.677 x 10^-6^) | 5.64 x 10^-6^ (1.041 x 10^-6^, 1.167 x 10^-5^) | 3.71 x 10^-6^ (1.978 x 10^-7^, 1.476 x 10^-5^) | 0.07 (0.059, 0.073) |
| **80** | 6.65 x 10^-6^ (1.875 x 10^-6^, 9.812 x 10^-6^) | 6.74 x 10^-6^ (1.393 x 10^-6^, 1.372 x 10^-5^) | 3.88 x 10^-6^ (3.781 x 10^-7^, 1.602 x 10^-5^) | 0.07 (0.061, 0.077) |
| **85** | 1.33 x 10^-5^ (3.373 x 10^-6^, 1.831 x 10^-5^) | 7.80 x 10^-6^ (2.650 x 10^-6^, 2.574 x 10^-5^) | 5.23 x 10^-6^ (4.995 x 10^-7^, 3.390 x 10^-5^) | 0.08 (0.065, 0.084) |
| **90** | 2.03 x 10^-5^ (5.827 x 10^-6^, 3.072 x 10^-5^) | 1.60 x 10^-5^ (4.724 x 10^-6^, 4.648 x 10^-5^) | 1.33 x 10^-5^ (1.187 x 10^-6^, 5.634 x 10^-5^) | 0.09 (0.077, 0.099) |
| **95** | 3.19 x 10^-5^ (6.151 x 10^-6^, 5.326 x 10^-5^) | 2.03 x 10^-5^ (7.879 x 10^-6^, 1.104 x 10^-4^) | 3.95 x 10^-5^ (2.131 x 10^-6^, 8.112 x 10^-5^) | 0.07 (0.061, 0.078) |
|  | **Tibial SMA_ML_** | | | |
| **5** | 3.11 x 10^-6^ (9.092 x 10^-7^, 6.455 x 10^-6^) | 5.13 x 10^-6^ (1.014 x 10^-6^, 1.135 x 10^-5^) | 2.92 x 10^-6^ (5.104 x 10^-7^, 1.526 x 10^-5^) | 0.08 (0.074, 0.082) |
| **10** | 1.17 x 10^-5^ (4.163 x 10^-6^, 2.459 x 10^-5^) | 9.80 x 10^-6^ (3.471 x 10^-6^, 3.320 x 10^-5^) | 2.37 x 10^-5^ (9.300 x 10^-7^, 4.074 x 10^-5^) | 0.08 (0.067, 0.088) |
| **15** | 5.93 x 10^-6^ (1.856 x 10^-6^, 9.295 x 10^-6^) | 3.87 x 10^-6^ (1.175 x 10^-6^, 1.118 x 10^-5^) | 7.20 x 10^-6^ (2.374 x 10^-7^, 1.455 x 10^-5^) | 0.07 (0.056, 0.075) |
| **20** | 4.04 x 10^-6^ (1.369 x 10^-6^, 6.996 x 10^-6^) | 2.59 x 10^-6^ (1.001 x 10^-6^, 9.026 x 10^-6^) | 5.84 x 10^-6^ (1.249 x 10^-7^, 1.047 x 10^-5^) | 0.06 (0.050, 0.066) |
| **25** | 3.39 x 10^-6^ (9.693 x 10^-7^, 5.383 x 10^-6^) | 2.22 x 10^-6^ (6.957 x 10^-7^, 6.792 x 10^-6^) | 3.61 x 10^-6^ (1.782 x 10^-7^, 8.556 x 10^-6^) | 0.05 (0.048, 0.061) |
| **30** | 2.82 x 10^-6^ (7.108 x 10^-7^, 4.060 x 10^-6^) | 1.84 x 10^-6^ (5.170 x 10^-7^, 4.834 x 10^-6^) | 2.32 x 10^-6^ (8.409 x 10^-8^, 6.161 x 10^-6^) | 0.05 (0.046, 0.058) |
| **35** | 2.39 x 10^-6^ (7.047 x 10^-7^, 3.901 x 10^-6^) | 1.45 x 10^-6^ (4.854 x 10^-7^, 5.036 x 10^-6^) | 3.41 x 10^-6^ (1.237 x 10^-7^, 6.212 x 10^-6^) | 0.05 (0.044, 0.054) |
| **40** | 2.38 x 10^-6^ (7.224 x 10^-7^, 4.159 x 10^-6^) | 1.26 x 10^-6^ (5.281 x 10^-7^, 5.613 x 10^-6^) | 3.73 x 10^-6^ (1.667 x 10^-7^, 6.931 x 10^-6^) | 0.05 (0.042, 0.051) |
| **45** | 2.37 x 10^-6^ (7.120 x 10^-7^, 4.004 x 10^-6^) | 1.21 x 10^-6^ (6.306 x 10^-7^, 6.460 x 10^-6^) | 3.79 x 10^-6^ (1.511 x 10^-7^, 7.645 x 10^-6^) | 0.05 (0.040, 0.049) |
| **50** | 2.45 x 10^-6^ (7.408 x 10^-7^, 4.304 x 10^-6^) | 1.21 x 10^-6^ (7.031 x 10^-7^, 6.868 x 10^-6^) | 3.84 x 10^-6^ (1.724 x 10^-7^, 9.022 x 10^-6^) | 0.04 (0.039, 0.047) |
| **55** | 2.62 x 10^-6^ (7.661 x 10^-7^, 4.388 x 10^-6^) | 1.09 x 10^-6^ (7.249 x 10^-7^, 6.979 x 10^-6^) | 3.78 x 10^-6^ (1.695 x 10^-7^, 8.836 x 10^-6^) | 0.04 (0.037, 0.045) |
| **60** | 2.42 x 10^-6^ (7.076 x 10^-7^, 4.262 x 10^-6^) | 1.28 x 10^-6^ (6.282 x 10^-7^, 6.960 x 10^-6^) | 3.53 x 10^-6^ (3.202 x 10^-7^, 9.235 x 10^-6^) | 0.04 (0.036, 0.043) |
| **65** | 2.13 x 10^-6^ (7.120 x 10^-7^, 3.756 x 10^-6^) | 1.53 x 10^-6^ (6.055 x 10^-7^, 5.579 x 10^-6^) | 2.89 x 10^-6^ (1.809 x 10^-7^, 7.290 x 10^-6^) | 0.04 (0.035, 0.043) |
| **70** | 1.84 x 10^-6^ (5.996 x 10^-7^, 3.593 x 10^-6^) | 1.91 x 10^-6^ (5.008 x 10^-7^, 5.434 x 10^-6^) | 2.22 x 10^-6^ (1.830 x 10^-7^, 6.972 x 10^-6^) | 0.04 (0.035, 0.042) |
| **75** | 1.83 x 10^-6^ (6.336 x 10^-7^, 4.007 x 10^-6^) | 2.79 x 10^-6^ (6.015 x 10^-7^, 5.947 x 10^-6^) | 1.93 x 10^-6^ (2.074 x 10^-7^, 7.849 x 10^-6^) | 0.04 (0.036, 0.042) |
| **80** | 2.36 x 10^-6^ (7.312 x 10^-7^, 4.776 x 10^-6^) | 3.54 x 10^-6^ (7.769 x 10^-7^, 7.448 x 10^-6^) | 1.81 x 10^-6^ (3.593 x 10^-7^, 1.001 x 10^-6^) | 0.04 (0.037, 0.045) |
| **85** | 5.03 x 10^-6^ (1.407 x 10^-6^, 8.235 x 10^-6^) | 4.51 x 10^-6^ (1.138 x 10^-6^, 1.321 x 10^-5^) | 2.55 x 10^-6^ (6.898 x 10^-7^, 1.886 x 10^-5^) | 0.05 (0.042, 0.052) |
| **90** | 6.39 x 10^-6^ (1.946 x 10^-6^, 1.111 x 10^-5^) | 6.04 x 10^-6^ (1.565 x 10^-6^, 1.617 x 10^-5^) | 5.39 x 10^-6^ (5.882 x 10^-7^, 2.204 x 10^-5^) | 0.06 (0.050, 0.063) |
| **95** | 6.26 x 10^-6^ (1.513 x 10^-6^, 1.044 x 10^-5^) | 4.39 x 10^-6^ (1.763 x 10^-6^, 2.264 x 10^-5^) | 7.89 x 10^-6^ (4.882 x 10^-7^, 1.940 x 10^-5^) | 0.05 (0.043, 0.051) |
|  | **Tibial SMA_CC_** | | | |
| **5** | 7.54 x 10^-6^ (2.319 x 10^-6^, 1.530 x 10^-5^) | 6.88 x 10^-6^ (2.549 x 10^-6^, 2.502 x 10^-5^) | 9.75 x 10^-6^ (1.149 x 10^-6^, 3.596 x 10^-5^) | 0.09 (0.084, 0.096) |
| **10** | 1.48 x 10^-5^ (4.253 x 10^-6^, 2.480 x 10^-5^) | 8.64 x 10^-6^ (3.446 x 10^-6^, 3.214 x 10^-5^) | 1.94 x 10^-6^ (4.001 x 10^-7^, 4.000 x 10^-5^) | 0.08 (0.068, 0.091) |
| **15** | 5.01 x 10^-6^ (1.490 x 10^-6^, 7.804 x 10^-6^) | 3.81 x 10^-6^ (9.117 x 10^-7^, 9.243 x 10^-6^) | 6.11 x 10^-6^ (1.567 x 10^-7^, 1.117 x 10^-5^) | 0.06 (0.050, 0.068) |
| **20** | 3.23 x 10^-6^ (9.361 x 10^-7^, 6.069 x 10^-6^) | 2.78 x 10^-6^ (9.327 x 10^-7^, 9.669 x 10^-6^) | 3.82 x 10^-6^ (3.775 x 10^-7^, 1.093 x 10^-5^) | 0.05 (0.042, 0.051) |
| **25** | 2.80 x 10^-6^ (7.286 x 10^-7^, 4.896 x 10^-6^) | 2.19 x 10^-6^ (7.123 x 10^-7^, 6.909 x 10^-6^) | 1.92 x 10^-6^ (1.541 x 10^-7^, 8.833 x 10^-6^) | 0.04 (0.039, 0.047) |
| **30** | 2.42 x 10^-6^ (6.174 x 10^-7^, 3.489 x 10^-6^) | 1.62 x 10^-6^ (5.582 x 10^-7^, 5.536 x 10^-6^) | 1.25 x 10^-6^ (2.963 x 10^-7^, 7.562 x 10^-6^) | 0.04 (0.037, 0.044) |
| **35** | 2.20 x 10^-6^ (4.910 x 10^-7^, 3.262 x 10^-6^) | 1.19 x 10^-6^ (4.933 x 10^-7^, 4.872 x 10^-6^) | 9.32 x 10^-7^ (1.683 x 10^-7^, 6.969 x 10^-6^) | 0.04 (0.036, 0.042) |
| **40** | 2.40 x 10^-6^ (5.000 x 10^-7^, 2.955 x 10^-6^) | 8.98 x 10^-7^ (4.233 x 10^-7^, 4.632 x 10^-6^) | 5.43 x 10^-7^ (1.765 x 10^-7^, 6.723 x 10^-6^) | 0.04 (0.035, 0.041) |
| **45** | 2.58 x 10^-6^ (4.951 x 10^-7^, 3.355 x 10^-6^) | 8.39 x 10^-7^ (5.087 x 10^-7^, 4.804 x 10^-6^) | 4.82 x 10^-7^ (1.182 x 10^-7^, 6.368 x 10^-6^) | 0.04 (0.034, 0.041) |
| **50** | 2.71 x 10^-6^ (6.302 x 10^-7^, 3.205 x 10^-6^) | 8.26 x 10^-7^ (4.570 x 10^-7^, 4.216 x 10^-6^) | 6.05 x 10^-7^ (9.888 x 10^-8^, 5.776 x 10^-6^) | 0.04 (0.034, 0.042) |
| **55** | 2.91 x 10^-6^ (5.997 x 10^-7^, 3.475 x 10^-6^) | 8.60 x 10^-7^ (4.134 x 10^-7^, 4.662 x 10^-6^) | 9.50 x 10^-7^ (5.876 x 10^-8^, 5.118 x 10^-6^) | 0.04 (0.034, 0.044) |
| **60** | 2.84 x 10^-6^ (6.276 x 10^-7^, 3.426 x 10^-6^) | 1.06 x 10^-6^ (4.094 x 10^-7^, 4.038 x 10^-6^) | 1.03 x 10^-6^ (7.428 x 10^-8^, 5.194 x 10^-6^) | 0.04 (0.034, 0.045) |
| **65** | 2.81 x 10^-6^ (6.326 x 10^-7^, 3.424 x 10^-6^) | 1.38 x 10^-6^ (4.266 x 10^-7^, 4.149 x 10^-6^) | 1.10 x 10^-6^ (1.119 x 10^-7^, 5.433 x 10^-6^) | 0.04 (0.035, 0.047) |
| **70** | 2.60 x 10^-6^ (6.953 x 10^-7^, 3.496 x 10^-6^) | 1.82 x 10^-6^ (4.638 x 10^-7^, 4.263 x 10^-6^) | 1.31 x 10^-6^ (1.034 x 10^-7^, 5.423 x 10^-6^) | 0.04 (0.036, 0.047) |
| **75** | 3.07 x 10^-6^ (8.223 x 10^-7^, 4.699 x 10^-6^) | 2.57 x 10^-6^ (6.557 x 10^-7^, 6.550 x 10^-6^) | 1.68 x 10^-6^ (1.461 x 10^-7^, 8.170 x 10^-6^) | 0.04 (0.038, 0.048) |
| **80** | 4.42 x 10^-6^ (1.179 x 10^-6^, 6.440 x 10^-6^) | 3.45 x 10^-6^ (1.032 x 10^-6^, 9.795 x 10^-6^) | 1.75 x 10^-6^ (3.685 x 10^-7^, 1.294 x 10^-6^) | 0.05 (0.041, 0.051) |
| **85** | 7.42 x 10^-6^ (1.761 x 10^-6^, 1.002 x 10^-5^) | 4.38 x 10^-6^ (1.395 x 10^-6^, 1.629 x 10^-5^) | 3.68 x 10^-6^ (7.949 x 10^-7^, 2.229 x 10^-5^) | 0.05 (0.046, 0.058) |
| **90** | 5.26 x 10^-6^ (1.672 x 10^-6^, 1.265 x 10^-5^) | 6.06 x 10^-6^ (2.218 x 10^-6^, 2.017 x 10^-5^) | 1.04 x 10^-5^ (7.428 x 10^-7^, 2.519 x 10^-5^) | 0.06 (0.056, 0.067) |
| **95** | 1.15 x 10^-5^ (2.361 x 10^-6^, 1.894 x 10^-5^) | 4.75 x 10^-6^ (3.285 x 10^-6^, 3.932 x 10^-5^) | 1.04 x 10^-5^ (5.283 x 10^-7^, 2.707 x 10^-5^) | 0.05 (0.040, 0.050) |
|  | **Fibular CSA** | | | |
| **5** | 6.69 x 10^-6^ (1.198 x 10^-6^, 8.462 x 10^-6^) | 2.36 x 10^-6^ (1.738 x 10^-6^, 1.620 x 10^-5^) | 4.34 x 10^-6^ (5.166 x 10^-7^, 1.599 x 10^-5^) | 0.05 (0.047, 0.054) |
| **10** | 3.10 x 10^-6^ (7.929 x 10^-7^, 3.748 x 10^-6^) | 9.21 x 10^-7^ (4.780 x 10^-7^, 4.281 x 10^-6^) | 1.17 x 10^-6^ (8.029 x 10^-8^, 5.429 x 10^-6^) | 0.04 (0.033, 0.044) |
| **15** | 1.66 x 10^-6^ (4.078 x 10^-7^, 2.184 x 10^-6^) | 6.07 x 10^-7^ (3.377 x 10^-7^, 3.416 x 10^-6^) | 8.90 x 10^-7^ (8.562 x 10^-8^, 3.700 x 10^-6^) | 0.03 (0.028, 0.035) |
| **20** | 1.04 x 10^-6^ (2.892 x 10^-7^, 1.695 x 10^-6^) | 6.08 x 10^-7^ (2.559 x 10^-7^, 2.508 x 10^-6^) | 1.19 x 10^-6^ (7.164 x 10^-8^, 2.909 x 10^-6^) | 0.03 (0.028, 0.034) |
| **25** | 6.94 x 10^-7^ (2.841 x 10^-7^, 1.658 x 10^-6^) | 6.81 x 10^-7^ (2.435 x 10^-7^, 2.610 x 10^-6^) | 1.94 x 10^-6^ (5.412 x 10^-8^, 2.969 x 10^-6^) | 0.03 (0.029, 0.034) |
| **30** | 9.27 x 10^-7^ (2.726 x 10^-7^, 1.452 x 10^-6^) | 5.20 x 10^-7^ (2.336 x 10^-7^, 2.139 x 10^-6^) | 1.26 x 10^-6^ (4.340 x 10^-8^, 2.430 x 10^-6^) | 0.03 (0.030, 0.035) |
| **35** | 1.18 x 10^-6^ (3.217 x 10^-7^, 1.865 x 10^-6^) | 5.28 x 10^-7^ (2.728 x 10^-7^, 2.816 x 10^-6^) | 1.19 x 10^-6^ (5.963 x 10^-8^, 3.265 x 10^-6^) | 0.03 (0.030, 0.035) |
| **40** | 1.32 x 10^-6^ (3.459 x 10^-7^, 1.991 x 10^-6^) | 5.95 x 10^-7^ (3.438 x 10^-7^, 3.151 x 10^-6^) | 1.25 x 10^-6^ (1.022 x 10^-7^, 3.966 x 10^-6^) | 0.03 (0.030, 0.035) |
| **45** | 1.29 x 10^-6^ (2.959 x 10^-7^, 1.943 x 10^-6^) | 5.61 x 10^-7^ (2.894 x 10^-7^, 2.704 x 10^-6^) | 8.84 x 10^-7^ (7.367 x 10^-8^, 3.875 x 10^-6^) | 0.03 (0.031, 0.036) |
| **50** | 1.08 x 10^-6^ (2.951 x 10^-7^, 1.839 x 10^-6^) | 6.61 x 10^-7^ (2.846 x 10^-7^, 2.863 x 10^-6^) | 8.91 x 10^-7^ (8.347 x 10^-8^, 3.881 x 10^-6^) | 0.03 (0.031, 0.036) |
| **55** | 1.05 x 10^-6^ (2.856 x 10^-7^, 1.561 x 10^-6^) | 5.49 x 10^-7^ (3.256 x 10^-7^, 2.672 x 10^-6^) | 9.12 x 10^-7^ (1.085 x 10^-7^, 3.451 x 10^-6^) | 0.03 (0.031, 0.036) |
| **60** | 1.20 x 10^-6^ (2.691 x 10^-7^, 1.820 x 10^-6^) | 5.41 x 10^-7^ (3.745 x 10^-7^, 2.993 x 10^-6^) | 9.03 x 10^-7^ (9.046 x 10^-8^, 4.091 x 10^-6^) | 0.03 (0.031, 0.036) |
| **65** | 1.60 x 10^-6^ (3.276 x 10^-7^, 2.175 x 10^-6^) | 5.94 x 10^-7^ (4.699 x 10^-7^, 4.009 x 10^-6^) | 1.38 x 10^-6^ (1.255 x 10^-7^, 4.535 x 10^-6^) | 0.03 (0.031, 0.035) |
| **70** | 1.85 x 10^-6^ (4.274 x 10^-7^, 2.587 x 10^-6^) | 6.90 x 10^-7^ (4.553 x 10^-7^, 4.611 x 10^-6^) | 1.56 x 10^-6^ (1.351 x 10^-7^, 5.362 x 10^-6^) | 0.03 (0.031, 0.035) |
| **75** | 2.72 x 10^-6^ (5.347 x 10^-7^, 3.555 x 10^-6^) | 9.45 x 10^-7^ (6.641 x 10^-7^, 6.282 x 10^-6^) | 1.89 x 10^-6^ (2.173 x 10^-7^, 7.985 x 10^-6^) | 0.03 (0.031, 0.036) |
| **80** | 4.66 x 10^-6^ (8.237 x 10^-7^, 5.452 x 10^-6^) | 8.25 x 10^-7^ (8.899 x 10^-7^, 1.026 x 10^-5^) | 1.34 x 10^-6^ (3.046 x 10^-7^, 1.062 x 10^-5^) | 0.03 (0.032, 0.038) |
| **85** | 5.81 x 10^-6^ (1.145 x 10^-6^, 6.562 x 10^-6^) | 9.51 x 10^-7^ (1.222 x 10^-6^, 1.060 x 10^-5^) | 7.65 x 10^-7^ (3.121 x 10^-7^, 1.227 x 10^-5^) | 0.04 (0.033, 0.043) |
| **90** | 1.06 x 10^-5^ (1.978 x 10^-6^, 1.231 x 10^-5^) | 1.77 x 10^-6^ (2.164 x 10^-6^, 2.128 x 10^-5^) | 1.61 x 10^-6^ (4.475 x 10^-7^, 2.345 x 10^-5^) | 0.05 (0.043, 0.053) |
| **95** | 9.22 x 10^-6^ (1.805 x 10^-6^, 1.044 x 10^-5^) | 2.67 x 10^-6^ (1.577 x 10^-6^, 1.505 x 10^-5^) | 1.06 x 10^-6^ (4.943 x 10^-7^, 2.004 x 10^-5^) | 0.06 (0.053, 0.066) |
|  | **Fibular SMA_ML_** | | | |
| **5** | 5.06 x 10^-6^ (1.016 x 10^-6^, 6.486 x 10^-6^) | 1.90 x 10^-6^ (1.298 x 10^-6^, 1.349 x 10^-5^) | 3.95 x 10^-6^ (2.055 x 10^-7^, 1.101 x 10^-5^) | 0.04 (0.035, 0.043) |
| **10** | 6.87 x 10^-6^ (1.254 x 10^-6^, 7.487 x 10^-6^) | 7.37 x 10^-7^ (1.138 x 10^-6^, 1.375 x 10^-5^) | 1.14 x 10^-6^ (3.037 x 10^-7^, 1.380 x 10^-5^) | 0.03 (0.022, 0.030) |
| **15** | 1.32 x 10^-6^ (2.876 x 10^-7^, 1.722 x 10^-6^) | 5.19 x 10^-7^ (3.254 x 10^-7^, 3.233 x 10^-6^) | 6.19 x 10^-7^ (1.085 x 10^-7^, 3.508 x 10^-6^) | 0.02 (0.017, 0.021) |
| **20** | 8.09 x 10^-7^ (1.804 x 10^-7^, 1.222 x 10^-6^) | 3.35 x 10^-7^ (1.972 x 10^-7^, 1.900 x 10^-6^) | 4.22 x 10^-7^ (4.926 x 10^-8^, 2.069 x 10^-6^) | 0.02 (0.016, 0.019) |
| **25** | 6.11 x 10^-7^ (1.710 x 10^-7^, 9.680 x 10^-7^) | 3.09 x 10^-7^ (1.652 x 10^-7^, 1.652 x 10^-6^) | 7.84 x 10^-7^ (5.345 x 10^-8^, 2.030 x 10^-6^) | 0.02 (0.016, 0.019) |
| **30** | 7.67 x 10^-7^ (1.670 x 10^-7^, 8.357 x 10^-7^) | 1.72 x 10^-7^ (1.232 x 10^-7^, 1.096 x 10^-6^) | 2.18 x 10^-7^ (2.479 x 10^-8^, 1.235 x 10^-6^) | 0.02 (0.016, 0.020) |
| **35** | 7.99 x 10^-7^ (1.771 x 10^-7^, 8.990 x 10^-7^) | 1.64 x 10^-7^ (1.138 x 10^-7^, 1.082 x 10^-6^) | 2.56 x 10^-7^ (2.142 x 10^-8^, 1.386 x 10^-6^) | 0.02 (0.016, 0.021) |
| **40** | 8.43 x 10^-7^ (1.807 x 10^-7^, 1.023 x 10^-6^) | 2.06 x 10^-7^ (1.786 x 10^-7^, 1.662 x 10^-6^) | 2.66 x 10^-7^ (5.974 x 10^-8^, 2.080 x 10^-6^) | 0.02 (0.016, 0.019) |
| **45** | 7.67 x 10^-7^ (1.659 x 10^-7^, 9.539 x 10^-7^) | 2.38 x 10^-7^ (1.715 x 10^-7^, 1.576 x 10^-6^) | 1.88 x 10^-7^ (7.316 x 10^-8^, 2.112 x 10^-6^) | 0.02 (0.016, 0.019) |
| **50** | 7.08 x 10^-7^ (1.535 x 10^-7^, 9.606 x 10^-7^) | 2.80 x 10^-7^ (1.609 x 10^-7^, 1.600 x 10^-6^) | 2.26 x 10^-7^ (7.416 x 10^-8^, 1.991 x 10^-6^) | 0.02 (0.017, 0.020) |
| **55** | 8.09 x 10^-7^ (1.471 x 10^-7^, 9.168 x 10^-7^) | 2.61 x 10^-7^ (1.733 x 10^-7^, 1.750 x 10^-6^) | 1.45 x 10^-7^ (7.767 x 10^-8^, 2.147 x 10^-6^) | 0.02 (0.017, 0.020) |
| **60** | 1.08 x 10^-6^ (1.557 x 10^-7^, 1.151 x 10^-6^) | 2.86 x 10^-7^ (1.850 x 10^-7^, 1.978 x 10^-6^) | 8.50 x 10^-8^ (7.693 x 10^-8^, 2.317 x 10^-6^) | 0.02 (0.017, 0.020) |
| **65** | 1.33 x 10^-6^ (1.769 x 10^-7^, 1.266 x 10^-6^) | 4.00 x 10^-7^ (2.012 x 10^-7^, 2.349 x 10^-6^) | 1.43 x 10^-7^ (1.020 x 10^-7^, 2.487 x 10^-6^) | 0.02 (0.017, 0.020) |
| **70** | 1.27 x 10^-6^ (1.796 x 10^-7^, 1.338 x 10^-6^) | 4.79 x 10^-7^ (2.369 x 10^-7^, 2.443 x 10^-6^) | 1.43 x 10^-7^ (7.547 x 10^-8^, 2.543 x 10^-6^) | 0.02 (0.017, 0.020) |
| **75** | 1.77 x 10^-6^ (2.696 x 10^-7^, 2.031 x 10^-6^) | 7.95 x 10^-7^ (3.370 x 10^-7^, 3.740 x 10^-6^) | 2.47 x 10^-7^ (9.238 x 10^-8^, 3.768 x 10^-6^) | 0.02 (0.017, 0.021) |
| **80** | 4.07 x 10^-6^ (5.562 x 10^-7^, 4.546 x 10^-6^) | 7.36 x 10^-7^ (7.208 x 10^-7^, 8.273 x 10^-6^) | 3.58 x 10^-7^ (2.611 x 10^-7^, 6.944 x 10^-6^) | 0.02 (0.018, 0.023) |
| **85** | 3.61 x 10^-6^ (6.778 x 10^-7^, 4.135 x 10^-6^) | 7.65 x 10^-7^ (7.294 x 10^-7^, 7.118 x 10^-6^) | 2.91 x 10^-7^ (2.085 x 10^-7^, 7.633 x 10^-6^) | 0.02 (0.018, 0.027) |
| **90** | 4.20 x 10^-6^ (7.741 x 10^-7^, 4.908 x 10^-6^) | 9.51 x 10^-7^ (8.912 x 10^-7^, 8.852 x 10^-6^) | 4.98 x 10^-7^ (2.233 x 10^-7^, 9.129 x 10^-6^) | 0.03 (0.027, 0.034) |
| **95** | 1.56 x 10^-6^ (4.809 x 10^-7^, 2.830 x 10^-6^) | 1.61 x 10^-6^ (5.179 x 10^-7^, 4.362 x 10^-6^) | 9.30 x 10^-7^ (1.677 x 10^-7^, 5.872 x 10^-6^) | 004 (0.034, 0.039) |
|  | **Fibular SMA_CC_** | | | |
| **5** | 3.42 x 10^-6^ (5.757 x 10^-7^, 4.254 x 10^-6^) | 8.72 x 10^-7^ (8.867 x 10^-7^, 7.625 x 10^-6^) | 2.05 x 10^-6^ (4.112 x 10^-7^, 9.372 x 10^-6^) | 0.03 (0.028, 0.033) |
| **10** | 2.27 x 10^-6^ (4.134 x 10^-7^, 2.108 x 10^-6^) | 2.22 x 10^-7^ (2.525 x 10^-7^, 2.495 x 10^-6^) | 1.10 x 10^-7^ (3.821 x 10^-8^, 3.086 x 10^-6^) | 0.02 (0.020, 0.028) |
| **15** | 8.01 x 10^-7^ (1.716 x 10^-7^, 8.643 x 10^-7^) | 1.15 x 10^-7^ (1.115 x 10^-7^, 1.089 x 10^-6^) | 2.52 x 10^-7^ (2.678 x 10^-8^, 1.302 x 10^-6^) | 0.02 (0.017, 0.022) |
| **20** | 3.88 x 10^-7^ (1.154 x 10^-7^, 5.718 x 10^-7^) | 1.55 x 10^-7^ (6.643 x 10^-8^, 6.774 x 10^-7^) | 5.67 x 10^-7^ (9.266 x 10^-9^, 8.513 x 10^-7^) | 0.02 (0.016, 0.021) |
| **25** | 3.76 x 10^-7^ (1.327 x 10^-7^, 8.574 x 10^-7^) | 2.20 x 10^-7^ (1.577 x 10^-7^, 1.389 x 10^-6^) | 1.09 x 10^-6^ (3.702 x 10^-8^, 1.343 x 10^-7^) | 0.02 (0.016, 0.019) |
| **30** | 4.29 x 10^-7^ (1.142 x 10^-7^, 6.589 x 10^-7^) | 2.63 x 10^-7^ (1.123 x 10^-7^, 1.115 x 10^-6^) | 4.26 x 10^-7^ (2.179 x 10^-8^, 1.259 x 10^-6^) | 0.02 (0.016, 0.019) |
| **35** | 4.96 x 10^-7^ (1.224 x 10^-7^, 7.290 x 10^-7^) | 2.46 x 10^-7^ (1.059 x 10^-7^, 1.205 x 10^-6^) | 4.17 x 10^-7^ (3.598 x 10^-8^, 1.375 x 10^-6^) | 0.02 (0.017, 0.020) |
| **40** | 4.78 x 10^-7^ (1.374 x 10^-7^, 7.928 x 10^-7^) | 3.09 x 10^-7^ (1.340 x 10^-7^, 1.334 x 10^-6^) | 6.56 x 10^-7^ (2.812 x 10^-8^, 1.503 x 10^-6^) | 0.02 (0.017, 0.020) |
| **45** | 4.54 x 10^-7^ (1.378 x 10^-7^, 7.208 x 10^-7^) | 2.70 x 10^-7^ (1.189 x 10^-7^, 1.165 x 10^-6^) | 5.17 x 10^-7^ (3.036 x 10^-8^, 1.343 x 10^-6^) | 0.02 (0.017, 0.020) |
| **50** | 4.48 x 10^-7^ (1.265 x 10^-7^, 7.101 x 10^-7^) | 2.87 x 10^-7^ (1.236 x 10^-7^, 1.281 x 10^-6^) | 4.70 x 10^-7^ (3.487 x 10^-8^, 1.426 x 10^-6^) | 0.02 (0.017, 0.020) |
| **55** | 3.96 x 10^-7^ (1.053 x 10^-7^, 6.805 x 10^-7^) | 2.90 x 10^-7^ (1.282 x 10^-7^, 1.206 x 10^-6^) | 5.52 x 10^-7^ (3.995 x 10^-8^, 1.408 x 10^-6^) | 0.02 (0.018, 0.020) |
| **60** | 4.12 x 10^-7^ (1.207 x 10^-7^, 7.642 x 10^-7^) | 2.99 x 10^-7^ (1.545 x 10^-7^, 1.299 x 10^-6^) | 6.54 x 10^-7^ (3.082 x 10^-8^, 1.391 x 10^-6^) | 0.02 (0.017, 0.020) |
| **65** | 3.97 x 10^-7^ (1.203 x 10^-7^, 8.658 x 10^-7^) | 3.97 x 10^-7^ (1.401 x 10^-7^, 1.495 x 10^-6^) | 8.34 x 10^-7^ (4.042 x 10^-8^, 1.517 x 10^-6^) | 0.02 (0.017, 0.020) |
| **70** | 3.62 x 10^-7^ (1.374 x 10^-7^, 8.456 x 10^-7^) | 4.44 x 10^-7^ (1.656 x 10^-7^, 1.485 x 10^-6^) | 1.16 x 10^-6^ (3.446 x 10^-8^, 1.720 x 10^-6^) | 0.02 (0.017, 0.020) |
| **75** | 6.45 x 10^-7^ (1.809 x 10^-7^, 1.242 x 10^-6^) | 4.28 x 10^-7^ (2.453 x 10^-7^, 2.223 x 10^-6^) | 1.41 x 10^-6 7^ (6.213 x 10^-8^, 2.273 x 10^-6^) | 0.02 (0.017, 0.020) |
| **80** | 9.11 x 10^-7^ (2.200 x 10^-7^, 1.465 x 10^-6^) | 3.71 x 10^-7^ (2.373 x 10^-7^, 2.535 x 10^-6^) | 1.19 x 10^-6^ (8.234 x 10^-8^, 2.661 x 10^-6^) | 0.02 (0.017, 0.021) |
| **85** | 1.91 x 10^-6^ (3.730 x 10^-7^, 2.324 x 10^-6^) | 5.13 x 10^-7^ (4.298 x 10^-7^, 3.594 x 10^-6^) | 3.09 x 10^-7^ (8.402 x 10^-8^, 4.826 x 10^-6^) | 0.02 (0.019, 0.025) |
| **90** | 7.10 x 10^-6^ (1.083 x 10^-6^, 7.638 x 10^-6^) | 6.65 x 10^-7^ (1.285 x 10^-6^, 1.514 x 10^-5^) | 4.25 x 10^-7^ (3.252 x 10^-7^, 1.286 x 10^-5^) | 0.03 (0.026, 0.034) |
| **95** | 6.91 x 10^-6^ (1.248 x 10^-6^, 7.692 x 10^-6^) | 9.59 x 10^-7^ (1.195 x 10^-6^, 1.194 x 10^-5^) | 8.52 x 10^-7^ (3.637 x 10^-6^, 1.307 x 10^-5^) | 0.04 (0.036, 0.046) |
