# Supplementary material for: Functional Morphology and Morphological Diversification of Hind Limb Cross-Sectional Traits in Mustelid Mammals
Source: Integr Org Biol. 2020 Jan 8;2(1):obz032. doi: 10.1093/iob/obz032 (PMC7671153; doi:10.1093/iob/obz032)
Supplement: obz032_Supplementary_Data [file obz032_supplementary_data.zip › Tables S1-S3 Revised.docx]

**Table S1**. Akaike weights for trait diversification models fitted to femoral cross-sectional traits. Models fitted include group mean Brownian motion models with one, three, and four rates (BM1, BM3/BM3_r, and B4, respectively) and Ornstein-Uhlenbeck models with one, three, and four phenotypic optima (OU1, OU3/OU3_r, and OU4, respectively). BM3 and OU3 have their respective distinct rates and optima based upon three functional categories: scansoriality, natatoriality, and a third category combining fossorial and generalized mustelids. BM3_r and OU3_r have their respective weights and optima based upon three categories: scansoriality, generalist, and a third category combining natatorial and fossorial mustelids. BM4 and OU4 have their respective rates and optima based upon each of the four locomotor categories occurring within extant mustelids: fossorial, generalized, natatorial, and scansorial. Also fitted was an Early Burst (EB) model (Harmon et al., 2010). For each increment of bone length (5-95%), the best fitting model is highlighted in bold and blue.

|  | **Femoral CSA** | | | | | | | | | | | | | | | | | | |
| --- | --- | --- | --- | --- | --- | --- | --- | --- | --- | --- | --- | --- | --- | --- | --- | --- | --- | --- | --- |
| **Model** | **5** | **10** | **15** | **20** | **25** | **30** | **35** | **40** | **45** | **50** | **55** | **60** | **65** | **70** | **75** | **80** | **85** | **90** | **95** |
| BM1 | 0.0 | 0.0 | 0.0 | 0.0 | 0.0 | 0.0 | 0.0 | 0.0 | 0.0 | 0.0 | 0.0 | 0.0 | 0.0 | 0.0 | 0.0 | 0.0 | 0.0 | 0.0 | 0.0 |
| OU1 | 0.0 | 0.0 | 0.0 | 0.0 | 0.0 | 0.0 | 0.0 | 0.0 | 0.0 | 0.0 | 0.0 | 0.0 | 0.0 | 0.0 | 0.0 | 0.0 | 0.0 | 0.0 | 0.0 |
| BM3 | **95.0** | **91.2** | **87.6** | **90.1** | **87.0** | **90.0** | **92.4** | **93.2** | **91.1** | **90.0** | **80.9** | **81.6** | **83.3** | **86.1** | **79.3** | **89.9** | **97.2** | **95.5** | **95.3** |
| OU3 | 0.0 | 0.0 | 0.0 | 0.0 | 0.0 | 0.0 | 0.0 | 0.0 | 0.0 | 0.0 | 0.0 | 0.0 | 0.0 | 0.0 | 0.0 | 0.0 | 0.0 | 0.0 | 0.1 |
| BM3_r | 0.0 | 0.0 | 0.0 | 0.0 | 0.0 | 0.0 | 0.0 | 0.0 | 0.0 | 0.0 | 0.0 | 0.0 | 0.0 | 0.0 | 0.0 | 0.0 | 0.0 | 0.0 | 0.0 |
| OU3_r | 0.0 | 0.0 | 0.0 | 0.0 | 0.0 | 0.0 | 0.0 | 0.0 | 0.0 | 0.0 | 0.0 | 0.0 | 0.0 | 0.0 | 0.0 | 0.0 | 0.0 | 0.0 | 0.0 |
| BM4 | 5.0 | 8.8 | 12.4 | 9.9 | 13.0 | 10.0 | 7.6 | 6.8 | 8.9 | 10.0 | 19.1 | 18.4 | 16.7 | 13.9 | 20.7 | 10.1 | 2.8 | 4.5 | 4.5 |
| OU4 | 0.0 | 0.0 | 0.0 | 0.0 | 0.0 | 0.0 | 0.0 | 0.0 | 0.0 | 0.0 | 0.0 | 0.0 | 0.0 | 0.0 | 0.0 | 0.0 | 0.0 | 0.0 | 0.0 |
| EB | 0.0 | 0.0 | 0.0 | 0.0 | 0.0 | 0.0 | 0.0 | 0.0 | 0.0 | 0.0 | 0.0 | 0.0 | 0.0 | 0.0 | 0.0 | 0.0 | 0.0 | 0.0 | 0.0 |
|  | **Femoral SMA_ML_** | | | | | | | | | | | | | | | | | | |
| BM1 | 0.1 | 0.2 | 1.4 | 0.0 | 0.6 | 0.5 | 0.3 | 0.2 | 0.1 | 0.0 | 0.0 | 0.0 | 0.0 | 0.0 | 0.0 | 0.0 | 0.0 | 0.0 | 0.0 |
| OU1 | 0.0 | 0.1 | 0.4 | 0.0 | 0.2 | 0.1 | 0.1 | 0.0 | 0.0 | 0.0 | 0.0 | 0.0 | 0.0 | 0.0 | 0.0 | 0.0 | 0.0 | 0.0 | 0.0 |
| BM3 | 0.0 | 0.1 | 21.8 | 0.7 | **45.4** | **53.9** | 31.0 | **65.4** | **87.1** | **96.8** | **96.8** | **95.7** | **93.5** | **93.0** | **89.1** | **82.9** | **77.2** | 0.1 | 9.0 |
| OU3 | **73.3** | **56.2** | **58.2** | 24.4 | 9.5 | 15.1 | **36.2** | 20.7 | 5.5 | 0.2 | 0.0 | 0.0 | 0.0 | 0.0 | 0.2 | 7.9 | 11.2 | **56.4** | **55.1** |
| BM3_r | 0.0 | 0.1 | 0.1 | 0.0 | 0.5 | 0.3 | 0.2 | 0.1 | 0.1 | 0.0 | 0.0 | 0.0 | 0.0 | 0.0 | 0.0 | 0.0 | 0.0 | 0.0 | 0.0 |
| OU3_r | 0.1 | 0.3 | 0.1 | 0.0 | 0.1 | 0.1 | 0.0 | 0.0 | 0.0 | 0.0 | 0.0 | 0.0 | 0.0 | 0.0 | 0.0 | 0.0 | 0.0 | 0.0 | 0.0 |
| BM4 | 0.0 | 0.3 | 0.7 | 0.1 | 12.0 | 8.6 | 2.3 | 3.1 | 4.5 | 2.9 | 3.1 | 4.3 | 6.4 | 6.9 | 10.5 | 3.6 | 7.8 | 0.0 | 0.3 |
| OU4 | 26.5 | 42.6 | 16.8 | **74.6** | 31.6 | 21.4 | 29.7 | 10.3 | 2.7 | 0.1 | 0.0 | 0.0 | 0.0 | 0.0 | 0.1 | 5.5 | 3.9 | 43.4 | 35.6 |
| EB | 0.0 | 0.1 | 0.4 | 0.0 | 0.2 | 0.1 | 0.1 | 0.1 | 0.0 | 0.0 | 0.0 | 0.0 | 0.0 | 0.0 | 0.0 | 0.0 | 0.0 | 0.0 | 0.0 |
|  | **Femoral SMA_CC_** | | | | | | | | | | | | | | | | | | |
| BM1 | 0.6 | 1.1 | 0.0 | 0.3 | 0.0 | 0.0 | 0.0 | 0.0 | 0.0 | 0.0 | 0.0 | 0.0 | 0.0 | 0.0 | 0.0 | 0.0 | 0.0 | 0.0 | 1.0 |
| OU1 | 0.2 | 0.3 | 0.0 | 0.1 | 0.0 | 0.0 | 0.0 | 0.0 | 0.0 | 0.0 | 0.0 | 0.0 | 0.0 | 0.0 | 0.0 | 0.0 | 0.0 | 0.0 | 0.3 |
| BM3 | 29.6 | 19.1 | **94.7** | **86.9** | **92.3** | **94.4** | **95.5** | **96.2** | **92.9** | **91.6** | **92.4** | **94.5** | **95.4** | **95.7** | **93.4** | **92.0** | **84.4** | 0.1 | 19.3 |
| OU3 | **49.6** | 8.4 | 0.0 | 1.0 | 0.0 | 0.0 | 0.0 | 0.0 | 0.0 | 0.0 | 0.0 | 0.0 | 0.0 | 0.0 | 0.0 | 0.2 | 3.4 | **50.5** | **55.1** |
| BM3_r | 0.2 | 13.4 | 0.1 | 0.6 | 0.0 | 0.0 | 0.0 | 0.0 | 0.0 | 0.0 | 0.0 | 0.0 | 0.0 | 0.0 | 0.0 | 0.0 | 0.0 | 0.0 | 0.0 |
| OU3_r | 0.1 | 1.2 | 0.0 | 0.1 | 0.0 | 0.0 | 0.0 | 0.0 | 0.0 | 0.0 | 0.0 | 0.0 | 0.0 | 0.0 | 0.0 | 0.0 | 0.0 | 0.0 | 0.2 |
| BM4 | 1.9 | 10.4 | 5.1 | 8.3 | 7.7 | 5.6 | 4.5 | 3.8 | 7.1 | 8.4 | 7.6 | 5.5 | 4.6 | 4.2 | 6.6 | 7.6 | 8.4 | 0.0 | 0.7 |
| OU4 | 17.6 | **45.8** | 0.0 | 2.5 | 0.0 | 0.0 | 0.0 | 0.0 | 0.0 | 0.0 | 0.0 | 0.0 | 0.0 | 0.0 | 0.0 | 0.2 | 3.8 | 49.3 | 23.0 |
| EB | 0.2 | 0.3 | 0.0 | 0.2 | 0.0 | 0.0 | 0.0 | 0.0 | 0.0 | 0.0 | 0.0 | 0.0 | 0.0 | 0.0 | 0.0 | 0.0 | 0.0 | 0.0 | 0.4 |

**Table S2**. Akaike weights for trait diversification models fitted to tibial cross-sectional traits. Models fitted include group mean Brownian motion models with one, three, and four rates (BM1, BM3/BM3_r, and B4, respectively) and Ornstein-Uhlenbeck models with one, three, and four phenotypic optima (OU1, OU3/OU3_r, and OU4, respectively). BM3 and OU3 have their respective distinct rates and optima based upon three functional categories: scansoriality, natatoriality, and a third category combining fossorial and generalized mustelids. BM3_r and OU3_r have their respective weights and optima based upon three categories: scansoriality, generalist, and a third category combining natatorial and fossorial mustelids. BM4 and OU4 have their respective rates and optima based upon each of the four locomotor categories occurring within extant mustelids: fossorial, generalized, natatorial, and scansorial. Also fitted was an Early Burst (EB) model (Harmon et al., 2010). For each increment of bone length (5-95%), the best fitting model is highlighted in bold and blue.

|  | **Tibial CSA** | | | | | | | | | | | | | | | | | | |
| --- | --- | --- | --- | --- | --- | --- | --- | --- | --- | --- | --- | --- | --- | --- | --- | --- | --- | --- | --- |
| **Model** | **5** | **10** | **15** | **20** | **25** | **30** | **35** | **40** | **45** | **50** | **55** | **60** | **65** | **70** | **75** | **80** | **85** | **90** | **95** |
| BM1 | 0.7 | **29.1** | **30.9** | **32.4** | **28.5** | 23.0 | 21.8 | 20.7 | 22.4 | **23.7** | **26.8** | **29.1** | **22.4** | 15.9 | 17.5 | 13.7 | 10.8 | 9.6 | 0.1 |
| OU1 | 0.6 | 8.3 | 8.8 | 9.2 | 8.1 | 6.5 | 6.2 | 5.9 | 6.3 | 6.7 | 7.6 | 8.3 | 6.4 | 4.5 | 5.0 | 3.9 | 3.1 | 2.7 | **48.2** |
| BM3 | 0.0 | 3.7 | 2.4 | 3.5 | 2.9 | 2.9 | 3.1 | 4.5 | 6.7 | 11.3 | 15.8 | 13.6 | 10.5 | 8.4 | 7.1 | 12.0 | **25.1** | 5.4 | 0.0 |
| OU3 | 1.7 | 17.7 | 11.1 | 12.1 | 12.9 | 9.5 | 9.6 | 9.9 | 10.7 | 10.2 | 9.3 | 9.0 | 12.4 | 18.9 | 19.2 | **23.0** | 22.5 | 31.8 | 4.9 |
| BM3_r | 0.1 | 4.2 | 4.7 | 4.0 | 4.3 | 5.1 | 4.2 | 4.7 | 4.6 | 5.3 | 6.4 | 4.8 | 4.9 | 4.7 | 5.1 | 4.5 | 4.1 | 1.5 | 0.0 |
| OU3_r | **60.1** | 18.0 | 17.5 | 19.5 | 23.3 | **27.7** | **26.8** | 21.6 | 17.8 | 14.7 | 11.9 | 12.9 | 17.3 | **21.0** | **21.7** | 17.4 | 10.1 | 7.6 | 2.9 |
| BM4 | 0.0 | 0.2 | 0.4 | 0.2 | 0.2 | 0.5 | 0.5 | 0.8 | 0.9 | 1.5 | 1.6 | 1.7 | 1.5 | 1.0 | 0.9 | 1.4 | 2.0 | 0.3 | 0.0 |
| OU4 | 36.1 | 8.5 | 8.8 | 9.7 | 10.9 | 16.3 | 20.9 | **25.4** | **23.2** | 18.0 | 10.5 | 8.8 | 12.0 | 17.0 | 16.3 | 18.0 | 18.3 | **38.1** | 1.0 |
| EB | 0.6 | 10.2 | 15.3 | 9.4 | 8.9 | 8.4 | 6.7 | 6.6 | 7.3 | 8.5 | 10.0 | 11.8 | 12.6 | 8.5 | 7.5 | 6.1 | 3.9 | 2.8 | 42.8 |
|  | **Tibial SMA_ML_** | | | | | | | | | | | | | | | | | | |
| BM1 | 4.3 | **40.7** | **33.3** | **40.8** | **40.2** | **33.0** | **34.0** | **35.1** | **36.3** | **39.4** | **43.0** | **44.8** | **45.2** | **40.3** | **33.8** | **24.2** | **23.2** | 14.7 | 1.4 |
| OU1 | 1.8 | 11.5 | 9.4 | 11.6 | 11.4 | 9.4 | 9.7 | 10.0 | 10.3 | 11.2 | 12.2 | 12.7 | 12.8 | 11.4 | 9.6 | 6.9 | 6.6 | 4.2 | 42.3 |
| BM3 | 1.1 | 3.5 | 3.2 | 2.9 | 2.2 | 2.0 | 2.7 | 3.5 | 4.1 | 3.7 | 4.3 | 3.4 | 2.7 | 2.6 | 3.1 | 5.3 | 13.2 | 6.1 | 0.0 |
| OU3 | 8.1 | 10.4 | 8.6 | 9.0 | 8.0 | 7.4 | 8.2 | 7.8 | 8.1 | 7.8 | 6.5 | 6.3 | 6.8 | 8.3 | 8.5 | 9.7 | 11.2 | 19.6 | 4.9 |
| BM3_r | 1.4 | 4.6 | 3.7 | 3.8 | 3.6 | 4.5 | 5.7 | 6.6 | 6.2 | 5.4 | 5.2 | 3.6 | 2.5 | 2.2 | 2.9 | 3.6 | 2.7 | 2.2 | 0.0 |
| OU3_r | **43.3** | 12.7 | 13.1 | 11.9 | 14.2 | 17.8 | 16.3 | 14.9 | 13.6 | 12.1 | 10.2 | 10.8 | 11.6 | 15.6 | 20.5 | 21.5 | 13.8 | 13.6 | 6.6 |
| BM4 | 0.1 | 0.2 | 0.2 | 0.2 | 0.2 | 0.3 | 0.3 | 0.3 | 0.3 | 0.3 | 0.3 | 0.3 | 0.3 | 0.3 | 0.4 | 1.1 | 1.9 | 0.6 | 0.0 |
| OU4 | 38.1 | 3.9 | 5.7 | 5.2 | 6.2 | 8.5 | 8.5 | 9.3 | 8.9 | 7.4 | 5.2 | 4.8 | 4.9 | 7.8 | 11.6 | 20.9 | 20.7 | **34.4** | 2.5 |
| EB | 1.8 | 12.6 | 22.7 | 14.7 | 13.8 | 17.1 | 14.6 | 12.5 | 12.2 | 12.8 | 13.1 | 13.3 | 13.3 | 11.5 | 9.6 | 6.9 | 6.8 | 4.6 | **42.3** |
|  | **Tibial SMA_CC_** | | | | | | | | | | | | | | | | | | |
| BM1 | 14.8 | **29.2** | **27.7** | 27.1 | **31.7** | **33.5** | **30.5** | 19.3 | 12.3 | 9.9 | 11.2 | 13.5 | 18.3 | **22.6** | **25.2** | **21.1** | **19.0** | 9.4 | 0.6 |
| OU1 | 4.2 | 8.3 | 7.9 | 7.7 | 9.0 | 9.5 | 8.6 | 5.5 | 3.5 | 2.8 | 3.2 | 3.8 | 5.2 | 6.4 | 7.1 | 6.0 | 5.4 | 2.7 | **46.6** |
| BM3 | 2.6 | 5.1 | 2.0 | 1.7 | 2.0 | 2.8 | 7.0 | **28.3** | **43.8** | **51.9** | **54.8** | **41.2** | **22.7** | 9.8 | 6.7 | 12.6 | 17.7 | 4.2 | 0.1 |
| OU3 | 13.4 | 11.8 | 7.6 | 6.5 | 5.3 | 3.8 | 3.8 | 2.5 | 2.0 | 2.1 | 2.4 | 3.8 | 6.1 | 10.3 | 10.5 | 11.2 | 13.6 | **27.5** | 3.7 |
| BM3_r | 3.4 | 6.8 | 6.2 | 4.0 | 4.5 | 4.9 | 5.7 | 11.8 | 10.4 | 8.7 | 6.9 | 7.2 | 7.8 | 6.2 | 5.4 | 6.1 | 4.6 | 4.1 | 0.0 |
| OU3_r | 32.4 | 20.4 | 25.4 | **34.6** | 29.5 | 26.1 | 23.4 | 10.3 | 5.2 | 3.9 | 4.1 | 7.1 | 13.0 | 19.9 | 21.7 | 19.4 | 17.5 | 26.9 | 2.9 |
| BM4 | 0.2 | 0.3 | 0.2 | 0.2 | 0.2 | 0.6 | 1.6 | 10.8 | 15.2 | 13.9 | 9.3 | 9.5 | 5.6 | 1.7 | 1.0 | 2.0 | 1.3 | 0.3 | 0.0 |
| OU4 | **24.6** | 5.8 | 8.5 | 10.5 | 8.8 | 9.3 | 10.7 | 6.0 | 3.9 | 3.2 | 2.7 | 4.1 | 7.0 | 11.0 | 13.2 | 14.3 | 15.1 | 22.1 | 1.1 |
| EB | 4.3 | 12.4 | 14.5 | 7.7 | 9.1 | 9.6 | 8.7 | 5.6 | 3.7 | 3.6 | 5.4 | 9.8 | 14.3 | 12.0 | 9.3 | 7.3 | 5.9 | 2.7 | 44.9 |

**Table S3**. Akaike weights for trait diversification models fitted to fibular cross-sectional traits. Models fitted include group mean Brownian motion models with one, three, and four rates (BM1, BM3/BM3_r, and B4, respectively) and Ornstein-Uhlenbeck models with one, three, and four phenotypic optima (OU1, OU3/OU3_r, and OU4, respectively). BM3 and OU3 have their respective distinct rates and optima based upon three functional categories: scansoriality, natatoriality, and a third category combining fossorial and generalized mustelids. BM3_r and OU3_r have their respective weights and optima based upon three categories: scansoriality, generalist, and a third category combining natatorial and fossorial mustelids. BM4 and OU4 have their respective rates and optima based upon each of the four locomotor categories occurring within extant mustelids: fossorial, generalized, natatorial, and scansorial. Also fitted was an Early Burst (EB) model (Harmon et al., 2010). For each increment of bone length (5-95%), the best fitting model is highlighted in bold and blue.

|  | **Fibular CSA** | | | | | | | | | | | | | | | | | | |
| --- | --- | --- | --- | --- | --- | --- | --- | --- | --- | --- | --- | --- | --- | --- | --- | --- | --- | --- | --- |
| **Model** | **5** | **10** | **15** | **20** | **25** | **30** | **35** | **40** | **45** | **50** | **55** | **60** | **65** | **70** | **75** | **80** | **85** | **90** | **95** |
| BM1 | 0.5 | 6.2 | 9.1 | 9.2 | 9.1 | 11.4 | 15.2 | 14.7 | 9.2 | 4.4 | 2.5 | 2.0 | 1.6 | 2.2 | 1.4 | 0.2 | 0.0 | 1.1 | 1.3 |
| OU1 | 0.1 | 1.8 | 2.6 | 2.6 | 2.6 | 3.2 | 4.3 | 4.2 | 2.6 | 1.2 | 0.7 | 0.6 | 0.5 | 0.7 | 0.6 | 0.1 | 0.0 | 0.3 | 0.4 |
| BM3 | 2.4 | 10.2 | 4.8 | 3.9 | 8.3 | 4.4 | 4.3 | 3.9 | 5.4 | 2.4 | 2.6 | 2.2 | 1.0 | 1.5 | 4.3 | **82.0** | **97.1** | **45.8** | 13.1 |
| OU3 | 8.5 | 12.2 | 8.0 | 6.4 | 4.9 | 7.5 | 8.2 | 9.7 | 12.2 | 8.3 | 4.6 | 2.8 | 2.0 | 4.8 | 11.5 | 3.4 | 0.1 | 22.5 | **52.1** |
| BM3_r | 0.6 | **26.4** | 15.6 | 9.9 | 15.8 | 13.6 | 11.1 | 7.7 | 7.7 | 4.1 | 5.4 | 2.2 | 0.6 | 0.5 | 0.2 | 0.2 | 0.0 | 7.6 | 5.6 |
| OU3_r | **61.1** | 24.3 | **35.9** | **46.5** | **42.8** | **40.3** | **38.8** | **36.5** | 25.8 | 34.8 | 36.5 | 35.6 | 43.8 | 31.5 | 13.9 | 0.6 | 0.0 | 3.0 | 1.5 |
| BM4 | 0.1 | 1.0 | 0.7 | 0.4 | 1.2 | 0.5 | 0.3 | 0.3 | 0.5 | 0.3 | 0.6 | 0.5 | 0.2 | 0.2 | 1.1 | 9.2 | 2.8 | 2.2 | 0.6 |
| OU4 | 26.4 | 8.3 | 18.7 | 16.5 | 11.3 | 11.1 | 10.6 | 17.7 | **32.1** | **42.5** | **45.8** | **53.3** | **49.8** | **57.8** | **66.4** | 4.1 | 0.0 | 17.2 | 25.0 |
| EB | 0.1 | 9.8 | 4.8 | 4.6 | 4.0 | 8.0 | 7.1 | 5.3 | 4.5 | 2.0 | 1.3 | 0.7 | 0.5 | 0.7 | 0.6 | 0.1 | 0.0 | 0.3 | 0.4 |
|  | **Fibular SMA_ML_** | | | | | | | | | | | | | | | | | | |
| BM1 | 0.1 | 5.6 | 17.0 | 16.8 | **25.7** | 11.3 | 12.9 | 18.3 | 10.5 | 3.9 | 0.7 | 0.2 | 0.1 | 0.2 | 0.6 | 0.0 | 0.0 | 2.0 | 3.8 |
| OU1 | 0.0 | 1.6 | 4.8 | 4.8 | 7.3 | 3.2 | 3.7 | 5.2 | 3.0 | 1.1 | 0.3 | 0.1 | 0.1 | 0.2 | 1.0 | 0.1 | 0.0 | 0.6 | 1.1 |
| BM3 | 0.2 | **60.2** | 2.6 | 4.1 | 4.0 | 13.6 | 28.4 | 17.3 | 9.8 | 2.6 | 2.7 | 2.2 | 0.8 | 2.0 | 19.3 | **88.5** | **98.0** | **67.9** | 3.3 |
| OU3 | **67.9** | 4.0 | 11.4 | 10.0 | 8.7 | 3.8 | 3.3 | 7.0 | 10.3 | 7.6 | 1.9 | 0.6 | 0.4 | 1.4 | 8.1 | 3.3 | 0.0 | 4.9 | 15.4 |
| BM3_r | 0.1 | 17.6 | 2.9 | 4.5 | 5.8 | **40.1** | **30.3** | **20.6** | 14.4 | 3.3 | 2.0 | 1.3 | 0.1 | 0.1 | 0.1 | 0.1 | 0.0 | 12.8 | 3.5 |
| OU3_r | 11.3 | 4.3 | **33.3** | **35.7** | 25.2 | 11.6 | 8.1 | 18.1 | **29.6** | **51.2** | **67.5** | **74.3** | **76.6** | **66.4** | **36.8** | 0.8 | 0.0 | 4.2 | **44.6** |
| BM4 | 0.0 | 2.1 | 0.2 | 0.2 | 0.5 | 1.6 | 2.1 | 0.9 | 0.8 | 0.2 | 0.5 | 0.9 | 0.2 | 0.5 | 2.9 | 5.5 | 1.9 | 3.2 | 0.2 |
| OU4 | 20.3 | 3.0 | 23.0 | 19.0 | 13.4 | 3.3 | 2.1 | 7.3 | 18.7 | 29.0 | 24.2 | 20.1 | 21.7 | 28.9 | 30.2 | 1.7 | 0.0 | 3.9 | 26.5 |
| EB | 0.0 | 1.6 | 4.8 | 4.9 | 9.6 | 11.4 | 9.2 | 5.3 | 3.0 | 1.1 | 0.3 | 0.1 | 0.1 | 0.2 | 1.0 | 0.1 | 0.0 | 0.6 | 1.6 |
|  | **Fibular SMA_CC_** | | | | | | | | | | | | | | | | | | |
| BM1 | 1.3 | 0.0 | 2.9 | 11.4 | **25.5** | 16.8 | 18.9 | 21.4 | **24.2** | 14.0 | 6.7 | 4.1 | 4.3 | 2.4 | 0.5 | 2.6 | 0.2 | 0.1 | 0.6 |
| OU1 | 0.4 | 0.0 | 0.8 | 3.2 | 7.3 | 4.9 | 5.6 | 6.6 | 6.9 | 4.0 | 1.9 | 1.3 | 1.6 | 1.1 | 0.3 | 1.0 | 0.1 | 0.1 | 0.2 |
| BM3 | 1.0 | 0.0 | 3.6 | 9.5 | 2.3 | 1.5 | 2.7 | 1.3 | 3.4 | 3.4 | 1.1 | 0.6 | 0.2 | 0.1 | 0.1 | 2.8 | **88.6** | **81.0** | 5.9 |
| OU3 | 1.8 | 0.0 | 0.9 | 4.3 | 5.4 | 7.7 | 9.1 | 13.4 | 16.1 | 15.4 | 8.3 | 8.8 | 7.7 | 16.8 | 30.5 | 30.8 | 0.2 | 3.9 | **64.4** |
| BM3_r | 2.1 | **99.7** | **72.7** | 21.6 | 16.2 | 2.3 | 2.6 | 1.9 | 2.8 | 1.8 | 1.4 | 0.5 | 0.4 | 0.4 | 0.1 | 0.4 | 0.4 | 5.4 | 4.9 |
| OU3_r | **75.2** | 0.0 | 6.7 | **24.5** | 21.2 | **47.3** | **46.0** | **35.1** | 21.1 | 28.1 | 33.1 | 36.0 | **46.9** | 30.6 | 8.5 | 8.8 | 0.1 | 1.8 | 1.3 |
| BM4 | 0.1 | 0.2 | 3.0 | 1.4 | 0.4 | 0.1 | 0.1 | 0.1 | 0.2 | 0.4 | 0.4 | 0.2 | 0.2 | 0.1 | 0.1 | 12.9 | 10.0 | 4.7 | 0.2 |
| OU4 | 17.7 | 0.0 | 2.9 | 10.1 | 14.4 | 14.7 | 9.4 | 13.5 | 18.4 | **28.9** | **45.2** | **47.2** | 37.2 | **47.3** | **59.7** | **39.8** | 0.3 | 2.9 | 22.4 |
| EB | 0.4 | 0.1 | 6.6 | 14.1 | 7.3 | 4.9 | 5.6 | 6.6 | 6.9 | 4.0 | 1.9 | 1.3 | 1.6 | 1.1 | 0.3 | 1.0 | 0.1 | 0.1 | 0.2 |
