# Supplementary material for: Functional Morphology and Morphological Diversification of Hind Limb Cross-Sectional Traits in Mustelid Mammals
Source: Integr Org Biol. 2020 Jan 8;2(1):obz032. doi: 10.1093/iob/obz032 (PMC7671153; doi:10.1093/iob/obz032)
Supplement: obz032_Supplementary_Data [file obz032_supplementary_data.zip › Tables S4-S6 Revised.docx]

|  | | | | | | | | |
| --- | --- | --- | --- | --- | --- | --- | --- | --- |
| **Increment** | **Model** | **α** | **Lower C.L** | **Upper C.L.** | **t** | **t_Height_** | **t_Mean_** | **t_Min_** |
| **Femoral SMA_ML_** | | | | | | | | |
| **5** | **OU3** | **2.03** | **0.204** | **15.141** | **0.342** | **0.019** | **0.087** | **0.834** |
| **10** | **OU3** | **2.03** | **0.191** | **15.592** | **0.342** | **0.019** | **0.087** | **0.834** |
| **15** | **OU3** | **1.13** | **0.175** | **15.160** | **0.611** | **0.034** | **0.156** | **1.491** |
| **20** | **OU4** | **2.02** | **0.208** | **19.947** | **0.343** | **0.019** | **0.088** | **0.837** |
| **35** | **OU3** | **1.38** | **0.189** | **15.334** | **0.501** | **0.028** | **0.128** | **1.221** |
| **90** | **OU3** | **2.16** | **0.206** | **15.280** | **0.320** | **0.018** | **0.082** | **0.781** |
| **95** | **OU3** | **1.31** | **0.207** | **15.224** | **0.528** | **0.030** | **0.135** | **1.287** |
| **Femoral SMA_CC_** | | | | | | | | |
| **5** | **OU3** | **1.74** | **0.165** | **15.238** | **0.399** | **0.022** | **0.102** | **0.973** |
| **10** | **OU4** | **2.90** | **0.191** | **19.946** | **0.238** | **0.013** | **0.061** | **0.583** |
| **90** | **OU3** | **1.78** | **0.196** | **15.449** | **0.390** | **0.022** | **0.100** | **0.951** |
| **95** | **OU3** | **1.41** | **0.165** | **15.155** | **0.491** | **0.028** | **0.126** | **1.199** |
|  |  |  |  |  |  |  |  |  |

**Table S4**. α values and phylogenetic half-lives for OU models that were determined to be the best fitting model for femoral cross-sectional traits. ‘Increment’ denotes the sections along the femur’s length at which traits were measured, expressed as the percentage of femoral length relative to the femur’s proximal end. For alpha – the strength of selection – if upper and lower confidence limits include a value of 0.0, then the estimate of alpha does not significantly differ from a value of 0.0, and the model therefore cannot be distinguished from Brownian motion (Cooper et al., 2016). t represents phylogenetic half life (= ln(2)/α). To gauge the magnitude of phylogenetic half-life relative to the sampled phylogeny (Cooper et al., 2016), phylogenetic half life is also expressed as a fraction of tree height, mean branch length, and minimum branch length (t_Height_, t_Mean_, and t_Min_, respectively). Values highlighted in bold and blue denote significant values of alpha.

**Table S5**. α values and phylogenetic half-lives for OU models that were determined to be the best fitting model for tibial cross-sectional traits. ‘Increment’ denotes the sections along the tibia’s length at which traits were measured, expressed as the percentage of tibial length relative to the tibia’s proximal end. For alpha – the strength of selection – if upper and lower confidence limits include a value of 0.0, then the estimate of alpha does not significantly differ from a value of 0.0, and the model therefore cannot be distinguished from Brownian motion (Cooper et al., 2016). t represents phylogenetic half life (= ln(2)/α). To gauge the magnitude of phylogenetic half-life relative to the sampled phylogeny (Cooper et al., 2016), phylogenetic half life is also expressed as a fraction of tree height, mean branch length, and minimum branch length (t_Height_, t_Mean_, and t_Min_, respectively). Values highlighted in bold and blue denote significant values of alpha.

|  | | | | | | | | |
| --- | --- | --- | --- | --- | --- | --- | --- | --- |
| **Increment** | **Model** | **α** | **Lower C.L** | **Upper C.L.** | **t** | **t_Height_** | **t_Mean_** | **t_Min_** |
| **Tibial CSA** | | | | | | | | |
| **5** | **OU3_r** | **5.02** | **0.165** | **19.948** | **0.138** | **0.008** | **0.035** | **0.337** |
| 30 | OU3_r | 0.03 | < 0.0001 | 19.928 | 23.105 | 1.299 | 5.900 | 56.354 |
| 35 | OU3_r | 0.05 | < 0.0001 | 19.942 | 13.863 | 0.779 | 3.540 | 33.812 |
| **40** | **OU4** | **0.07** | **0.021** | **16.783** | **9.735** | **0.547** | **2.486** | **23.744** |
| **45** | **OU4** | **0.07** | **0.026** | **15.421** | **9.938** | **0.559** | **2.538** | **24.240** |
| 70 | OU3_r | 0.01 | < 0.0001 | 2.645 | 69.315 | 3.896 | 17.701 | 43.173 |
| 75 | OU3_r | 0.01 | < 0.0001 | 19.105 | 69.315 | 3.896 | 17.701 | 43.173 |
| 80 | OU3 | 0.00 | < 0.0001 | 0.412 | 3335.838 | 187.512 | 851.864 | 8136.189 |
| **90** | **OU4** | **0.42** | **0.093** | **19.929** | **1.658** | **0.093** | **0.424** | **4.045** |
| 95 | OU1 | 0.95 | -2.804 | 4.697 | 0.732 | 0.041 | 0.187 | 1.786 |
| **Tibial SMA_ML_** | | | | | | | | |
| **5** | **OU3_r** | **0.42** | **0.022** | **19.946** | **1.650** | **0.093** | **0.421** | **4.024** |
| **90** | **OU4** | **0.15** | **0.046** | **19.396** | **4.602** | **0.259** | **1.175** | **11.224** |
| **Tibial SMA_CC_** | | | | | | | | |
| **5** | **OU4** | **0.13** | **0.033** | **19.549** | **5.266** | **0.296** | **1.345** | **12.844** |
| 20 | OU3_r | 0.07 | < 0.0001 | 19.942 | 9.902 | 0.557 | 2.529 | 24.151 |
| 90 | OU3 | 0.04 | < 0.0001 | 0.904 | 19.210 | 1.080 | 4.906 | 46.853 |
| 95 | OU1 | 0.75 | -2.830 | 4.333 | 0.922 | 0.052 | 0.236 | 2.250 |

**Table S6**. α values and phylogenetic half-lives for OU models that were determined to be the best fitting model for fibular cross-sectional traits. ‘Increment’ denotes the sections along the fibula’s length at which traits were measured, expressed as the percentage of fibular length relative to the fibula’s proximal end. For alpha – the strength of selection – if upper and lower confidence limits include a value of 0.0, then the estimate of alpha does not significantly differ from a value of 0.0, and the model therefore cannot be distinguished from Brownian motion (Cooper et al., 2016). t represents phylogenetic half life (= ln(2)/α). To gauge the magnitude of phylogenetic half-life relative to the sampled phylogeny (Cooper et al., 2016), phylogenetic half-life is also expressed as a fraction of tree height, mean branch length, and minimum branch length (t_Height_, t_Mean_, and t_Min_, respectively). Values highlighted in bold and blue denote significant values of alpha.

|  | | | | | | | | | | |
| --- | --- | --- | --- | --- | --- | --- | --- | --- | --- | --- |
| **Increment** | **Model** | **α** | **Lower C.L** | | **Upper C.L.** | **t** | **t_Height_** | | **t_Mean_** | **t_Min_** |
| **Fibular CSA** | | | | | | | | | | |
| **5** | **OU3_r** | **0.38** | **0.128** | | **19.935** | **1.824** | **0.103** | | **0.466** | **4.449** |
| 15 | OU3_r | 0.05 | < 0.0001 | | 19.910 | 13.863 | 0.779 | | 3.540 | 33.812 |
| 20 | OU3_r | 0.05 | < 0.0001 | | 19.811 | 13.863 | 0.779 | | 3.540 | 33.812 |
| 25 | OU3_r | 0.07 | < 0.0001 | | 19.742 | 9.902 | 0.557 | | 2.529 | 24.151 |
| 30 | OU3_r | 0.02 | < 0.0001 | | 8.019 | 34.657 | 1.948 | | 8.850 | 84.529 |
| 35 | OU3_r | 0.06 | < 0.0001 | | 19.801 | 11.552 | 0.649 | | 2.950 | 28.176 |
| 40 | OU3_r | 0.08 | < 0.0001 | | 19.940 | 8.664 | 0.487 | | 2.213 | 21.132 |
| **45** | **OU4** | **0.15** | **0.085** | | **19.813** | **4.721** | **0.265** | | **1.206** | **11.514** |
| **50** | **OU4** | **0.22** | **0.102** | | **19.923** | **3.211** | **0.181** | | **0.820** | **7.833** |
| **55** | **OU4** | **0.26** | **0.113** | | **19.849** | **2.692** | **0.151** | | **0.688** | **6.568** |
| **60** | **OU4** | **0.36** | **0.115** | | **19.921** | **1.944** | **0.109** | | **0.496** | **4.741** |
| **65** | **OU4** | **0.62** | **0.147** | | **19.945** | **1.124** | **0.063** | | **0.287** | **2.743** |
| **70** | **OU4** | **0.55** | **0.123** | | **19.944** | **1.269** | **0.071** | | **0.324** | **3.096** |
| **75** | **OU4** | **1.39** | **0.154** | | **19.946** | **0.498** | **0.028** | | **0.127** | **1.214** |
| **95** | **OU3** | **0.23** | **0.122** | | **9.692** | **2.967** | **0.167** | | **0.758** | **7.236** |
| **Fibular SMA_ML_** | | | | | | | | | | |
| **5** | **OU3** | **0.26** | | **0.129** | **13.774** | **2.672** | **0.150** | | **0.682** | **6.517** |
| **15** | **OU3_r** | **0.22** | | **0.035** | **19.941** | **3.151** | **0.177** | | **0.804** | **7.685** |
| **20** | **OU3_r** | **0.14** | | **0.020** | **19.932** | **4.951** | **0.278** | | **1.264** | **12.076** |
| **45** | **OU3_r** | **0.27** | | **0.016** | **19.940** | **2.567** | **0.144** | | **0.656** | **6.261** |
| **50** | **OU3_r** | **0.38** | | **0.030** | **19.945** | **1.824** | **0.103** | | **0.466** | **4.449** |
| **55** | **OU3_r** | **0.89** | | **0.038** | **19.946** | **0.779** | **0.044** | | **0.199** | **1.9** |
| **60** | **OU3_r** | **1.59** | | **0.075** | **19.948** | **0.436** | **0.025** | | **0.111** | **1.063** |
| **65** | **OU3_r** | **1.86** | | **0.150** | **19.948** | **0.373** | **0.021** | | **0.095** | **0.909** |
| **70** | **OU3_r** | **1.92** | | **0.124** | **19.947** | **0.361** | **0.020** | | **0.092** | **0.881** |
| 95 | OU3_r | 0.13 | | < 0.0001 | 19.994 | 5.332 | 0.300 | | 1.362 | 13.005 |
| **Fibular SMA_CC_** | | | | | | | | | | |
| **5** | **OU3_r** | **0.56** | | **0.054** | **19.947** | **1.238** | | **0.070** | **0.316** | **3.019** |
| 20 | OU3_r | 0.00 | | < 0.0001 | 0.616 | 41755.85 | | 2347.153 | 10663.08 | 101843.5 |
| **30** | **OU3_r** | **0.15** | | **0.026** | **19.926** | **4.621** | | **0.260** | **1.180** | **11.271** |
| **35** | **OU3_r** | **0.13** | | **0.026** | **19.918** | **5.332** | | **0.300** | **1.362** | **13.005** |
| **40** | **OU3_r** | **0.13** | | **0.022** | **19.939** | **5.332** | | **0.300** | **1.362** | **13.005** |
| **50** | **OU4** | **0.18** | | **0.095** | **19.939** | **3.890** | | **0.219** | **0.993** | **9.489** |
| **55** | **OU4** | **0.32** | | **0.121** | **19.935** | **2.172** | | **0.122** | **0.555** | **5.297** |
| **60** | **OU4** | **0.42** | | **0.144** | **19.943** | **1.660** | | **0.093** | **0.424** | **4.049** |
| **65** | **OU3_r** | **0.41** | | **0.098** | **19.944** | **1.691** | | **0.095** | **0.432** | **4.123** |
| **70** | **OU4** | **0.63** | | **0.145** | **19.942** | **1.099** | | **0.062** | **0.281** | **2.680** |
| **75** | **OU4** | **1.94** | | **0.194** | **19.947** | **0.358** | | **0.020** | **0.091** | **0.872** |
| **80** | **OU4** | **0.88** | | **0.163** | **19.946** | **0.786** | | **0.044** | **0.201** | **1.917** |
| **95** | **OU3** | **0.40** | | **0.146** | **14.442** | **1.728** | | **0.097** | **0.441** | **4.215** |
